# Control of dynamic *sp*^3^-C stereochemistry

**DOI:** 10.1038/s41557-023-01156-7

**Published:** 2023-03-13

**Authors:** Aisha N. Bismillah, Toby G. Johnson, Burhan A. Hussein, Andrew T. Turley, Promeet K. Saha, Ho Chi Wong, Juan A. Aguilar, Dmitry S. Yufit, Paul R. McGonigal

**Affiliations:** 1 Department of Chemistry, Durham University, Durham, UK; 2 Department of Chemistry, University of York, York, UK

**Keywords:** Stereochemistry, Coordination chemistry, Supramolecular chemistry

## Abstract

Stereogenic *sp*^3^-hybridized carbon centres are fundamental building blocks of chiral molecules. Unlike dynamic stereogenic motifs, such as *sp*^3^-nitrogen centres or atropisomeric biaryls, *sp*^3^-carbon centres are usually fixed, requiring intermolecular reactions to undergo configurational changes. Here we report the internal enantiomerization of fluxional carbon cages and the consequences of their adaptive configurations for the transmission of stereochemical information. The *sp*^3^-carbon stereochemistry of the rigid tricyclic cages is inverted through strain-assisted Cope rearrangements, emulating the low-barrier configurational dynamics typical for *sp*^3^-nitrogen inversion or conformational isomerism. This dynamic enantiomerization can be stopped, restarted or slowed by external reagents, while the configuration of the cage is controlled by neighbouring, fixed stereogenic centres. As part of a phosphoramidite–olefin ligand, the fluxional cage acts as a conduit to transmit stereochemical information from the ligand while also transferring its dynamic properties to chiral-at-metal coordination environments, influencing catalysis, ion pairing and ligand exchange energetics.

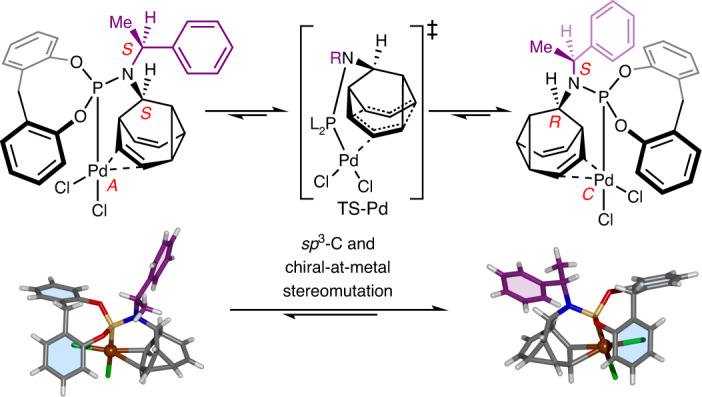

## Abstract (web summary)

Stereogenic *sp*^3^-hybridized carbon centres are the principal building blocks of chiral organic molecules. Usually, these centres are configurationally fixed. Now, low-energy pericyclic rearrangements have been used to create rigid cage molecules with fluxional *sp*^3^-stereochemistry, influencing chiral information transfer. The *sp*^3^-carbon stereochemistry of the cages is inverted through strain-assisted Cope rearrangements.

## Main

The hugely varied three-dimensional (3D) structures—and therefore the hugely varied properties—of many organic molecules emerge from combining just a few types of atomic building blocks. For example, 19 of the 22 proteinogenic amino acids are formed solely from *sp*^2^- or *sp*^3^-hybridized carbon, nitrogen and oxygen atoms, capped by hydrogen substituents. Of this small array of elemental building blocks, it is tetrahedral *sp*^3^-carbon^1–4^ and *sp*^3^-nitrogen^5–10^ atoms that have the potential to form stereogenic centres, creating chiral structures.

Chirality also arises in organic molecules by virtue of motifs other than stereogenic atoms. However, although stereochemical inversion of some planar chiral motifs^11–13^, helices^14–16^ and stereogenic *sp*^3^-nitrogen centres^7–10^ can occur rapidly and reversibly through low-barrier conformational isomerism, *sp*^3^-carbon centres cannot generally undergo spontaneous stereochemical changes. For example, the energy barrier to pyramidal inversion of methane is greater than its C–H bond dissociation energy^17–19^. Accordingly, unlike other stereogenic motifs^11–16^, *sp*^3^-carbon centres cannot generally adapt to surrounding chiral moieties and cannot be controllably switched by the application of external stimuli.

Instead, intermolecular reactions are usually necessary^20,21^ to invert individual stereogenic carbon centres, proceeding through mechanisms involving high-energy bond-breaking and bond-making steps^22^ with pentavalent transition states^20^ (for example, S_N_2 reactions) or trigonal intermediates^21^, such as carbocations, carbanions or radicals. Of course, it is this stability of *sp*^3^-carbon’s tetrahedral geometry that makes it essential to the chiral skeletal diversity of organic compounds. It allows for predictable synthesis of configurationally stable molecules. Yet, the stability also limits the extent to which the complex 3D connectivity of aliphatic structures can exhibit dynamic, adaptive stereochemistry^23^.

There have been impressive, but rare, examples of small covalent systems^24–28^ capable of *sp*^3^-carbon enantiomerization by low-barrier intramolecular processes. However, they do so without external control of their rate or direction to a single stereoisomer. Only multicomponent interlocked molecules, in which a ring shuttles along a prochiral axle^29,30^, have been amenable to external control. There have been no compact and controllable dynamic *sp*^3^-carbon building blocks. Therefore, it has not been possible to investigate the transmission of stereochemical information through such systems^7,9,10^.

In this Article we report a series of chiral fluxional carbon cages^26–29^ that exhibit responsive *sp*^3^-carbon-centred stereochemistry, adapting to and transmitting surrounding stereochemical information. By applying density functional theory (DFT) calculations and solution- and solid-state NMR spectroscopy, in combination with X-ray crystallography, we establish the extent to which their dynamic Cope rearrangements^31,32^ are controlled by neighbouring, fixed stereogenic centres. We have found that a substantial energetic bias of more than 10 kJ mol^−1^ can be exercised over the stereochemical equilibria by a single fixed stereocentre. The rearrangements proceed rapidly at rates more commonly associated with low-barrier conformational changes of aliphatic systems (for example, a cyclohexane ring-flip energy barrier of ~43 kJ mol^−1^) rather than a configurational change. We show that these rapid constitutional dynamics can be halted by covalent modification of the cage through a [2 + 2 + 2] cycloaddition reaction, then subsequently restarted after a cycloreversion. The rearrangement rate is also attenuated upon coordination of the fluxional cage to Pd(II) or Ru(II) as part of a phosphoramidite–olefin ligand. By its inclusion in the simple ligand design, the fluxional cage transmits stereochemical information to the metal ion—either through the covalent ligand backbone or by ion pairing with a chiral counterion. This property is exploited in enantioselective catalysis of an allylic substitution reaction, as well as in creating chiral-at-metal stereogenic centres that adopt the configurational dynamics of the cage.

## Results

**Fig. 1:**
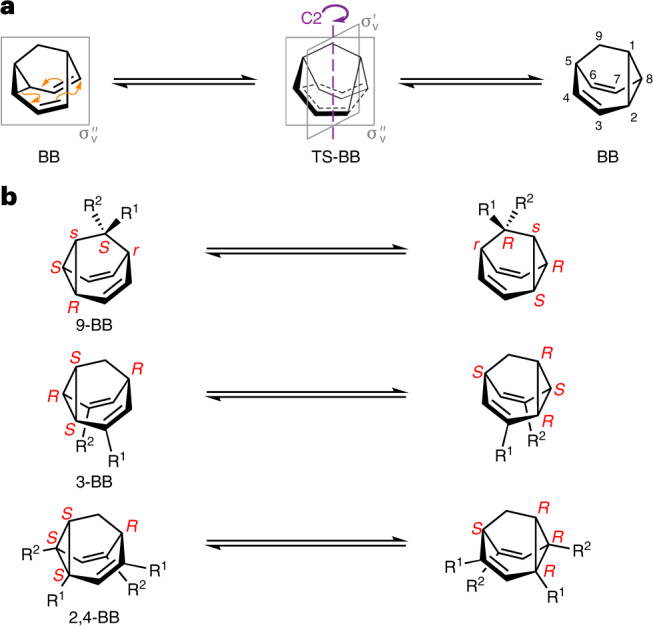
Multiple dynamic *sp*^3^-carbon centres. **a**,**b**, Fluxional *sp*^3^-carbon stereochemistry arises in BBs when the structures interchanged by their Cope rearrangements (**a**) are desymmetrized with any of the three substitution patterns shown in **b**. Cahn–Ingold–Prelog priorities are chosen to be R^1^ > C > R^2^ for the assignment of absolute configuration. When assigning a descriptor to position 9 of 9-BB, the cyclopropyl bridgehead C1 has precedence over the divinyl bridgehead C5 (Supplementary Fig. 1). 3-BB and 2,4-BB each have four chirotopic (*R*/*S*) centres, whereas the 9-BB pattern gives rise to five stereogenic centres, of which three are chirotopic and two are achirotopic (*r*/*s*).

The Cope rearrangement of barbaralane (BB) is an example (Fig. 1a) of a narcissistic^25,33^ automerization—it gives rise to a degenerate structure through a transition state (TS-BB) bearing an internal mirror plane (${\upsigma}_{{{\mathrm{v}}}}^{\prime}$) that is not present in the minimum energy structure. We noted that by desymmetrizing BB (Fig. 1b) using either a 9-BB, 3-BB or 2,4-BB substitution pattern, the mirror plane present at the energy minimum (${\upsigma}_{{{\mathrm{v}}}}^{\prime\prime}$) is lost, while the mirror plane formed in the transition state (${\upsigma}_{{{\mathrm{v}}}}^{\prime}$) is retained. As a result, the Cope rearrangement inverts simultaneously some, or all, of the four or five stereogenic centres present in the structure. Given that the rearrangement of BB is known to proceed with a remarkably low free energy of activation, Δ*G*^‡^, of 32.3 kJ mol^−1^ (Supplementary Table 7)^34–38^, chiral 9-BB, 3-BB or 2,4-BB derivatives should undergo rapid enantiomerization.

## Diastereomeric adaptation

**Fig. 2:**
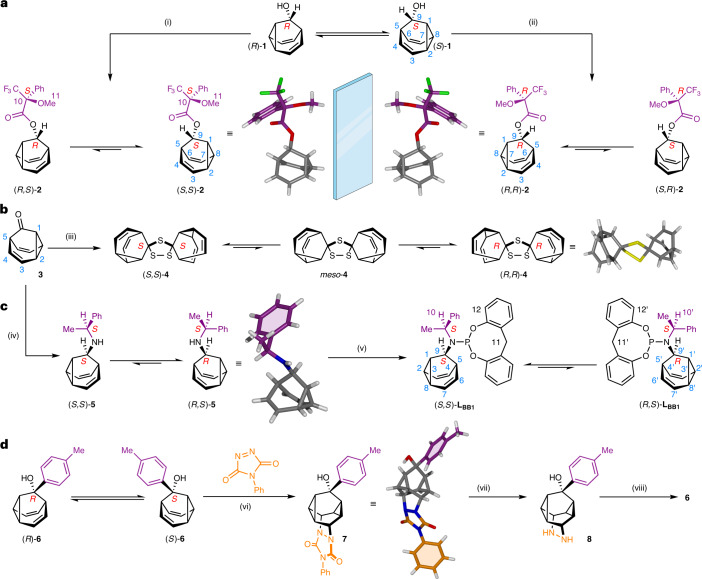
Diastereomeric adaptation and manipulation of chiral BBs. **a**,**b**, The dynamic *sp*^3^-C stereochemical equilibrium of the BB cage is degenerate in **1** but becomes biased towards one stereoisomer upon attaching a chiral auxiliary (**a**) or by dimerization through a spirocyclic bridge (**b**). **c**, The position of the stereochemical equilibrium changes (and inverts) upon modifying the structure of a chiral auxiliary, remote from the BB unit. **d**, Further control of the *sp*^3^-C stereochemistry is exerted by a cycloaddition reaction, which freezes and symmetrizes the structure, before subsequent cycloreversion re-establishes the dynamic stereochemical equilibrium. Reagents and conditions: (i) 1. (*S*)-MTPA, (COCl)_2_, hexanes, DMF, room temperature (r.t.) to −20 °C, 16 h. 2. **1**, DMAP, Et_3_N, CHCl_3_, r.t., 5 d, 58%. (ii) 1. (*R*)-MTPA, (COCl)_2_, hexanes, DMF, r.t. to −20 °C, 16 h. 2. **1**, DMAP, Et_3_N, CHCl_3_, r.t., 3 d, 79%. (iii) **3**, Lawesson’s reagent, PhMe, 110 °C, 18 h, 13%. (iv) 1. **3**, (*S*)-1-phenylethylamine, AcOH, MeOH, r.t., 30 min. 2. NaBH_3_CN, 100 °C, 16 h, 89%. (v) **5**, PCl_3_, Et_3_N, CH_2_Cl_2_, 0 °C, 3 h. 2. 2,2′-methylenediphenol, CH_2_Cl_2_, 0 °C to r.t., 16 h, 44%. (vi) **6**, PTAD, CH_2_Cl_2_, 50 °C, 24 h, 85%. (vii) **7**, NaOH, ^*i*^PrOH, 85 °C, 24 h, taken on crude. (viii) CuCl_2_, HCl_(aq)_, 0 °C, 4 h, 48% from **7**. X-ray structures are shown in stick representation. Compound **4** crystallizes in a centrosymmetric space group, that is, (*S*,*S*)-**4** and (*R*,*R*)-**4** are both present, but only (*R*,*R*)-**4** is shown for clarity. Diffraction data for crystals of (*R*,*S*)-**5** allow only assignment of relative stereochemistry. MTPA, α-methoxy-α-trifluoromethylphenylacetic acid; DMF, *N*,*N*-dimethylformamide; DMAP, 4-(dimethylamino)pyridine; PTAD, phenyl-1,2,4-triazoline-3,5-dione.

**Fig. 3:**
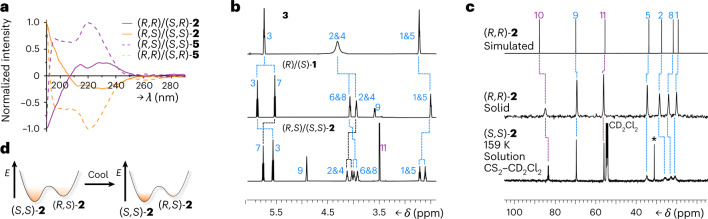
Spectroscopic evidence of *sp*^3^-carbon adaptation to covalently tethered chiral auxiliaries. **a**, Normalized circular dichroism spectra of **2** (115 μM in MeCN) and **5** (210 μM in MeCN) confirm that antipodal equilibrium mixtures give equal and opposite absorbances. **b**, A comparison of partial solution ^1^H NMR spectra (CDCl_3_, 298 K) shows the reduced symmetry of the chiral 9-BB motif: top, **3** (700 MHz); middle, (*R*)/(*S*)-**1** (600 MHz); bottom, (*R*,*S*)/(*S*,*S*)-**2** (600 MHz). Resonances are labelled according to the numbering in Fig. 2. **c**, Comparison of partial ^13^C{^1^H} NMR spectra: top, solid-state chemical shifts calculated from the X-ray crystal structure of (*R*,*R*)-**2** in CASTEP v17.2^65^ using the Perdew–Burke–Ernzerhof functional^66^ and on-the-fly generated pseudopotentials; middle, (*R*,*R*)-**2** as a powder at ambient temperature (105 MHz); bottom, (*S*,*S*)-**2** as a solution in 5:1 CS_2_–CD_2_Cl_2_ at low temperature (125 MHz, 159 K). The asterisk indicates the resonance of residual acetone. **d**, The Boltzmann distribution of isomers shifts towards a single stereoisomer at low temperatures; for example, a free energy difference of ~5 kJ mol^−1^ would give an ~90:10 equilibrium mixture at room temperature, but >98:2 at 159 K, so NMR data would be expected to show a single, major species, as is apparent when comparing the three spectra in **c**.

We targeted 9-BB **1** (Fig. 2) as a convenient example of the 9-BB substitution pattern that bears a hydroxyl group for synthetic elaboration. The Cope rearrangement involving positions 2–8 of **1** (Fig. 2a) causes enantiomerization of the whole cage and formally inverts the stereochemistry of position 9 by effectively ‘swapping’ the cyclopropyl and alkene substituents connected to the stereocentre. Compound **1** was synthesized (Supplementary Scheme 1) by a three-step route from ethynyl magnesium bromide and tropylium tetrafluoroborate, using a gold-catalysed enyne cycloisomerization^39,40^ to form the BB backbone. When labelling **1** and subsequent compounds, a single stereochemical descriptor is included to indicate the configuration at position 9 of the BB (Fig. 2a), for example, (*R*)-**1** and (*S*)-**1**, omitting the additional stereochemical labels of positions 1, 2, 5 and 8 for simplicity (Fig. 1). Treatment of **1** with Mosher’s acid chloride (Fig. 2a) produces a set of Mosher’s esters **2** in which the configurationally fixed stereocentre is introduced at a distance of three covalent bonds from the dynamic BB unit. An additional descriptor for the configuration of the Mosher’s ester group is included in the labels for **2**. Derivatization with (*S*)-Mosher’s acid gives a dynamic mixture of two diastereomers, (*R*,*S*)-**2** and (*S*,*S*)-**2**, whereas (*R*)-Mosher’s acid gives (Fig. 2a) the antipodal mixture, (*S*,*R*)-**2** and (*R*,*R*)-**2**. Solutions of the two antipodal dynamic mixtures give opposite circular dichroism spectra (Fig. 3a), as would be expected.

DFT modelling using the ωB97X-D functional^41^, 6-311++G(d,p) basis set^42,43^ and a CS_2_ polarizable continuum solvent model using the integral equation formalism variant^44^ was employed to compare (Supplementary Table 7) the stereoisomerization energetics of BB, **1** and **2**. Using these parameters, the automerization of BB is predicted to proceed with a calculated activation free energy, ${{{\mathrm{{\Delta}}}}}{G}_{\rm{calc}}^{\ddagger}$, of 38.5 kJ mol^−1^, which is ~6 kJ mol^−1^ higher than the experimentally measured^35^ activation free energy, ${{{\mathrm{{\Delta}}}}}{G}_{\rm{exp}}^{\ddagger}$, of 32.3 kJ mol^−1^, in keeping with previous DFT investigations^36,37^. DFT methods systematically overestimate the energy barrier to Cope rearrangement of BBs, but nevertheless allow useful comparisons of trends in activation energies and are known to predict accurately the relative energy minima of isomers^36,37^. The computationally predicted ${{{\mathrm{{\Delta}}}}}{G}_{\rm{calc}}^{\ddagger}$ values for **1** (38.0 kJ mol^−1^) and **2** (35.5 kJ mol^−1^) are very similar to BB, indicating that the hydroxyl or ester group substitutions at position 9 do not appreciably change the rapid kinetics.

The absence of the ${\upsigma}_{{{\mathrm{v}}}}^{\prime\prime}$ mirror plane in **1** is evident (Fig. 3b) in its solution-state ^1^H NMR spectrum—H3 and H7 are magnetically inequivalent, for example. However, the rapid enantiomerization induces a ${\upsigma}_{{{\mathrm{v}}}}^{\prime}$ mirror plane to the time-averaged structure of **1**, so only six distinct methine resonances are observed overall. The additional, fixed stereocentre of **2** breaks this ${\upsigma}_{{{\mathrm{v}}}}^{\prime}$ symmetry. Consequently, nine distinct signals corresponding to the BB methine groups are observed (Fig. 3b).

An energy difference, Δ*G*_calc_, of 4.5 kJ mol^−1^ is computed (Supplementary Table 7) for the rearrangement of **2**. The influence of the (*S*)-Mosher’s ester group moulds the configuration of the cage unit, which preferentially adopts its *S* form, biasing the equilibrium towards (*S*,*S*)-**2**. Consistent with this prediction, a single crystal (Fig. 2a) obtained from the dynamic (*S*)-Mosher’s ester mixture was found to contain (*S*,*S*)-**2** as a frozen^34^, single stereoisomer. An equal and opposite outcome is observed from the (*R*)-Mosher’s ester mixture, giving the enantiomeric (*R*,*R*)-**2** solid-state structure.

To establish the nature of the dynamic solution-state mixtures, we compared (Fig. 3c) the solid-state ^13^C{^1^H} NMR spectrum of enantiopure (*R*,*R*)-**2** crystals to a spectrum obtained using a sample of the (*S*,*S*)-**2** crystals dissolved in 5:1 CS_2_–CD_2_Cl_2_, generating a dynamic mixture of (*R*,*S*)-**2** and (*S*,*S*)-**2**. Cooling the solution to 159 K causes the BB ^13^C{^1^H} NMR resonances to enter the slow exchange regime. As 159 K is only ~20 K below the observed coalescence temperature (Supplementary Fig. 66) for this low-barrier process, some resonances exhibit exchange broadening. At this low temperature, the decrease of available thermal energy causes the Boltzmann distribution to shift (Fig. 3d) further towards the lowest-energy isomer^34^. The solid-state chemical shifts of the BB *sp*^3^-carbons 1, 2, 5, 8 and 9 are assigned (Fig. 3b) by comparison to the calculated chemical shifts of (*R*,*R*)-**2**. The resonances of the solution sample match up well with those of (*R*,*R*)-**2** in the solid state, allowing us to assign the resolved solution-state diastereomer as (*S*,*S*)-**2**. The solution-state analysis is thus consistent with the diastereomeric preference predicted by DFT and observed in the solid state. The 9-BB cage undergoes dynamic diastereomeric adaptation under the influence of the configurationally fixed Mosher’s ester group.

The dynamic stereochemical equilibrium can also be biased in the absence of a fixed stereogenic element. Dimerization of two 9-BB-type cages through a spirocylic linkage breaks the degeneracy of the equilibrium. By treating (Fig. 2b) barbaralone **3** with Lawesson’s reagent, we isolated trithiolane **4**, which undergoes dynamic rearrangements between an achiral isomer, *meso*-**4**, and a pair of enantiomers, (*S*,*S*)-**4** and (*R*,*R*)-**4**. A small Δ*G*_calc_ of 0.7 kJ mol^−1^ is predicted (Supplementary Table 7) to favour the pair of enantiomers over the *meso* form in the solution state. Single crystals grown from a solution of **4** contain a racemic mixture of the two chiral stereoisomers.

Further chemical modification to the substituent at position 9 can substantially influence, and even invert, the cage’s equilibrium distribution. The chiral phosphoramidite–olefin^45–47^ ligand **L**_**BB1**_ was synthesized (Fig. 2c) by first subjecting **3** to reductive amination with (*S*)-1-phenylethylamine to afford a mixture of (*S*,*S*)-**5** and (*R*,*S*)-**5**. Sequential treatment of the amine with PCl_3_ then 2,2′-methylenediphenol affords **L**_**BB1**_. The 2,2′-methylenediphenol functionality was selected as it lacks fixed stereochemistry but has been shown to adopt dynamically chiral conformations as part of phosphoramidite ligands^45^. Comparing **L**_**BB1**_ to **5** reveals that the differing size and shape of the substituent at position 9 drives the dynamic stereochemical equilibria of the fluxional cage towards opposite configurations. The solution-phase equilibrium of the secondary amine is weighted (Supplementary Table 7) towards the (*R*,*S*)-**5** diastereomer by a Δ*G*_calc_ of 3.8 kJ mol^−1^, matching the structure observed by X-ray analysis (Fig. 2c) of a single crystal. By contrast, the (*S*,*S*)-**L**_**BB1**_ diastereomer of the phosphoramidite is favoured with a Δ*G*_calc_ of 20.2 kJ mol^−1^. The large magnitude of Δ*G*_calc_ for **L**_**BB1**_ highlights that the configurational dynamics of the 9-BB motif (Fig. 1) correlate with notable changes in its 3D shape^34^ and, therefore, its energy. At the same time, the opposing stereochemistry of cages **5** and **L**_**BB1**_ demonstrates that the malleable *sp*^3^-carbon configuration adapts to changes in the nearby steric environment.

## Manipulating rates and transfer of stereochemistry

To exert further control over the fluxional enantiomerization, we sought to exploit the reactivity of the BBs’ skipped diene units. The fluxional rearrangements can be stopped entirely by engaging the alkene units in covalent bonding, whereas coordination of the *π* electrons to a transition-metal ion^38^ modulates the rearrangement rate instead.

**Fig. 4:**
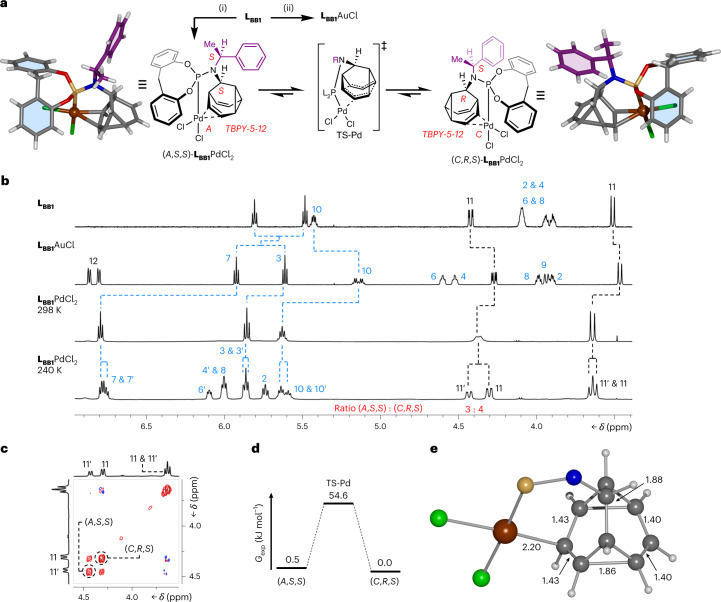
Transfer of dynamic *sp*^3^-carbon stereochemistry in Au(I) and Pd(II) complexes. **a**, Bidentate coordination of **L**_**BB1**_ to PdCl_2_ leads to cc-Cope rearrangement in which the Pd ‘walks’ along the side of the BB cage, modulating the rearrangement rate. For comparison, monodentate ligand coordination is observed with AuCl. Reagents and conditions: (i) **L**_**BB1**_, PdCl_2_(NCMe)_2_, CDCl_3_, r.t., 15 min, 98%. (ii) **L**_**BB1**_, Me_2_S·AuCl, CDCl_3_, r.t., 10 min, 93%. X-ray crystal structures are shown in stick representation, with a ball for metal ions. Solvent molecules are omitted for clarity. Two structurally similar conformers of each **L**_**BB1**_PdCl_2_ stereoisomer are present in the unit cell, but only one of each is shown for clarity. **b**, Partial ^1^H NMR (CDCl_3_) spectra: top, **L**_**BB1**_ (599 MHz, 298 K); second row, **L**_**BB1**_AuCl (599 MHz, 298 K); third row, **L**_**BB1**_PdCl_2_ (499 MHz, 298 K); bottom, **L**_**BB1**_PdCl_2_ (499 MHz, 240 K). Resonances are labelled according to the numbering for **L**_**BB1**_ in Fig. 2. The spectrum at 240 K shows the two **L**_**BB1**_PdCl_2_ complexes in slow exchange in a ratio of 3:4. **c**, A partial ^1^H-^1^H EXSY NMR spectrum (499 MHz, CDCl_3_, 240 K, mixing time *τ*_m_ = 200 ms) showing exchange peaks (red) between resonances of the minor (H_11′_) and major (H_11_) diastereomers as well as COSY peaks (blue) of geminal proton pairs. **d**, Free energy diagram for the cc-Cope rearrangement. **e**, Ball-and-stick representation of the DFT-calculated (ωB97X-D/6-311++G(d,p)/SDD/CS_2_)^67^ geometry of **L**_**BB1**_PdCl_2_, showing the BB cage at the transition state, TS-Pd. A truncated structure omitting phosphorus and nitrogen substituents is shown for clarity with selected bond lengths given in ångstroms.

**Fig. 5:**
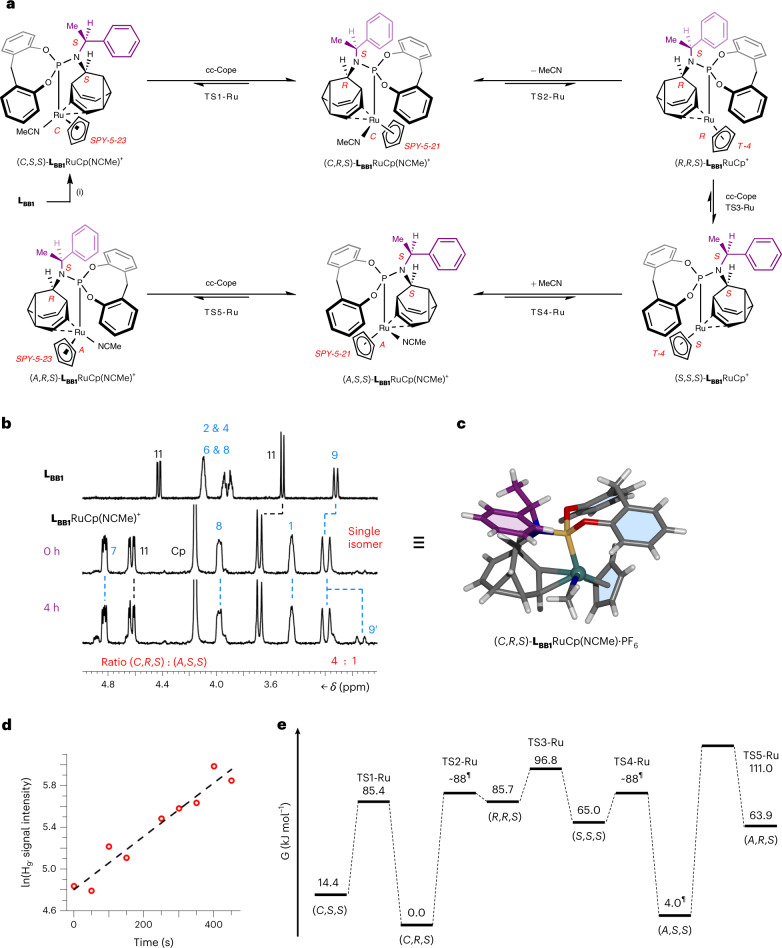
Transfer of dynamic *sp*^3^-carbon stereochemistry in chiral-at-Ru(II) complexes. **a**, Four diastereomeric square pyramidal complexes are linked by cc-Cope rearrangements and exchange of an MeCN ligand, which proceeds through two intermediate tetrahedral complexes. A non-coordinated PF_6_^−^ counterion is omitted from the structural formula of each complex for clarity. Reagents and conditions: (i) **L**_**BB1**_, CpRu(NCMe)_3_·PF_6_, CDCl_3_, r.t., 5 min, 69%. **b**, Comparison of the partial ^1^H NMR (CDCl_3_, 298 K) spectra: top, **L**_**BB1**_ (599 MHz); middle, a sample of **L**_**BB1**_RuCp(NCMe)·PF_6_ analysed immediately after dissolving a crystalline sample (400 MHz); bottom, the same sample after allowing it to equilibrate for 4 h (400 MHz), revealing that an initially observed single isomer reaches a 4:1 equilibrium mixture. Resonances are labelled according to the numbering for **L**_**BB1**_ in Fig. 2. **c**, (*C*,*R*,*S*)-**L**_**BB1**_RuCp(NCMe)·PF_6_ is identified in the solid-state X-ray crystal structure, which is shown in stick representation with a ball for the Ru(II) ion. Solvent molecules and the PF_6_^−^ counterion are omitted for clarity. **d**, Integration of the ^1^H NMR (400 MHz, CDCl_3_, 298 K) resonance corresponding to H9′ of (*A*,*S*,*S*)-**L**_**BB1**_RuCp(NCMe)·PF_6_ upon dissolving a crystalline sample of (*C*,*R*,*S*)-**L**_**BB1**_RuCp(NCMe)·PF_6_ reveals a first-order increase in concentration with *k*_obs_ = 2.56 × 10^−3^ s^−1^. **e**, A potential energy surface for isomerization for the cc-Cope processes. Values of ${{{\mathrm{{\Delta}}}}}{G}_{\rm{calc}}$ and ${{{\mathrm{{\Delta}}}}}{G}_{\rm{calc}}^{\ddagger}$ (ωB97X-D/6-311++G(d,p)/SDD/CS_2_)^67^ are given except where ¶ indicates experimentally measured (**b**,**d**) equilibrium (Δ*G*_exp_) and ligand exchange (${{{\mathrm{{\Delta}}}}}{G}_{\rm{exp}}^{\ddagger}$) energies (in units of kJ mol^−1^).

An enantiomerizing mixture of 9-(*p*-tolyl)barbaralol **6** engages (Fig. 2d) in a [2 + 2 + 2] cycloaddition reaction with phenyl-1,2,4-triazoline-3,5-dione^48^, giving rise to **7**. This reaction halts the rearrangement while also symmetrizing the structure by forming a second cyclopropyl group. Subsequently, the fluxional cage can be regenerated (Fig. 2d) in a two-step transformation through diazinane **8** (Supplementary Fig. 62), which undergoes cycloreversion with loss of N_2_ upon oxidation with CuCl_2_. Alternatively, coordination of Pd(II) (Fig. 4) or Ru(II) (Fig. 5) to **L**_**BB1**_ causes a reduction in the rate of the Cope rearrangement, as discussed below.

**L**_**BB1**_ and PdCl_2_ form (Fig. 4a) a chiral-at-metal^49,50^ complex, **L**_**BB1**_PdCl_2_, linking the *sp*^3^-carbon configurational inversion to the *A*/*C* isomerism^51^ of the distorted trigonal bipyramidal (*TBPY*-5-12) coordination environment (Supplementary Fig. 2). Both possible stereoisomers, arising from coordination of (*S*,*S*)-**L**_**BB1**_ or (*R*,*S*)-**L**_**BB1**_ through their phosphorus centre and an alkene, are observed (Fig. 4a) in the X-ray crystal structure of the **L**_**BB1**_PdCl_2_ complex. The alkene coordination is also evident (Fig. 4b) in the solution state by ^1^H NMR spectroscopy. For comparison, a monodentate **L**_**BB1**_AuCl complex (Fig. 4a) was prepared, which shows only small changes in the ^1^H NMR chemical shifts of its alkene signals H3 and H7 relative to the free ligand (Fig. 4b). The room-temperature spectrum of **L**_**BB1**_PdCl_2_, on the other hand, reveals a large change in the chemical shift of H7, consistent with coordination of Pd(II) to the alkene on the same face as the phosphoramidite group.

At 240 K, the ^1^H NMR spectrum reveals (Fig. 4b) the two **L**_**BB1**_PdCl_2_ isomers in slow exchange. Two sets of signals are observed in a 3:4 ratio, corresponding to a small free energy difference, Δ*G*_exp_, of 0.5 kJ mol^−1^ between the two isomers. Consistent with this observation, DFT calculations predict (Supplementary Table 7) a small Δ*G*_calc_ of 1.8 kJ mol^−1^ in favour of (*C*,*R*,*S*)-**L**_**BB1**_PdCl_2_.

Further NMR and DFT analyses elucidate the mechanism by which the **L**_**BB1**_PdCl_2_ complex isomerizes. Depending on the placement and nature of substituents around the Cope substrate, metal coordination can either stabilize a charged, intermediate species as part of a stepwise associative rearrangement mechanism, or it can increase the rate of a concerted rearrangement pathway by stabilizing the transition state^52^. Consequently, Pd(II) salts and other cationic metal ions are known to accelerate Cope rearrangements^53,54^. Remarkably, coordination of the Pd(II) to one face of the fluxional cage in **L**_**BB1**_PdCl_2_ has the opposite effect, slowing down the Cope rearrangement. Unlike **2**, for example, whose ^1^H NMR resonances (499 MHz) enter the slow exchange regime below 160 K (Supplementary Fig. 64), the slower rearrangement of **L**_**BB1**_PdCl_2_ is resolved by ^1^H NMR spectroscopy at the higher temperature of 240 K. Using 2D ^1^H–^1^H exchange spectroscopy (EXSY) at 240 K (Fig. 4c), we measured a rate of exchange, *k*, of 6.48 s^−1^, indicating a ${{{\mathrm{{\Delta}}}}}{G}_{\rm{exp}}^{\ddagger}$ of 54.6 kJ mol^−1^ for **L**_**BB1**_PdCl_2_ (Fig. 4d). The DFT-calculated transition-state structure (Fig. 4e) shows pairs of equidistant C–C bonds, as would be expected for a coordination-coupled Cope (cc-Cope) rearrangement (Fig. 4a) in which the Pd(II) remains bound to the cage through a concerted rearrangement step. The DFT-predicted ${{{\mathrm{{\Delta}}}}}{G}_{\rm{calc}}^{\ddagger}$ of 60.6 kJ mol^−1^ for this cc-Cope mechanism matches well with the ${{{\mathrm{{\Delta}}}}}{G}_{\rm{exp}}^{\ddagger}$ of 54.6 kJ mol^−1^.

These data indicate that the metal ion ‘walks’ along one side of the BB cage as the Cope rearrangement proceeds, moving back and forth in sync with the pericyclic reaction^52^. Consequently, the BB not only transmits the stereochemical information from the fixed *sp*^3^-carbon stereocentre through its dynamic *sp*^3^-carbon framework, biasing the chiral-at-metal configuration, but it also imparts a novel mechanism of intramolecular configurational change at a pentavalent stereocentre, which differs from the established pseudorotation and turnstile mechanisms^55^.

The dynamic *sp*^3^-carbon stereochemistry of **L**_**BB1**_ can also be linked to an intermolecular ligand exchange process. The cyclopentadienyl (Cp) half-sandwich Ru(II) complex^56^ **L**_**BB1**_RuCp(NCMe)·PF_6_ (Fig. 5) has a stereogenic, distorted square pyramidal Ru(II) centre (Supplementary Fig. 91) coordinated to a labile MeCN ligand. While cc-Cope rearrangements interconvert the *SPY*-5-21 and *SPY*-5-23 configurational isomers^51^ (Fig. 5a), MeCN dissociation forms the distorted tetrahedral (*T*-4) chiral-at-metal species **L**_**BB1**_RuCp·PF_6_, which mediates *A*/*C* stereochemical inversion.

Ru(II) coordination slows the Cope rearrangement sufficiently for a single stereoisomer to be resolved as a metastable species under ambient conditions (Fig. 5b). Upon dissolving single crystals of **L**_**BB1**_RuCp(NCMe)·PF_6_, obtained by slow evaporation, the ^1^H NMR spectrum shows the presence of a single complex (Fig. 5b) with resonances distinct from non-coordinated **L**_**BB1**_. After allowing the sample to fully equilibrate at room temperature for four hours, a new set of peaks is observed (Fig. 5b) at a ratio of 4:1 in favour of the initially observed isomer, equivalent to a Δ*G*_exp_ of 4.0 kJ mol^−1^. X-ray analysis (Fig. 5c) of the crystalline sample reveals the identity of the energetically favoured isomer to be (*C*,*R*,*S*)-**L**_**BB1**_RuCp(NCMe)·PF_6_.

We measured the isomerization rate of (*C*,*R*,*S*)-**L**_**BB1**_RuCp(NCMe)·PF_6_ by monitoring (Fig. 5d) the first-order growth in intensity of the resonance at 3.1 ppm corresponding to the H9′ signal of (*A*,*S*,*S*)-**L**_**BB1**_RuCp(NCMe)·PF_6_—the isomer calculated (Fig. 5e) to be the next most stable stereoisomer. The *k*_obs_ of 2.56 × 10^−3^ s^−1^ at 298 K allows us to determine a ${{{\mathrm{{\Delta}}}}}{G}_{\rm{exp}}^{\ddagger}$ of 87.8 kJ mol^−1^. Comparison of this value to (1) maxima of the computed potential energy surface (Fig. 5e), (2) a CD_3_CN exchange experiment (Supplementary Fig. 70) and (3) literature measurements of MeCN dissociation from Cp half-sandwich Ru(II) complexes^55^ suggests that the cc-Cope and MeCN exchange processes occur at similar rates. To achieve the (*C*,*R*,*S*)-to-(*A*,*S*,*S*) isomerization observed by NMR, the complex must undergo both ligand exchange and cc-Cope steps (Fig. 5e). Overall, the energetic bias towards (*C*,*R*,*S*)-**L**_**BB1**_RuCp(NCMe)·PF_6_ and observation of its stepwise stereomutation to (*A*,*S*,*S*)-**L**_**BB1**_RuCp(NCMe)·PF_6_ illustrate that the fluxional *sp*^3^-carbon cage mediates the transfer of stereochemical information with high fidelity from the single, fixed benzylamino stereocentre through its rigid, tricyclic structure.

## Dynamic stereocontrol by ion pairing

**Fig. 6:**
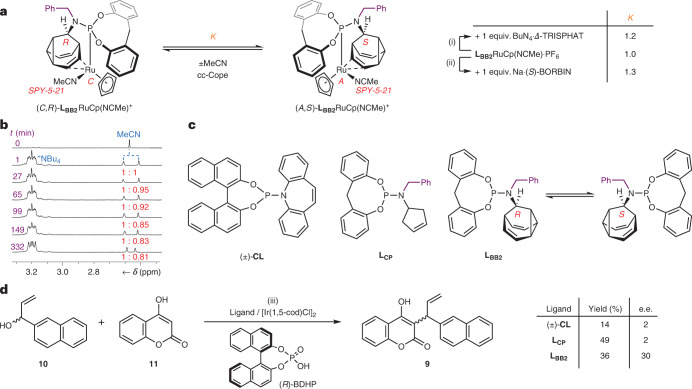
Chiral counterion-directed *sp*^3^-carbon and chiral-at-metal stereochemistry applied to enantioselective catalysis. **a**, The enantiomerization of **L**_**BB2**_RuCp(NCMe)^+^ is degenerate in the presence of an achiral counterion. In the presence of chiral anions, the ion-pair interactions break the degeneracy and bias the equilibrium towards one stereoisomer of the BB–metal complex, that is, *K* ≠ 1. Note: only the two lowest-energy stereoisomers are shown for clarity. Reagents and conditions: (i) **L**_**BB2**_RuCp(NCMe)·PF_6_, BuN_4_·*Δ*-TRISPHAT, CDCl_3_, r.t. (ii) **L**_**BB2**_RuCp(NCMe)·PF_6_, Na·(*S*)-BORBIN, CDCl_3_, r.t. **b**, Comparison of the partial ^1^H NMR spectra (400 MHz, CDCl_3_, 298 K) of **L**_**BB2**_RuCp(NCMe)·PF_6_ before (top row) and after (other rows) the addition of 1 equiv. of BuN_4_·Δ-TRISPHAT. Integration of the MeCN resonances shows that the initially racemic **L**_**BB2**_RuCp(NCMe)^+^ ion gradually becomes enriched in one stereoisomer (*K* = 1.2). **c**,**d**, The stereoinduction (**c**) arising from three phosphoramidite ligands was compared in an iridium-catalysed allylic substitution (**d**). We hypothesize that the chiral anion formed in situ biases the stereochemical equilibrium of the cationic **L**_**BB2**_Ir *π*-allyl intermediate, leading to its improved performance relative to the control ligands lacking dynamic *sp*^3^-C stereochemistry. Reagents and conditions: (iii) 1. [Ir(1,5-cod)Cl_2_] (4 mol%), ligand (16 mol%), THF, 30 min, r.t. 2. (*R*)-BDHP (10 mol%), r.t., 24 h. Δ-TRISPHAT*,* Δ-tris(tetrachloro-1,2-benzenediolato)phosphate(V); (*S*)-BORBIN, bis[(*S*)-1,1′-bis-2-naphtholato]borate; 1,5-cod, 1,5-cyclooctadiene; (*R*)-BDHP, (*R*)-1,1′-binaphthyl-2,2′-diyl hydrogen phosphate.

Having observed transmission of stereochemical information within the covalent frameworks of the **L**_**BB1**_ complexes, we investigated the influence of chiral counterions^57^ on the degenerate enantiomerization (Fig. 6a) of the cationic **L**_**BB2**_RuCp(NCMe)^+^ complex, which lacks a fixed stereocentre in its ligand structure. The complex was synthesized (Supplementary Scheme 3) as its hexafluorophosphate salt, **L**_**BB2**_RuCp(NCMe)·PF_6_, in a manner analogous to its permanently chiral homologue **L**_**BB1**_RuCp(NCMe)·PF_6_ (Fig. 5). X-ray analysis of single crystals confirmed (Supplementary Fig. 94) the expected structure of **L**_**BB2**_RuCp(NCMe)·PF_6_ and revealed the presence (Supplementary Fig. 95) of both the (*C*,*R*)- and (*A*,*S*)-isomers in the crystal unit cell.

In the absence of chiral anions, a single ^1^H NMR signal is observed for the coordinated MeCN ligand of **L**_**BB2**_RuCp(NCMe)·PF_6_ in CDCl_3_ solution (Fig. 6b). However, the addition of one molar equivalent of a chiral shift reagent, Bu_4_N·*Δ*-TRISPHAT^58^ (Fig. 6b) or Na·(*S*)-BORBIN^59^ (Supplementary Fig. 71), splits the signal in two. Rapid and reversible counterion exchange in the presence of Bu_4_N·*Δ*-TRISPHAT establishes an equilibrium mixture that includes diastereomeric ion pairs, for example, (*C*,*R*)-**L**_**BB2**_RuCp(NCMe)·*Δ*-TRISPHAT and (*A*,*S*)-**L**_**BB2**_RuCp(NCMe)·*Δ*-TRISPHAT, which give distinct NMR resonances. Therefore, by tracking the relative intensities of these resonances over time (Fig. 6b and Supplementary Fig. 72) we can monitor changes in sample composition as the chiral anion biases the **L**_**BB2**_RuCp(NCMe)^+^ complex towards one stereoisomer. Under these conditions, the *Δ*-TRISPHAT sample evolves from a 1:1 mixture of stereoisomers to a 1:0.81 mixture over several hours, corresponding to an equilibrium constant, *K*, of 1.2, and Δ*G*_exp_ ≈ 0.5 kJ mol^−1^. The timeframe of the sample’s evolution is consistent with the slow kinetics of isomerization measured (Fig. 5) for **L**_**BB1**_RuCp(NCMe)·PF_6_, suggesting that the stereomutation is again proceeding by cc-Cope and MeCN ligand exchange. The (*S*)-BORBIN sample reaches (Supplementary Fig. 72) a *K* of 1.3 over a similar time period. Overall, these experiments establish a means of noncovalent control of the BB stereochemistry. In principle, *K* could be further increased by removal or omission of any competing achiral anions (such as PF_6_^−^) from the reaction mixture and optimization of solvent and concentration.

Based on these results, we hypothesized that this counterion-directed stereochemistry of cationic **L**_**BB2**_ complexes could be exploited in enantioselective ion-pair catalysis^60^. Unusually, the fixed stereochemistry of the chiral anion would be passed to the catalytically active, fluxional metal complex to transiently generate an enantioenriched ligand framework in situ. To probe this concept, we screened **L**_**BB2**_ and two control ligands (Fig. 6c) in the enantioselective synthesis of **9** (Fig. 6d) through iridium-catalysed allylic substitution of alcohol **10** by hydroxycoumarin **11**^61^. We used chiral phosphoric acid (*R*)-BDHP, which we expected to protonate **10** and induce formation of the iridium-stabilized allylic cation intermediate while simultaneously generating an equivalent of a chiral phosphate anion. The optimized literature conditions^61^ for this allylic substitution employ an achiral Lewis acid (Yb(OTf)_3_) rather than a Brønsted acid to generate the allylic cation, in conjunction with enantiopure Carreira’s^46^ phosphoramidite–olefin ligand, **CL**, to impart enantioselectivity. Pleasingly, replacing these reagents with (*R*)-BDHP and racemic (±)-**CL** leads to the formation of **9**, albeit in just 14% isolated yield. Importantly, however, there is essentially no enantioinduction under the influence of (±)-**CL**. The product is obtained with a negligible enantiomeric excess (e.e.) of just 2%. It appears that the chiral phosphoric acid alone does little to override the stereochemical preference (or overall lack of it) arising from the racemic ligand. Using achiral phosphoramidite–olefin ligand **L**_**CP**_ leads to a similar outcome. **L**_**CP**_ bears many of the same structural features of **L**_**BB2**_, but with an achiral cyclopentene unit in place of the dynamically chiral 9-BB substructure. Compound **9** is produced in 49% yield and just 2% e.e. using **L**_**CP**_. Conversely, our fluxionally chiral ligand, **L**_**BB2**_, delivers an improved e.e. Using **L**_**BB2**_, we isolated **9** in 36% yield and 30% e.e. Contrasting this result with the outcome of the reactions using **L**_**CP**_ and (±)-**CL** supports the idea that the chiral phosphate counterion biases the covalent **L**_**BB2**_ ligand stereochemistry of the cationic intermediate complex (Supplementary Scheme 5), which in turn improves the enantioinduction in the key bond-forming step. Although the resulting e.e. is moderate for this particular set of reaction conditions, it suggests that the use of fluxional *sp*^3^-carbon units may enhance the design of ligand frameworks for ion-pair catalysis^60^ and other forms of enantioselective synthesis.

## Conclusions

Cope rearrangements of chiral 9-BB cages simultaneously invert every stereogenic *sp*^3^-carbon centre of their structures. These configurational rearrangements occur rapidly and reversibly, achieving the uncommon property of dynamic *sp*^3^-carbon stereochemistry—one that has remained surprisingly rare since Le Bel^1^ and van’t Hoff^2^ first identified tetrahedral carbon as a source of molecular chirality in 1874. Both the rate of *sp*^3^-carbon inversion and the equilibrium distribution of isomers are sensitive to changes in the 9-BB structure. On the one hand, the dynamics of the rearrangement processes are controlled through manipulation of covalent bonding or metal coordination of the 9-BB olefin groups, providing convenient functional handles. On the other hand, the cage adapts its configuration to minimize steric interactions with nearby fixed stereogenic elements and, in so doing, is able to transmit the stereochemical information across its rigid, tricyclic backbone. When interfaced with transition-metal complexes, the dynamic cage conveys a stereochemical preference to the chiral-at-metal^49,50^ centre. Controllable and adaptable *sp*^3^-carbon stereochemistry of this kind can be exploited in enantioselective synthesis^7,9,10,30,45,62,63^ and chiral functional materials^64^.

## Online content

Any methods, additional references, Nature Portfolio reporting summaries, source data, extended data, supplementary information, acknowledgements, peer review information; details of author contributions and competing interests; and statements of data and code availability are available at 10.1038/s41557-023-01156-7.

## Supplementary information

Supplementary Information Supplementary Figs. 1–96, Tables 1–41, schemes 1–5, discussion and experimental procedures.

Supplementary Data 1 Crystallographic data for compound **1**; CCDC reference 2068012.

Supplementary Data 2 Crystallographic data for compound (*S*,*S*)-**2**; CCDC reference 2068015.

Supplementary Data 3 Crystallographic data for compound (*R*,*R*)-**2**; CCDC reference 2068016.

Supplementary Data 4 Crystallographic data for compound **4**; CCDC reference 2068013.

Supplementary Data 5 Crystallographic data for compound (*R,S*)-**5**; CCDC reference 2068014.

Supplementary Data 6 Crystallographic data for compound **7**; CCDC reference 2068017.

Supplementary Data 7 Crystallographic data for compound **L**_**BB1**_PdCl_2_; CCDC reference 2068018.

Supplementary Data 8 Crystallographic data for compound (*C*,*R*,*S*)-**L**_**BB1**_RuCp(NCMe)·PF_6_; CCDC reference 2068020.

Supplementary Data 9 Crystallographic data for compound **L**_**BB2**_RuCp(NCMe)·PF_6_; CCDC reference 2173984.

Supplementary Data 10 Crystallographic data for compound **S2**; CCDC reference 2068019.

Supplementary Data 11 Structure factors for compound **1**; CCDC reference 2068012.

Supplementary Data 12 Structure factors for compound (*S*,*S*)-**2**; CCDC reference 2068015.

Supplementary Data 13 Structure factors for compound (*R*,*R*)-**2**; CCDC reference 2068016.

Supplementary Data 14 Structure factors for compound **4**; CCDC reference 2068013.

Supplementary Data 15 Structure factors for compound (*R*,*S*)-**5**; CCDC reference 2068014.

Supplementary Data 16 Structure factors for compound **7**; CCDC reference 2068017.

Supplementary Data 17 Structure factors for compound **L**_**BB1**_PdCl_2_; CCDC reference 2068018.

Supplementary Data 18 Structure factors for compound (*C*,*R*,*S*)-**L**_**BB1**_RuCp(NCMe)·PF_6_; CCDC reference 2068020.

Supplementary Data 19 Structure factors for compound **L**_**BB2**_RuCp(NCMe)·PF_6_; CCDC reference 2173984.

Supplementary Data 20 Structure factors for compound **S2**; CCDC reference 2068019.

## Data Availability

Crystallographic data for the structures reported in this Article have been deposited at the Cambridge Crystallographic Data Centre, under deposition numbers CCDC 2068012 (**1**), 2068013 (**4**), 2068014 ((*R*,*S*)-**5**), 2068015 ((*S*,*S*)-**2**), 2068016 ((*R*,*R*)-**2**), 2068017 (**7**), 2068018 (**L**_**BB1**_PdCl_2_), 2068019 (**S2**), 2068020 ((*C*,*R*,*S*)-**L**_**BB1**_RuCp(NCMe)·PF_6_) and 2173984 (**L**_**BB2**_RuCp(NCMe)·PF_6_). Copies of the data can be obtained free of charge via https://www.ccdc.cam.ac.uk/structures/. All other data supporting the findings of this study are available within the paper and its Supplementary Information.
