# Supplementary material for: Control of dynamic sp3-C stereochemistry
Source: Nat Chem. 2023 Mar 13;15(5):615–24. doi: 10.1038/s41557-023-01156-7 (PMC10159849; doi:10.1038/s41557-023-01156-7)
Supplement: Supplementary file 1 — Supplementary Figs. 1–96, Tables 1–41, schemes 1–5, discussion and experimental procedures. [file 41557_2023_1156_MOESM1_ESM.pdf]

nature chemistry

Article

https://doi.org/10.1038/s41557-023-01156-7

Control of dynamic *sp*^{3}-C stereochemistry

In the format provided by the authors and unedited

Table of Contents

1. General Methods S1
2. Synthetic Procedures S4
3. ^{1}H, ^{13}C, ^{19}F and ^{31}P NMR Spectroscopic Characterisation of Synthesised Compounds S36
    3.1 Structural Assignment by Two-Dimensional NMR S88
4. Enantioselective Ion-Pair Catalysis S90
5. High-Performance Liquid Chromatography S91
6. Additional NMR Spectroscopic Measurements S95
    6.1 *In Situ* Formation of Diazinane **8** S95
    6.2 Desymmetrisation of the Barbaralyl Core S96
    6.3 Variable-Temperature NMR Spectroscopy of (*R*,*S*)/(*S*,*S*)-**2** S98
    6.4 ^{13}C NMR Spectroscopic Comparison of (*R*,*S*)/(*S*,*S*)-**2** S101
    6.5 Dynamic NMR Spectroscopy of (*A*,*S*,*S*)/(*C*,*R*,*S*)-$\mathbf{L_{BB1}}$$PdCl_2$ S104
    6.6 Dynamic NMR Spectroscopy of (*C*,*R*,*S*)-$\mathbf{L_{BB1}}$RuCp(NCMe)·PF_6 S107
    6.7 NMR Measurements of (*A*,*S*)/(*C*,*R*)-$\mathbf{L_{BB2}}$RuCp(NCMe)·PF_6 with Chiral Counterions S111
7. Photoluminescence Quantum Yield (PLQY) Measurements of (*C*,*R*,*S*)-$\mathbf{L_{BB1}}$RuCp(NCMe)·PF_6 S113
8. X-Ray Crystallographic Analysis S115
    8.1. (*R*,*S*)-**1** S115
    8.2. (*S*,*S*)-**2** S116
    8.3. (*R*,*R*)-**2** S118
    8.4. (*R*,*R*)/(*S*,*S*)-**4** S120
    8.5. **7** S121
    8.6. **S1** S123
    8.7. (*R*,*S*)-**5** S124
    8.8. (*A*,*S*,*S*)/(*C*,*R*,*S*)-$\mathbf{L_{BB1}}$$PdCl_2$ S126
    8.9. (*C*,*R*,*S*)-$\mathbf{L_{BB1}}$RuCp(NCMe)·PF_6 S129
    8.10. (*A*,*S*)/(*C*,*R*)-$\mathbf{L_{BB2}}$RuCp(NCMe)·PF_6 S131
9. *In Silico* Modelling S132
    9.1 General Methods S132
    9.2 Optimised Structures S134
    9.3 Conformational Search Results S186
10. References S187

1. General Methods

All reagents were purchased from commercial suppliers (Sigma-Aldrich, Acros Organics, or Alfa Aesar) and used without further purification. Sodium bis[(*S*)-1,1′-bis-2-naphtholato]borate (*S*-BORBIN),[1] (±)-5-(dinaphtho[2,1-*d*:1',2'-*f*][1,3,2]dioxaphosphepin-4-yl)-5*H*-dibenzo[*b*,*f*]azepine ((±)-Carreira's Ligand or (±)-**CL**),[2] (±)-1,1′-binaphthyl-2,2′-diyl hydrogenphosphate ((±)-BDHP)[3], (±)-1-(naphthalen-2-yl)prop-2-en-1-ol [**10**][4] and cyclopent-3-en-1-ammonium chloride (**S4**)[5] were prepared according to literature procedures. Analytical thin-layer chromatography (TLC) was performed on neutral aluminium sheet silica gel plates and visualised under UV irradiation (254 nm). Nuclear magnetic resonance (NMR) spectra were recorded using a Bruker Advance (III)-400 (^{1}H 400.130 MHz and ^{13}C 100.613 MHz), Varian Inova-500 (^{1}H 500.130 MHz and ^{13}C 125.758 MHz), Varian VNMRS-600 (^{1}H 600.130 MHz and ^{13}C 150.903 MHz) or a Varian VNMRS-700 (^{1}H 700.130 MHz and ^{13}C 176.048 MHz) spectrometer, at a constant temperature of 298 K unless otherwise stated. For variable-temperature (VT) measurements, operating temperatures were calibrated using an internal calibration solution of MeOH and glycerol. Chemical shifts (δ) are reported in parts per million (ppm) relative to the signals corresponding to residual non-deuterated solvents [$CDCl_3$: δ = 7.26 or 77.16. CD_3CN: δ = 1.94, 1.32 or 118.26]. Coupling constants (*J*) are reported in Hertz (Hz). ^{13}C NMR experiments were proton-decoupled, whereas ^{19}F NMR experiments are coupled and generally referenced to an internal standard, hexafluorobenzene (HFB, δ = –164.99 ppm). ^{31}P NMR experiments are decoupled for compounds (*R*,*S*)/(*S*,*S*)-**L$_{BB}$**, (*R*,*S*)/(*S*,*S*)-**L$_{BB1}$**AuCl, (*A*,*R*,*S*)/(*C*,*S*,*S*)-**L$_{BB11}$**$PdCl_2$ and (*R*)/(*S*)-**L$_{BB2}$**, but coupled for compounds (*C*,*R*,*S*)/(*S*,*S*)-**L$_{BB1}$**RuCp(NCMe)·PF_6 and (*A*,*S*)/(*C*,*R*)-**L$_{BB2}$**RuCp(NCMe)·PF_6. Assignments of ^{1}H and ^{13}C NMR signals were accomplished by two-dimensional (2D) NMR spectroscopy (COSY, NOESY, HSQC, HMBC). NMR spectra were processed using MestReNova version 10.0. Data are reported as follows: chemical shift; multiplicity; coupling constants; integral and assignment. A Solid-State ^{13}C NMR spectrum was recorded on

Bruker Advance III HD spectrometer at 100.63 MHz with a 3.2 mm (rotor o.d.) magic-angle spinning probe at a spin rate of 10 kHz. The spectrum was recorded using cross-polarisation with total suppression of spinning sidebands with a contact time of 1-ms and a recycle delay of 120-s. The spectrum was recorded at ambient probe temperature (approximately 25 °C) and at a spin rate of 10 kHz. Spectra are reported relative to an external sample of neat tetramethylsilane (referencing was carried out by setting the high-frequency signal from adamantane to 38.5 ppm). Low-resolution Atmospheric Solids Analysis Probe (ASAP)-MS were performed using a Waters Xevo QTOF equipped with an ASAP. High-resolution (HR) electrospray ionisation (HR-ESI) and ASAP (HR-ASAP) mass spectra were measured using a Waters LCT Premier XE high resolution, accurate mass UPLC ES MS (also with ASAP ion source). Melting points were recorded using a Gallenkamp (Sanyo) apparatus and are uncorrected. Chiral high-performance liquid chromatography (HPLC) was run on a PerkinElmer Series 200 HPLC system with diode array detector using a Diacel ChiralPak AD column (250 cm × 4.6 mm, 5µm – standard column) eluting with hexanes–^{i}PrOH (9:1) including 0.1% trifluoroacetic acid (TFA) in the mobile phase with a flow rate of 1 mL per minute. The data was evaluated using software called TotalChrom with extraction of the chromatogram at 254 nm. Absorption spectra were measured on a Shimadzu UV-3600 UV-VIS-NIR spectrophotometer and photoluminescence spectra were measured a Jobin Yvon Fluorolog. The X-ray single-crystal diffraction data were collected at a temperature of 120.0(2) K using λMoKα radiation (λ =0.71073Å). Compounds **1**, (*S*,*R*)-**5**, (*S*,*R*)-**2** and (*A*,*S*)/(*C*,*R*)-**L$_{\mathbf{BB2}}$**RuCp(NCMe)·PF_6 were collected on a Bruker D8Venture (Photon100 CMOS detector, IµS-microsource, focusing mirrors) diffractometer. Compounds **S1**, (*A*,*R*,*S*)/(*C*,*S*,*S*)-**L$_{\mathbf{BB11}}$**$PdCl_2$ and (*C*,*R*,*S*)-**L$_{\mathbf{BB1}}$**RuCp(NCMe)·PF_6 were collected on a Photon III MM (CPAD detector, IµS-III-microsource, focusing mirrors) diffractometer. Compounds (*R*,*S*)-**2**, (*R*,*R*)/(*S*,*S*)-**4** and **7** were collected on an Agilent XCalibur (Sapphire-3 CCD detector, fine-focus sealed tube, graphite monochromator)

diffractometer. All diffractometers were equipped with Cryostream (Oxford Cryosystems) open-flow nitrogen cryostats. All structures were solved by direct method and refined by full-matrix least squares on F^2 for all data using Olex2[6] and SHELX[7] software. All non-hydrogen atoms were refined anisotropically, hydrogen atoms in structures (*S*,*R*)-**2**, (*R*,*S*)-**2**, **S1** and **7** were freely refined in isotropic approximation, the hydrogen atoms in remaining structures were placed at the calculated positions and refined in riding mode. Crystallographic data for these structures have been deposited within the Cambridge Crystallographic Data Centre as supplementary publication CCDC-2068012–2068020 and 2173984.

2. Synthetic Procedures

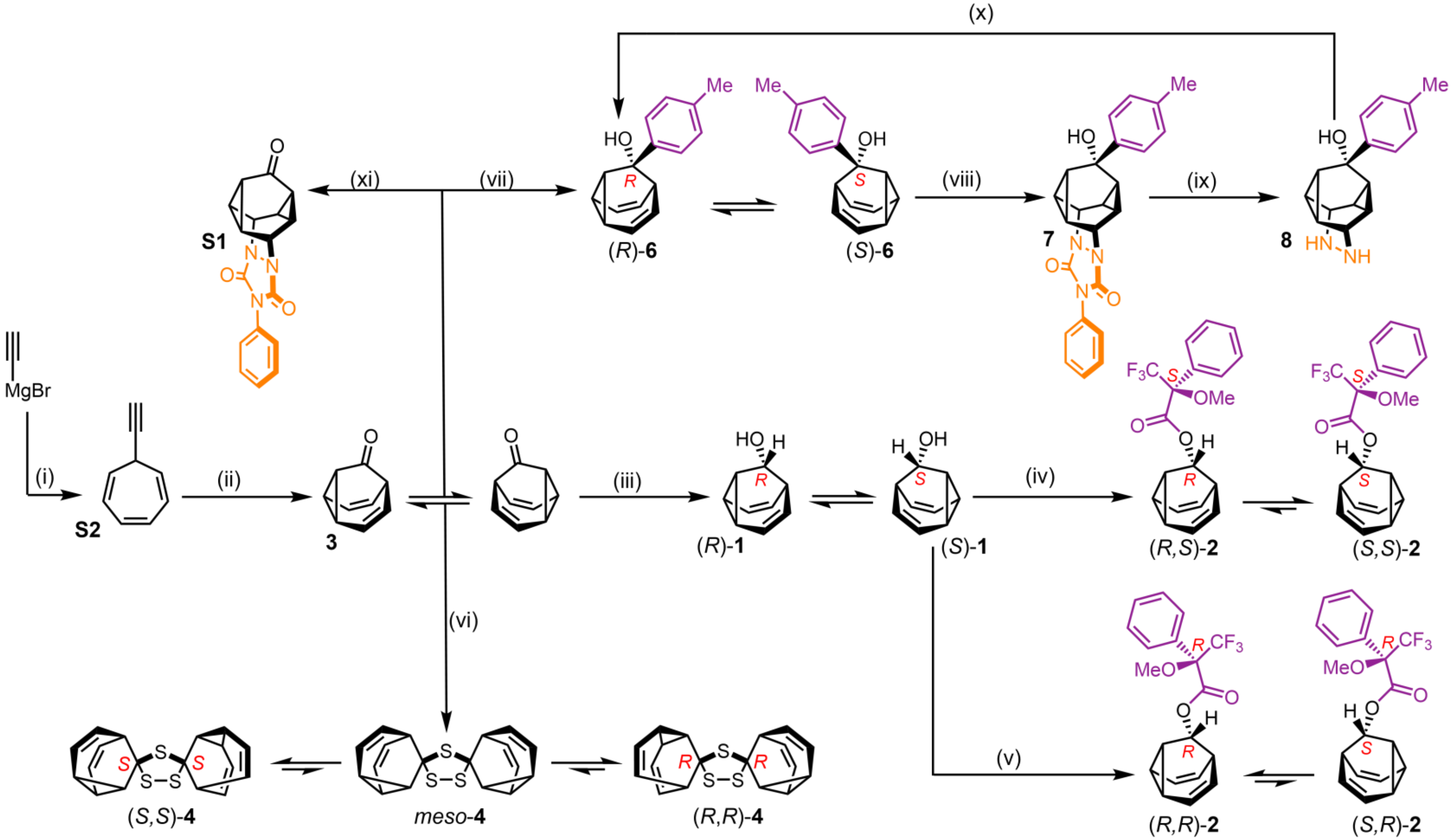

Scheme S1. Synthesis of **S2**, **3**, (*R*)/(*S*)-**1**, (*R*,*S*)/(*S*,*S*)-**2**, (*R*,*R*)/(*S*,*R*)-**2**, (*R,R*)/*meso*/(*S,S*)-**4**, *(R)*/(*S*)-**6**, **7**, **8** and **S1** according to modified literature procedures.[8,9] Reagents and conditions: (i) 1. LiCl / THF / –78 °C / 40 min, 2. Tropylium tetrafluoroborate / –78 °C to rt, (ii) IPrAu(MeCN)BF_4 (5 mol%) / Ph_2SO / CH_2Cl_2 / rt / 16 h, (iii) $LiAlH_4$ / Et_2O / 0 °C / 3 h, (iv) 1. $(COCl)_2$ / (*S*)-(+)-Mosher's acid / hexanes / DMF / 90 min / rt, 2. DMAP / $CHCl_3$ / Et_3N / rt / 5 d, (v) 1. $(COCl)_2$ / (*R*)-(+)-Mosher's acid / hexanes / DMF / 90 min / rt, 2. DMAP / $CHCl_3$ / Et_3N / rt / 5 d, (vi) Lawesson's Reagent / PhMe / 110 °C / 18 h, (vii) 1. Mg turnings / THF / 1-bromo-4-fluorobenzene / reflux to rt. 2. 0 °C to rt, (viii) PTAD / CH_2Cl_2 / 50 °C / 24 h, (ix) NaOH / ^{i}PrOH / 85 °C / 24 h, (x) $HCl_{(aq)}$ / $CuCl_2$ in H_2O / 0 °C / 4 h and (xi) PTAD / CH_2Cl_2 / 50 °C / 72 h. IPr = 1,3-bis(2,6-diisopropylphenyl)imidazol-2-ylidene. Lawesson's reagent = 2,4-bis(4-methoxyphenyl)-2,4-dithioxo-1,3,2,4-dithiadiphosphetane. PTAD = 4-phenyl-1,2,4-triazoline-3,5-dione

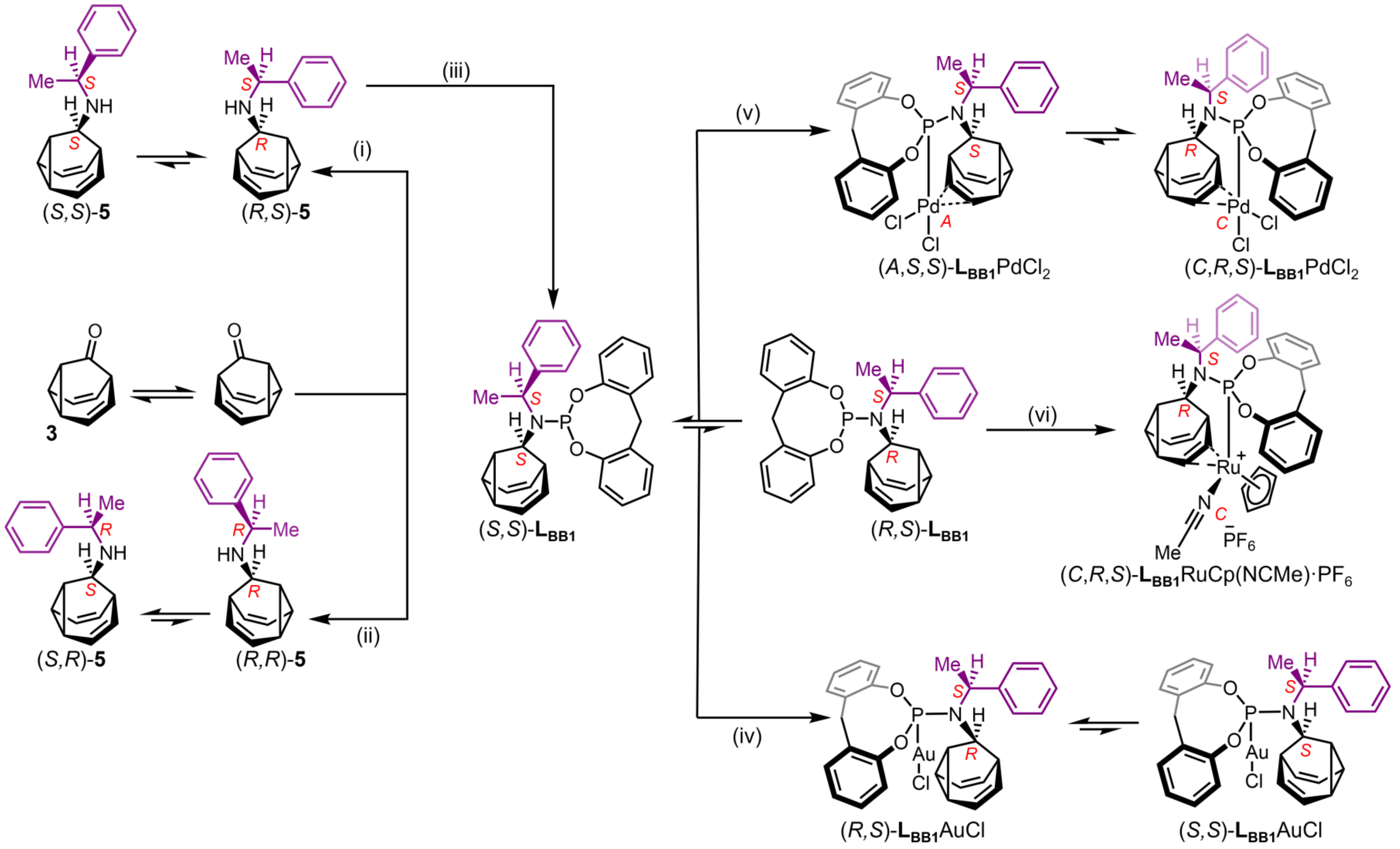

Scheme S2. Synthesis of (*S*,*S*)/(*R*,*S*)-**5**, (*S*,*R*)/(*R*,*R*)-**5**, (*R*,*S*)/(*S*,*S*)-$\mathbf{L_{BB1}}$, (*R*,*S*)/(*S*,*S*)-$\mathbf{L_{BB1}}$AuCl, (*A*,*S*,*S*)/(*C*,*R*,*S*)-$\mathbf{L_{BB1}}$$PdCl_2$ and (*C*,*R*,*S*)-$\mathbf{L_{BB1}}$RuCp(NCMe)·PF_6. Reagents and conditions: (i) 1. (*S*)-(+)-1-phenylethylamine / CH_3COOH, / MeOH / 30 min / rt, 2. $NaBH_3CN$ / 16 h / 100 °C, (ii) 1. (*R*)-(–)-1-phenylethylamine / CH_3COOH / MeOH / 30 min / rt, 2. $NaBH_3CN$ / 13 d / rt, (iii) 1. PCl_3 / Et_3N / CH_2Cl_2 / 0 °C / 3 h, 2. 2,2′-methylenediphenol / rt / 16 h, (iv) Me_2S·AuCl / $CDCl_3$ / rt / 10 min, (v) $PdCl_2$·MeCN **/** $CDCl_3$ / rt / 15 min and (vi) $CpRu(NCMe)_3$·PF_6 / $CDCl_3$ / rt / 5 min.

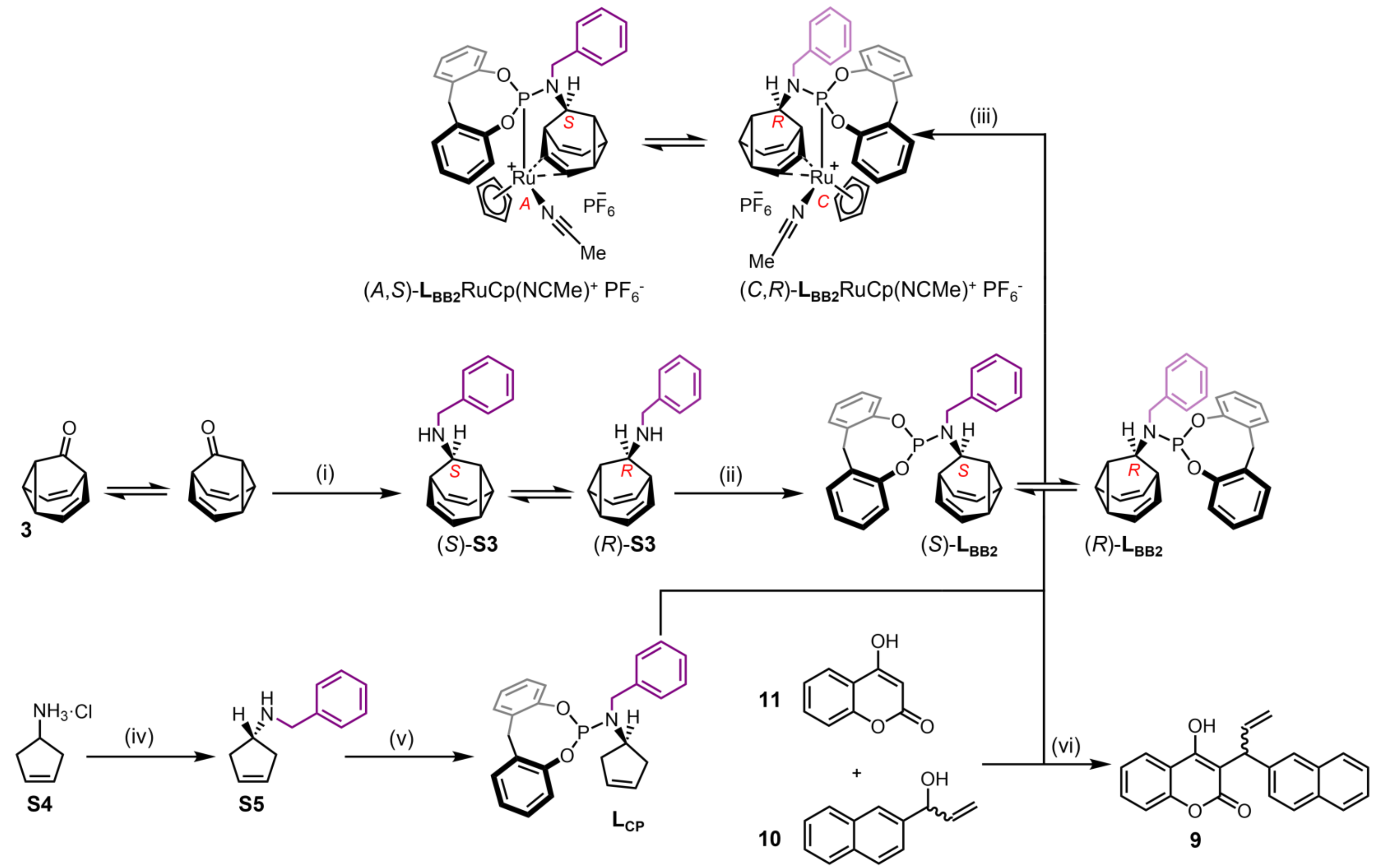

Scheme S3. Synthesis of (*R*)/(*S*)-**S3**, (*R*)/(*S*)-**L$_{BB2}$**, (*A*,*S*)/(*C*,*R*)-**L$_{BB2}$**RuCp(NCMe)·PF_6, **S5**, **L$_{CP}$** and **9**. Reagents and conditions: (i) 1. Benzylamine / AcOH / MeOH / 30 min, rt. 2. $NaBH_3CN$ / 4 d / rt, (ii) 1. PCl_3 / Et_3N / CH_2Cl_2 / 0 °C / 3 h, 2. 2,2'-methylenediphenol / rt / 16 h, (iii) $CpRu(NCMe)_3\cdot PF_6$ / $CDCl_3$ / rt / 5 min, (iv) 1. Benzaldehyde / AcOH / MeOH / rt / 30 min, 2. $NaBH_3CN$ / rt / 36 h, (v) 1. PCl_3 / Et_3N / CH_2Cl_2 / 0 °C / 3 h, 2. 2,2'-methylenediphenol / rt / 16 h and (vi) 1. $[Ir(1,5\text{-cod})Cl]_2$ / **Ligand** / THF / 30 min / rt, 2. (*R*)-(−)1,1'-Binaphthyl-2,2'-diyl hydrogenphosphate ((*R*)-(−)BDHP) / rt / 24 h.

7-Ethynylcyclohepta-1,3,5-triene [**S2**]:

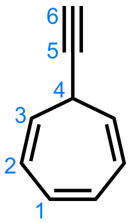

Anhydrous LiCl (0.524 g, 12.40 mmol) was placed in an oven-dried round-bottomed flask fitted with a septum under an N_2 atmosphere. Anhydrous THF was added (38 mL) and the resulting solution was cooled to –78 °C before adding a solution of ethynyl magnesium bromide (22.5 mL, 12.2 mmol, 0.5 M in THF) and stirring for 10 min at this temperature. Tropylium tetrafluoroborate (1.00 g, 5.62 mmol) was added to the reaction mixture and the reaction mixture was stirred for 16 h, where the temperature was raised to rt, following removal of the ice bath. The reaction was quenched with a saturated aqueous solution of NH_4Cl (20 mL), then extracted with Et_2O (3 × 30 mL). The combined organic extracts were then dried over $MgSO_4$, filtered and the solvent removed under reduced pressure (rotary evaporator bath at 16 °C, ≥ 100 mbar). The crude residue was purified by column chromatography (Teledyne Isco CombiFlash Rf+ system, 24 g SiO_2, *n*-pentane) to give the title compound as a colouress oil (539 mg, 4.6 mmol, 83%). **^{1}H NMR** (600 MHz, $CDCl_3$) δ 6.66 (dd, *J* = 3.7, 2.7 Hz, 2H, H_1), 6.27 – 6.07 (m, 2H, H_2), 5.44 – 5.20 (m, 2H, H_3), 2.64 – 2.42 (m, 1H, H_4), 2.17 (d, *J* = 2.6 Hz, 1H, H_6). **^{13}C NMR** (151 MHz, $CDCl_3$) δ 131.2 (C_1), 125.1 (C_2), 123.0 (C_3), 85.8 (C_5), 68.5 (C_6), 31.5 (C_4). **HRMS-ASAP** *m/z* = 117.0701 $[M+H]^+$ (calculated for C_9H_9 = 117.0704).

Spectroscopic data were consistent with those published previously.[8]

Note on labelling and assignment of NMR peaks:

In room temperature solutions, each of the barbaralanes exist as mixtures of two rapidly interconverting valence (stereo)isomers (or automers in the case of **3**). For derivatives with nondegenerate isomers, the NMR spectroscopic assignments below are labelled according to numbering of the major species. However, the species are in fast exchange on account of rapid strain-

assisted Cope rearrangement. The chemical shifts of each nucleus are representative of the time-averaged chemical environment they experience as part of the two isomers (or more in the case of $\mathbf{L_{BB1}}$RuCp(NCMe)·PF_6). ^{1}H and ^{13}C NMR peaks have been assigned to nuclei unambiguously with the aid of two-dimensional (2D) NMR spectra where possible.

Tricyclo[3.3.1.0^{2,8}]nona-3,6-dien-9-one [**3**]:

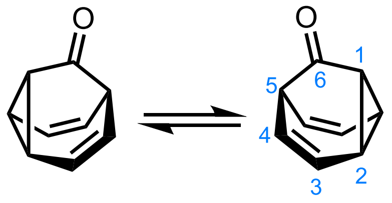

7-Ethynylcyclohepta-1,3,5-triene [**S2**] (1.0 g, 8.68 mmol) and diphenyl sulphoxide (3.51 g, 17.0 mmol) were charged in a flask and dissolved in anhydrous CH_2Cl_2 (15 mL) at 25 °C, with no particular precautions taken to exclude air. (Acetonitrile)[1,3-bis(2,6-diisopropylphenyl)-imidazol-2-ylidene]gold(I) tetrafluoroborate (309 mg, 0.43 mmol, 5 mol%) was added in one portion at the same temperature and the reaction mixture was stirred for 16 h. The reaction was quenched with 10 drops of Et_3N and the solvent was removed under reduced pressure. The crude residue was purified by column chromatography (Teledyne Isco CombiFlash Rf+ system, 24 g SiO_2, hexanes−EtOAc, gradient elution) to give the title compound as a light yellow solid (685 mg, 5.18 mmol, 60%). **M. P.** 55 – 57 °C (lit.[8] 50 – 51 °C). **^{1}H NMR** (700 MHz, $CDCl_3$) δ 5.97 – 5.51 (m, 2H, H_1 and H_5), 4.32 (br s, 4H, H_2 and H_4), 2.90 – 2.55 (m, 2H, H_3). **^{13}C NMR** (176 MHz, $CDCl_3$) δ 211.0 (C_6), 121.7 (C_2 and C_4, or C_3), 121.5 (C_2 and C_4, or C_3), 38.3 (C_1 and C_5). **HRMS-ASAP** *m/z* = 133.0648 $[M-H]^+$ (calculated for C_9H_9O = 133.0653).

Spectroscopic data were consistent with those published previously.[8]

Tricyclo[3.3.1.0^{2,8}]nona-3,6-dien-9-ol [(*R*)/(*S*)-**1**]:

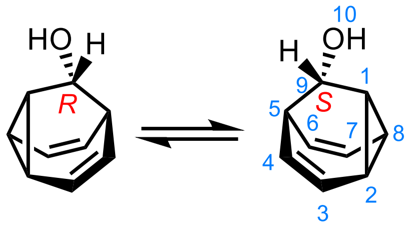

$LiAlH_4$ (120 mg, 3.16 mmol) was placed in an oven-dried round-bottomed flask fitted with a septum under a N_2 atmosphere and suspended in anhydrous Et_2O (5 mL). The mixture was cooled to 0 °C and then a solution of tricyclo[3.3.1.0^{2,8}]nona-3,6-dien-9-one [**3**] (200 mg, 1.51 mmol) in anhydrous Et_2O (5 mL) was added dropwise. The reaction mixture was stirred at this temperature for 3 h. The reaction was quenched with a saturated aqueous solution of potassium sodium tartrate (25 mL) and allowed to warm to rt before being extracted with Et_2O (3 × 30 mL). The combined organic extracts were dried over $MgSO_4$ and the solvent was removed under reduced pressure (rotary evaporator bath at 16 °C, ≥ 100 mbar). The crude residue was purified by column chromatography (Teledyne Isco CombiFlash Rf+ system, 24 g SiO_2, *n*-pentane−Et_2O, gradient elution) to give the title compound as a white solid (159 mg, 1.18 mmol, 78%). **M. P.** 82 – 84 °C (lit.[8] 86 – 88 °C). **^{1}H NMR** (400 MHz, $CDCl_3$) δ 5.88 (t, J = 7.9 Hz, 1H, H_3), 5.54 (t, J = 7.6 Hz, 1H, H_7), 4.16 – 4.04 (m, 2H, H_6 and H_8), 4.02 – 3.92 (m, 2H, H_2 and H_4), 3.61 (d, J = 3.3 Hz, 1H, H_9), 2.59 – 2.47 (m, 2H, H_1 and H_5), 1.14 (s, 1H, H_{10}). **^{13}C NMR** (151 MHz, $CDCl_3$) δ 123.3 (C_3), 120.9 (C_7), 76.4 (C_6 and C_8), 72.4 (C_2 and C_4), 62.5 (C_9), 31.8 (C_1 and C_5). **HRMS-ASAP** *m/z* = 117.0699 [M-OH]$^+$, calculated for C_9H_9: 117.0704.

Spectroscopic data were consistent with those published previously.[8]

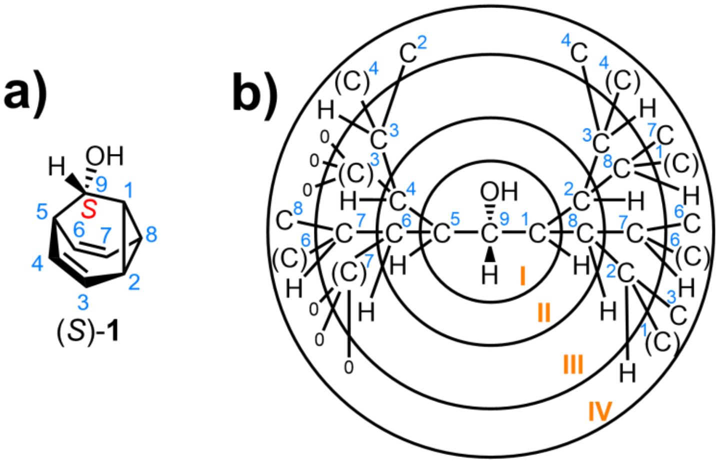

Figure S1. (a) Structural formula of (*S*)-**1** showing numbering scheme for C atoms. (b) Hierarchical diagraph used to assign the precedence of position 1 over position 5 according to the Cahn–Ingold–Prelog priority rules.

Assignment of stereochemical descriptors for the barbaralanes follows the Cahn–Ingold–Prelog priority rules. Taking position 9 of (*S*)-**1** as an example (Figure S1a), the priorities are assigned as O > C_1 > C_5 > H. A hierarchical diagraph (Figure S1b) aids in differentiating C_1 and C_5. The branch of the of the diagraph for C_5 has duplicate atoms in sphere III (corresponding to the C–C double bonds), leading to phantom atoms (atomic number 0) in sphere IV. The first duplicate atoms for C_1 arise in sphere IV – in this case, the duplicates correspond to (i) the C–C double bonds and (ii) the branches where the diagraph arrives back at a position already encountered of the cyclopropyl ring. Consequently, the first phantom atoms for the C_1 branch arise in sphere V. Therefore, sphere IV is the first point of difference, in which the C atoms of the C_1 branch have precedence over the phantom atoms of the C_5 branch.

Tricyclo[3.3.1.02,8]nona-3,6-dien-9-yl (*S*)-3,3,3-trifluoro-2-methoxy-2-phenylproponate [(*R*,*S*)/(*S*,*S*)-2]:

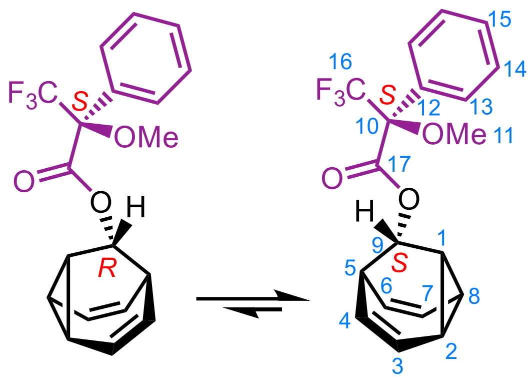

In an oven-dried round-bottomed flask fitted with a septum under a N_2 atmosphere, oxalyl chloride (50 µL, 0.58 mmol) was added to a stirred solution of (*S*)-(–)-Mosher's acid (28 mg, 0.12 mmol) in anhydrous hexanes (5 mL) and anhydrous DMF (10 µL, 0.13 mmol). The reaction mixture was left to stir for 90 min at rt before being cooled to −20 °C in a freezer for 60 h. The reaction mixture was filtered and the filtrate was concentrated under reduced pressure. This residue, tricyclo[3.3.1.$0^{2,8}$]nona-3,6-dien-9-ol [(*R*)/(*S*)-**1**] (18 mg, 0.13 mmol) and DMAP (13 mg, 0.11 mmol) were dissolved in anhydrous $CHCl_3$ (1.5 mL) before Et_3N (55 µL, 0.39 mmol) was added dropwise to the stirred reaction mixture at rt under an N_2 atmosphere. The reaction was left to stir for 5 d, after which it was quenched with a saturated aqueous solution of NH_4Cl (25 mL) and extracted with $CHCl_3$ (3 × 25 mL). The combined organic extracts were washed with H_2O (1 × 50 mL), dried over $MgSO_4$, filtered and the solvent was removed under reduced pressure. The crude residue was purified by chromatography using a short column of SiO_2 in a pasteur pipette and eluting with hexanes, to give the title compound as a white solid (25 mg, 0.07 mmol, 58%). **M. P.** 79 – 81°C. **^{1}H NMR** (600 MHz, $CDCl_3$) δ 7.56 – 7.50 (m, 2H, H_{14}), 7.42 – 7.35 (m, 3H, H_{13} and H_{15}), 5.77 (t, *J* = 7.9 Hz, 1H, H_7), 5.58 (t, *J* = 7.6 Hz, 1H, H_3), 4.93 (t, *J* = 3.1 Hz, 1H, H_9), 4.19 – 4.12 (m, 1H, H_2 or H_4), 4.10 – 4.04 (m, 1H, H_2 or H_4), 4.04 – 3.99 (m, 1H, H_6 or H_8), 3.97 – 3.93 (m, 1H, H_8 or H_6), 3.53 (s, 3H, H_{17}), 2.77 – 2.71 (m, 1H, H_5 or H_1), 2.67 – 2.61 (m, 1H, H_1 or H_5). **^{13}C NMR** (151 MHz, $CDCl_3$) δ 166.4 (C_{10}), 132.4 (C_{12}), 129.6 (C_{15}), 128.4 (C_{13}), 127.6 (C_{14}), 123.4 (q, J_{CF} = 288 Hz, C_{16}), 121.8 (C_7), 121.3 (C_3), 84.6 (q, J_{CF} = 27 Hz, C_{11}), 76.3 (C_2 or C_4), 74.4 (C_6 or C_8), 73.4 (C_2

or C4), 70.9 (C6 or C8), 69.8 (C9), 55. 6 (C17), 28.3 (C1 or C5), 27.8 (C5 or C1). **^{19}F NMR** (376 MHz, $CDCl_3$) δ −75.2 (s, F16). **HRMS-ASAP** *m/z* = 351.1213 $[M+H]^+$, calculated for $C_{19}H_{18}O_3F_3$: 351.1208.

Tricyclo[3.3.1.0^{2,8}]nona-3,6-dien-9-yl (*R*)-3,3,3-tri-fluoro-2-methoxy-2-phenylproponate [(*R*,*R*)/(*S*,*R*)-2]:

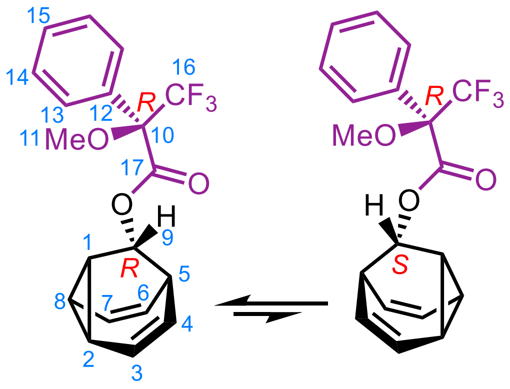

In an oven-dried round-bottomed flask fitted with a septum under a N_2 atmosphere, oxalyl chloride (100 µL, 1.18 mmol) was added to a stirred solution of (*R*)-(+)-Mosher's acid (56 mg, 0.24 mmol) in anhydrous hexanes (8 mL) and anhydrous DMF (4.8 µL, 0.06 mmol). The reaction mixture was left to stir for 7 h at rt before being cooled to −20 °C in a freezer for 16 h. The reaction mixture was filtered and the filtrate was concentrated under reduced pressure. This residue, tricyclo[3.3.1.0^{2,8}]nona-3,6-dien-9-ol [(*R*)/(*S*)-**1**] (40 mg, 0.30 mmol) and DMAP (26 mg, 0.21 mmol) were dissolved in anhydrous $CHCl_3$ (3 mL) before Et_3N (120 µL, 0.86 mmol) was added dropwise to the stirred reaction mixture at rt under an N_2 atmosphere. The reaction was left to stir for 3 d, after which it was quenched with a saturated aqueous solution of NH_4Cl (50 mL) and extracted with $CHCl_3$ (3 × 50 mL). The combined organic extracts were washed with H_2O (1 × 100 mL), dried over $MgSO_4$, filtered and the solvent was removed under reduced pressure. The crude residue was purified by chromatography using a short column of SiO_2 in a pasteur pipette and eluting with hexanes, to give the title compound as a white solid (66 mg, 0.19 mmol, 79%). **M. P.** 79 – 81°C. **^{1}H NMR** (700 MHz, $CDCl_3$) δ 7.60 – 7.50 (m, 2H, H14), 7.43 – 7.35 (m, 3H, H13 and H15), 5.77 (t, *J* = 7.9 Hz, 1H, H7), 5.58 (t, *J* = 7.6 Hz, 1H, H3),

4.94 (t, J = 3.2 Hz, 1H, H_9), 4.17 – 4.14 (m, 1H, H_2 or H_4), 4.08 – 4.04 (m, 1H, H_4 or H_2) 4.03 – 4.00 (m, 1H, H_6 or H_8), 3.97 – 3.94 (m, 1H, H_8 or H_6), 3.53 (s, 3H, H_{17}), 2.76 – 2.73 (m, 1H, H_5 or H_1), 2.65 – 2.62 (m, 1H, H_5 or H_1). **^{13}C NMR** (176 MHz, $CDCl_3$) δ 166.4 (C_{10}), 132.4 (C_{12}), 129.6 (C_{15}), 128.4 (C_{13}), 127.6 (C_{14}), 123.4 (q, J_{CF} = 288 Hz, C_{16}), 121.8 (C_7), 121.3 (C_3), 84.6 (q, J_{CF} = 27 Hz, C_{11}), 76.2 (C_2 or C_4), 74.4 (C_8 or C_6), 73. (C_2 6 or C_4), 70.9 (C_8 or C_6), 69.7 (C_9), 55.3 (C_{17}), 28.3 (C_1 or C_5), 27.8 (C_5 or C_1). **^{19}F NMR** (376 MHz, CDC_{13}) δ –75.2 (s, F_{16}). **HRMS-ASAP** *m/z* = 351.1203 $[M+H]^+$, calculated for $C_{19}H_{18}O_3F_3$: 351.1208.

Dispiro(1,2,4-trithiolane-3,2':5,2''-dibarbaralane) [(*R,R*)/*meso*/(*S,S*)-**4**]:

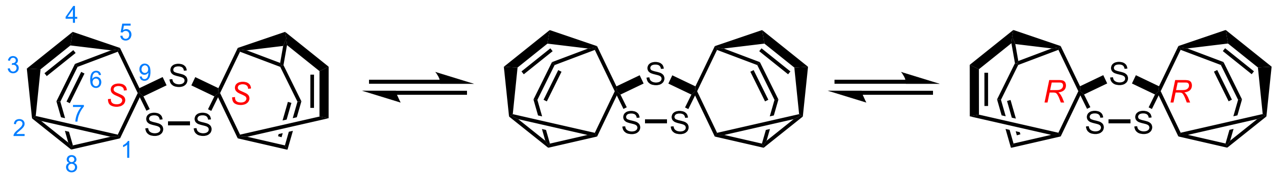

Tricyclo[3.3.1.0^{2,8}] nona-3,6-dien-9-one [**3**] (50 mg, 0.38 mmol) was dissolved in anhydrous PhMe (2 mL, 0.2 M) and 2,4-bis(4-methoxyphenyl)-2,4-dithioxo-1,3,2,4-dithiadiphosphetane (Lawesson's reagent, 100 mg, 0.25 mmol) was added to the stirred reaction mixture under an N_2 atmosphere. The reaction mixture was then heated to 110 °C and left to stir for 18 h. After cooling to rt, the solvent was removed under reduced pressure. The crude residue was purified by preparative TLC, eluting with hexanes−EtOAc (4:1) to give the title compound as a colourless solid (15 mg, 0.05 mmol, 13% yield). The material was further purified by recrystallisation, preparing a saturated MeCN solution at 80 °C and slowly cooling to rt over 2 d. **M. P.** 208 – 210 °C. **^{1}H NMR** (600 MHz, $CDCl_3$) δ 5.72 (t, J = 7.7 Hz, 4H, H_3 or H_4), 5.70 (t, J = 7.7 Hz, 4H, H_3 or H_4), 4.37 – 4.24 (m, 4H, H_2 or H_5), 4.24 – 4.17 (m, 4H, H_2 or H_5), 3.33 – 2.79 (m, 4H, H_1). **^{13}C NMR** (151 MHz, $CDCl_3$) δ 122.4 (C_3 or C_4), 121.1 (C_3 or C_4), 77.4 (C_2 or C_5), 77.3 (C_2 or C_5), 37.0 (C_1) [C_9 not observed due slow relaxation of

quaternary environment]. **HRMS-ASAP** *m/z* = 329.0495 [M+H]$^+$, calculated for $C_{18}H_{17}S_3$: 329.0492.

6-(4-Tolyl)bicyclo[3.2.2]nona-3,8-dien-6-ol [*(R)* /(*S*)-**6**]:

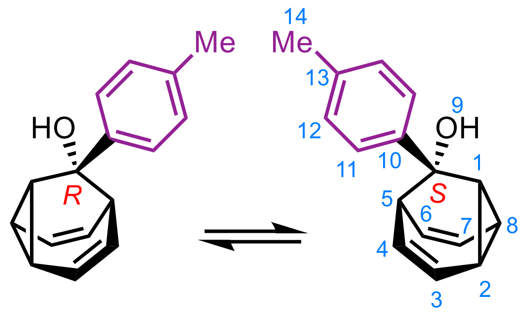

Magnesium turnings (100 mg, 4.54 mmol) and I_2 (57 mg, 0.23 mmol, 5 mol%) were placed in an oven-dried two-necked round-bottomed flask with a condenser and a septum under an N_2 atmosphere. The flask was gently heated with a heat gun until the I_2 started to sublime. The flask was cooled down to rt. A quarter of a solution of 4-bromotoluene (760 mg, 4.5 mmol) in anhydrous THF (10 mL) was added to the reaction mixture, which was heated until it reached reflux. Upon gentle reflux, the remaining solution of 4-bromotoluene in anhydrous THF was added dropwise over 30 min. The reaction mixture was heated at reflux for 30 min before cooling to rt. Tricyclo[3.3.1.0^{2,8}]nona-3,6-dien-9-one [**3**] (111 mg, 0.84 mmol) was transferred to an oven-dried round-bottomed flask, and the flask was purged with N_2. Anhydrous THF (10 mL) was added and the solution was cooled to 0 °C. The Grignard solution (prepared above) was added dropwise over 30 min to the tricyclo[3.3.1.0^{2,8}]nona-3,6-dien-9-one solution. The reaction mixture was stirred for 16 h, and the temperature was raised from 0 °C to rt, following removal of the ice bath. The reaction was quenched with a saturated aqueous solution of NH_4Cl (10 mL), then extracted with EtOAc (3 × 20 mL). The combined organic extracts were dried over $MgSO_4$, filtered and the solvent removed under reduced pressure. The crude residue was purified by column chromatography (Teledyne Isco CombiFlash Rf+ system, 24 g SiO_2, hexanes−CH_2Cl_2, gradient elution including 0.5% Et_3N in the elution) to yield the title compound as a cream-coloured solid (136 mg, 0.61 mmol,

80%). **M. P.** 59 – 61°C. **^{1}H NMR** (700 MHz, $CDCl_3$) δ 7.33 (d, *J* = 8.2 Hz, 2H, H_{11}), 7.12 (d, *J* = 7.9 Hz, 2H, H_{12}), 5.91 (t, *J* = 7.7 Hz, 1H, H_7), 5.58 (t, *J* = 7.6 Hz, 1H, H_3), 4.28 – 4.24 (m, 2H, H_8 and H_6), 4.24 – 4.21 (m, 2H, H_4 and H_2), 2.83 – 2.57 (m, 2H, H_5 and H_1), 2.33 (d, *J* = 0.7 Hz, 3H, H_{14}), 1.98 (s, 1H, H_9). **^{13}C NMR** (176 MHz, $CDCl_3$) δ 140.4 (C_{10}), 136.8 (C_{13}), 128.6 (C_{12}), 126.4 (C_{11}), 123.2 (C_7), 120.9 (C_3), 77.9 (C_4 and C_2), 75.5 (C_8 and C_6), 68.6 (C_9), 38.2 (C_5 and C_1), 21.2 (C_{14}). **HRMS-ASAP** *m/z* = 207.1154 $[M-OH]^+$, calculated for $C_{16}H_{15}$: 207.1174.

11-Hydroxy-11-(4-tolylphenyl)-5-phenyl-3,5,7-triazatetracyclo[8.2.2.0-^{2,8}.0^{3,7}]tetradecane-4,6-dione [**7**]:

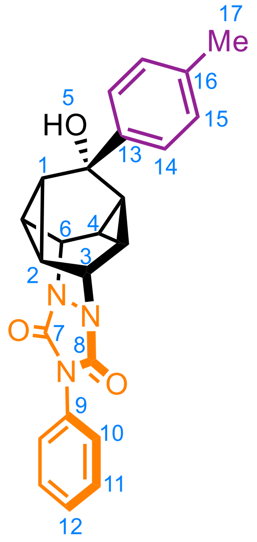

6-(4-Tolyl)bicyclo[3.2.2]nona-3,8-dien-6-ol [*(R)*/(*S*)-**6**] (15 mg, 0.07 mmol) and 4-phenyl-1,2,4-triazoline-3,5-dione (PTAD) (23 mg, 0.13 mmol) were dissolved in anhydrous CH_2Cl_2 (1.0 mL). The reaction mixture was heated to 50 °C and stirred for 24 h. The reaction mixture was cooled and diluted with CH_2Cl_2 (20 mL), then washed with saturated aqueous $NaHCO_3$ (3 × 30 mL). The organic phase was separated and dried over $MgSO_4$, filtered and the solvent removed under reduced pressure. The crude residue was purified by column chromatography (Teledyne Isco CombiFlash Rf+ system, 12 g SiO_2, CH_2Cl_2−EtOAc, gradient elution) to yield the title compound as a colourless solid (22 mg, 0.055 mmol, 85%). **M. P.** 186 – 188 °C (decomp.). **^{1}H NMR** (400 MHz, $CDCl_3$) δ 7.61 – 7.46 (m, 6H, H_{10}, H_{11} and H_{14}), 7.39 (t, *J* = 7.3 Hz, 1H, H_{12}), 7.30 – 7.23 (m, 2H, H_{15}), 5.24 (t, *J* = 5.4 Hz, 1H, H_6), 5.11 (t, *J* = 5.4 Hz, 1H, H_3), 2.41 (s, 3H, H_{17}), 2.14 (br s, 1H, H_5), 2.02 – 1.87 (m, 2H, H_2), 1.87 – 1.77 (m, 2H, H_4), 1.64 (t, *J* = 7.7 Hz, 2H, H_1). **^{13}C NMR** (151 MHz, CD_3CN) δ 156.5 (C_7 or C_8), 156.4 (C_7 or C_8), 145.0 (C_{13}), 137.7 (C_{16}), 133.1 (C_9), 129.9 (C_{15}), 129.8 (C_{10} or C_{11}), 129.2 (C_{12}),

127.2 (C_{10} or C_{11}), 126.2 (C_{14}), 65.4 (C_5), 50.4 (C_3), 49.9 (C_6), 27.4 (C_1), 21.0 (C_{17}), 18.8 (C_2), 16.0 (C_4). **HR-ESI MS** *m/z* = 400.1684 $[M+H]^+$, calculated for $C_{24}H_{22}N_3O_3^+$: 400.1661.

4-(*p*-Tolyl)decahydro-3,5,6-(epimethanetriyl)cyclopropa[*de*]cinnolin-4-ol [*(R)*/(*S*)-**8**] from **7**:

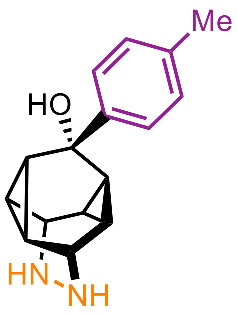

A solution of 11-hydroxy-11-(4-tolylphenyl)-5-phenyl-3,5,7-triazatetracyclo[8.2.2.0^{2,8}.0^{3,7}]tetra-decane-4,6-dione [**7**] (45 mg, 0.11 mmol) and NaOH (75 mg, 1.88 mmol) in *i*PrOH (6 mL) were heated to reflux for 24 h under N_2. Evidence of an intermediate diazinane [**8**] was observed by acquiring a crude ^{1}H NMR spectrum of an aliquot (Figure S62). The solution was cooled to 0 °C, then $HCl_{(aq)}$ (10%, 1.2 mL) and a solution of $CuCl_2$ (135 mg, 1.01 mmol) in H_2O (7.5 mL) was added. After stirring at rt for 4 h, a 35% solution of NH_3 in H_2O was added dropwise until a blue color persisted. The reaction mixture was cooled and extracted with EtOAc (3 × 15 mL). The organic phase washed with brine (50 mL) and H_2O (50 mL). The organic phase was dried over $MgSO_4$, filtered and the solvent removed under reduced pressure. The crude residue was purified by column chromatography (Teledyne Isco CombiFlash Rf+ system, 4 g SiO_2, hexanes−Et_2O, gradient elution) to yield 6-(4-tolyl)bicyclo[3.2.2]nona-3,8-dien-6-ol [*(R)*/(*S*)-**6**] as colourless solid (12 mg, 0.05 mmol, 48%). Characterisation data matched those shown above for *(R)*/(*S*)-**6**.

6-Phenyl-4,6,9-triazahexacyclo[8.5.0.0^{2,15}.0^{3,12}.0^{4,9}.0^{11,13}]pentadecane-5,8,14-trione [**S1**]:

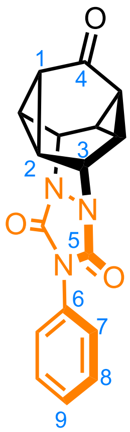

Tricyclo[3.3.1.0^{2,8}]nona-3,6-dien-9-one [**3**] (70 mg, 0.52 mmol) and 4-phenyl-1,2,4-triazoline-3,5-dione (PTAD) (180 mg, 1.03 mmol) were dissolved in anhydrous CH_2Cl_2 (2.5 mL). The reaction mixture was heated to 50 °C and stirred for 72 h. The reaction mixture was cooled and diluted with CH_2Cl_2 (20 mL), then washed with saturated aqueous $NaHCO_3$ (3 × 30 mL). The organic phase was separated and dried over $MgSO_4$, filtered and the solvent removed under reduced pressure. The crude residue was purified by column chromatography (Teledyne Isco CombiFlash Rf+ system, 12 g SiO_2, CH_2Cl_2−EtOAc, gradient elution) to yield the desired title as a colourless solid. (45 mg, 0.15 mmol, 28%). **M. P.** 171 – 173 °C (decomp.). **^{1}H NMR** (600 MHz, CD_3CN) δ 7.56 – 7.48 (m, 4H, H_8 and H_7), 7.47 – 7.42 (m, 1H, H_9), 5.62 – 4.64 (m, 2H, H_3), 2.33 (ddd, J = 7.8, 3.7, 2.7 Hz, 4H, H_2), 1.67 (t, J = 7.7 Hz, 2H, H_1). **^{13}C NMR** (151 MHz, CD_3CN) δ 202.4 (C_5), 156.8 (C_4), 132.8 (C_6), 123.0 (C_7), 129.4 (C_9), 127.3 (C_8), 48.9 (C_3), 27.4 (C_2), 24.7 (C_1). **HR-ESI MS** *m/z* = 308.1032 $[M+H]^+$, calculated for $C_{17}H_{14}N_3O_3^+$: 308.1035.

***N*-(*S*)-1-(Phenylethynyl)-tricyclo[3.3.1.0^{2,8}]-nona-3,6-dien-9-amine** [(*S*,*S*)/(*R*,*S*)-**5**]:

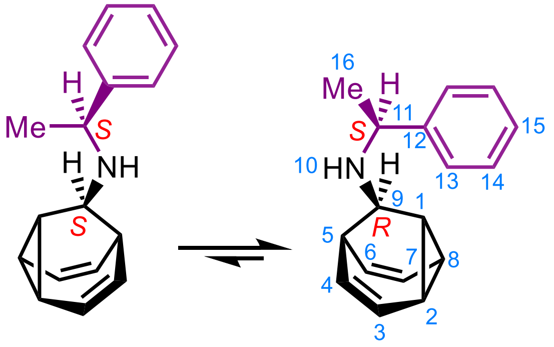

In an oven-dried round-bottomed flask fitted with a septum under a N_2 atmosphere, tricyclo[3.3.1.0^{2,8}]nona-3,6-dien-9-one [**3**] (80 mg, 0.61 mmol) and (*S*)-(–)-1-phenylethylamine (250 µL, 1.96 mmol) were dissolved in anhydrous MeOH (0.80 mL). Glacial acetic acid (25 µL) was added dropwise to the stirred reaction mixture and left

for 30 min. Sodium cyanoborohydride (50 mg, 0.80 mmol, 1.2 equiv.) was then added to the reaction mixture, which was left to stir for 16 h at 100 °C. The mixture was quenched with 5 drops of Et_3N and the solvent was removed under reduced pressure. This material was dissolved in an aqueous solution of $NaHCO_3$ (50 mL) and extracted with CH_2Cl_2 (5 × 25 mL). The combined organic extracts were washed with brine (1 × 50 mL), H_2O (1 × 50 mL), dried over $MgSO_4$, filtered and the solvent was removed under reduced pressure. The crude residue was purified by column chromatography (Teledyne Isco CombiFlash Rf+ system, 4 g SiO_2, hexanes–EtOAc, gradient including 1% Et_3N in the elution) to give the title compound as a colourless oil (71 mg, 0.30 mmol, 49%). **^{1}H NMR** (700 MHz, $CDCl_3$) δ 7.36 – 7.28 (m, 4H, H_{13} and H_{14}), 7.22 (tt, *J* = 7.2, 1.5 Hz, 1H, H_{15}), 5.81 (t, *J* = 7.8 Hz, 1H, H_7), 5.52 (t, *J* = 7.7 Hz, 1H, H_3), 4.23 – 4.20 (m, 1H, H_2 or H_4), 4.14 – 4.11 (m, 1H, H_2 or H_4), 3.88 (q, *J* = 6.5 Hz, 1H, H_{11}), 3.85 – 3.82 (m, 1H, H_8 or H_6), 3.76 – 3.72 (m, 1H, H_8 or H_6), 2.35 – 2.33 (m, 1H, H_9), 2.33 – 2.30 (m, 2H, H_1 and H_5), 1.28 (d, *J* = 6.5 Hz, 3H, H_{16}), 1.03 (br s, 1H, H_{10}). **^{13}C NMR** (176 MHz, $CDCl_3$) δ 146.1 (C_{12}), 128.5 (C_{13}), 126.9 (C_{15}), 126.9 (C_{14}), 123.1 (C_7), 121.1 (C_3), 81.4 (C_2 or C_4), 77.4 (C_8 or C_6), 71.2 (C_2 or C_4), 67.4 (C_8 or C_6), 54.9 (C_{11}), 44.6 (C_9), 29.7 (C_1 or C_5), 29.1 (C_5 or C_5), 25.3 (C_{16}). **HRMS-ASAP** *m/z* = 238.1592 [M+H]+, calculated for $C_{17}H_{20}N$: 238.1596.

***N*-(*R*)-1-(Phenylethynyl)-tricyclo[3.3.1.0^{2,8}]-nona-3,6-dien-9-amine** [(*R*,*R*)/(*R*,*R*)-**5**]:

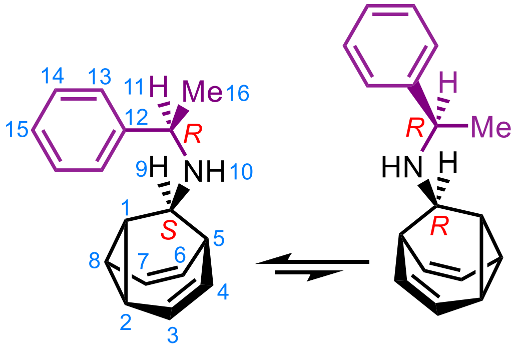

In an oven-dried round-bottomed flask fitted with a septum under a N_2 atmosphere, tricyclo[3.3.1.0^{2,8}]nona-3,6-dien-9-one (80 mg, 0.61 mmol) and (*R*)-(+)-1-phenylethylamine (250 µL, 1.96 mmol) were dissolved in anhydrous MeOH (0.80 mL). Glacial acetic acid (25 µL)

was added dropwise to the stirred reaction mixture and left for 30 min. Sodium cyanoborohydride (50 mg, 0.80 mmol, 1.2 equiv.) was then added to the reaction mixture, which was left to stir for 13 d at rt. The mixture was quenched with 5 drops of Et_3N and the solvent was removed under reduced pressure. This material was dissolved in an aqueous solution of $NaHCO_3$ (50 mL) and extracted with CH_2Cl_2 (5 × 25 mL). The combined organic extracts were washed with brine (1 × 50 mL), H_2O (1 × 50 mL), dried over $MgSO_4$, filtered and the solvent was removed under reduced pressure. The crude residue was purified by column chromatography (Teledyne Isco CombiFlash Rf+ system, 4 g SiO_2, hexanes–EtOAc, gradient including 1% Et_3N in the elution) to give the title compound as a colourless oil (112 mg, 0.47 mmol, 78%). **^{1}H NMR** (600 MHz, $CDCl_3$) δ 7.37 – 7.28 (m, 4H, H_{13} and H_{14}), 7.22 (tt, *J* = 7.1, 1.6 Hz, 1H, H_{15}), 5.81 (t, *J* = 7.8 Hz, 1H, H_7), 5.52 (t, *J* = 7.7 Hz, 1H, H_3), 4.23 – 4.20 (m, 1H, H_2 or H_4), 4.14 – 4.11 (m, 1H, H_2 or H_4), 3.88 (q, *J* = 6.5 Hz, 1H, H_{11}), 3.85 – 3.82 (m, 1H, H_6 or H_8), 3.76 – 3.72 (m, 1H, H_6 or H_8), 2.36 – 2.33 (m, 1H, H_9), 2.33 – 2.30 (m, 2H, H_1 and H_5), 1.28 (d, *J* = 6.5 Hz, 3H, H_{16}), 1.03 (br s, 1H, H_{10}). **^{13}C NMR** (151 MHz, $CDCl_3$) δ 146.1 (C_{12}), 128.5 (C_{13}), 126.9 (C_{15}), 126.8 (C_{14}), 123.1 (C_7), 121.1 (C_3), 81.4 (C_2 or C_4), 77.5 (C_6 or C_8), 71.2 (C_2 or C_4), 67.4 (C_6 or C_8), 58.4 (C_{11}), 45.2 (C_9), 29.7 (C_1 or C_5), 29.1 (C_5 or C_1), 25.3 (C_{16}). **HRMS-ASAP** *m/z* = 238.1592 $[M+H]^+$, calculated for $C_{17}H_{20}N$: 238.1596.

***N*-(1-Phenylethyl)-*N*-{tricyclo[3.3.1.0^{2,8}]nona-2,6-dien-9-yl}-9,11-dioxa-10-phosphatricyclo-[10. 4.0.0^{3,8}]hexadeca-1(16),3,5,7,12,14-hexaen-10-amine** [(*R*,*S*)/(*S*,*S*)-**L$_{BB1}$**]:

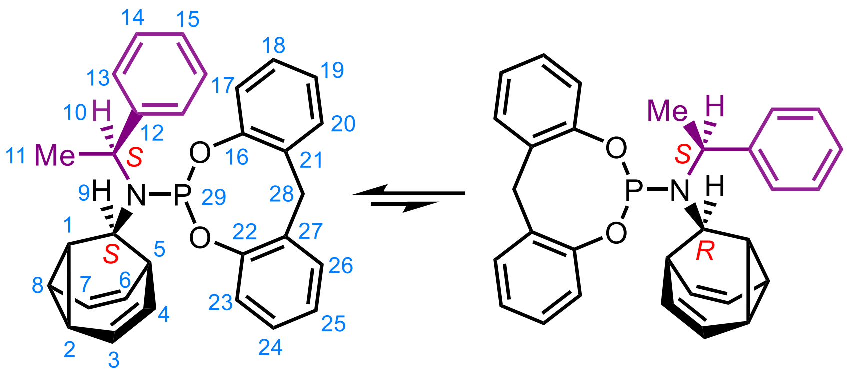

PCl_3 (27.5 mg, 200 μmol), Et_3N (162 mg, 1.6 mmol) and anhydrous CH_2Cl_2 (2.0 mL) were placed in an oven-dried vial under an N_2 atmosphere and cooled to 0 °C. A solution of *N*-(*S*)-1-(phenylethynyl)-tricyclo[3.3.1.0^{2,8}]-nona-3,6-dien-9-amine [(*S*,*S*)/(*R*,*S*)-**5**] (47.5 mg, 200 μmol) in anhydrous CH_2Cl_2 (2.0 mL) was added dropwise by syringe over 10 min and the resulting mixture stirred at 0 °C for 3 h. A solution of 2,2′-methylenediphenol (40.0 mg, 200 μmol) in anhydrous CH_2Cl_2 (2.0 mL) was added dropwise by syringe over 10 min, then the resulting mixture was allowed to warm to rt and stirred for 16 h. The solution was poured into a mixture of H_2O (30 mL) and brine (5 mL), then extracted with CH_2Cl_2 (4 × 10 mL). The combined organic extracts were dried over K_2CO_3 then the solvent was removed under reduced pressure. The crude residue was purified by column chromatography (Teledyne Isco CombiFlash Rf+ system, 12 g neutral Al_2O_3, hexanes−CH_2Cl_2, gradient elution), giving the title compound as a colourless solid powder (41.4 mg, 89 μmol, 44%). The ligand was stored in a vial flushed with nitrogen to prevent degradation. **M. P.** 159 – 160 °C. **^{1}H NMR** (600 MHz, $CDCl_3$) δ 7.63 – 7.58 (m, 2H, H_{13}), 7.35 (t, *J* = 7.7 Hz, 2H, H_{14}), 7.31 (dt, *J* = 7.7, 2.1 Hz, 2H, H_{17} and H_{23}), 7.28 – 7.23 (m, 1H, H_{15}), 7.14 (t, *J* = 7.6 Hz, 2H, H_{19} and H_{25}), 7.03 – 6.97 (m, 4H, H_{18}, H_{20}, H_{26} and H_{24}), 5.81 (t, *J* = 7.8 Hz, 1H, H_3 or H_7), 5.48 (t,

J = 7.5 Hz, 1H, H_3 or H_7), 5.46 – 5.39 (m, 1H, H_{10}), 4.42 (dd, J = 12.8, 3.0 Hz, 1H, H_{28}), 4.09 (s, 2H, H_2 and/or H_4 and/or H_6 and/or H_8), 3.97 – 3.86 (m, 2H, H_2 and/or H_4 and/or H_6 and/or H_8), 3.51 (d, J = 12.8 Hz, 1H, H_{28}), 3.12 (d, J = 16.4 Hz, 1H, H_9), 2.65 – 2.59 (m, 1H, H_1 or H_5), 1.89 – 1.84 (m, 1H, H_1 or H_5), 1.73 (d, J = 7.1 Hz, 3H, H_{11}). **^{13}C NMR** (151 MHz, $CDCl_3$) δ 152.1 – 152.0 (m, 2C, C_{16} and C_{22}), 143.6 (C_{12}), 135.8 – 135.7 (m, 2C, C_{21} and C_{27}), 129.9 (d, J_{CP} = 7.4 Hz, 2C, C_{17} and C_{23}), 128.3 (C_{13}), 128.2 (C_{14}), 128.0 (2C, C_{19} and C_{25}), 126.8 (C_{15}), 124.8 (d, J_{CP} = 5.1 Hz, C_3), 124.4 (d, J_{CP} = 6.6 Hz, 2C, C_{20} and C_{28}), 123.2 (d, J_{CP} = 3.0 Hz, 2C, C_{18} and C_{24}), 121.5 (C_7), 74.3 (2C, C_2 and C_4), 73.2 (2C, C_6 and C_6), 51.9 (C_{10}), 45.1 (d, J_{CP} = 17.3 Hz, C_9), 34.3 (C_{28}), 31.7 (d, J_{CP} = 7.2 Hz, C_1 or C_5), 30.5 (d, J_{CP} = 5.5 Hz, C_1 or C_5), 20.2 (d, J_{CP} = 2.6 Hz, C_{11}). **^{31}P NMR** (243 MHz, $CDCl_3$) δ 139.3 (P_{29}). **HR-ESI-MS** *m/z* = 466.1923 $[M+H]^+$ (calculated for $C_{30}H_{29}NO_2P^+$ = 466.1936).

Chloro({10-[(1-phenylethyl)(tricyclco[3.3.1.0^{2,8}]nona-3,6-dien-9-yl})amino]-9-11-dioxa-10-phosphatricyclo-[10.4.0.0^{3,8}]hexadeca-1(16),3,5,7,12,14-hexaen-10-yl]gold [(*R*,*S*)/(*S*,*S*)-L_{BB1}AuCl]:

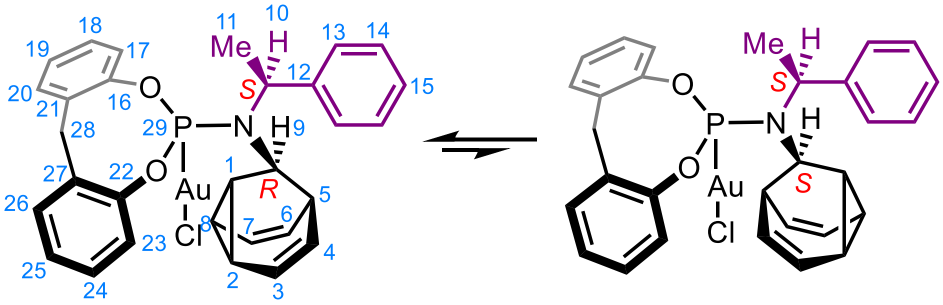

L_{BB1} (6.5 mg, 14 µmol) and $Me_2S \cdot AuCl$ (4.1 mg, 14 µmol) were dissolved in $CDCl_3$ (0.7 mL) and sonicated for 10 min to dissolve the solids. The mixture was then evaporated under reduced pressure and dried under high vacuum, to give the title compound as a pale yellow solid (9.1 mg, 13 µmol, 93%). **^{1}H NMR** (600 MHz, $CDCl_3$) δ 7.58 (d, J = 7.6 Hz, 2H, H_{13}), 7.42 (dd, J = 7.7, 7.7 Hz, 2H, H_{14}), 7.34 (t, J = 7.4 Hz, 1H, H_{15}), 7.28 (d, J = 7.4 Hz, 2H, H_{20} and H_{26}), 7.17 – 7.11 (m, 2H, H_{18}

and H_{24}), 7.11 – 7.06 (m, 2H, H_{19} and H_{25}), 6.87 (d, J = 8.1 Hz, 1H, H_{17} or H_{23}), 6.80 (d, J = 8.1 Hz, 1H, H_{17} or H_{23}), 5.92 (dd, J = 7.9, 7.9 Hz, 1H, H_7), 5.61 (dd, J = 7.6, 7.6 Hz, 1H, H_3), 5.14 (dq, J = 22.0, 7.0 Hz, 1H, H_{10}), 4.63 – 4.58 (m, 1H, H_6), 4.55 – 4.49 (m, 1H, H_4), 4.27 (dd, J = 13.0, 4.6 Hz, 1H, H_{28}), 4.02 – 3.96 (m, 1H, H_8), 3.94 (d, J = 12.7 Hz, 1H, H_9), 3.92 – 3.86 (m, 1H, H_2), 3.47 (d, J = 13.0 Hz, 1H, H_{28}), 2.86 – 2.80 (m, 1H, H_5), 2.60 – 2.55 (m, 1H, H_1), 1.96 (d, J = 6.9 Hz, 3H, H_{11}). **^{13}C NMR** (151 MHz, $CDCl_3$) δ 149.0 (d, J = 2.9 Hz, C_{16} or C_{22}), 148.6 (d, J_{CP} = 3.1 Hz, C_{16} or C_{22}), 142.9 (d, J_{CP} = 3.6 Hz, C_{12}), 134.7 (d, J_{CP} = 3.9 Hz, C_{21} or C_{27}), 134.6 (d, J_{CP} = 3.8 Hz, C_{21} or C_{27}), 130.1 (d, J_{CP} = 2.2 Hz, C_{20} or C_{26}), 130.1 (d, J_{CP} = 2.2 Hz, C_{20} or C_{26}), 129.1 (C_{18} or C_{24}), 129.1 (C_{18} or C_{24}), 128.7 (C_{14}), 127.7 (C_{13}), 127.6 (C_{15}), 126.8 (C_{19} or C_{25}), 126.8 (C_{19} or C_{25}), 123.6 (C_7), 123.2 (C_{17} or C_{23}), 123.2 (C_{17} or C_{23}), 121.7 (C_3), 84.8 (C_4), 81.0 (C_6), 53.1 (d, J_{CP} = 8.9 Hz, C_{10}), 47.9 (d, J_{CP} = 5.9 Hz, C_9), 33.5 (C_{28}), 31.1 (C_5), 28.3 (C_1), 21.7 (d, J = 3.4 Hz, C_{11}). **^{31}P NMR** (162 MHz, $CDCl_3$) δ 120.9 (P_{29}). **HR-ESI-MS** m/z = 720.1142 $[M+Na]^+$ (calculated for $C_{30}H_{28}AuClNNaO_2P^+$ = 720.1110).

Dichloro({10-[(1-phenylethyl)(tricyclco[3.3.1.0^{2,8}]nona-3,6-dien-9-yl})amino]-9-11-dioxa-10-phosphatricyclo-[10.4.0.0^{3,8}]hexadeca-1(16),3,5,7,12,14-hexaen-10-yl]palladium

[(A,S,S)/(C,R,S)-**L**$_{\mathbf{BB1}}$PdCl$_2$]:

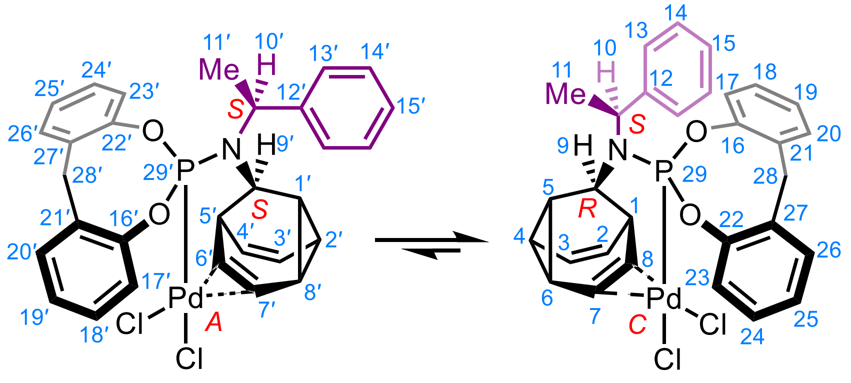

L_{BB1} (10.0 mg, 21.5 μmol) and $PdCl_2·(MeCN)_2$ (5.6 mg, 21.5 μmol) were suspended in $CDCl_3$ (0.7 mL) at rt and sonicated for 15 min until a homogeneous solution was formed. The solution was evaporated under reduced pressure to give the title compound as a pale yellow solid (13.5 mg, 0.02 mmol, 98%). Where possible, the ^{1}H and ^{13}C NMR resonances of the title compound have been assigned to a site in the structure of a specific diastereoisomer based on two-dimensional spectra acquired in the slow exchange regime (at 240 K). Unambiguous peak assignments are not possible in some cases on account of signal overlap. The number of nuclei represented by each signal is given without adjustment for the uneven population of the two diastereoisomers, which are present in a 1.3:1 ratio of (*C*,*R*,*S*)-**L_{BB}**$PdCl_2$ to (*A*,*S*,*S*)-**L_{BB1}**$PdCl_2$ at 240 K. **^{1}H NMR** (500 MHz, $CDCl_3$, 240 K) δ 7.53 (d, *J* = 7.6 Hz, 2H), 7.46 – 7.27 (m, 16H), 7.23 – 7.06 (m, 8H), 6.81 – 6.77 (m, 1H, H_7), 6.77 – 6.73 (m, 1H, $H_{7'}$), 6.10 (dd, *J* = 7.3, 4.6 Hz, 1H, $H_{6'}$), 6.04 – 5.96 (m, 2H, H_8 and $H_{4'}$), 5.90 – 5.82 (m, 2H, H_3 and $H_{3'}$), 5.74 (t, *J* = 7.9 Hz, 1H, H_2), 5.64 (q, *J* = 6.8 Hz, 1H, H_{10}), 5.58 (q, *J* = 6.9 Hz, 1H, $H_{10'}$), 4.43 (dd, *J* = 13.2, 3.4 Hz, 1H, $H_{28'}$), 4.30 (dd, *J* = 13.2, 3.3 Hz, 1H, H_{28}), 3.65 (d, *J* = 13.2 Hz, 1H, $H_{28'}$), 3.61 (d, *J* = 13.2 Hz, 1H, H_{28}), 3.12 – 3.05 (m, 2H, H_6 and $H_{5'}$), 3.02 – 2.95 (m, 2H, H_5 and H_4), 2.94 – 2.82 (m, 2H, H_9 and $H_{8'}$), 2.73 – 2.64 (m, 1H, $H_{2'}$), 2.57 – 2.40 (m, 2H, H_1 and $H_{9'}$), 1.73 (d, *J* = 7.0 Hz, 3H, H_{11}), 1.69 – 1.64 (m, 1H, $H_{1'}$), 1.61 (d, *J* = 6.8 Hz, 3H, $H_{11'}$). **^{13}C NMR** (151 MHz, $CDCl_3$, 240 K) δ 151.3, 151.2, 151.1, 148.5, 148.4, 139.0 (d, J_{CP} = 5.1 Hz, C_{12}), 138.9 ($C_{12'}$), 131.0, 131.0, 130.1, 130.0, 129.4, 129.3, 128.8, 128.7, 128.6, 128.3, 128.2, 127.7, 126.6, 126.1, 125.9, 125.7 ($C_{2'}$), 125.7, 125.1, 123.6, 123.0, 122.6, 108.0 ($C_{7'}$), 107.7 (C_7), 88.8 (C_6), 88.5 ($C_{8'}$), 55.6 (d, J_{CP} = 5.4 Hz, $C_{10'}$), 54.5 (d, J_{CP} = 5.8 Hz, C_{10}), 45.0, 44.9, 44.7, 44.6, 36.4, 35.2, 33.6 ($C_{28'}$), 33.5 (C_{28}), 33.4, 33.3, 29.5, 29.5, 26.8, 25.6, 18.3 ($C_{11'}$), 18.1 (C_{11}). The spectral data acquired at 298 K are also given below. At this temperature, the diastereoisomers are in fast exchange and most resonances are resolved, representing an average of the two structures. They are assigned using

the labelling system shown for (*C*,*R*,*S*)-**L_{BB1}**$PdCl_2$. Some peaks are broadened into the baseline at this temperature, so they are either missing or are reported as broad (br). **^{1}H NMR** (499 MHz, $CDCl_3$, 298 K) δ 7.48 (d, *J* = 7.6 Hz, 2H, H_{13}), 7.40 (t, *J* = 7.5 Hz, 2H, H_{14}), 7.36 – 7.33 (m, 1H, H_{15}), 7.32 (d, *J* = 7.4 Hz, 2H, H_{20} and H_{26}), 7.28 – 7.17 (m, 4H, H_{17}, H_{18}, H_{23} and H_{24}), 7.13 (t, *J* = 6.3 Hz, 2H, H_{19} and H_{25}), 6.79 (t, *J* = 6.5 Hz, 1H, H_7), 6.34 – 5.44 (br, 2H), 5.86 (t, *J* = 7.6 Hz, 1H, H_3), 5.67 – 5.58 (m, 1H, H_{10}), 4.37 (s, 1H, H_{28}), 3.64 (d, *J* = 13.3 Hz, 1H, H_{28}), 3.36 – 2.19 (br, 4H), 2.96 (d, J_{HP} = 31.6 Hz, 1H, H_9), 1.75 – 1.65 (m, 3H, H_{11}). **^{13}C NMR** (151 MHz, $CDCl_3$, 298 K) δ 139.4 (d, J_{CP} = 3.6 Hz, C_{12}), 130.2 (C_{20} and C_{26}), 128.9 (C_{14}), 128.6 (br), 128.6 (br), 128.3 (C_{15}), 128.1 (br, C_{13}), 126.3 (br, C_{19} and C_{25}), 125.4 (br, C_3), 123.4 (br, C_{17}, C_{18}, C_{23} and C_{24}), 107.8 (C_7), 55.2 (C_{10}), 45.1 (d, J_{CP} = 15.1 Hz, C_9), 33.9 (C_{28}), 18.2 (C_{11}). **^{31}P NMR** (243 MHz, $CDCl_3$, 298 K) δ 72.4 (P_{29}). **LR-ESI-MS** *m*/*z* = 642.307 $[M+H]^+$ (calculated for $C_{30}H_{29}Cl_2NO_2PPd^+$ = 642.0348).

Note on the stereochemical assignment of (*A*,*S*,*S*)/(*C*,*R*,*S*)-$L_{BB1}PdCl_2$:

The complex has been treated as a distorted trigonal bipyramidal complex (see Figure 4 in the manuscript and the X-ray Crystallographic Analysis Section 8, Figures S89–S91) for the assignment of stereochemical descriptor to the Pd centre, bearing apical P and Cl ligands, with equatorial Cl and C sites.[10] Although olefin ligands are typically regarded as a monodentate in organometallic nomenclature, it is necessary to treat the coordinated olefin of **L_{BB}** as an η^2-donor to account for its dissymmetry and accurately describe the stereochemical environment of the Pd centre. Consequently, the Pd centre is treated as being formally pentacoordinate. Treating the complex as being square planar and bearing a monodentate ligand would not capture the three-dimensional stereochemistry of the coordination as the olefin ligand is held in an orthogonal orientation to the Cl-Pd-Cl plane. Comparing the theoretical structures Pd1 and Pd2 (Figure S2) illustrates that, even

in the absence of a fixed stereocentre as part of the olefin ligand, the Pd coordination environment is chiral. The Cahn–Ingold–Prelog (CIP) priority of the ligands for stereochemical assignment of the Pd is Cl>P>C6>C7. Its configuration index is *TBPY-5-12*. Note also that the stereochemical descriptor for some of the sp^3-C stereocentres of the ligand (e.g., the barbaralane 9-position) are changed from the ligand precursor as the Pd coordination alters the CIP priorities assigned to the barbaralane skeleton.

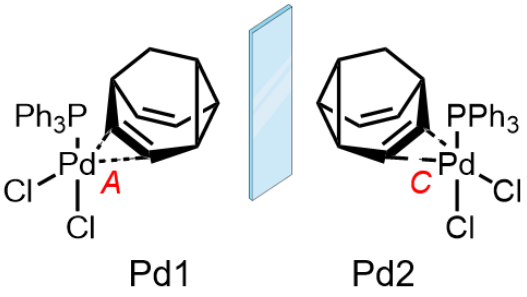

Figure S2. A theoretical enantiomeric pair of Pd complexes arising from the coordination of a dissymmetric olefin ligand (barbaralane) that is oriented orthogonally to the Cl-Pd-Cl plane.

***N*-[(1-Phenylethyl)({tricyclo[3.3.1.0^{2,8}]nona-2,6-dien-9-yl})ami-no]-19,20-dioxa-phospha-2-ru-thenpentacyco[9.8.2.0^{3,7}.0^{7,21}.0^{13,18}] henicosa-7(21),8,10,13,15,17-hexaen-2-yl}acetonitrile hexa-fluorophosphate** [(*C*,*R*,*S*)-**L$_{BB1}$**RuCp(NCMe)·PF_6]:

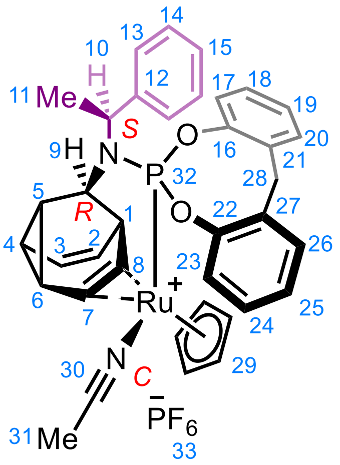

L_{BB1} (6.1 mg, 13.1 μmol) and CpRu(NCMe)$_3$·PF$_6$ (5.7 mg, 13.1 μmol) were suspended in $CDCl_3$ (0.7 mL) at rt and sonicated for 5 min until a homogeneous solution was formed. The resulting complex was isolated by slow diffusion of *i*Pr$_2$O vapour into the solution, which led to formation of a yellow precipitate. The mother liquor was decanted and the solid dried under reduced pressure. The resulting crude residue was then redissolved in $CDCl_3$ (0.7 mL). The solution was passed through a syringe tip filter then allowed to slowly evaporate to approximately one quarter of the volume, leading to the formation of crystals. The mother liquor was decanted and the solid dried under reduced pressure, affording the title compound as a yellow solid (7.4 mg, 9 μmol, 69%). The title complex is isolated as a single stereoisomer upon crystallisation but equilibrates over a period of several minutes-to-hours at room temperature in $CDCl_3$ solution, giving an approximately 4:1 mixture of the (*C*,*R*,*S*)- to the (*A*,*S*,*S*)-complex. NMR spectra for characterisation were obtained using a freshly crystallised sample and measured immediately after dissolution to allow assignment of the (*C*,*R*,*S*)-complex in its near-isomerically-pure form. Where possible, the ^{1}H and ^{13}C NMR resonances of the title compound have been assigned to a site in the structure based on two-dimensional spectra. Unambiguous peak assignments are not possible in some cases on account of signal overlap in the aromatic regions. **M. P.** 214 – 216 °C. **^{1}H NMR** (400 MHz, $CDCl_3$) δ 7.62 – 7.49 (m, 3H, H_{13-15} and/or H_{18-20} and/or H_{25-26}), 7.41 – 7.21 (m, 8H, H_{13-15} and/or H_{18-20} and/or H_{25-26}), 6.99 (d, J = 8.1 Hz, 1H, H_{23}), 6.88 (d, J = 6.9 Hz, 1H, H_{17}), 6.12 (t, J = 7.9 Hz, 1H, H_2), 5.99 (dd, J = 9.0, 6.2 Hz, 1H, H_3), 5.84 – 5.73 (m, 1H, H_{10}), 4.82 (dd, J = 8.0, 4.4 Hz, 1H, H_7), 4.62 (dd, J = 12.9, 3.7 Hz, 1H, H_{28}), 4.16 (s, 5H, H_{29}), 4.01 – 3.94 (m, 1H, H_8), 3.68 (d, J = 13.0 Hz, 1H, H_{28}), 3.49 – 3.40 (m, 1H, H_1), 3.19 (d, J = 22.0 Hz, 1H, H_9), 2.56 (d, J = 1.4 Hz, 3H, H_{31}), 2.09 – 1.98 (m, 1H, H_4), 1.93 – 1.84 (m, 1H, H_6), 1.77 (d, J = 7.0 Hz, 3H, H_{11}), 0.33 – 0.24 (m, 1H, H_5). **^{13}C NMR** (151 MHz, $CDCl_3$) δ 149.3, 141.6 (d, J_{CP} = 7.3 Hz, C_{12}), 135.0, 133.9, 130.8, 129.7 (C_{30}), 128.7,

128.5, 128.3, 128.1, 128.0, 128.0, 127.0, 126.5, 126.3, 124.7 (C_{23}), 123.3 (C_{17}), 84.7 (d, J_{CP} = 2.1 Hz, C_{29}), 52.9 (d, J_{CP} = 6.7 Hz, C_{10}), 51.0 (C_7), 47.4 (d, J = 15.7 Hz, C_9), 46.9 (C_8), 35.5 (d, J = 19.1 Hz, C_1), 34.0 (C_{28}), 25.0 (C_6), 24.9 (C_4), 19.1 (C_5), 16.2 (C_{11}), 4.8 (C_{31}). **^{19}F NMR** (376 MHz, $CDCl_3$) δ –73.0 (d, J_{PF} = 712.3 Hz, F_{33}). **^{31}P NMR** (162 MHz, $CDCl_3$) δ 162.8 (d, J_{PH} = 20.6 Hz, P_{32}), –144.3 (hept, J_{PF} = 712.3 Hz, P_{33}). **HR-ESI-MS** *m*/*z* = 667.1605 $[M-PF_6]^+$ (calculated for $C_{37}H_{36}N_2O_2P^{96}Ru^+$ = 667.1590).

Note on the stereochemical assignment of (*C*,*R*,*S*)-L_{BB1}RuCp(NCMe)·PF_6:

The complex has been treated as a distorted square pyramidal complex (see Figure 5 in the manuscript and the X-ray Crystallographic Analysis Section 8, Figures S92 and S93) for the assignment of stereochemical descriptor to the Ru center, bearing an apical Cp ligand, with the remaining N, P, and two C coordination sites close to being in a plane.[10] The CIP priority of the ligands for stereochemical assignment of the Ru is Cp>P>N>C8>C7. Note that Cp (μ_5-$C_5H_5^-$) is treated as a pseudo-atom of atomic number 30 and mass number 60 according to convention for arene-type polyhapto ligands.[11] The configuration index of the (*C*,*R*,*S*)- and (*A*,*R*,*S*)-complexes are *SPY-5-25*, whereas the (*C*,*S*,*S*)- and (*A*,*S*,*S*)-complexes are *SPY-5-24*. Note also that the stereochemical descriptor for some of the sp^3-C stereocentres of the ligand (e.g., the barbaralane 9-position) are changed from the ligand precursor as the Ru coordination alters the CIP priorities assigned to the barbaralane skeleton.

***N*-1-(Phenylmethyl)-tricyclo[3.3.1.0^{2,8}]-nona-3,6-dien-9-amine** [(*R*)/(*S*)-**S3**]:

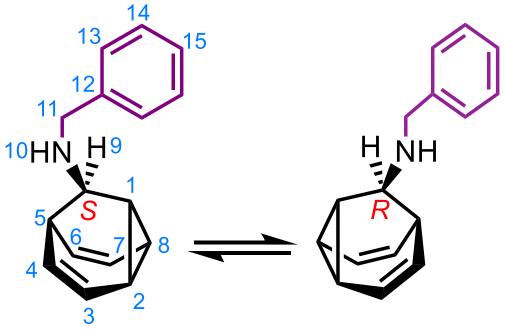

In an oven-dried round-bottomed flask fitted with a septum under a N_2 atmosphere, tricyclo[3.3.1.0^{2,8}]nona-3,6-dien-9-one [**3**] (50 mg, 0.38 mmol) and benzylamine (130 µL, 1.22 mmol) were dissolved in anhydrous MeOH (0.50 mL). Glacial acetic acid (16 µL) was added dropwise to the stirred reaction mixture and left for 30 min. Sodium cyanoborohydride (29 mg, 0.46 mmol, 1.2 equiv.) was then added to the reaction mixture, which was left to stir for 4 d at rt. The mixture was quenched with 5 drops of Et_3N and the solvent was removed under reduced pressure. This material was dissolved in a saturated aqueous solution of $NaHCO_3$ (50 mL) and extracted with CH_2Cl_2 (5 × 25 mL). The combined organic extracts were washed with brine (1 × 50 mL), H_2O (1 × 50 mL), dried over $MgSO_4$, filtered and the solvent was removed under reduced pressure. The crude residue was purified by column chromatography (Teledyne Isco CombiFlash Rf+ system, 12 g SiO_2, hexanes–EtOAc, gradient including 1% Et_3N in the elution) to give the title compound as a colourless oil (61 mg, 0.26 mmol, 69%) containing ~15% of the starting material benzylamine, which proved to be inseparable from (*R*)/(*S*)-**S3**. **^{1}H NMR** (600 MHz, $CDCl_3$) δ 7.33 – 7.26 (m, 4H, H_{13} and H_{14}), 7.26 – 7.14 (m, 1H, H_{15}), 5.76 (td, J = 7.9, 1.2 Hz, 1H, H_7), 5.54 (td, J = 7.7, 1.2 Hz, 1H, H_3), 4.07 – 3.99 (m, 2H, H_2 and H_4), 3.92 – 3.84 (m, 2H, H_6 and H_8), 3.70 (d, J = 1.2 Hz, 2H, H_{11}), 2.57 – 2.51 (m, 2H, H_1 and H_5), 2.47 – 2.44 (m, 1H, H_9), 1.17 (br s, 1H, H_{10}). **^{13}C NMR** (151 MHz, $CDCl_3$) δ 140.8 (C_{12}), 128.5 (C_{13} or C_{14}), 128.1 (C_{13} or C_{14}), 126.9 (C_{15}), 123.0 (C_7), 121.4 (C_3), 76.1 (C_2 and C_4), 72.3 (C_6 and C_8), 53.3 (C_9), 50.1 (C_{11}), 29.3 (C_1 and C_5). **HRMS-ASAP** m/z = 224.1412 $[M+H]^+$, calculated for $C_{16}H_{18}N^+$: 224.1434.

***N*-(1-Phenylmethyl)-*N*-{tricyclo[3.3.1.0^{2,8}]nona-2,6-dien-9-yl}-9,11-dioxa-10-phosphatricyclo-[10. 4.0.0^{3,8}]hexadeca-1(16),3,5,7,12,14-hexaen-10-amine** [(*S*)/(*R*)-**L$_{BB2}$**]:

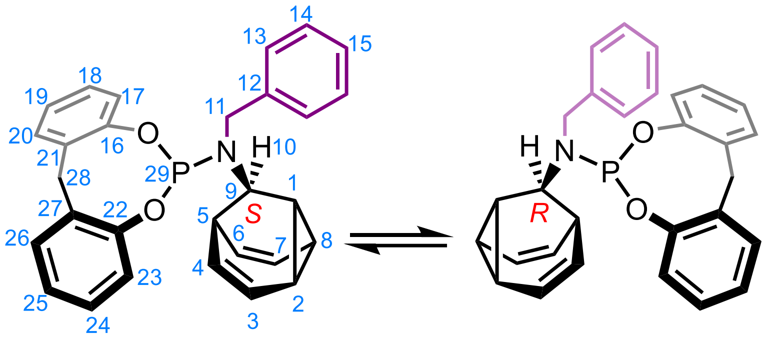

PCl_3 (135 mg, 0.98 mmol), Et_3N (794 mg, 7.84 mmol) and anhydrous CH_2Cl_2 (2.0 mL) were placed in an oven-dried vial under an N_2 atmosphere and cooled to 0 °C. A solution of *N*-1-(Phenylmethyl)-tricyclo[3.3.1.0^{2,8}]-nona-3,6-dien-9-amine [(*R*)/(*S*)-**S3**] (229 mg, 0.98 mmol) in anhydrous CH_2Cl_2 (2.0 mL) was added dropwise by syringe over 10 min and the resulting mixture stirred at 0 °C for 3 h. A solution of 2,2′-methylenediphenol (197 mg, 0.98 mmol) in anhydrous CH_2Cl_2 (2.0 mL) was added dropwise by syringe over 10 min, then the resulting mixture was allowed to warm to rt and stirred for 16 h. The solution was poured into a mixture of H_2O (30 mL) and brine (5 mL), then extracted with CH_2Cl_2 (4 × 10 mL). The combined organic extracts were dried over K_2CO_3 then the solvent was removed under reduced pressure. The crude residue was purified by column chromatography (Teledyne Isco CombiFlash Rf+ system, 8 g neutral Al_2O_3, hexanes−CH_2Cl_2, gradient elution), giving the title compound as a colourless solid powder (245 mg, 0.55 mmol, 56%). The ligand was stored in a vial flushed with nitrogen to prevent degradation. **M. P.** 100 – 102 °C. **^{1}H NMR** (600 MHz, $CDCl_3$) δ 7.50 (d, *J* = 7.6 Hz, 2H, H_{13}), 7.40 – 7.33 (m, 2H, H_{14}), 7.31 (dt, *J* = 7.6, 1.4 Hz, 2H, H_{20} and H_{26}), 7.29 – 7.24 (m, 1H, H_{15}), 7.21 – 7.12 (m, 2H, H_{19} and H_{25}), 7.07 (dd, *J* = 8.0, 1.3 Hz, 2H, H_{17} and H_{23}), 7.01 (t, *J* = 7.4 Hz, 2H, H_{18} and H_{24}), 5.91 (t, *J* = 7.6 Hz, 1H, H_7), 5.56 (t, *J* = 7.2 Hz, 1H, H_3), 4.53 (d, *J* = 2.4 Hz, 2H, H_{11}), 4.36 (dd, *J* = 12.8, 2.9 Hz, 1H, H_{28}), 4.08

(t, J = 7.1 Hz, 4H, H_2, H_4, H_6 and H_8), 3.52 (d, J = 12.9 Hz, 1H, H_{28}), 3.09 – 2.92 (m, 1H, H_{10}), 2.63 (td, J = 6.6, 2.4 Hz, 1H, H_1 and H_5). **^{13}C NMR** (151 MHz, $CDCl_3$) δ 151.8 (d, J_{CP} = 6.5 Hz, 2C, C_{16} and C_{22}), 139.8 (C_{12}), 135.6 (d, J_{CP} = 2.9 Hz, 2C, C_{21} and C_{27}), 129.9 (2C, C_{20} and C_{26}), 128.42 (d, J_{CP} = 4.4 Hz, 2C, C_{13} and C_{24}), 128.0 (2C, C_{19} and C_{25}), 127.0 (C_{15}), 124.42 (d, J_{CP} = 1.5 Hz, 2C, C_{18} and C_{24}), 123.8 (d, J_{CP} = 3.5 Hz, C_7), 123.1 (d, J_{CP} = 3.1 Hz, 2C, C_{17} and C_{23}), 121.9 (C_3), 76.4 (2C, C_2 and C_4), 74.0 (2C, C_6 and C_8), 48.1 (d, J_{CP} = 20.0, Hz, C_9), 47.5 (d, J_{CP} = 3.3, Hz, C_{11}), 34.2 (C_{28}), 28.9 (d, J_{CP} = 9.1 Hz, 2C, C_1 and C_5). **^{31}P NMR** (243 MHz, $CDCl_3$) δ 136.9 (P_{29}). **HRMS-ASAP** *m/z* = 452.1765 $[M+H]^+$, calculated for $C_{29}H_{27}NO_2P^+$: 452.1779.

***N*-[(1-Phenylmethyl)({tricyclo[3.3.1.0^{2,8}]nona-2,6-dien-9-yl})amino]-19,20-dioxa-phospha-2-ruthenpentacyco[9.8.2.0^{3,7}.0^{7,21}.0^{13,18}] henicosa-7(21),8,10,13,15,17-hexaen-2-yl}acetonitrile hexafluorophosphate** [(*A*,*S*)/(*C*,*R*)-**L$_{BB2}$**RuCp(NCMe)·PF_6]:

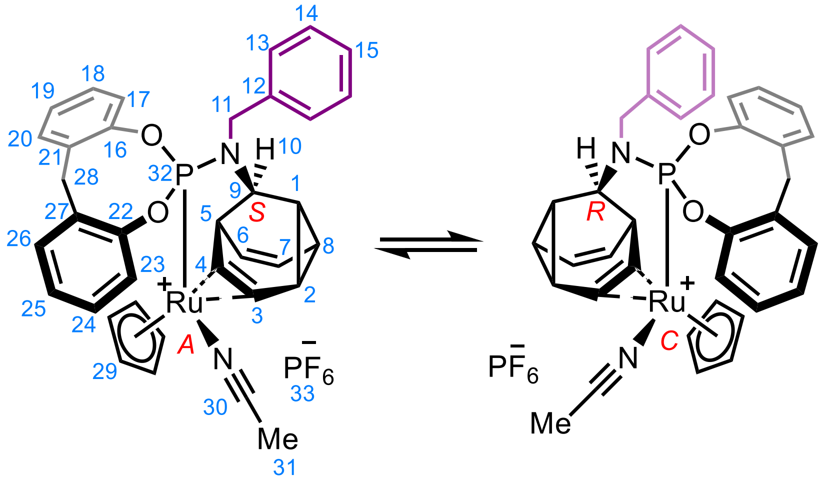

N-(1-Phenylmethyl)-*N*-{tricyclo[3.3.1.0^{2,8}]nona-2,6-dien-9-yl}-9,11-dioxa-10-phosphatricyclo-[10.4.0.0^{3,8}]hexadeca-1(16),3,5,7,12,14-hexaen-10-amine [(*S*)/(*R*)-**L$_{BB2}$**] (13 mg, 0.02 mmol) and $CpRu(NCMe)_3·PF_6$ (12.1 mg, 0.02 mmol) were suspended in $CHCl_3$ (1.5 mL) at rt and sonicated for 10 min until a homogeneous solution was formed. The resulting complex was isolated by slow

diffusion of $^{i}Pr_2O$ vapour into the solution, which led to formation of a yellow precipitate. The mother liquor was decanted and the solid dried under reduced pressure. The resulting crude residue was then redissolved in $CHCl_3$ (0.7 mL). The solution was passed through a syringe tip filter then allowed to slowly evaporate to approximately one quarter of the volume, leading to the formation of crystals over 6 d. The mother liquor was decanted and the solid dried under reduced pressure, affording the title compound as a yellow crystalline solid (16 mg, 19 µmol, 98%). **M. P.** 213 – 218 °C. **^{1}H NMR** (600 MHz, $CDCl_3$) δ 7.54 – 7.45 (m, 3H, H_{13-15} and/or H_{18-20} and/or H_{25-26}), 7.32 – 7.26 (m, 6H, H_{13-15} and/or H_{18-20} and/or H_{25-26}), 7.24 – 7.21 (m, 2H, H_{13-15} and/or H_{18-20} and/or H_{25-26}), 6.91 (d, *J* = 8.0 Hz, 1H, H_{17} or H_{23}), 6.89 – 6.85 (m, 1H, H_{17} or H_{23}), 6.13 – 6.04 (m, 1H, H_3 or H_4), 6.01 – 5.94 (m, 1H, H_3 or H_4), 5.15 – 5.04 (m, 1H, H_{11}), 4.91 – 4.81 (m, 1H, H_7), 4.52 (dd, *J* = 13.3, 3.4 Hz, 1H, H_{28}), 4.17 (s, 5H, H_{29}), 4.04 – 3.96 (m, 1H, H_{11}), 3.91 (d, *J* = 14.7 Hz, 1H, H_{28}), 3.68 (d, *J* = 13.3 Hz, 1H, H_6), 3.62 – 3.54 (m, 1H, H_5), 3.07 (d, *J* = 20.0 Hz, 1H, H_{10}), 2.49 (s, 3H, $_{31}$), 2.18 – 2.11 (m, 1H, H_2), 1.98 – 1.94 (m, 1H, H_8), 0.93 – 0.81 (m, 1H, H_1). As a result of the low solubility of the crystalline solid obtained, ^{13}C NMR and some two-dimensional spectroscopic data could not be acquired for this compound – therefore tentative assignment of the NMR resonances for this compound has been made by analogy to (*C*,*R*,*S*)-$\mathbf{L_{BB1}}$RuCp(NCMe)·PF_6. The identity of the compound was confirmed by ^{1}H, ^{19}F and ^{31}P NMR spectroscopy, single-crystal X-ray diffraction and HRMS. **^{19}F NMR** (376 MHz, $CDCl_3$) δ –72.9 (d, J_{PF} = 712.7 Hz, F_{33}). **^{31}P NMR** (243 MHz, CD_3CN) δ 162.0 (P_{32}), –146.1 (hept, J_{PF} = 720.2 Hz, P_{33}). **HRMS-ASAP** *m/z* = 618.1140 $[M-PF_6-MeCN]^+$, calculated $C_{34}H_{31}NO_2PRu^+$ for: 618.1136. The stereochemical assignment of (*A*,*S*)/(*C*,*R*)-$\mathbf{L_{BB2}}$RuCp(NCMe)·PF_6 has been made by analogy to (*C*,*R*,*S*)-$\mathbf{L_{BB1}}$RuCp(NCMe)·PF_6.

***N*-Benzylcyclopent-3-en-1-amine** [**S5**]:

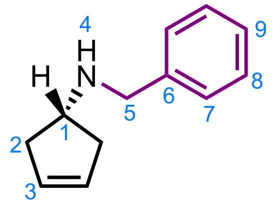

In an oven-dried round-bottomed flask fitted with a septum under a N_2 atmosphere, cyclopent-3-en-1-ammonium chloride (250 mg, 2.09 mmol) and benzylaldehyde (1.11 g, 10.5 mmol) were dissolved in anhydrous MeOH (2 mL). Glacial acetic acid (1 mL) was added dropwise to the stirred reaction mixture and left for 30 min. Sodium cyanoborohydride (394 mg, 6.27 mmol) was then added to the reaction mixture, which was left to stir for 36 h at rt. The mixture was quenched with three drops of Et_3N and the solvent was removed under reduced pressure. This material was dissolved in a saturated aqueous solution of $NaHCO_3$ (50 mL) and extracted with CH_2Cl_2 (5 × 25 mL). The combined organic extracts were washed with brine (1 × 50 mL), H_2O (1 × 50 mL), dried over $MgSO_4$, filtered and the solvent was removed under reduced pressure. The crude residue was purified by column chromatography (Teledyne Isco CombiFlash Rf+ system, 12 g SiO_2, hexanes-EtOAc) to give the title compound as a colourless oil (308.6 mg, 1.78 mmol, 85%). **^{1}H NMR** (600 MHz, $CDCl_3$) δ 7.38 – 7.30 (m, 4H, H_7 and H_8), 7.25 – 7.22 (m, 1H, H_9), 5.69 (s, 2H, H_3), 3.79 (s, 2H, H_5), 3.62 – 3.37 (m, 1H, H_1), 2.83 – 2.45 (m, 2H, H_2), 2.30 – 2.02 (m, 2H, H_2), 1.44 (s, 1H, H_4). **^{13}C NMR** (151 MHz, $CDCl_3$) δ 140.6 (C_6), 129.1 (C_3), 128.5 (C_7 or C_8), 128.4 (C_7 or C_8), 127.0 (C_9), 57.32 (C_1), 52.43 (C_5), 40.04 (C_2). **HRMS-ASAP** *m/z* = 173.1204 $[M+H]^+$, calculated $C_5H_{10}N^+$ for: 174.1282.

***N*-Benzyl-*N*-(cyclopent-3-en-1-yl)-dibenzodioxa-phospha-6-amine** [**L_{CP}**]:

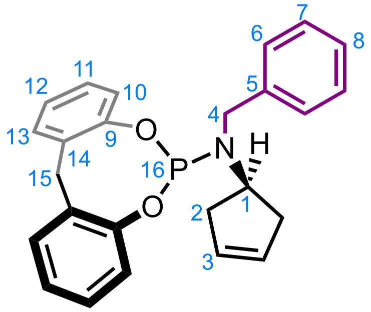

PCl_3 (79 mg, 0.58 mmol), Et_3N (467 mg, 4.6 mmol) and anhydrous CH_2Cl_2 (2.0 mL) were placed in an oven-dried vial under an N_2 atmosphere and cooled to 0 °C. A solution of *N*-benzylcyclopent-3-en-1-amine [**S5**] (100 mg, 0.58 mmol) in anhydrous CH_2Cl_2 (2.0 mL)

was added dropwise by syringe over 10 min and the resulting mixture stirred at 0 °C for 3 h. A solution of 2,2′-methylenediphenol (116 mg, 0.58 mmol) in anhydrous CH_2Cl_2 (2.0 mL) was added dropwise by syringe over 10 min, then the resulting mixture was allowed to warm to rt and stirred for 16 h. The solution was poured into a mixture of H_2O (30 mL) and brine (5 mL), then extracted with CH_2Cl_2 (4 × 10 mL). The combined organic extracts were dried over K_2CO_3 then the solvent was removed under reduced pressure. The crude residue was purified by column chromatography (Teledyne Isco CombiFlash Rf+ system, 12 g neutral Al_2O_3, hexanes−CH_2Cl_2, gradient elution), giving the title compound as a colourless oil which solidified upon standings (47 mg, 0.12 mmol, 20%). The ligand was stored in a vial flushed with nitrogen to prevent degradation. **M. P.** 74 – 76 °C. **^{1}H NMR** (600 MHz, $CDCl_3$) δ 7.55 – 7.46 (m, 2H, H_6), 7.37 (dd, J = 8.4, 6.9 Hz, 2H, H_7), 7.32 (dd, J = 7.5, 1.7 Hz, 2H, H_{13}), 7.31 – 7.26 (m, 1H, H_8), 7.15 (td, J = 7.7, 1.7 Hz, 2H, H_{10}), 7.05 – 7.02 (m, 2H, H_{12}), 7.02 – 6.99 (m, 2H, H_{11}), 5.76 (s, 2H, H_3), 4.54 (d, J = 6.0 Hz, 2H, H_4), 4.37 (dd, J = 12.9, 2.9 Hz, 1H, H_{17}), 4.21 (dddd, J = 15.6, 7.9, 5.0, 2.9 Hz, 1H, H_1), 3.57 (d, J = 12.9 Hz, 1H, H_{17}), 2.77 – 2.61 (m, 2H, H_2), 2.61 – 2.49 (m, 2H, H_2). **^{13}C NMR** (151 MHz, $CDCl_3$) δ 151.6 (d, J_{CP} = 5.5 Hz, 2C, C_9), 140.6 (d, J_{CP} = 2.3 Hz, C_5), 135.4 (d, J_{CP} = 2.9 Hz, 2C, C_{14}), 129.9 (d, J_{CP} = 1.3 Hz, 2C, C_{13}), 129.7 (d, J_{CP} = 1.3 Hz, 2C, C_3), 128.4 (C_7), 128.2 (C_6), 128.1 (d, J_{CP} = 1.4 Hz, 2C, C_{12}) 126.9 (C_8), 124.6 (d, J_{CP} = 1.5 Hz, 2C, C_{10}),123.0 (d, J_{CP} = 3.1 Hz, 2C, C_{11}), 56.6 (d, J_{CP} = 3.1 Hz, C_1), 47.1 (d, J_{CP} = 5.4 Hz, C_4), 38.5 (d, J_{CP} = 10.9 Hz, C_2), 34.2 (C_{15}). **^{31}P NMR** (243 MHz, $CDCl_3$) δ 139.0 (P_{16}). **HRMS-ASAP** *m/z* = 402.1612 $[M+H]^+$, calculated $C_{25}H_{25}NO_2P^+$ for: 401.1545.

General Procedure for the iridium-catalysed asymmetric allylic substitution to form 4-hydroxy-3-(1-(naphthalen-2-yl)allyl)-2*H*-chromen-2-one [**9**]:

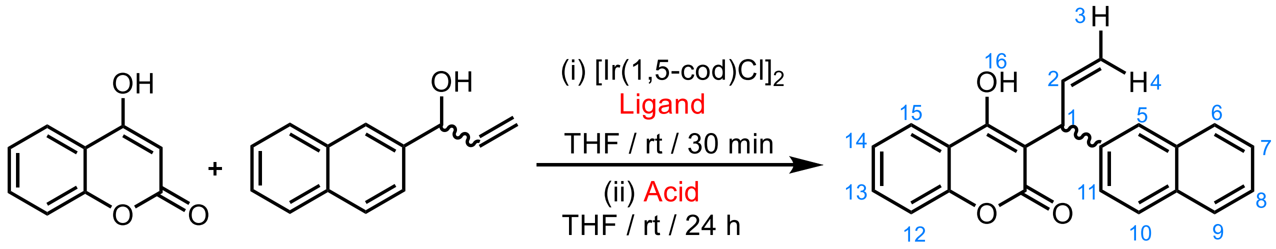

To an oven-dried microwave vial charged with a stirrer bar, was added [Ir(1,5-cod)Cl]$_2$ (1.7 mg, 2.5 µmol, 4 mol%) and the desired ligand (16 mol%). The microwave vial was sealed and mixture was subjected to 3 × purge-and-refill cycles (vacuum followed by N_2). To this microwave vial was added anhydrous and deoxygenated (3 × freeze–pump–thaw cycles under N_2) THF (0.2 mL). The mixture was left to stir at rt for 30 min. In a separate oven-dried microwave vial charged with a stirrer bar was added (±)-1-(naphthalen-2-yl)prop-2-en-1-ol [**10**] (22.7 mg, 0.123 mmol, 2 equiv.), 4-hydroxycoumarin (10 mg, 61.6 µmol, 1 equiv.) and the desired acid (10 mol%). The microwave vial was sealed and mixture was subjected to 3 × purge-and-refill cycles (vacuum followed by N_2). To this microwave vial was added anhydrous and deoxygenated (3 × freeze–pump–thaw cycles under N_2) THF (0.26 mL). This reagent solution was then added to the microwave vial containing the [Ir(1,5-cod)Cl]$_2$/ligand solution. The reaction mixture was left to stir at rt for 24 h before concentrating under reduced pressure. The crude residue was purified by column chromatography (Teledyne Isco CombiFlash Rf+ system, 4 g SiO_2, hexanes–EtOAc,) to give the title compound as a colourless oil. **^{1}H NMR** (400 MHz, $CDCl_3$) δ 7.85 (d, J = 8.5 Hz, 1H), 7.83 (d, J = 3.2 Hz, 1H), 7.80 (d, J = 2.1 Hz, 1H), 7.77 (dd, J = 8.0, 1.6 Hz, 1H), 7.56 (ddd, J = 8.6, 7.3, 1.6 Hz, 1H), 7.53 – 7.47 (m, 2H), 7.44 (dd, J = 8.5, 1.9 Hz, 1H), 7.34 (dd, J = 8.4, 1.1 Hz, 1H), 7.31 – 7.27 (m, 1H), 7.01 (s, 1H, H_5), 6.54 (ddd, J = 17.4, 10.3, 5.4 Hz, 1H, H_2), 5.55 (ddd, J = 10.3, 1.9, 1.1 Hz, 1H, H_3),

5.50 – 5.46 (m, 1H, H_1), 5.25 (ddd, J = 17.4, 2.1, 1.2 Hz, 1H, H_4). The enantiomeric ratio of the scalemic mixture was determined by chiral HPLC (ChiralPak AD column, isocratic elution with 9:1 hexanes–^{i}PrOH, including 0.1% TFA in the mobile phase, 254 nm detection).

Spectroscopic data were consistent with those published previously.[12]

Table S1. Comparison of the ligands, acids, yields and enantiomeric ratios for the iridium-catalysed asymmetric allylic substitution to form **9**.

Entry	Ligand	Acid	Yield / %	Enantiomeric Ratio
1	(±)-**CL**	(±)-BDHP	32	50:50
2	(±)-**CL**	(*R*)-(−)BDHP	14	51:49
3	$\mathbf{L_{BB2}}$	(*R*)-(−)BDHP	36	65:35
4	$\mathbf{L_{CP}}$	(*R*)-(−)BDHP	49	51:49

3. ^{1}H, ^{13}C, ^{19}F and ^{31}P NMR Spectroscopic Characterisation of Synthesised Compounds

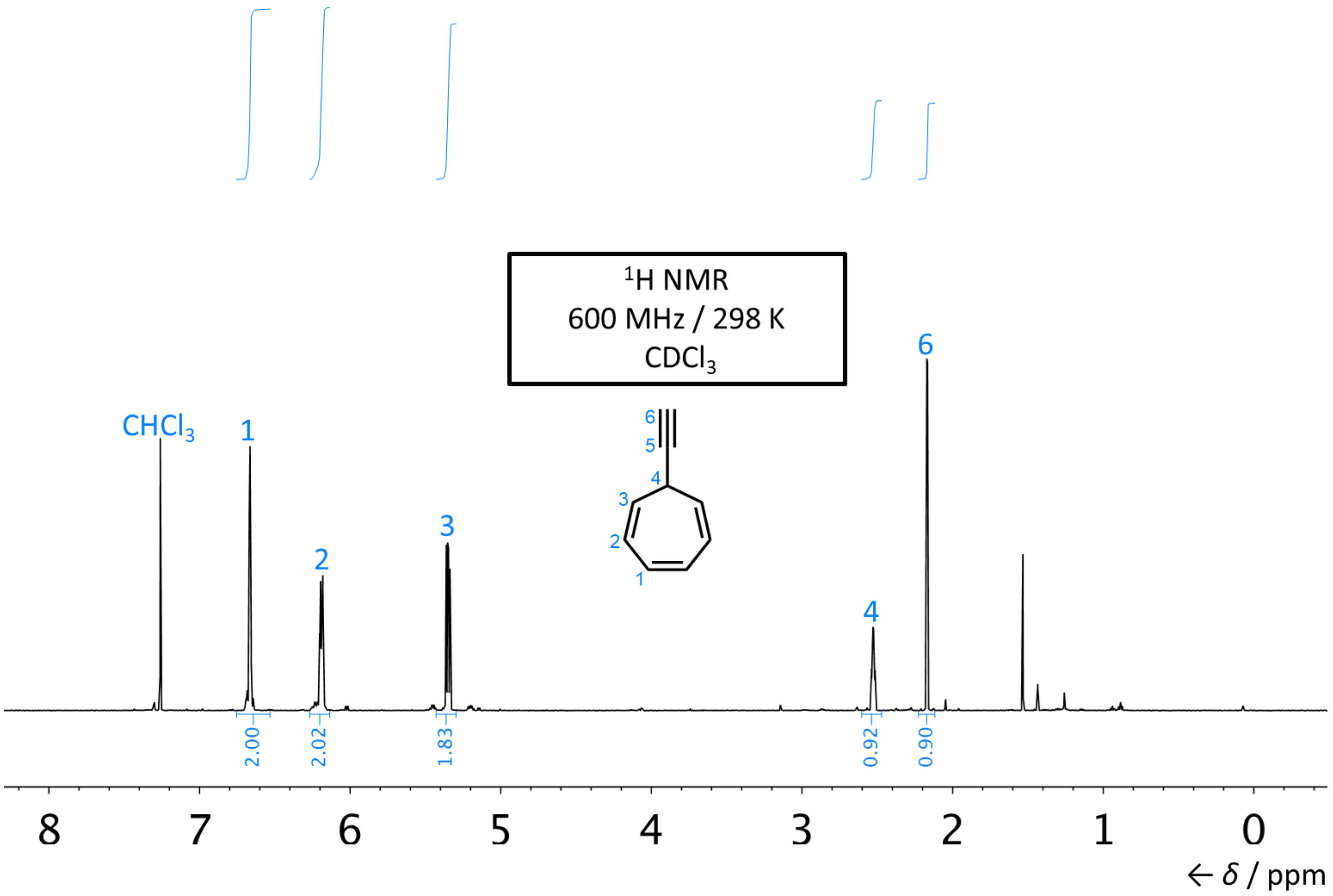

Figure S3. ^{1}H NMR spectrum of **S2**.

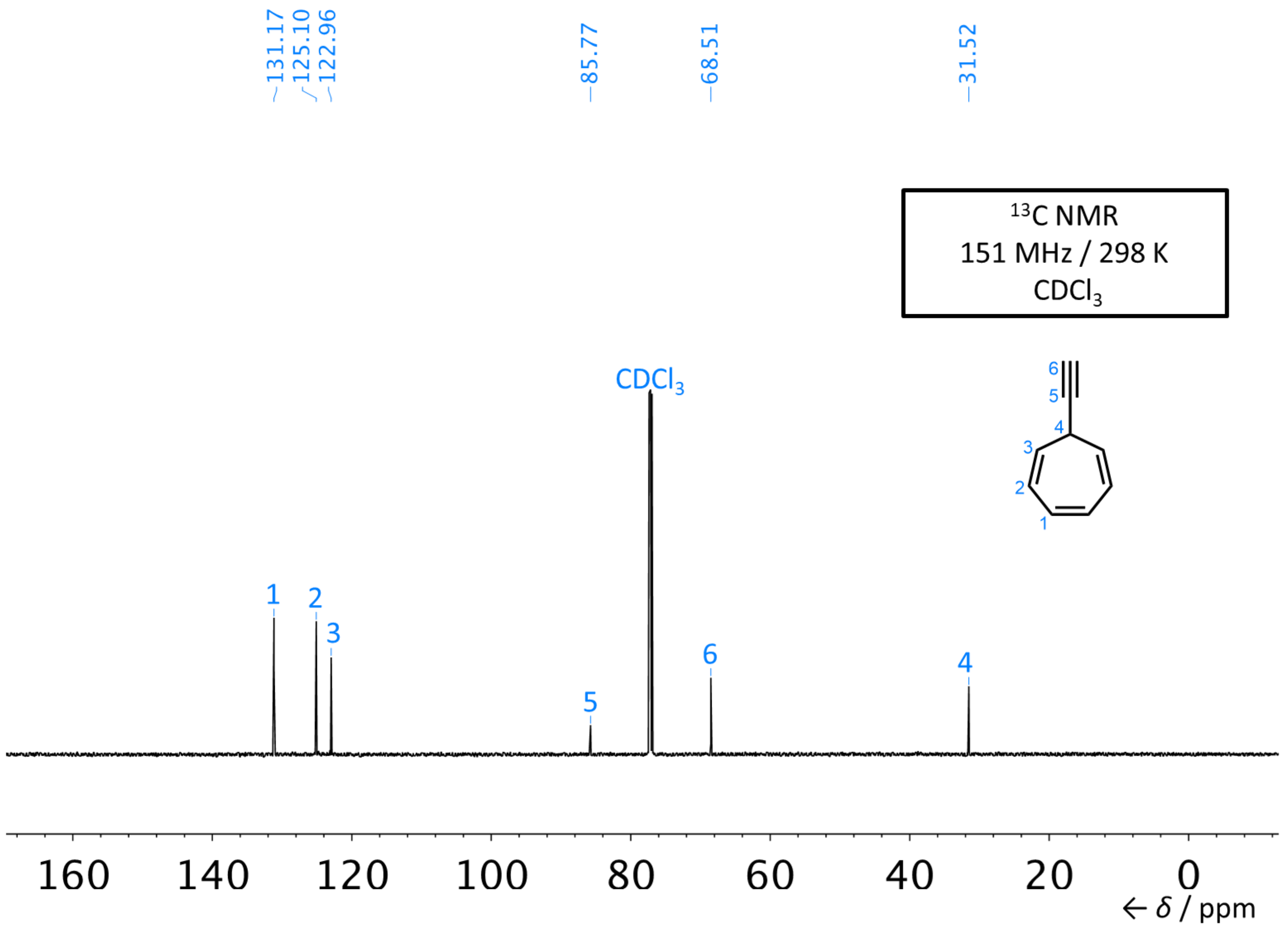

Figure S4. ^{13}C NMR spectrum of **S2**.

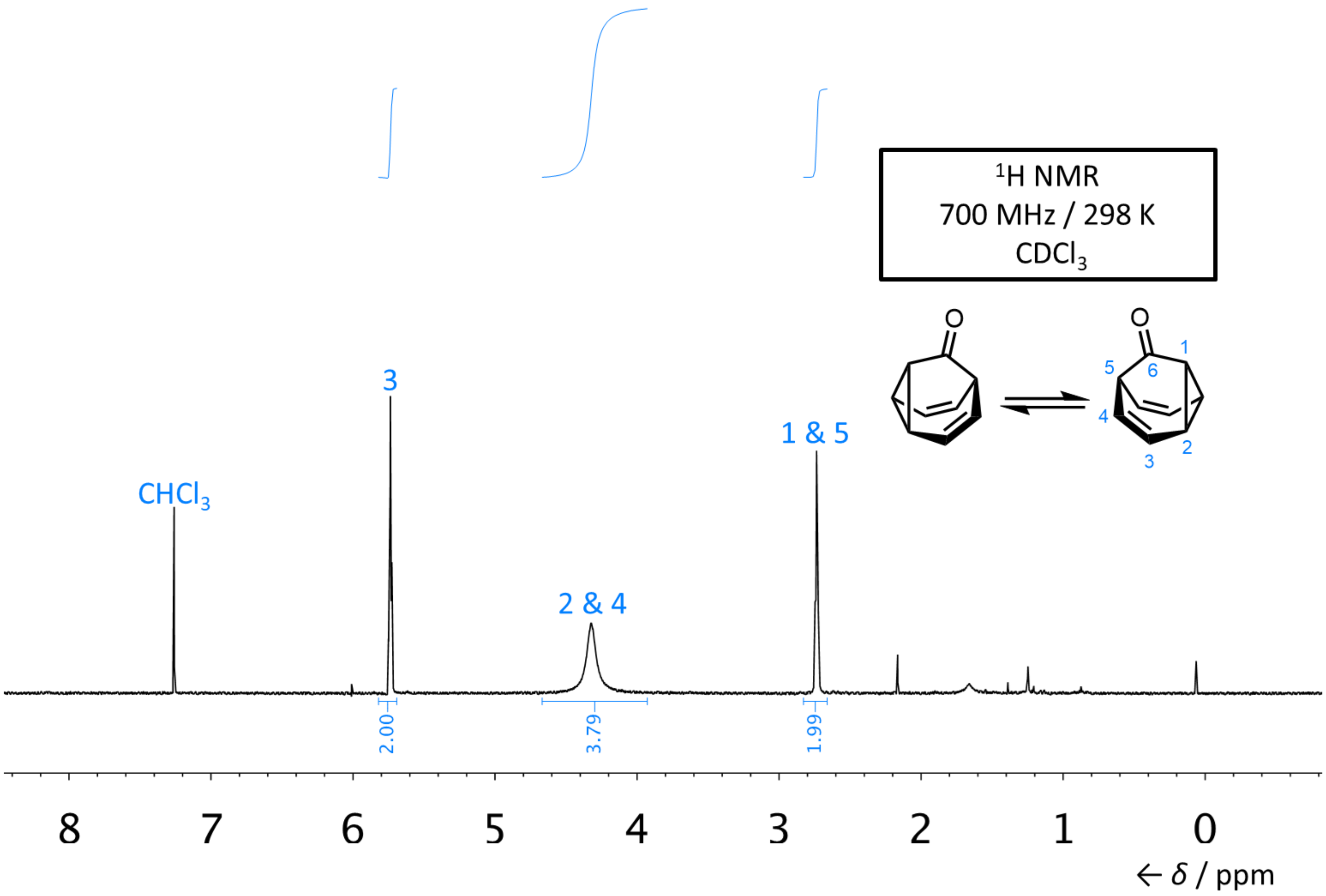

Figure S5. ^{1}H NMR spectrum of **3**.

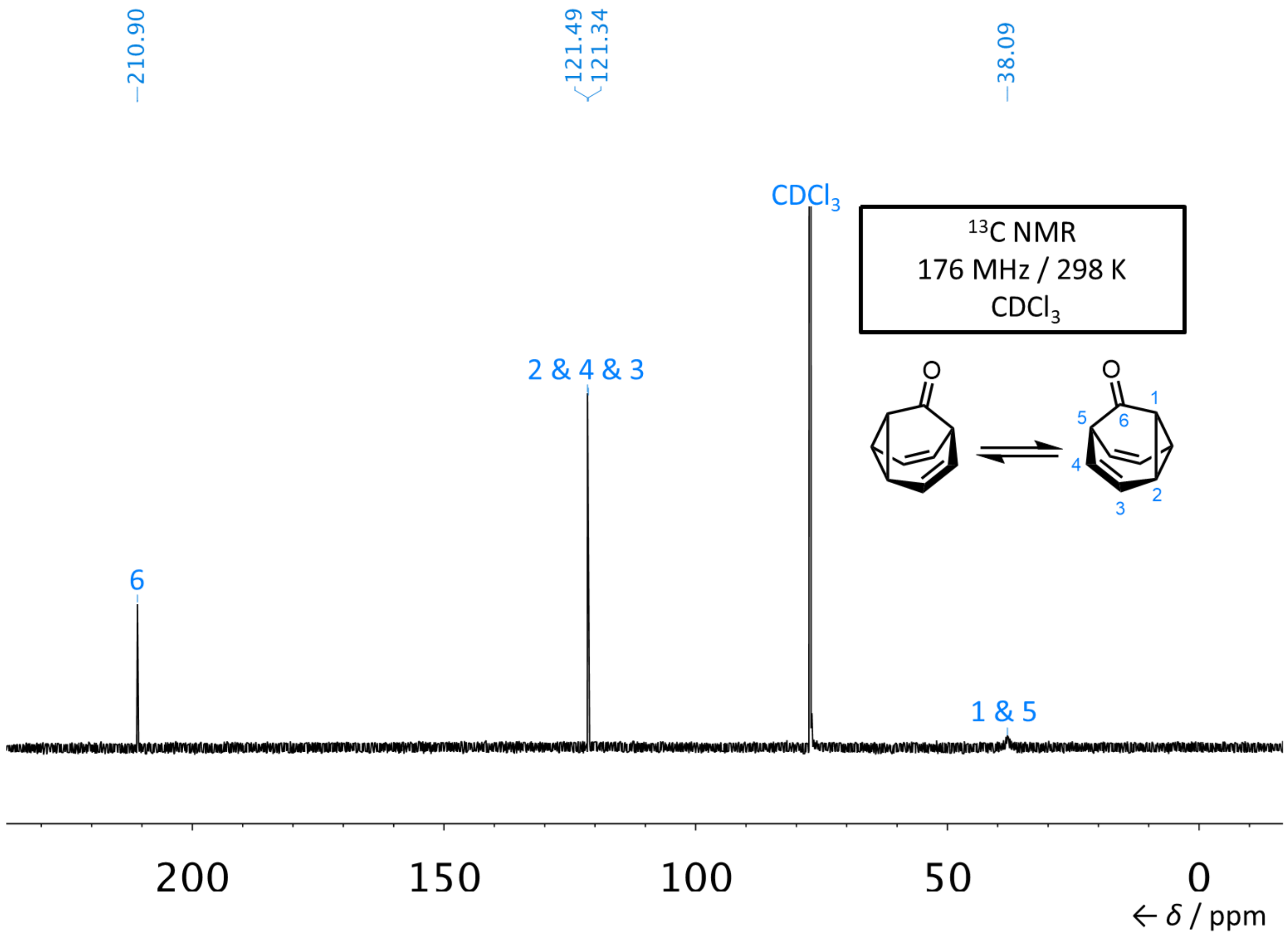

Figure S6. ^{13}C NMR spectrum of **3**.

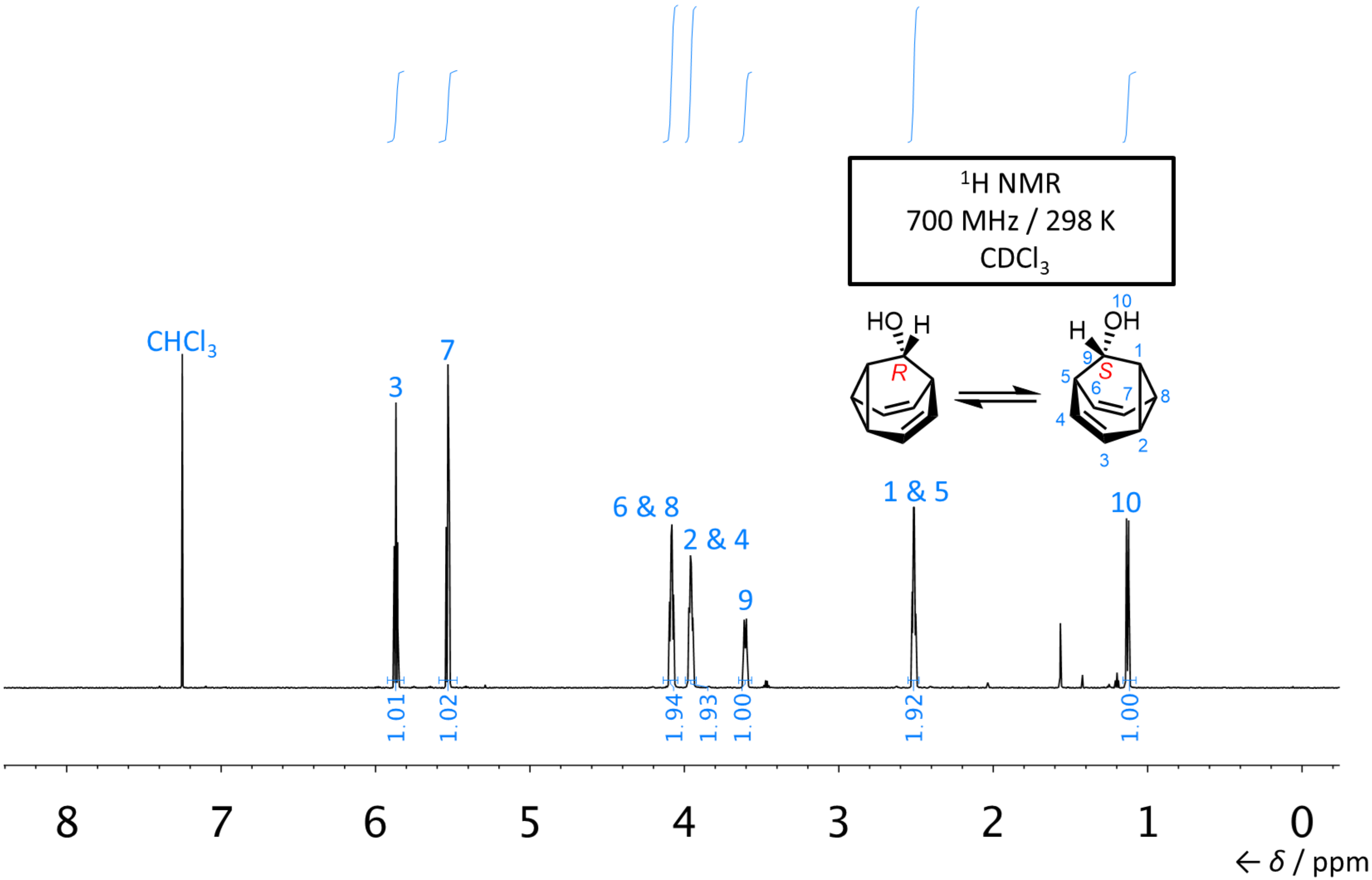

Figure S7. ^{1}H NMR spectrum of (*R*)/(*S*)-**1**.

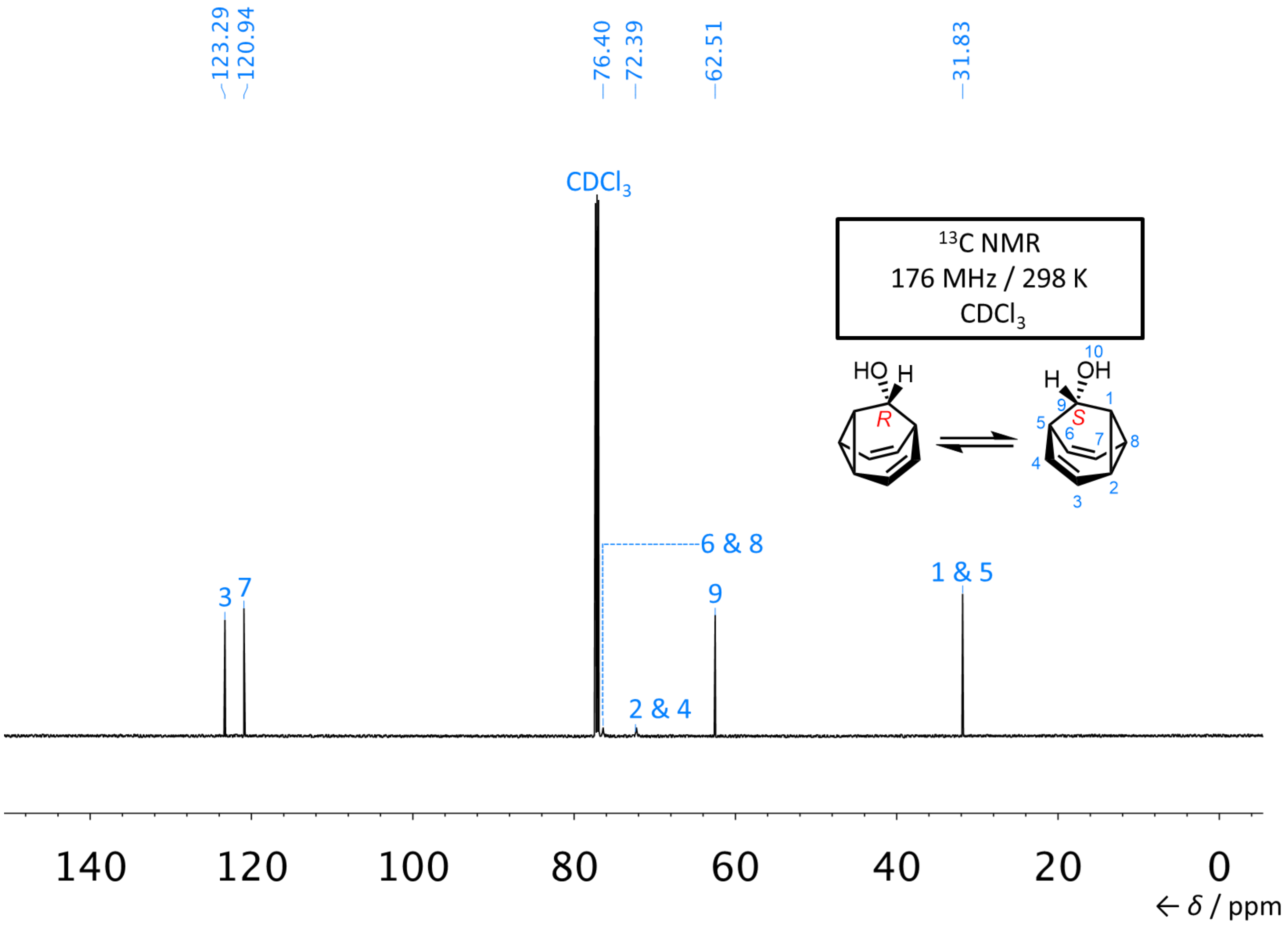

Figure S8. ^{13}C NMR spectrum of (*R*)/(*S*)-**1**.

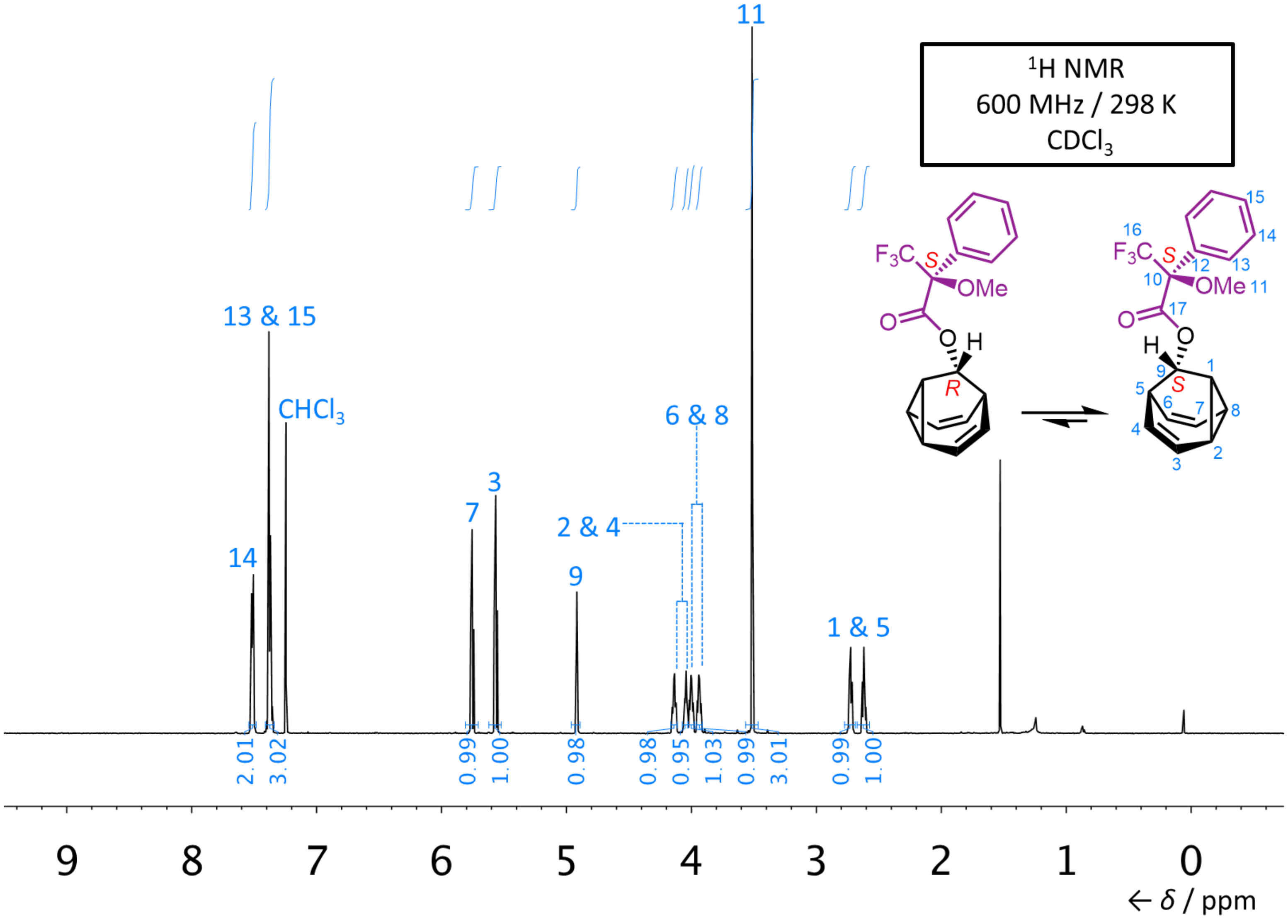

Figure S9. ^{1}H NMR spectrum of (*R*,*S*)/(*S*,*S*)-**2**.

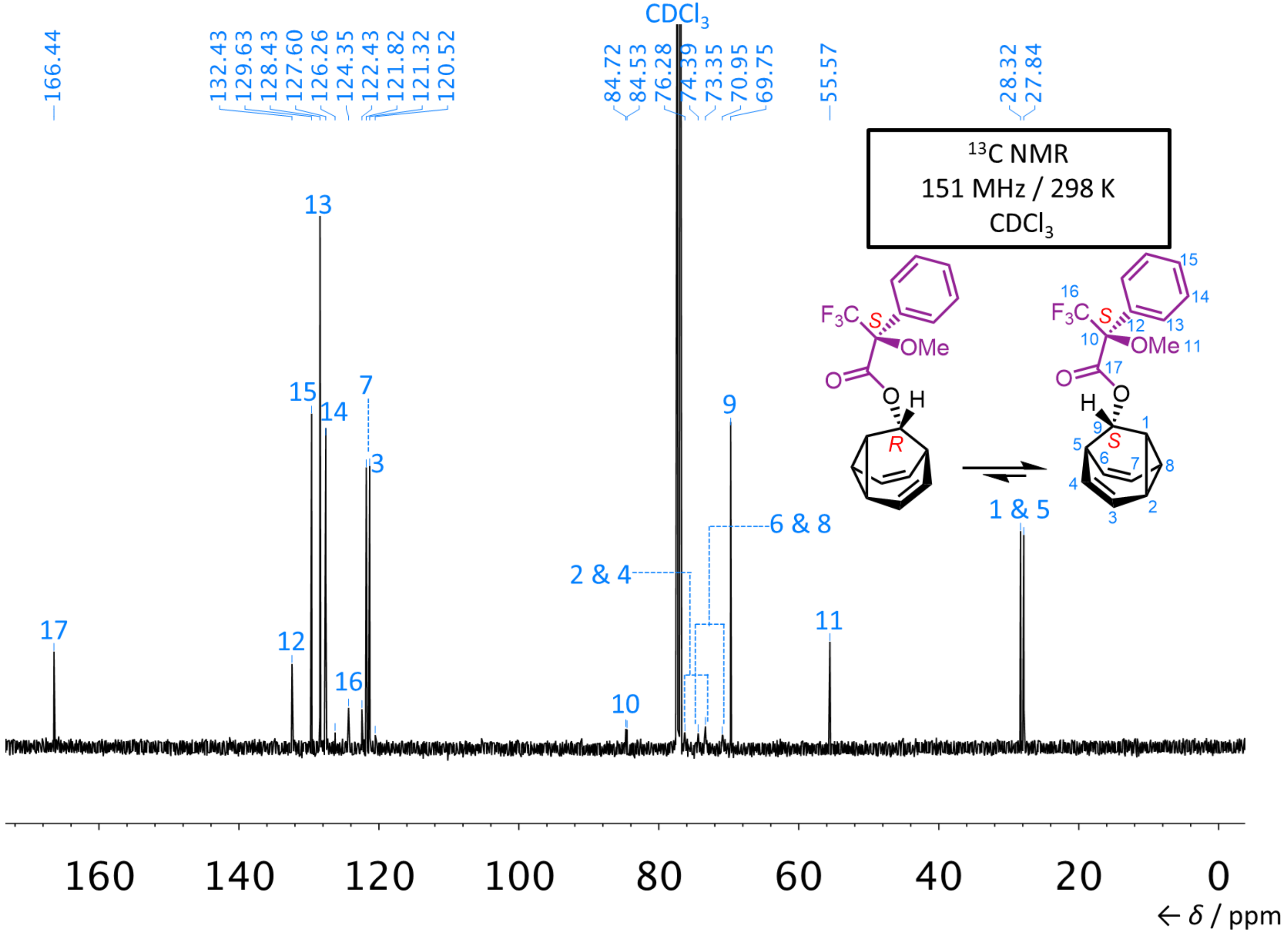

Figure S10. ^{13}C NMR spectrum of (*R*,*S*)/(*S*,*S*)-**2**.

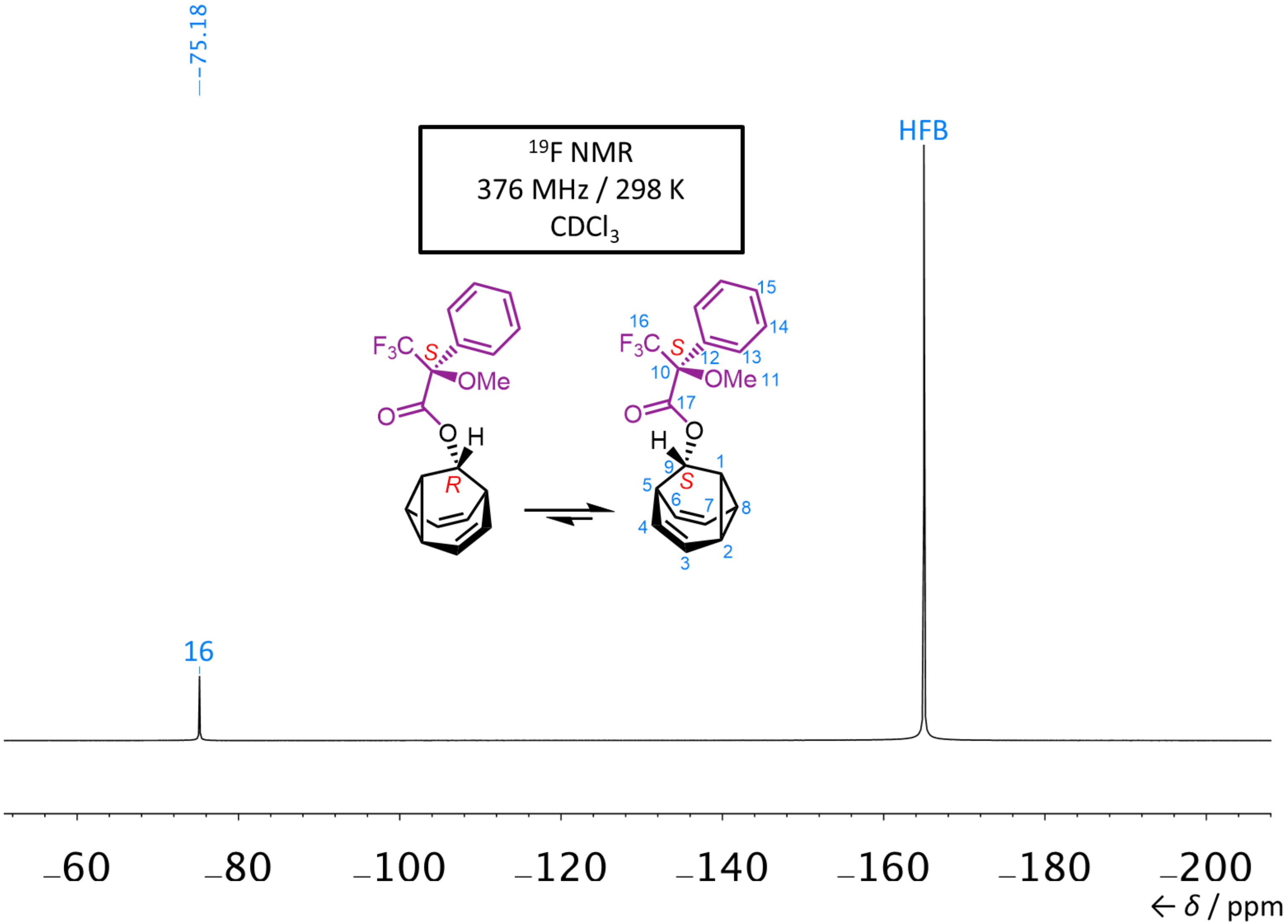

Figure S11. ^{19}F NMR spectrum of (*R*,*S*)/(*S*,*S*)-**2**.

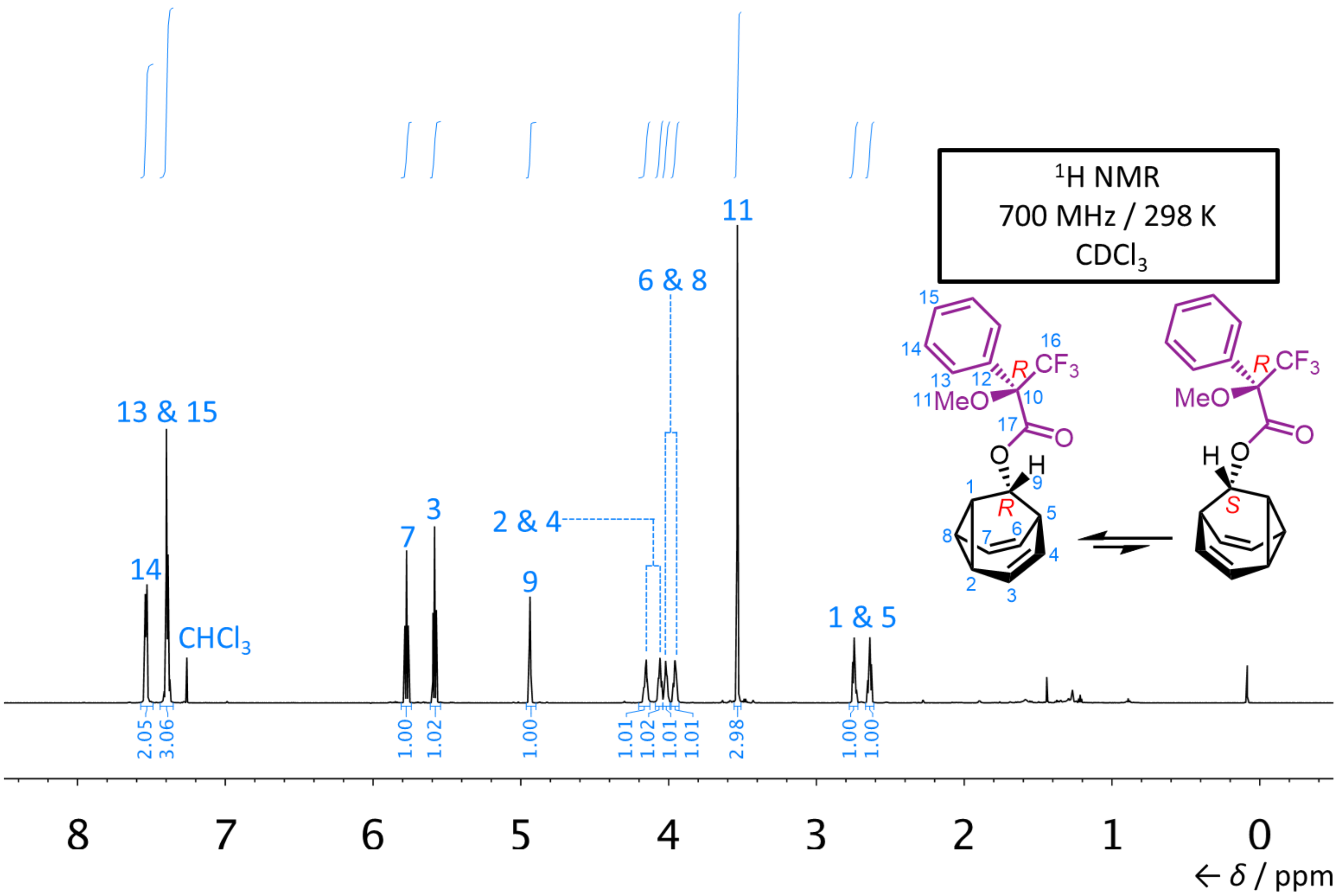

Figure S12. ^{1}H NMR spectrum of (*R*,*R*)/(*S*,*R*)-**2**.

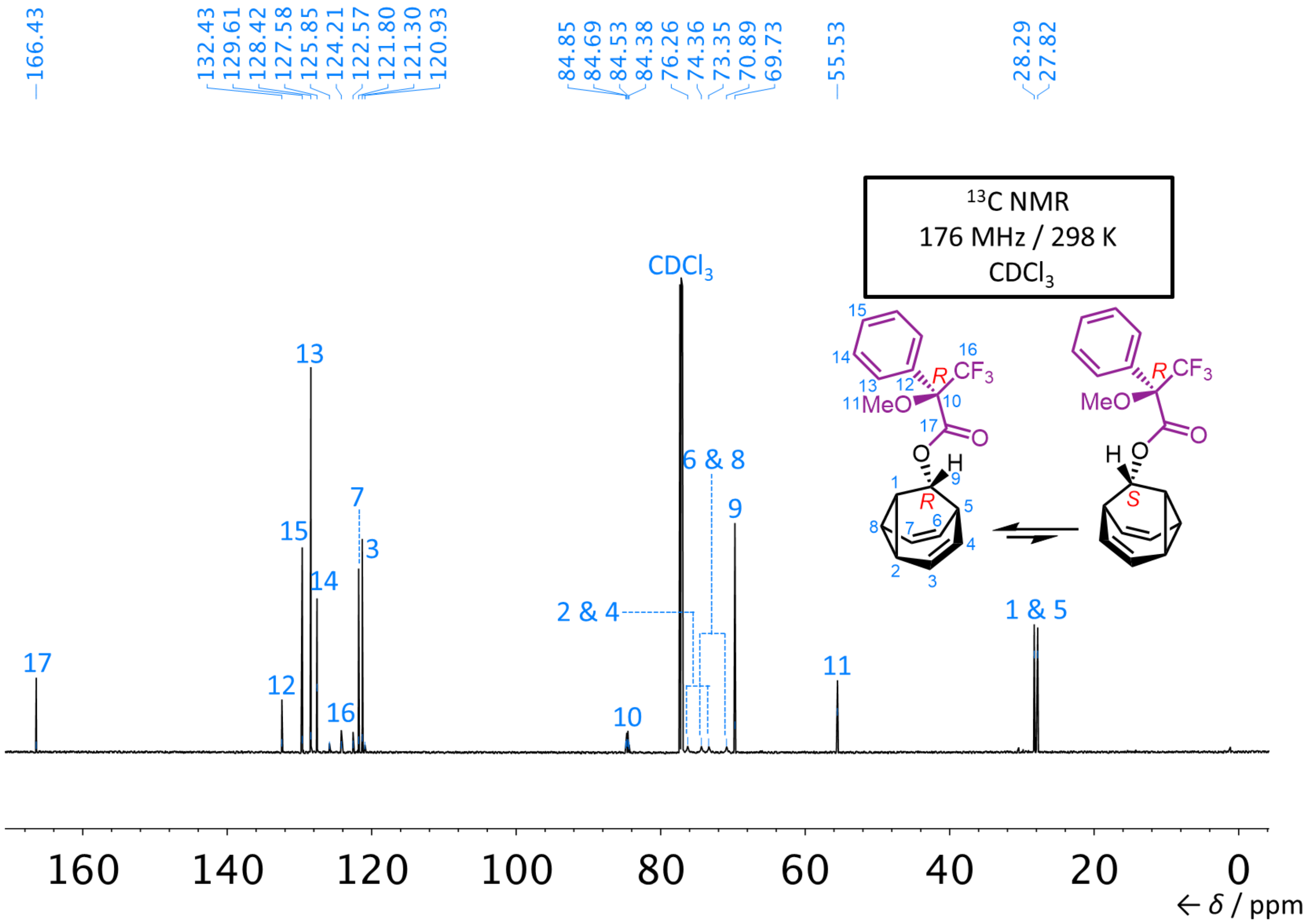

Figure S13. ^{13}C NMR spectrum of (*R*,*R*)/(*S*,*R*)-**2**.

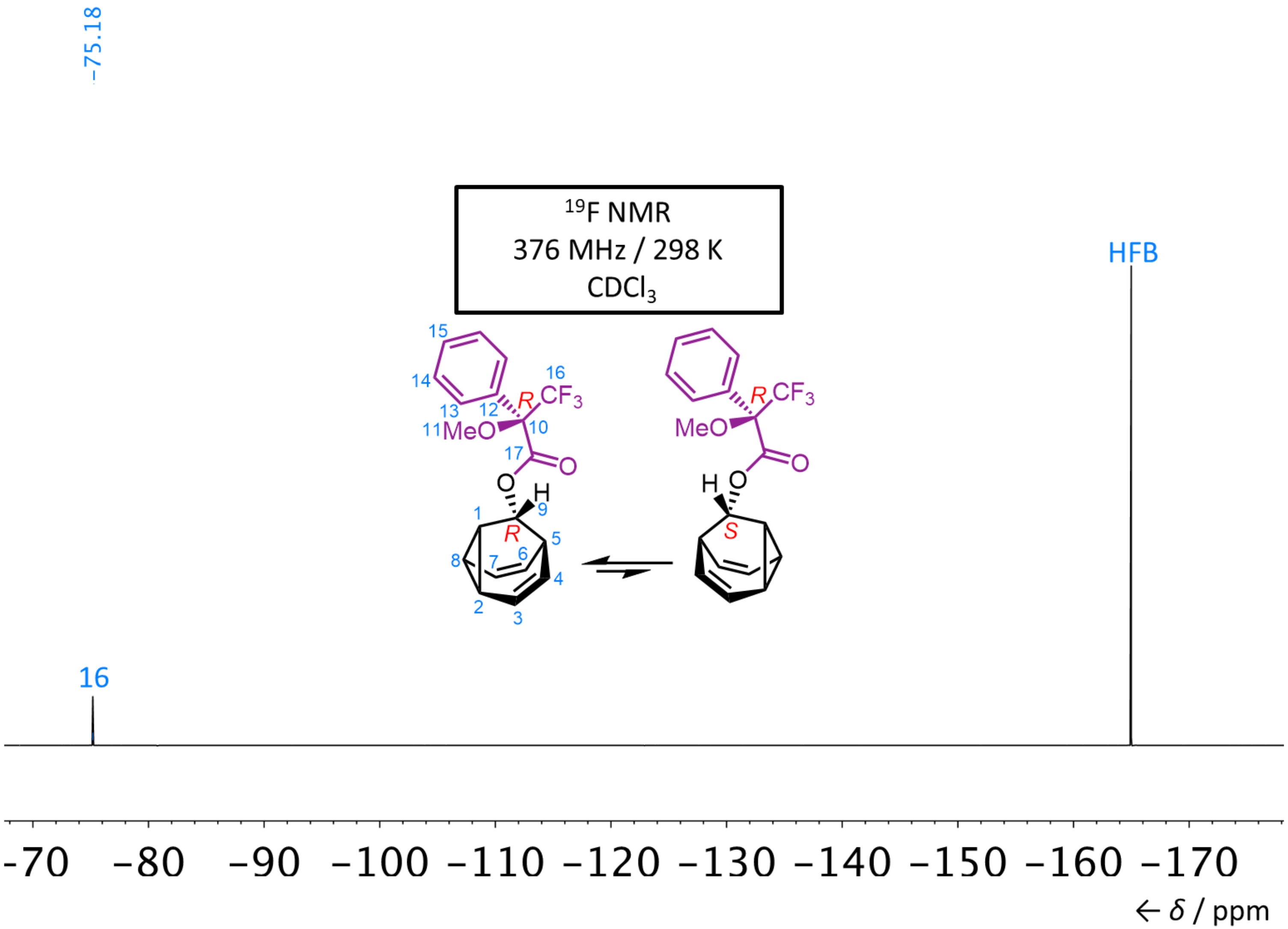

Figure S14. ^{19}F NMR spectrum of (*R*,*R*)/(*S*,*R*)-**2**.

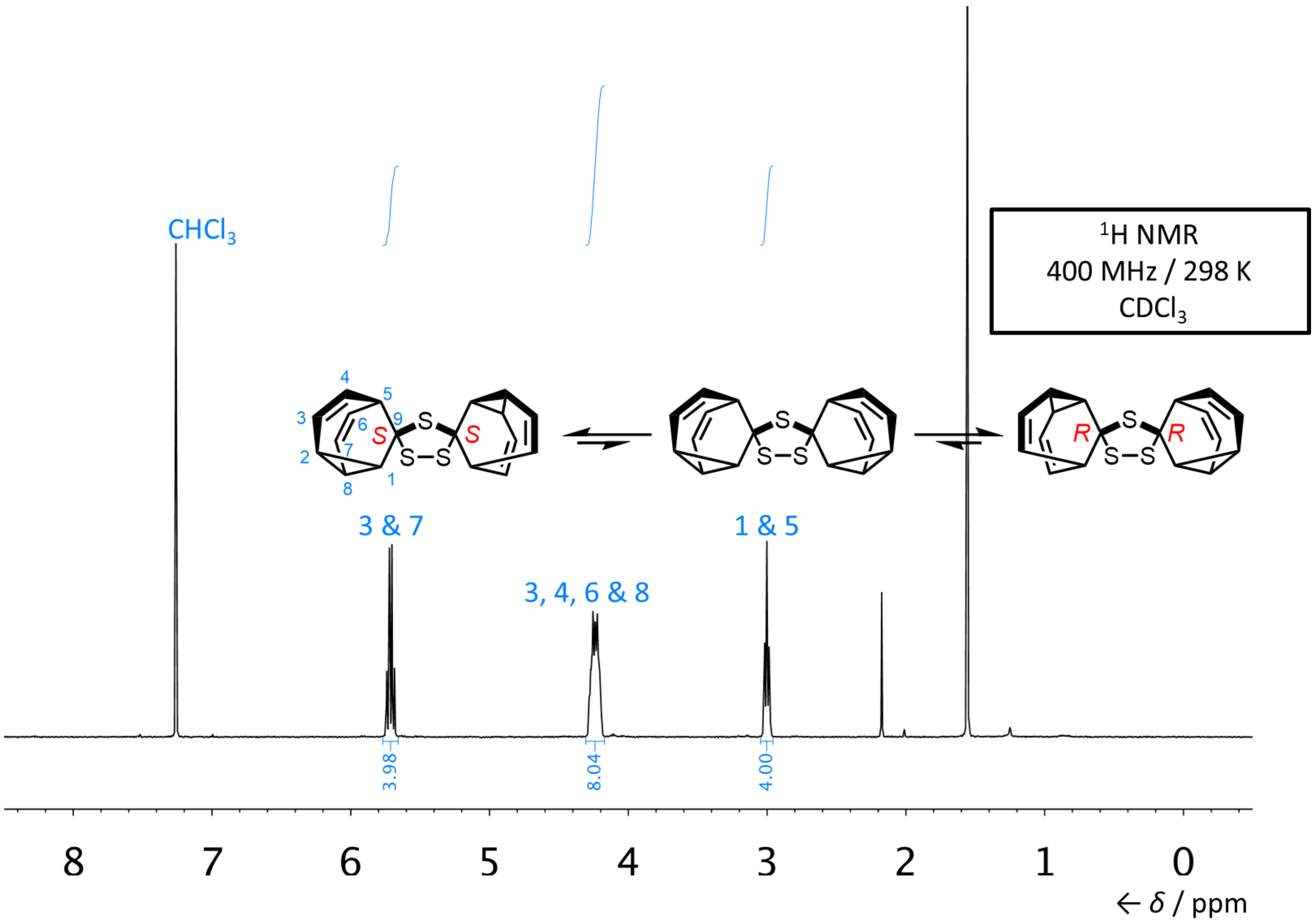

Figure S15. ^{1}H NMR spectrum of (*R,R*)/*meso*/(*S,S*)-**4**.

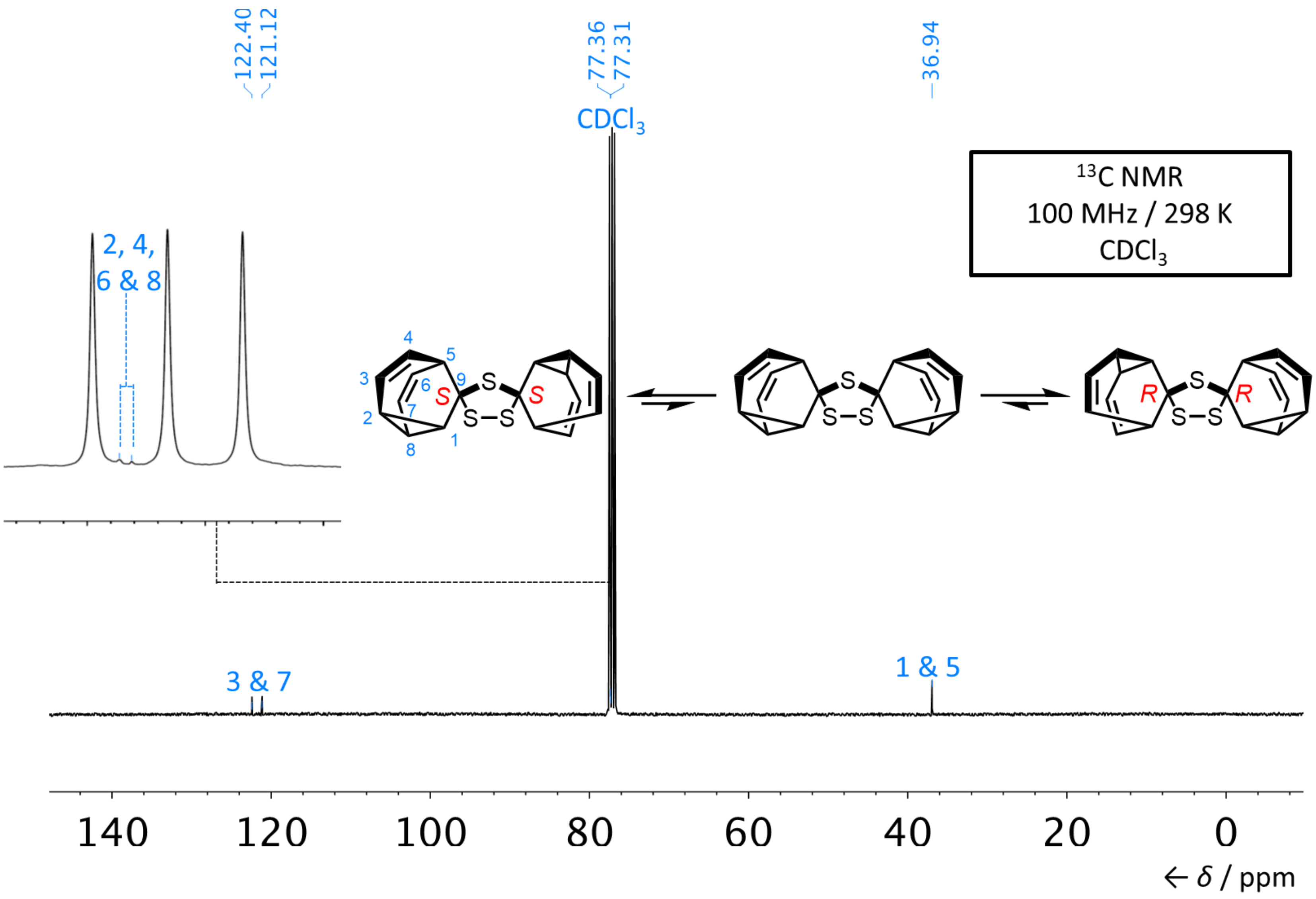

Figure S16. ^{13}C NMR spectrum of (*R,R*)/*meso*/(*S,S*)-**4**.

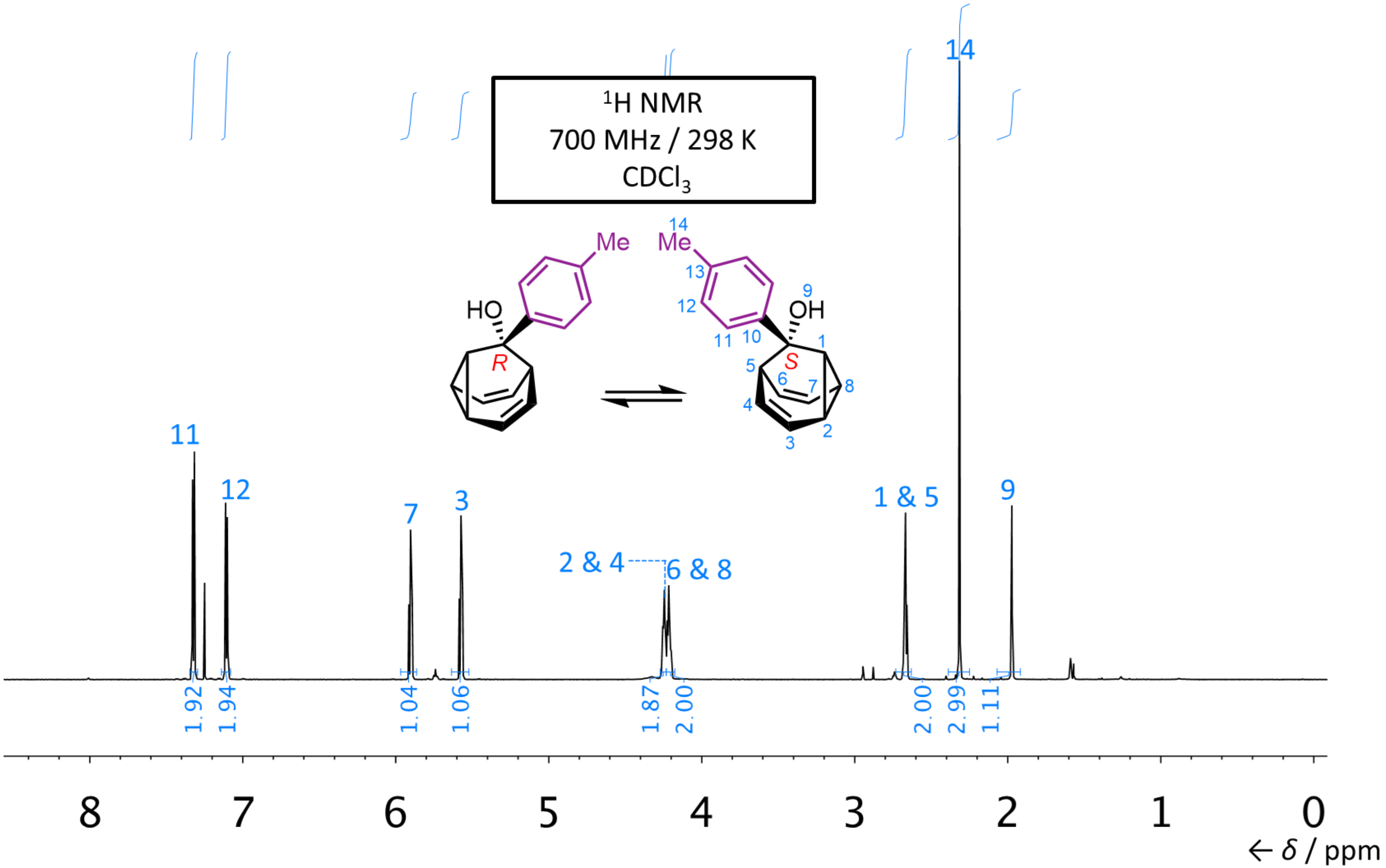

Figure S17. ^{1}H NMR spectrum of *(R)*/(*S*)-**6**.

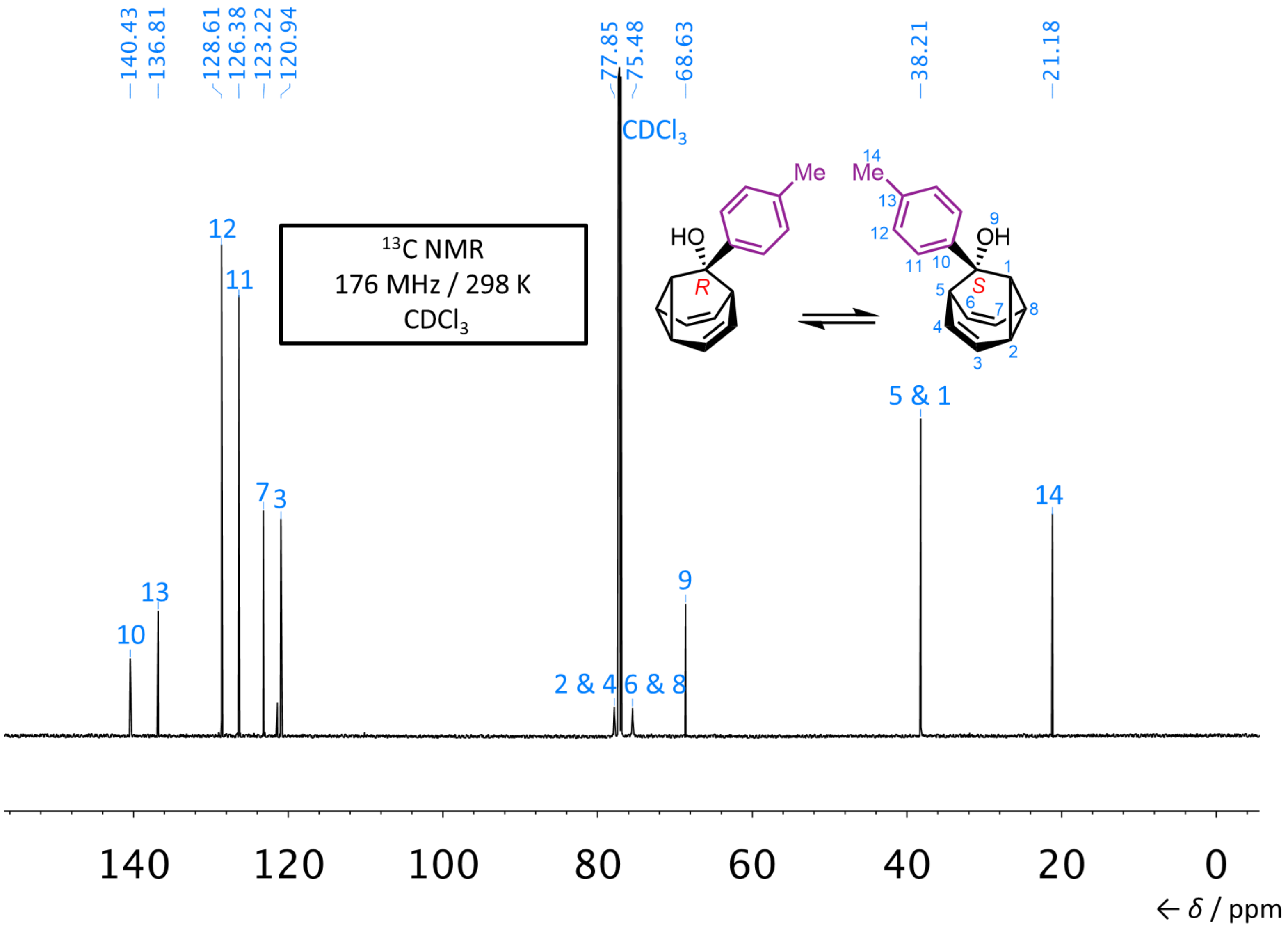

Figure S18. ^{13}C NMR spectrum of *(R)*/(*S*)-**6**.

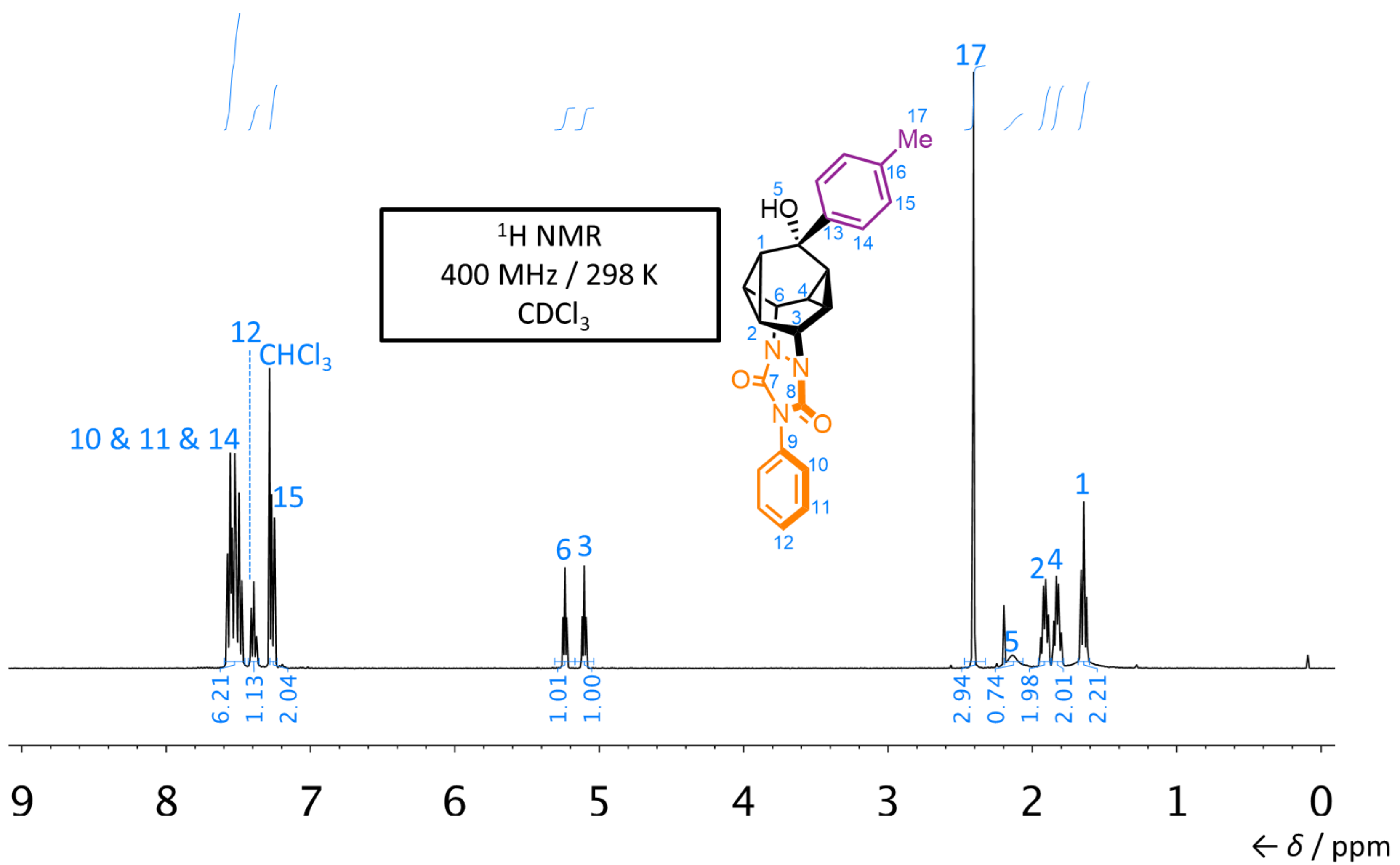

Figure S19. ^{1}H NMR spectrum of **7**.

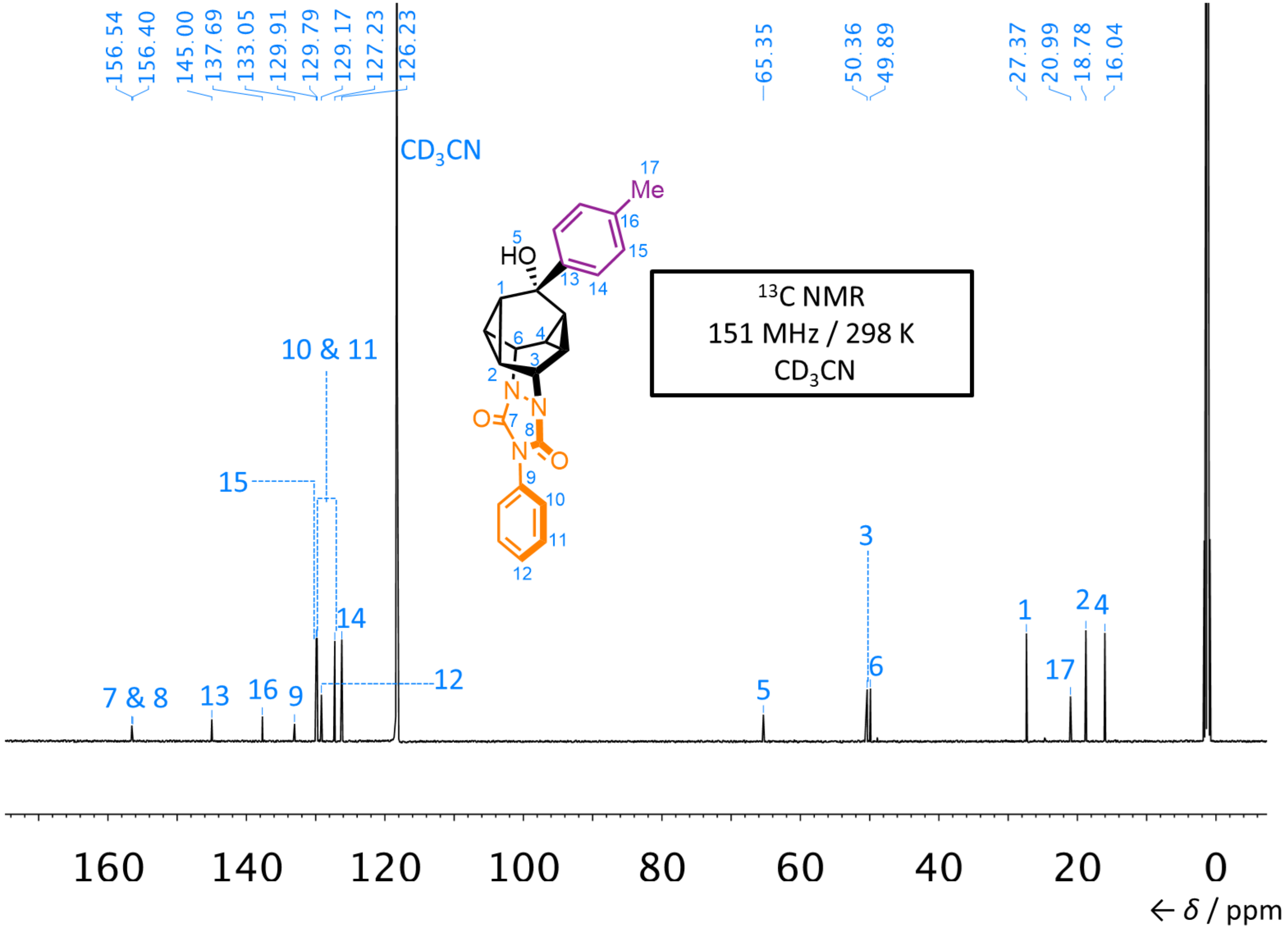

Figure S20. ^{13}C NMR spectrum of **7**.

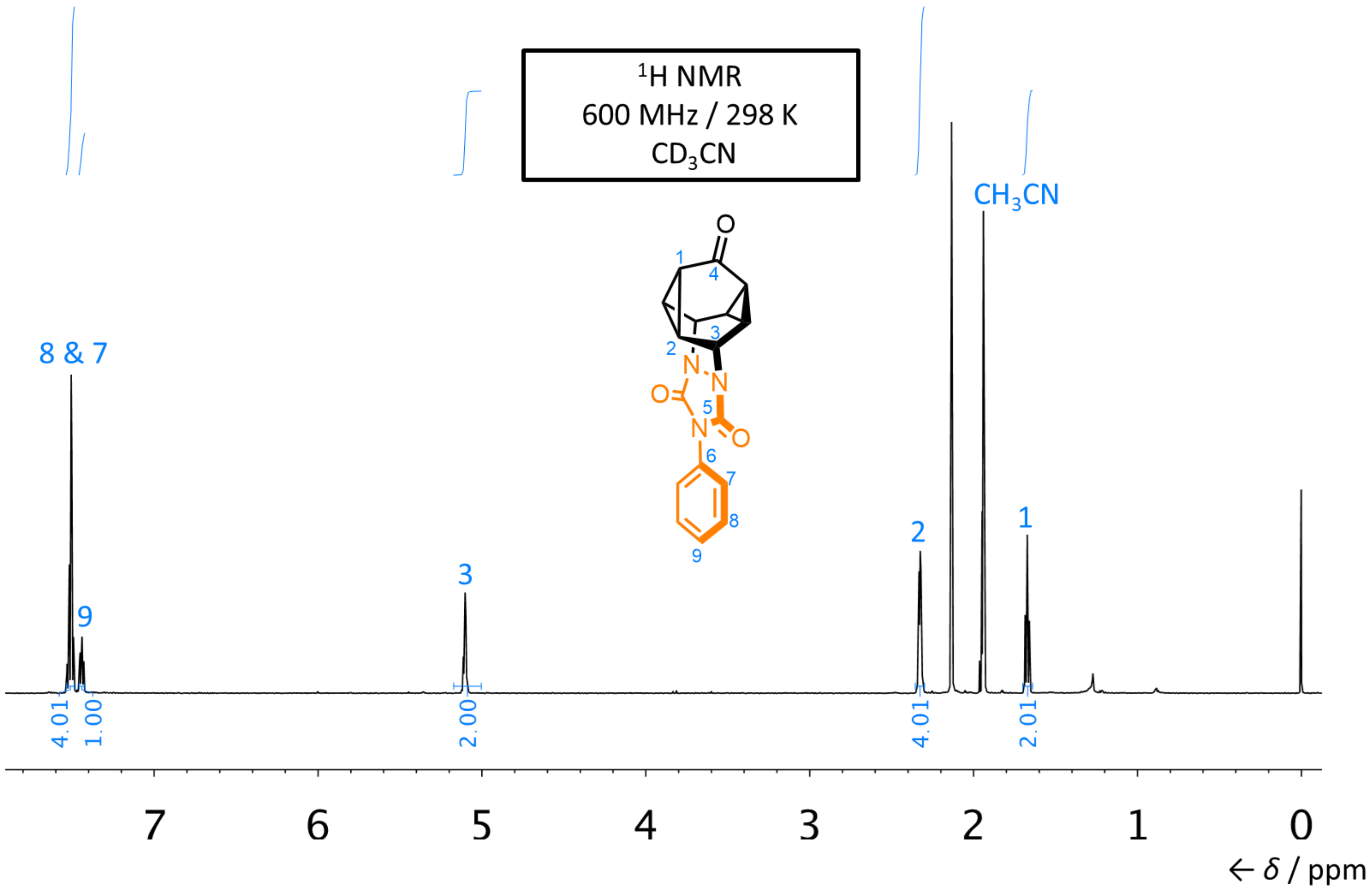

Figure S21. ^{1}H NMR spectrum of **S2**.

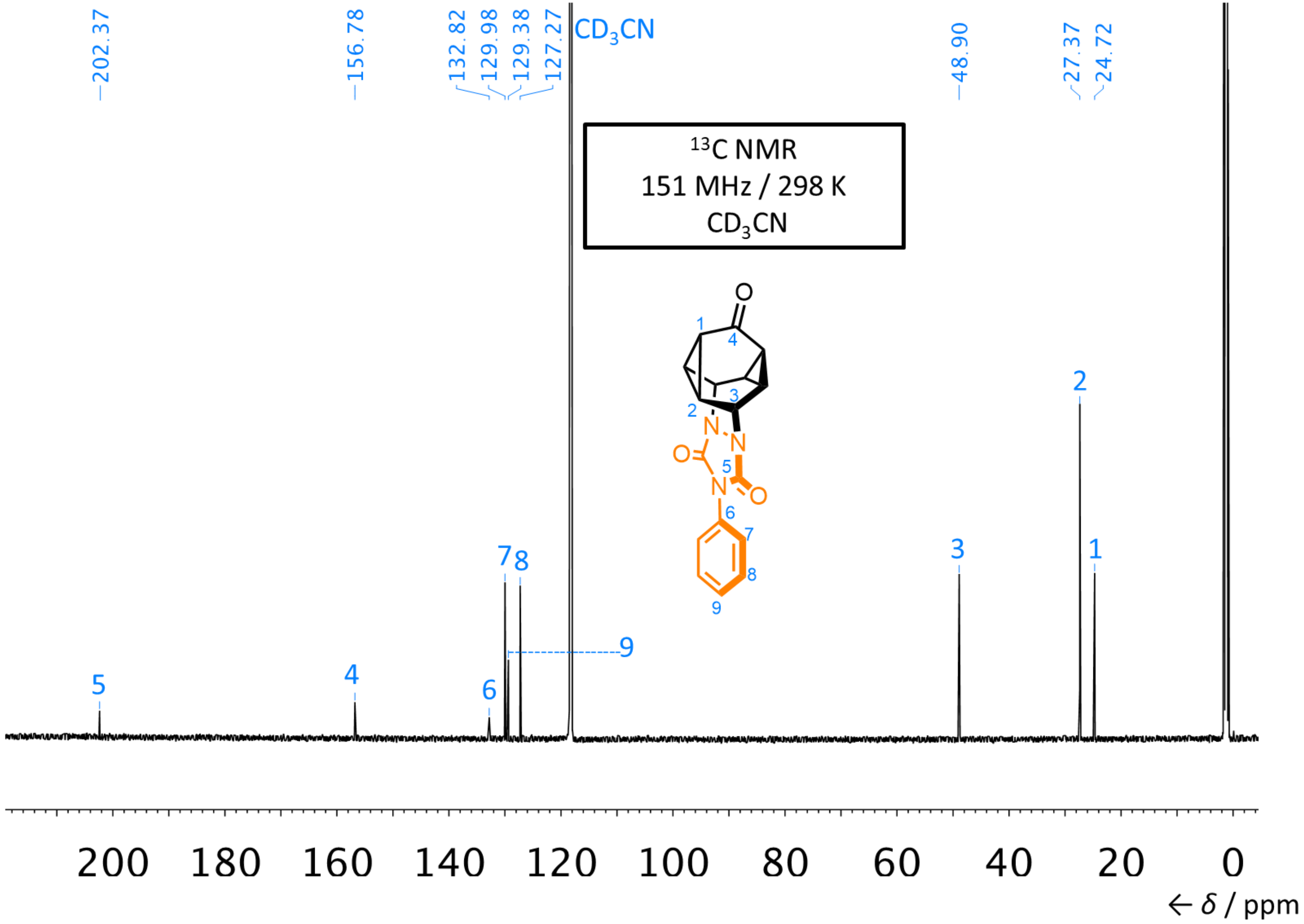

Figure S22. ^{13}C NMR spectrum of **S2**.

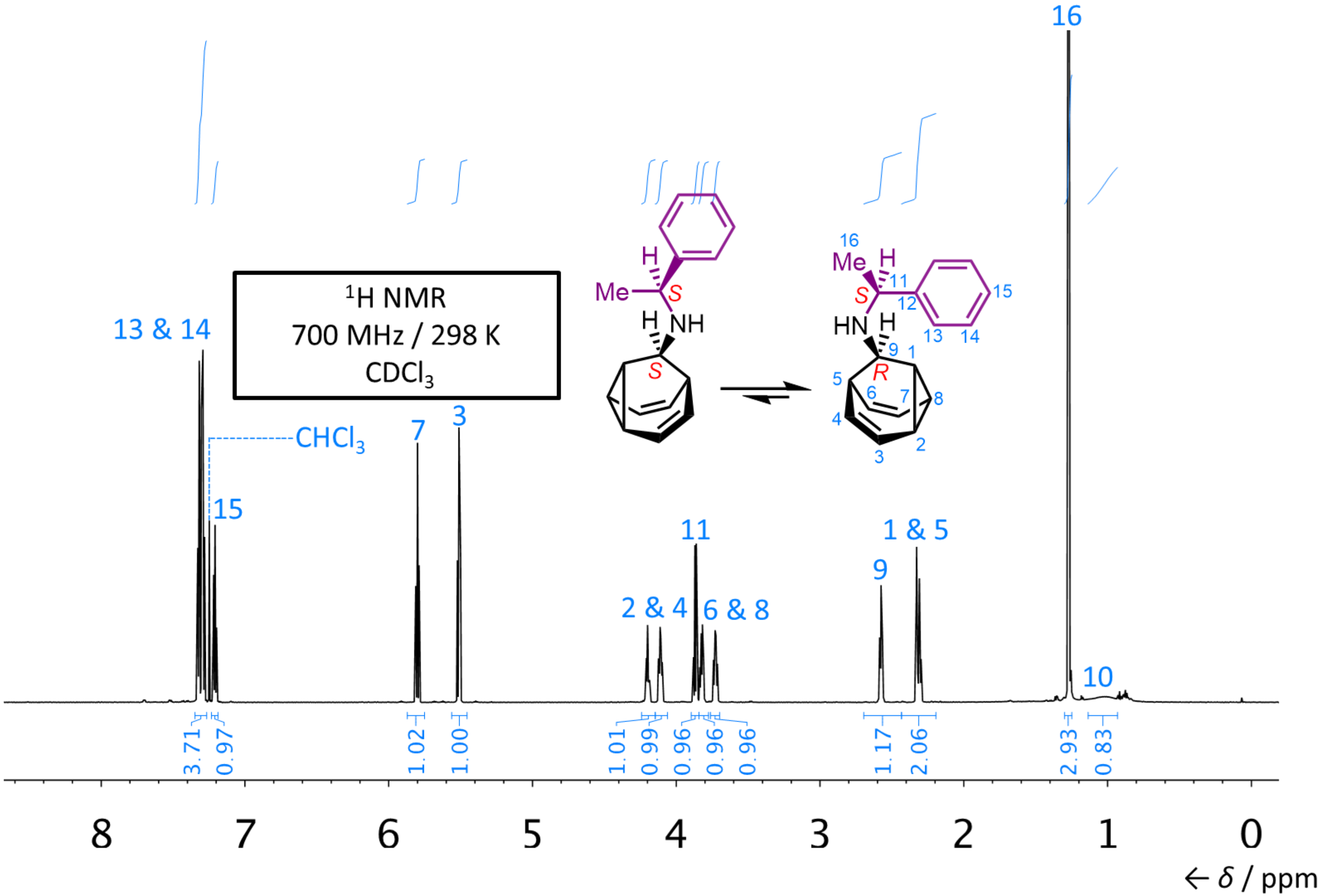

Figure S23. ^{1}H NMR spectrum of (*S*,*S*)/(*R*,*S*)-**5**.

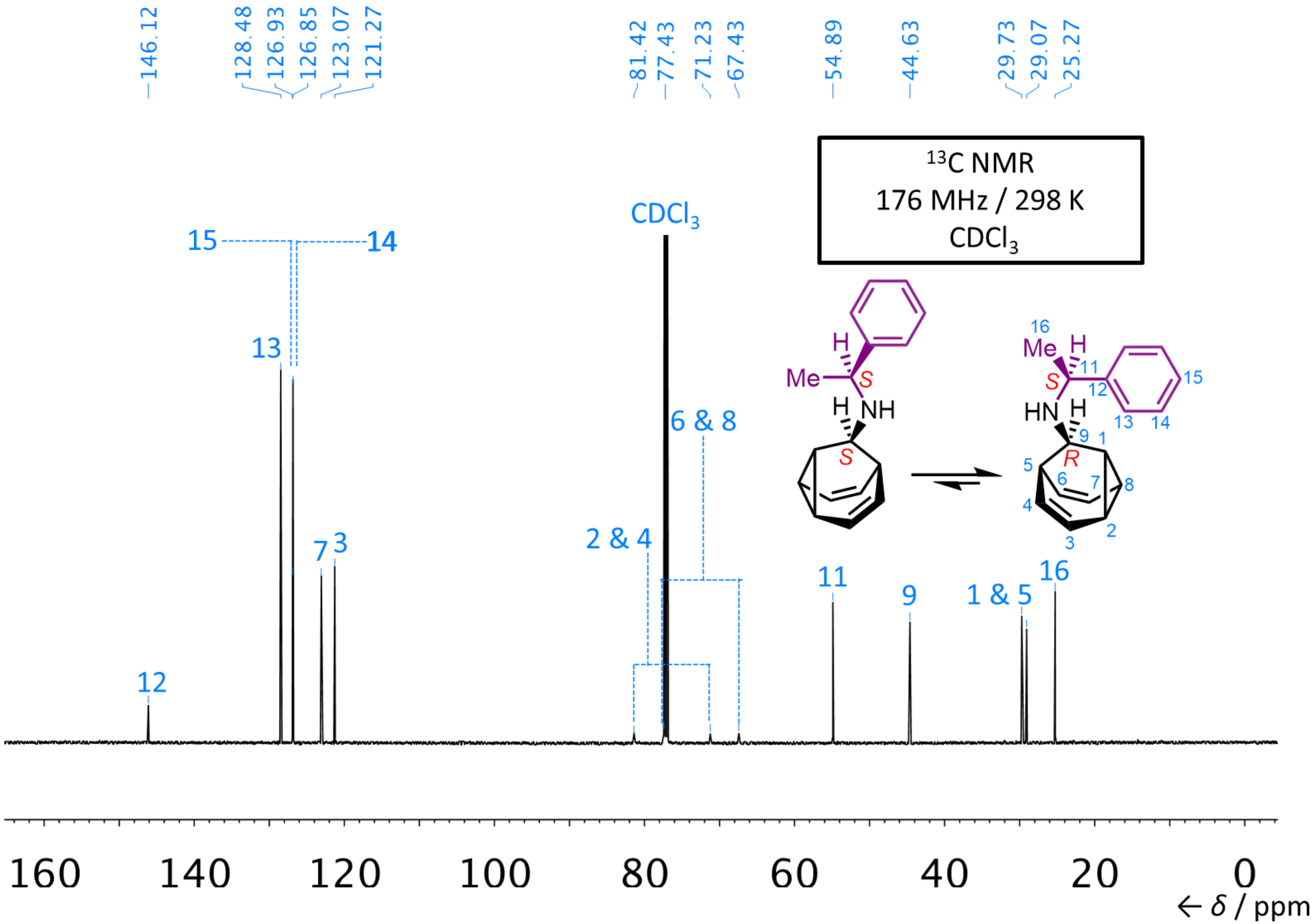

Figure S24. ^{13}C NMR spectrum of (*S*,*S*)/(*R*,*S*)-**5**.

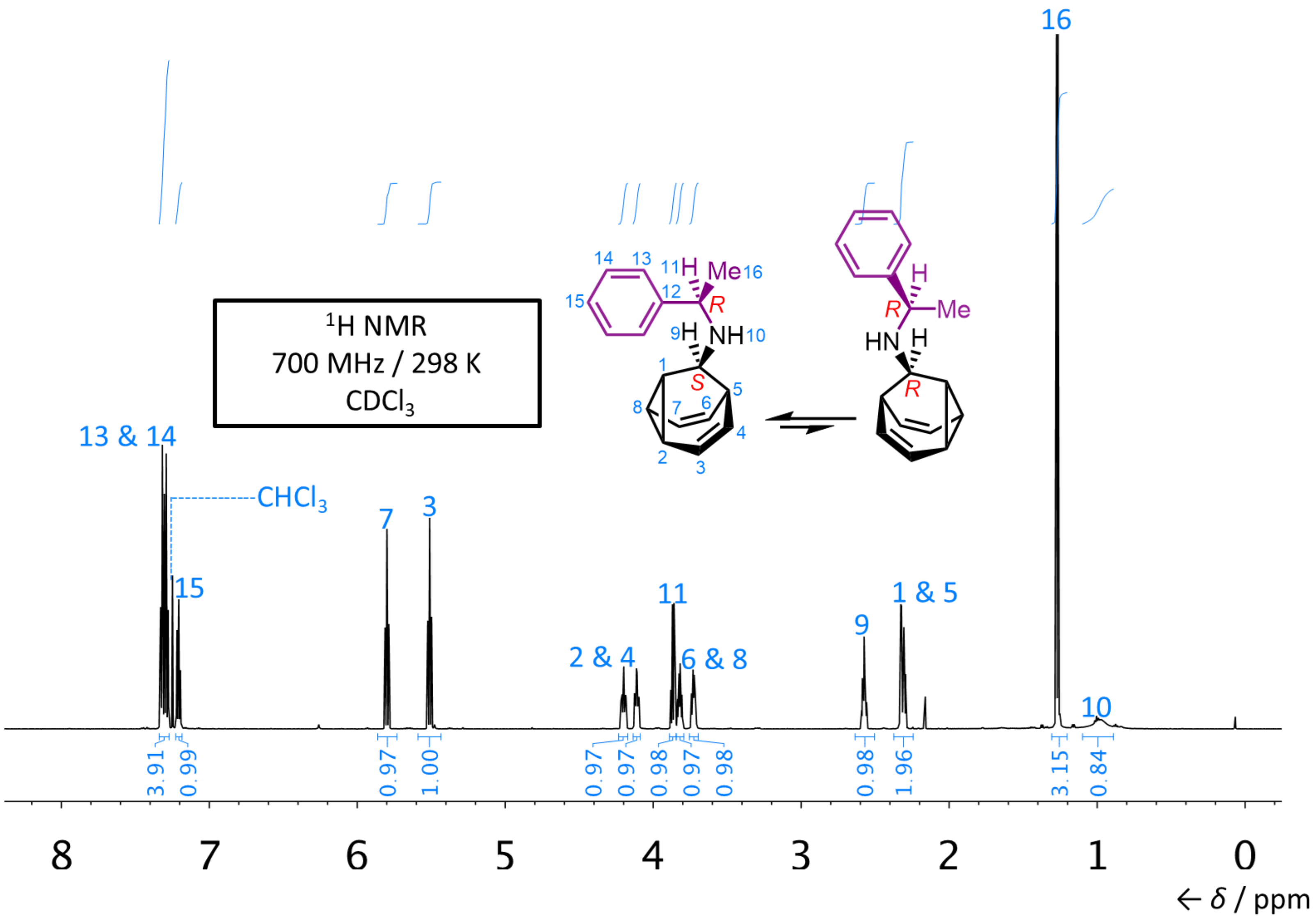

Figure S25. ^{1}H NMR spectrum of (*R*,*S*)/(*R*,*R*)-**5**.

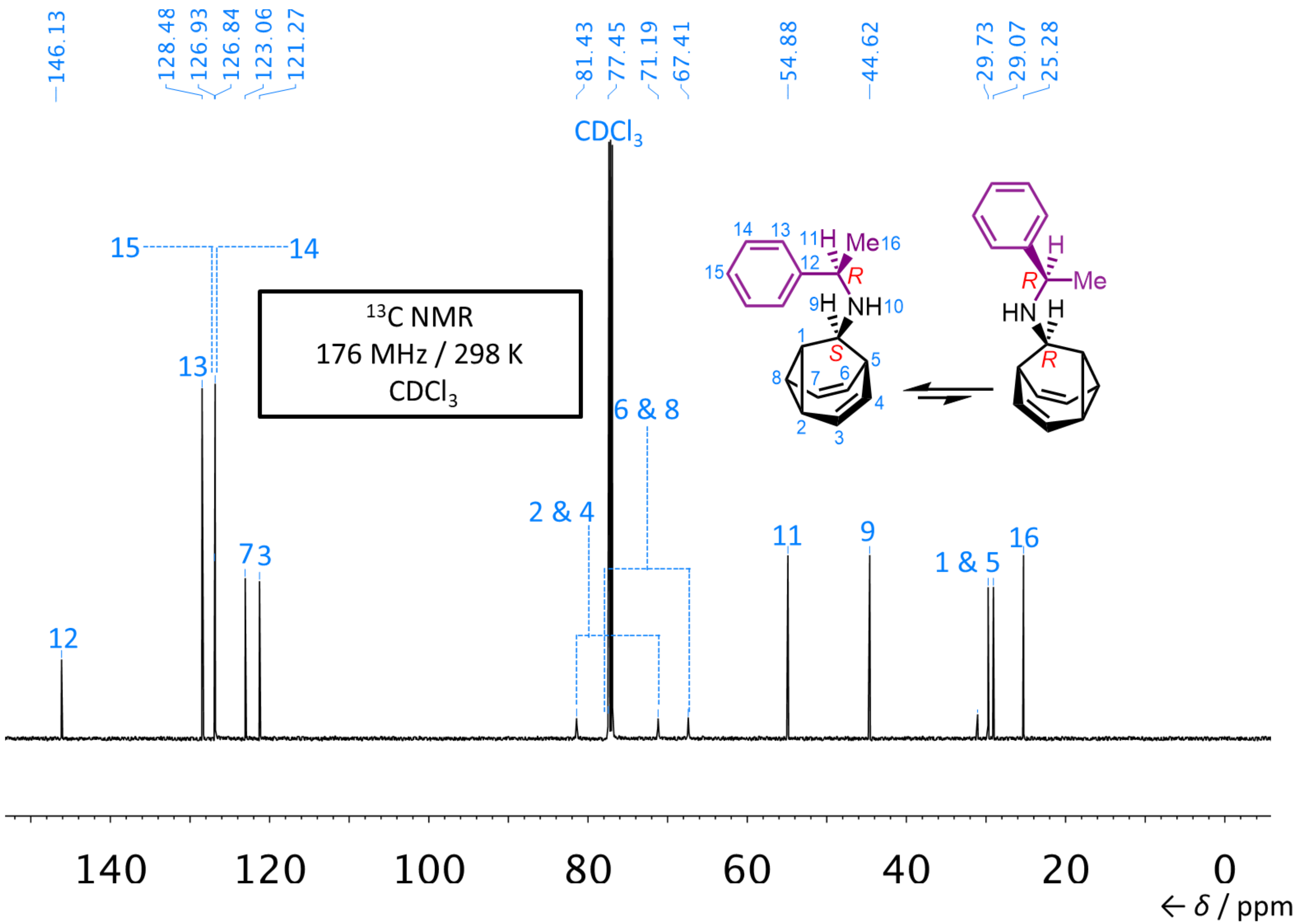

Figure S26. ^{13}C NMR spectrum of (*R*,*S*)/(*R*,*R*)-**5**.

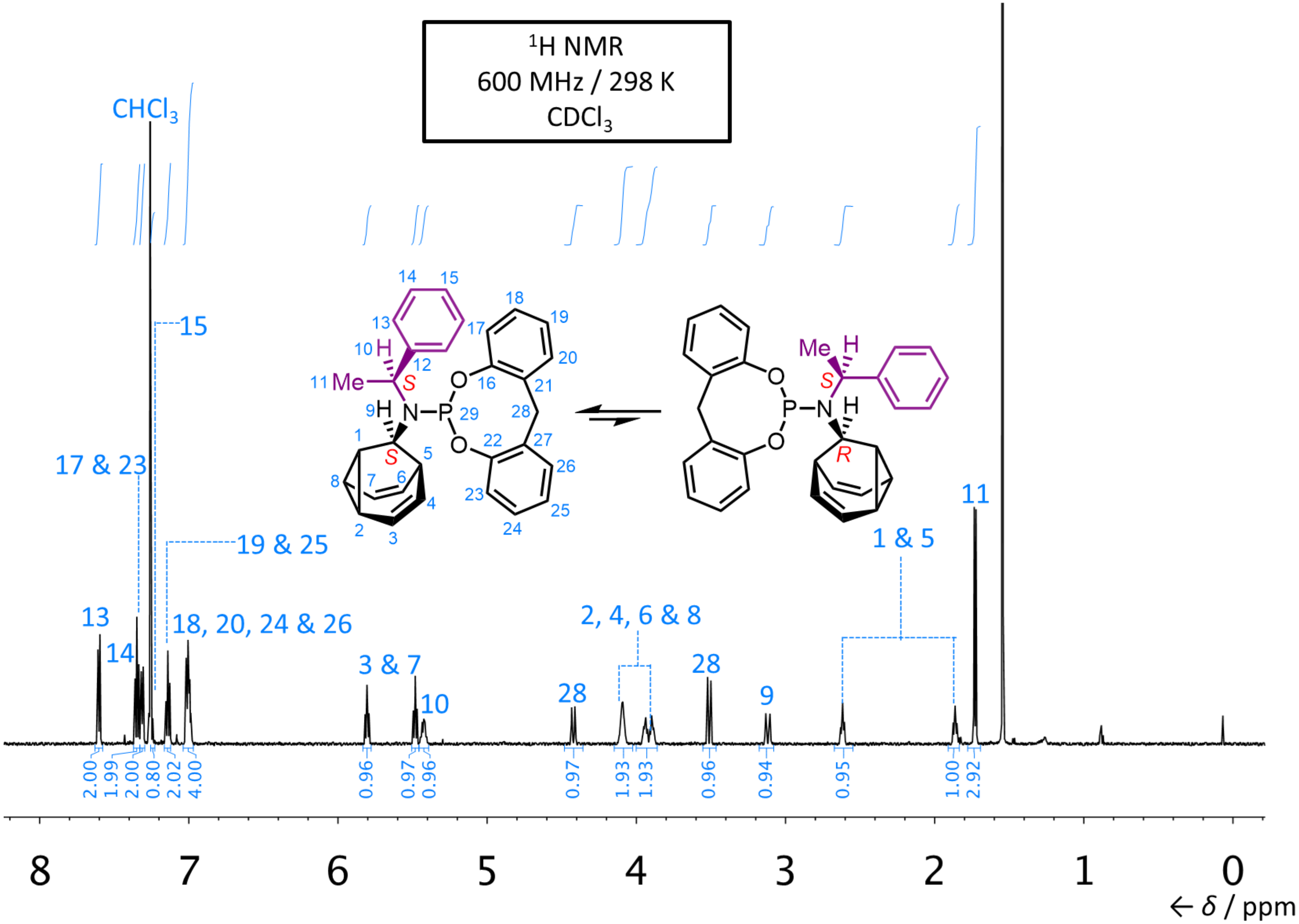

Figure S27. ^{1}H NMR spectrum of (*R*,*S*)/(*S*,*S*)-$\mathbf{L_{BB1}}$.

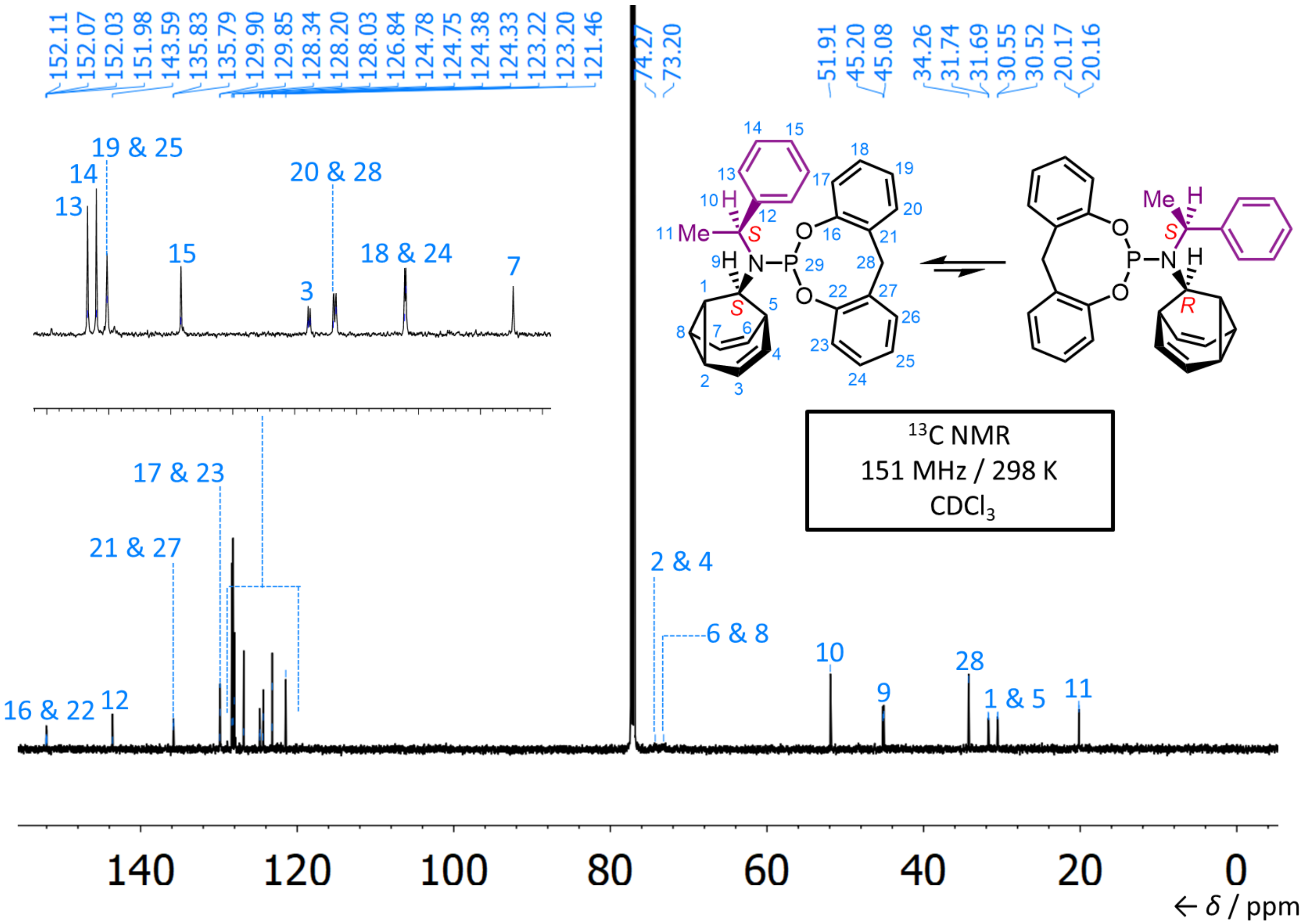

Figure S28. ^{13}C NMR spectrum of (*R*,*S*)/(*S*,*S*)-**L**$_{\mathbf{BB1}}$.

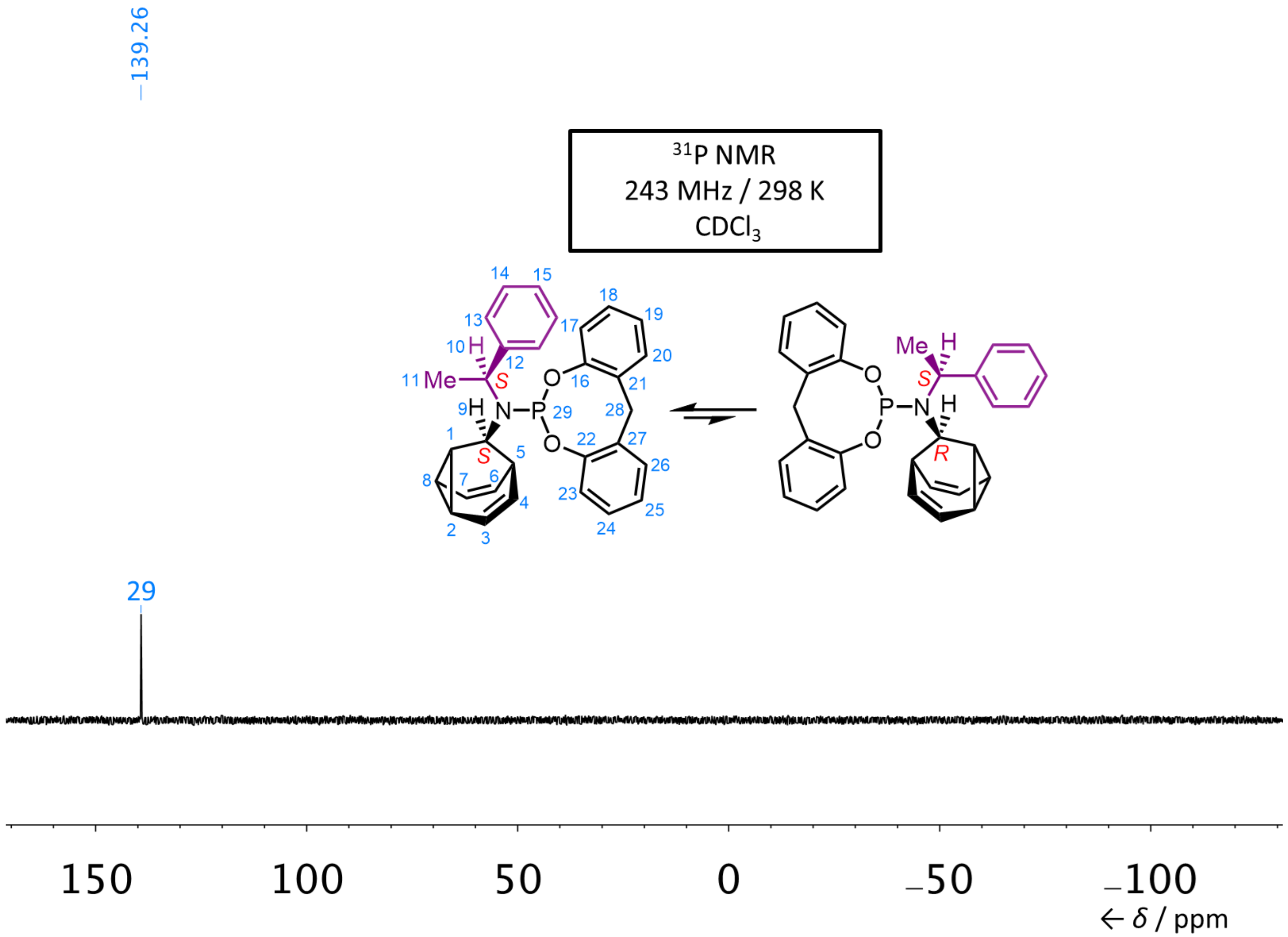

Figure S29. ^{31}P NMR spectrum of (R,S)/(S,S)-$\mathbf{L_{BB1}}$.

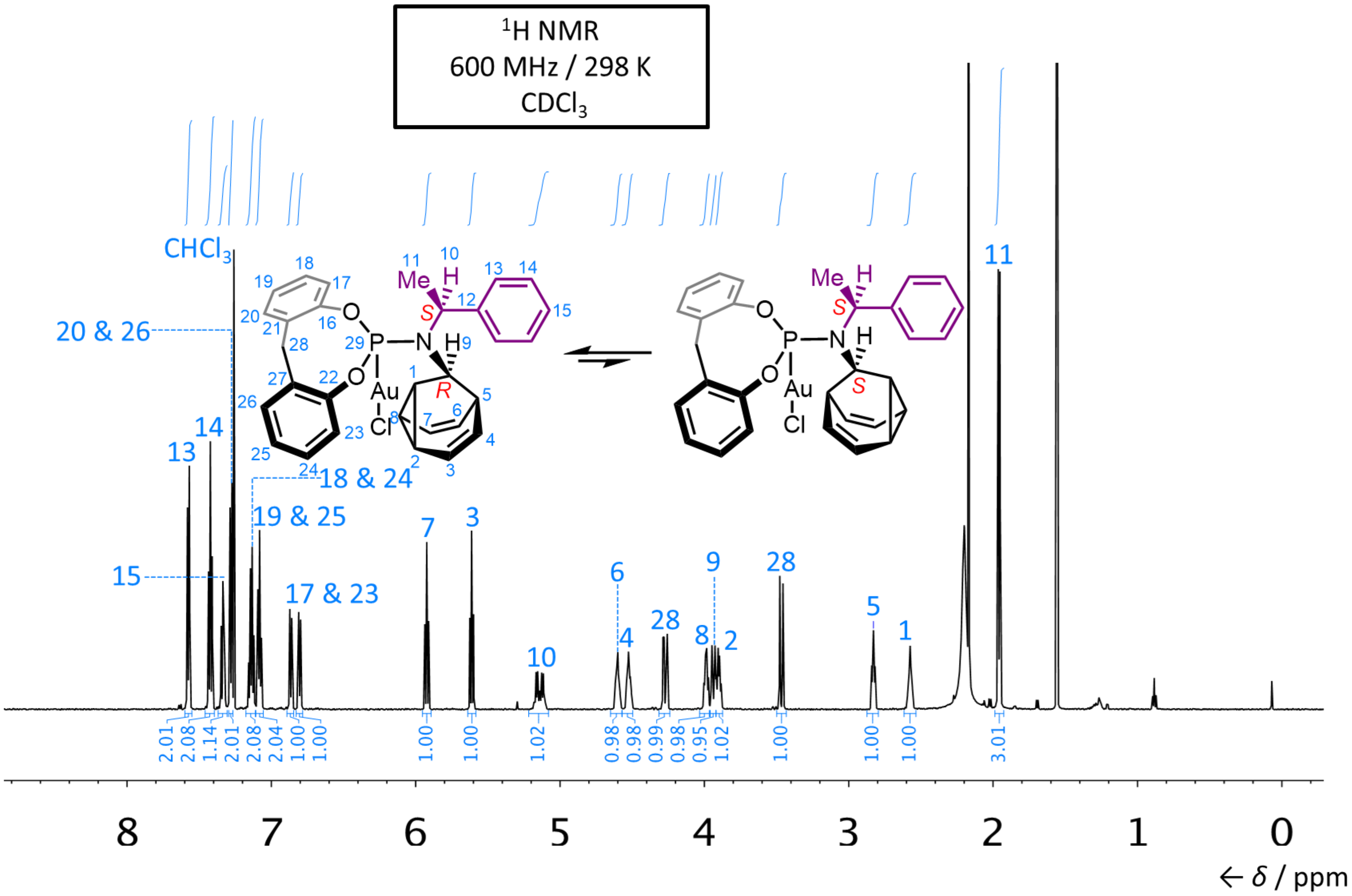

Figure S30. ^{1}H NMR spectrum of (R,S)/(S,S)-**L**$_{\mathbf{BB1}}$AuCl.

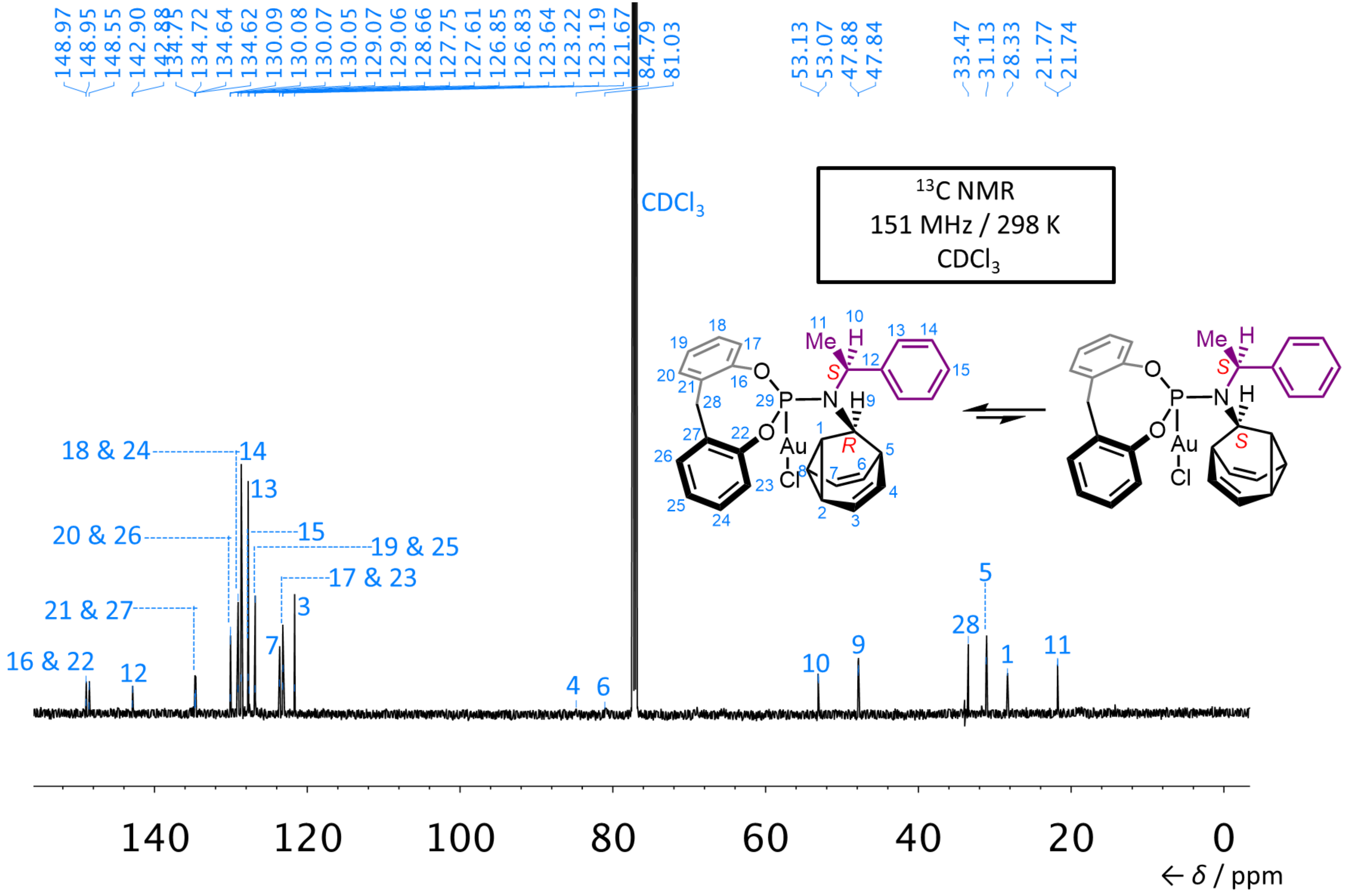

Figure S31. ^{13}C NMR spectrum of (*R*,*S*)/(*S*,*S*)-$\mathbf{L_{BB1}}$AuCl.

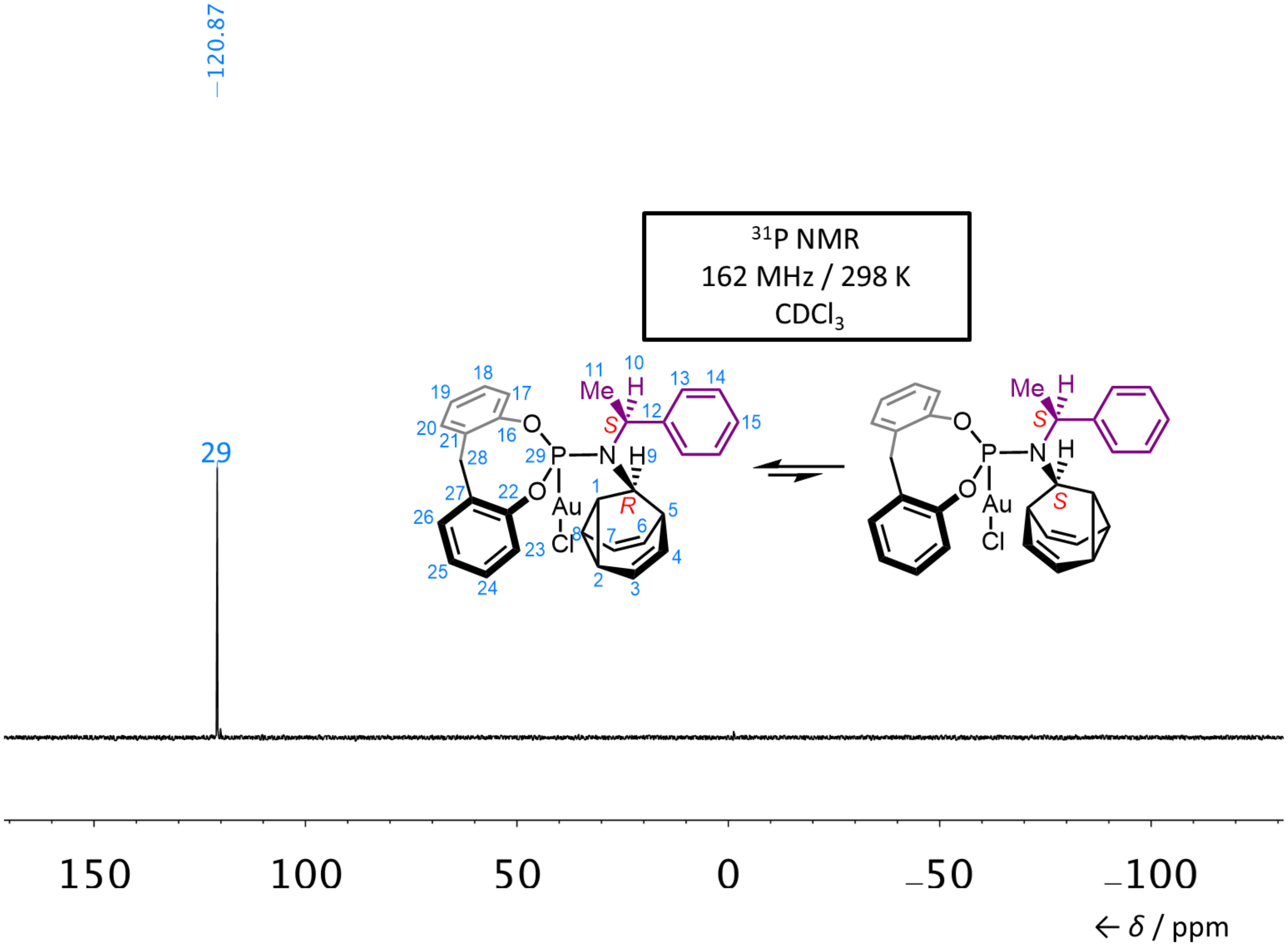

Figure S32. ^{31}P NMR spectrum of (*R*,*S*)/(*S*,*S*)-$\mathbf{L_{BB1}}$AuCl.

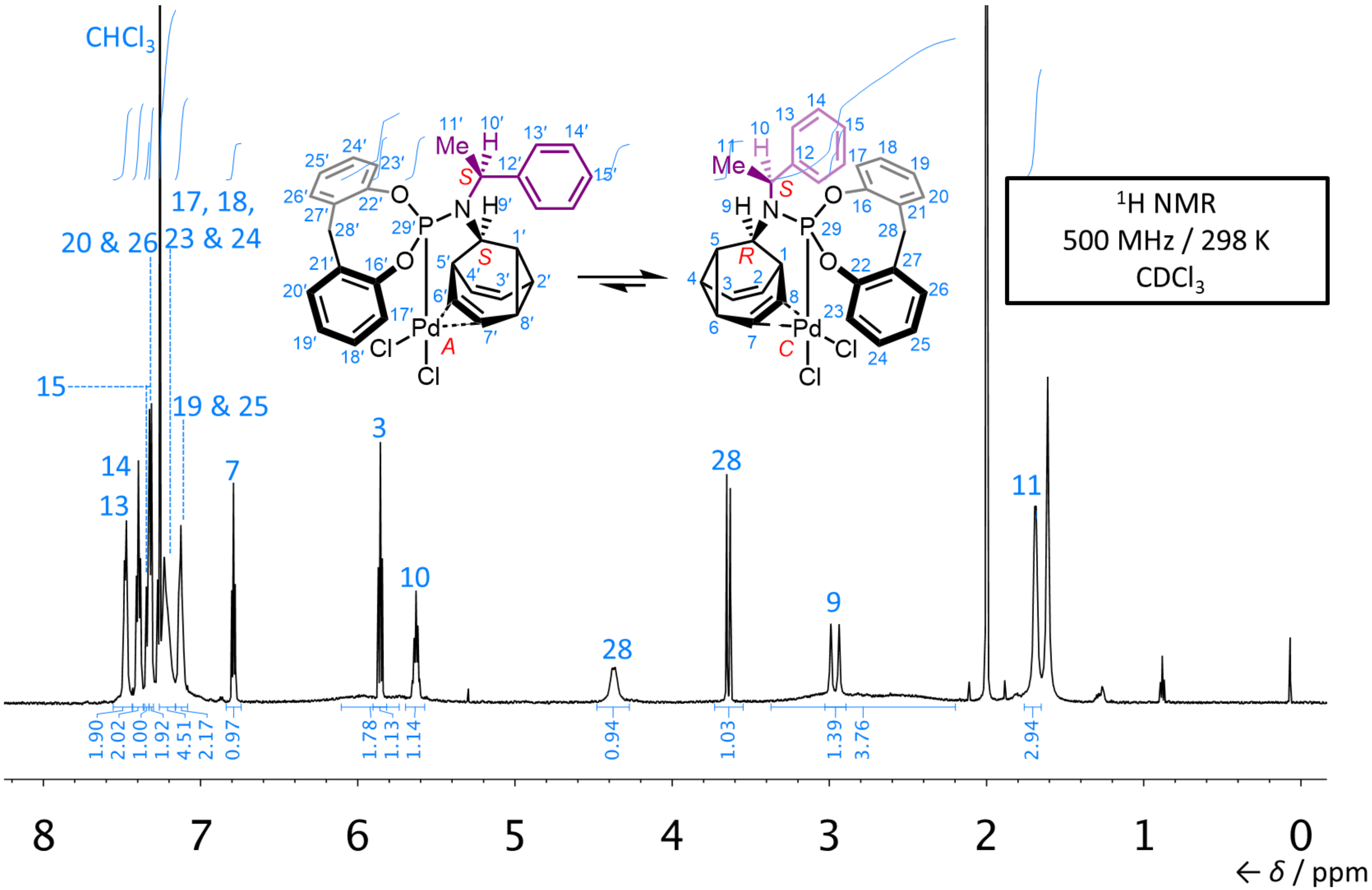

Figure S33. ^{1}H NMR spectrum of (*A*,*S*,*S*)/(*C*,*R*,*S*)-$\mathbf{L_{BB1}}$PdCl$_2$ (298 K).

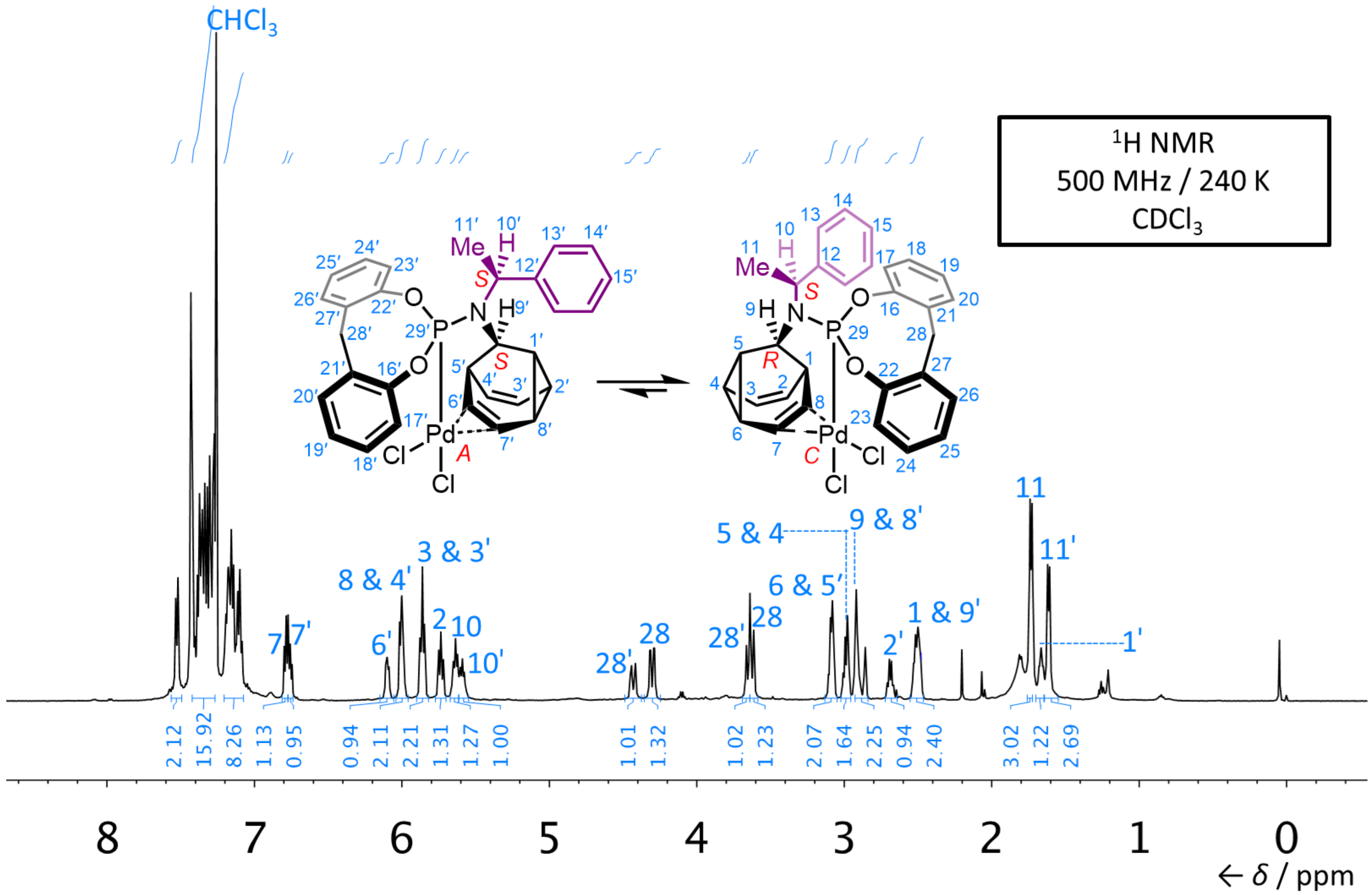

Figure S34. ^{1}H NMR spectrum of (A,S,S)/(C,R,S)-$\mathbf{L_{BB1}}PdCl_2$ (240 K).

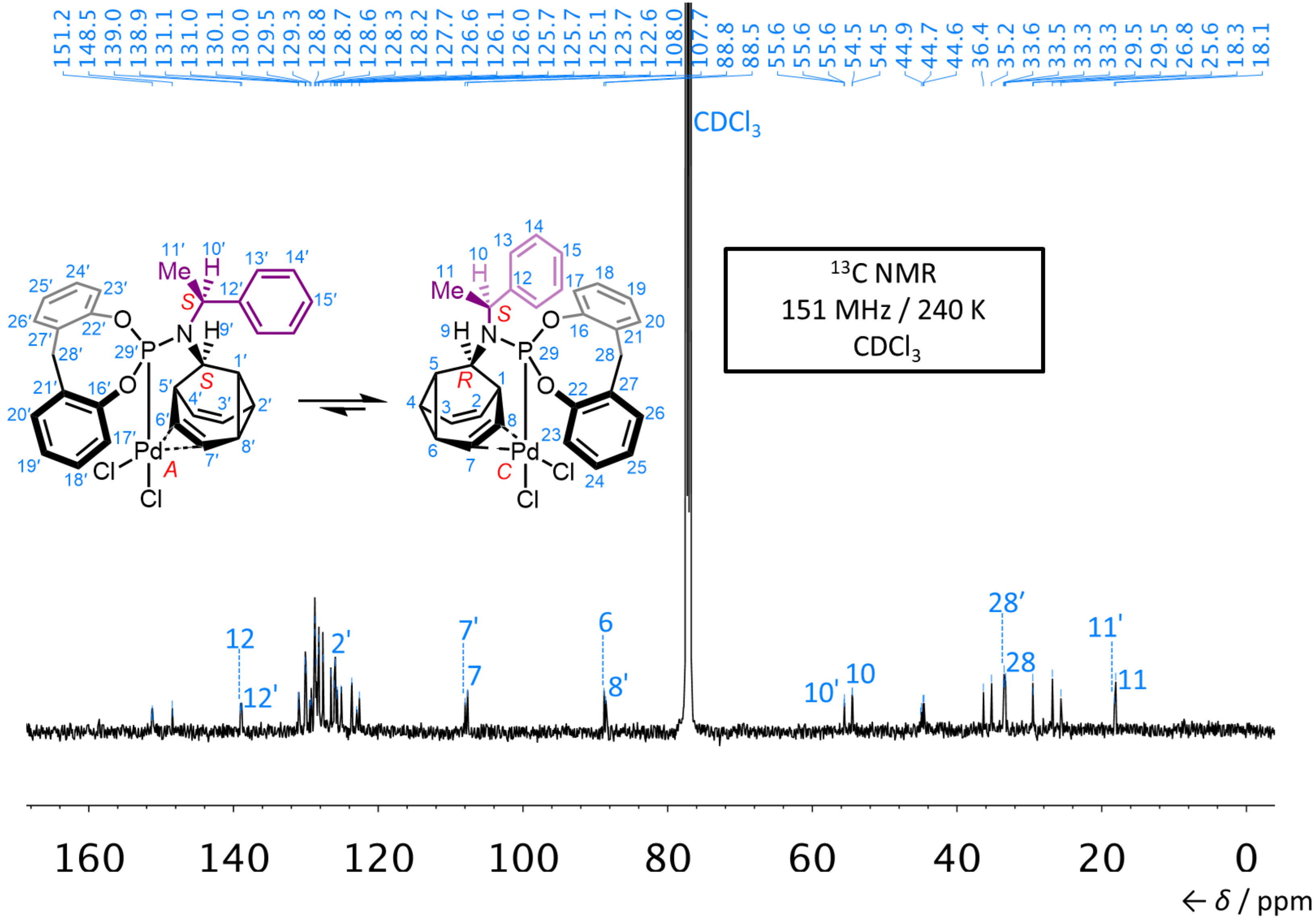

Figure S35. ^{13}C NMR spectrum of (*A*,*S*,*S*)/(*C*,*R*,*S*)-$\mathbf{L_{BB1}}$PdCl$_2$ (240 K).

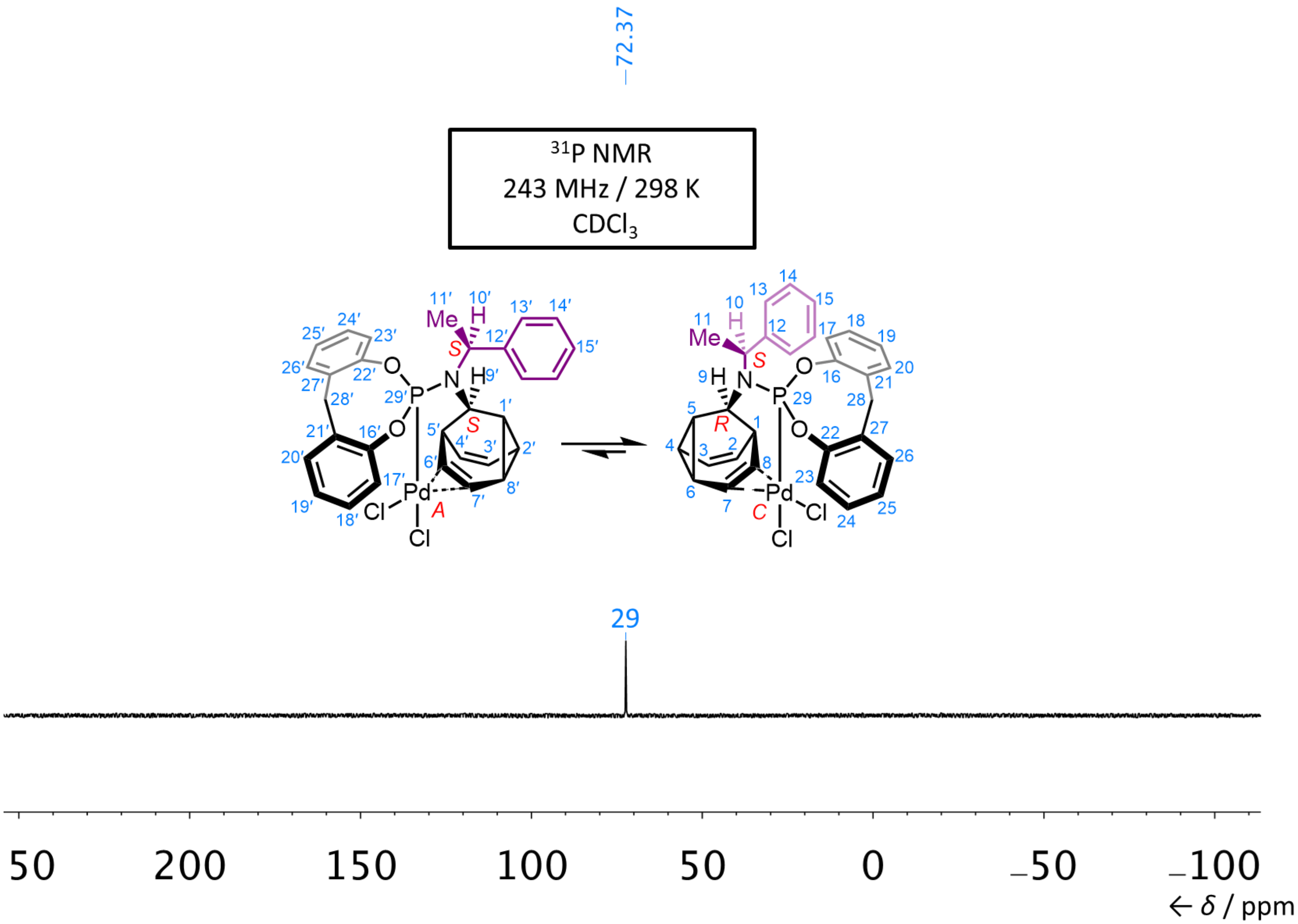

Figure S36. ^{31}P NMR spectrum of (*A*,*S*,*S*)/(*C*,*R*,*S*)-$\mathbf{L_{BB1}}$PdCl$_2$ (298 K).

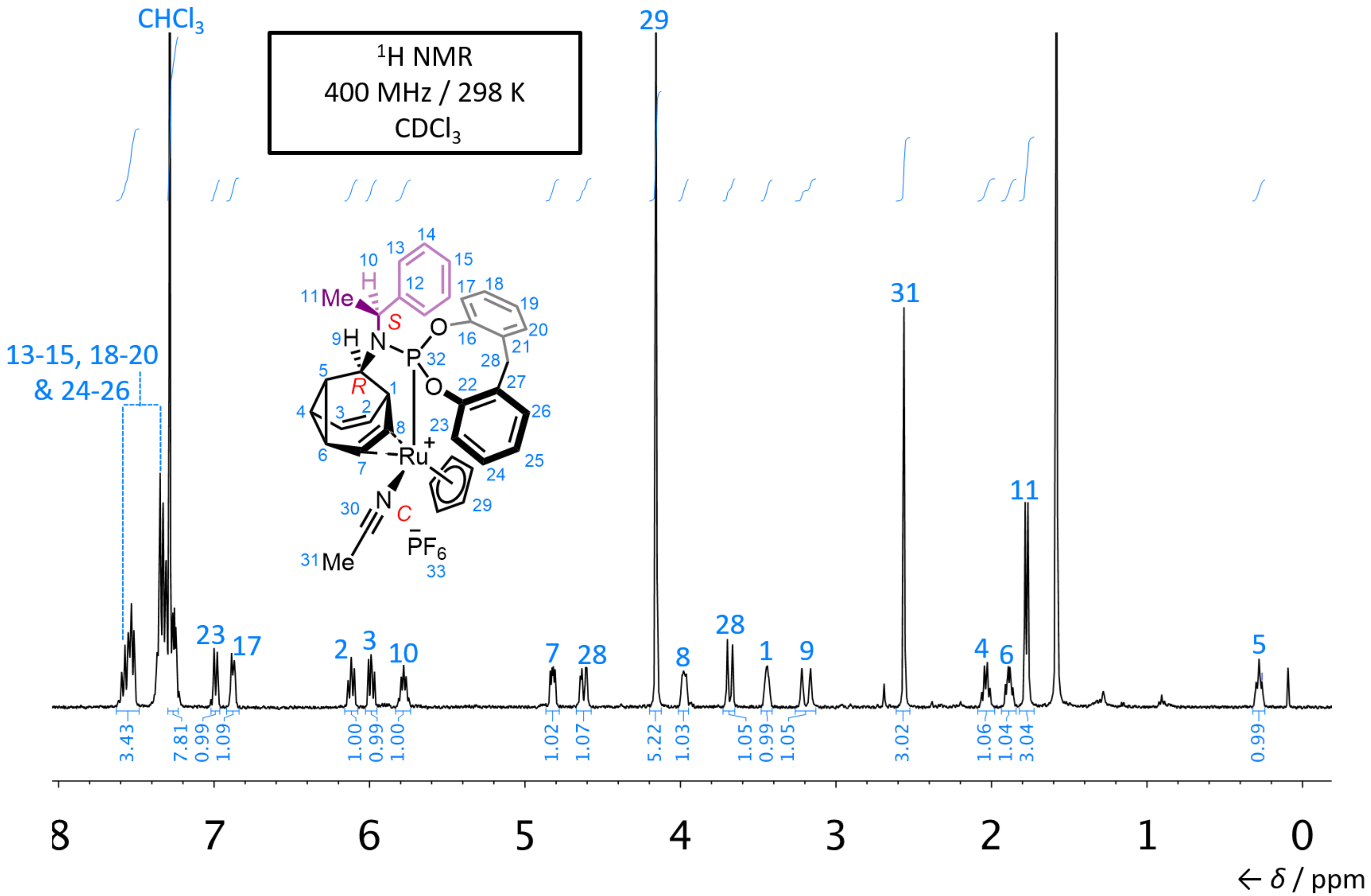

Figure S37. ^{1}H NMR spectrum of (*C*,*R*,*S*)-$\mathbf{L_{BB1}}$RuCp(NCMe)·PF_6.

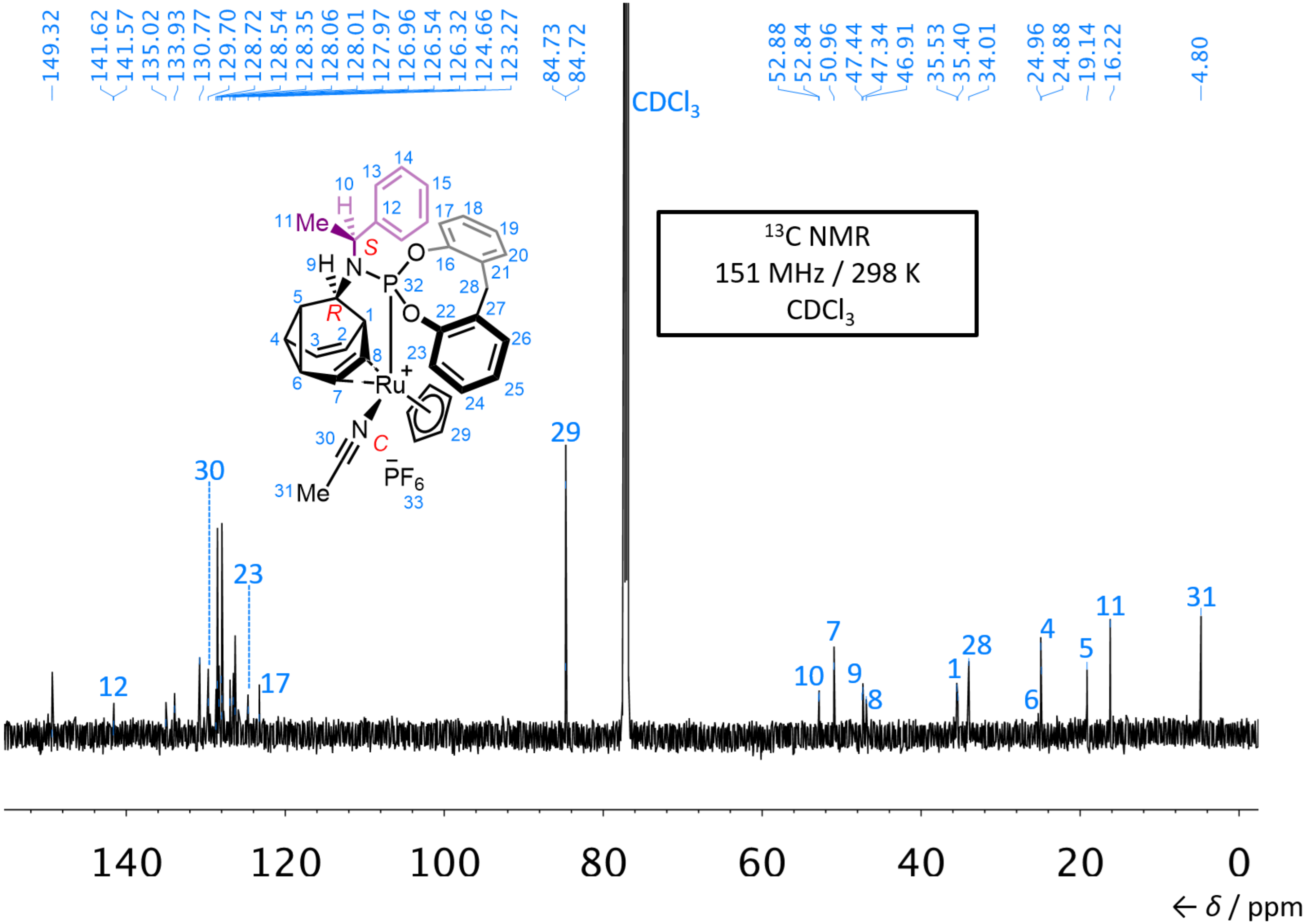

Figure S38. ^{13}C NMR spectrum of (*C*,*R*,*S*)-$\mathbf{L_{BB1}}$RuCp(NCMe)·PF_6.

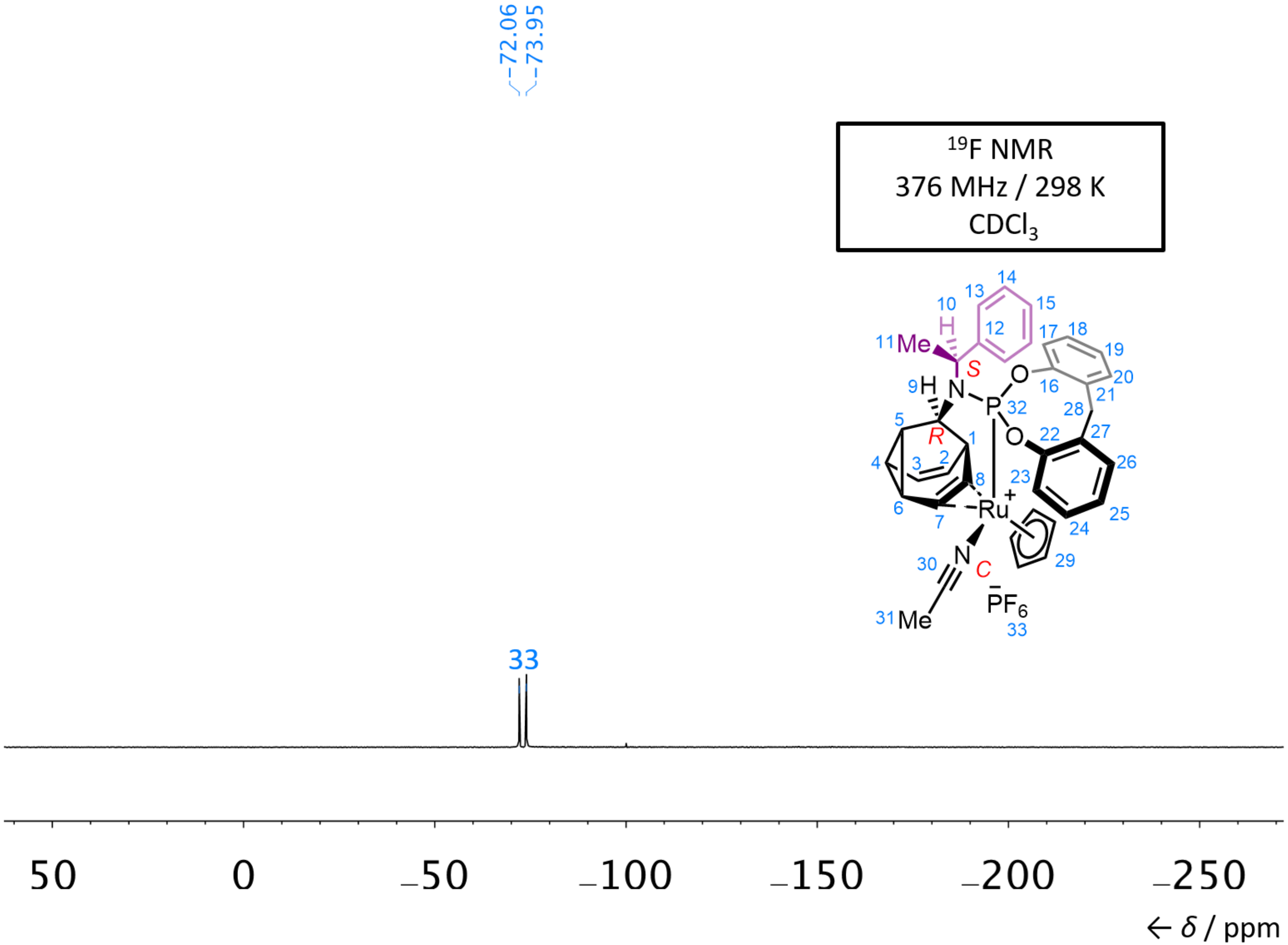

Figure S39. ^{19}F NMR spectrum of (*C*,*R*,*S*)-**L**$_{\mathbf{BB1}}$RuCp(NCMe)·PF_6.

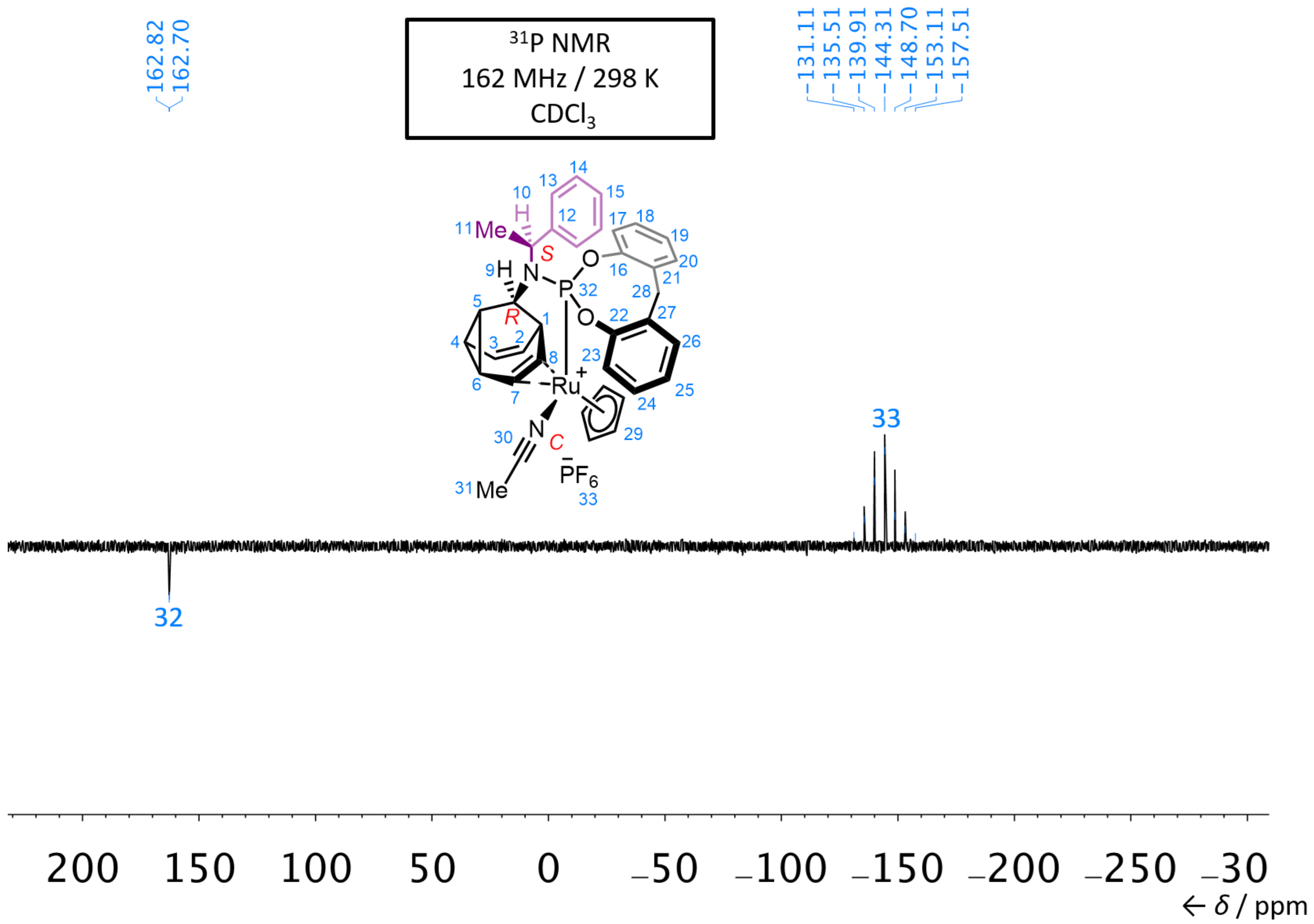

Figure S40. ^{31}P NMR spectrum of (*C*,*R*,*S*)-$\mathbf{L_{BB1}}$RuCp(NCMe)·PF_6.

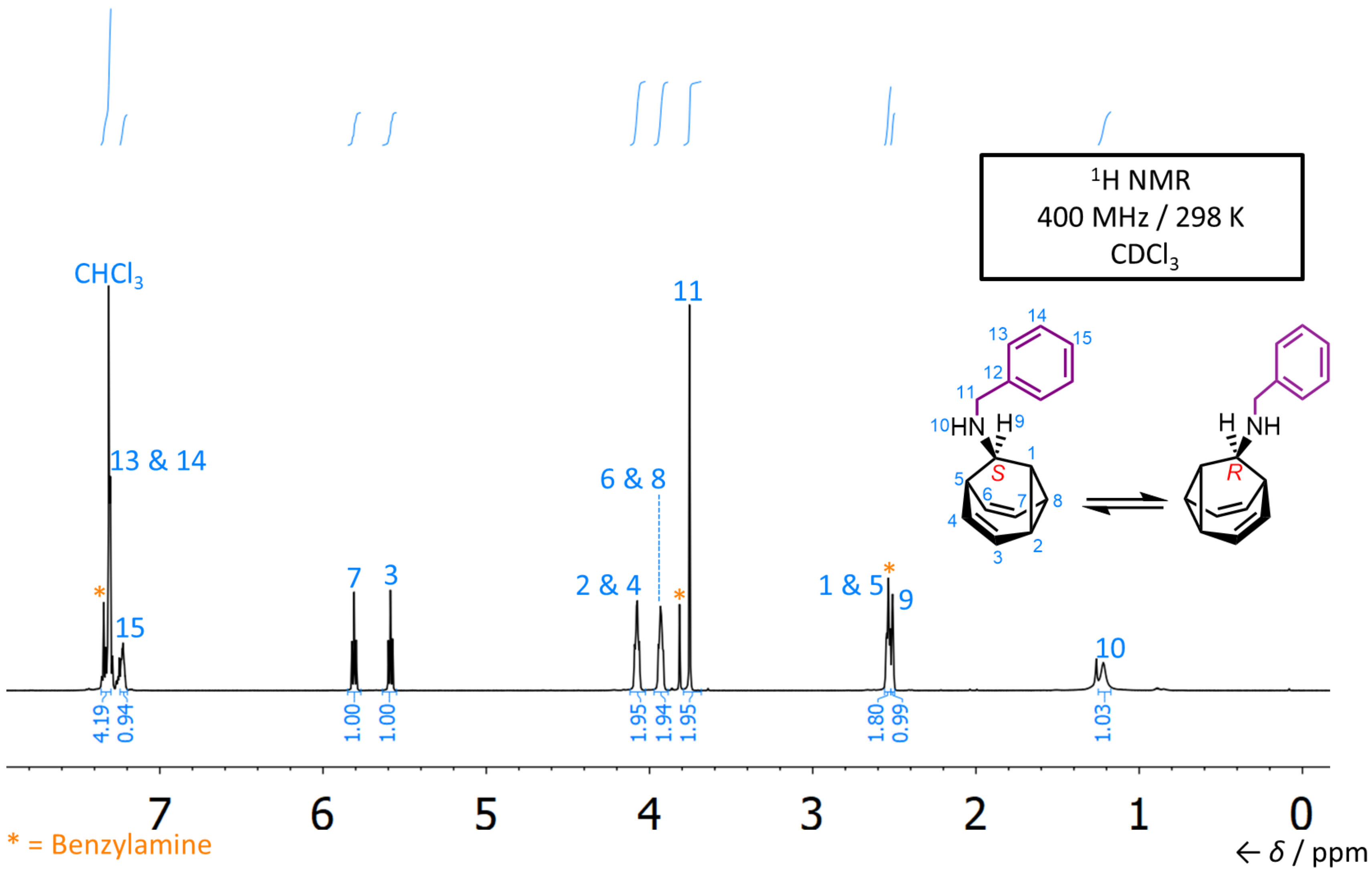

Figure S41. ^{1}H NMR spectrum of (*R*)/(*S*)-**S3**.

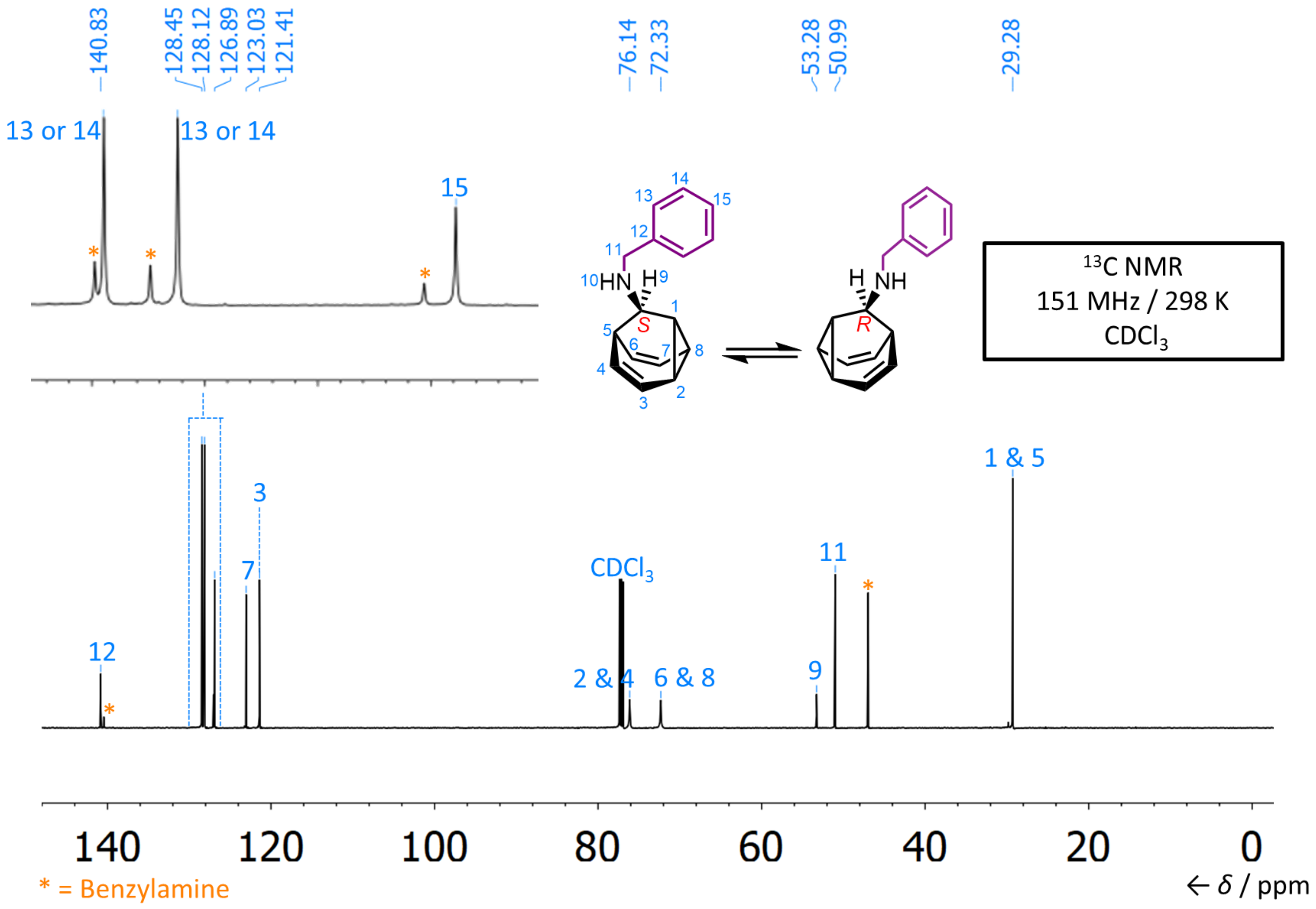

Figure S42. ^{13}C NMR spectrum of (*R*)/(*S*)-**S3**.

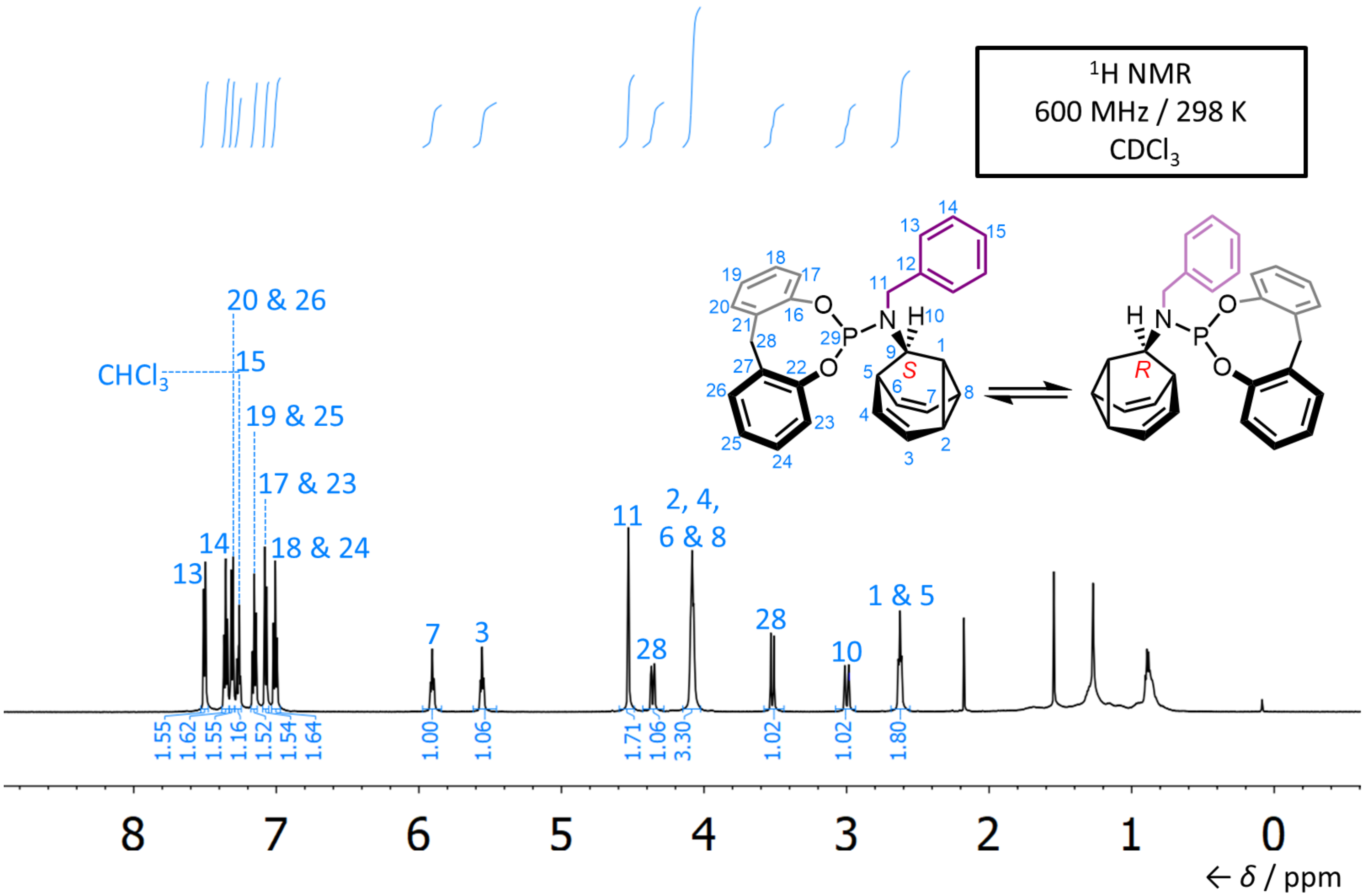

Figure S43. ^{1}H NMR spectrum of (*S*)/(*R*)-**L$_{BB2}$**.

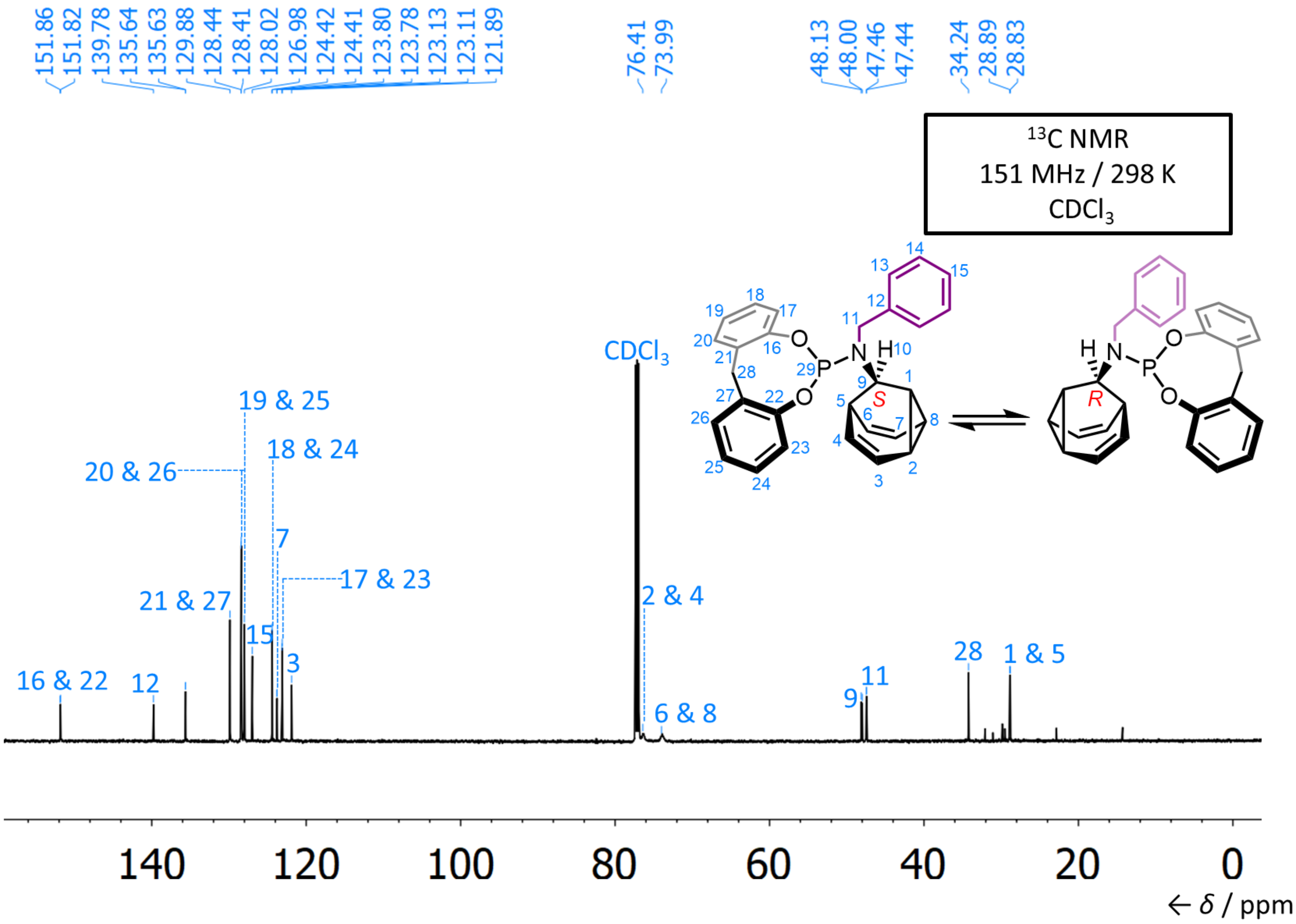

Figure S44. ^{13}C NMR spectrum of (*S*)/(*R*)-$\mathbf{L_{BB2}}$.

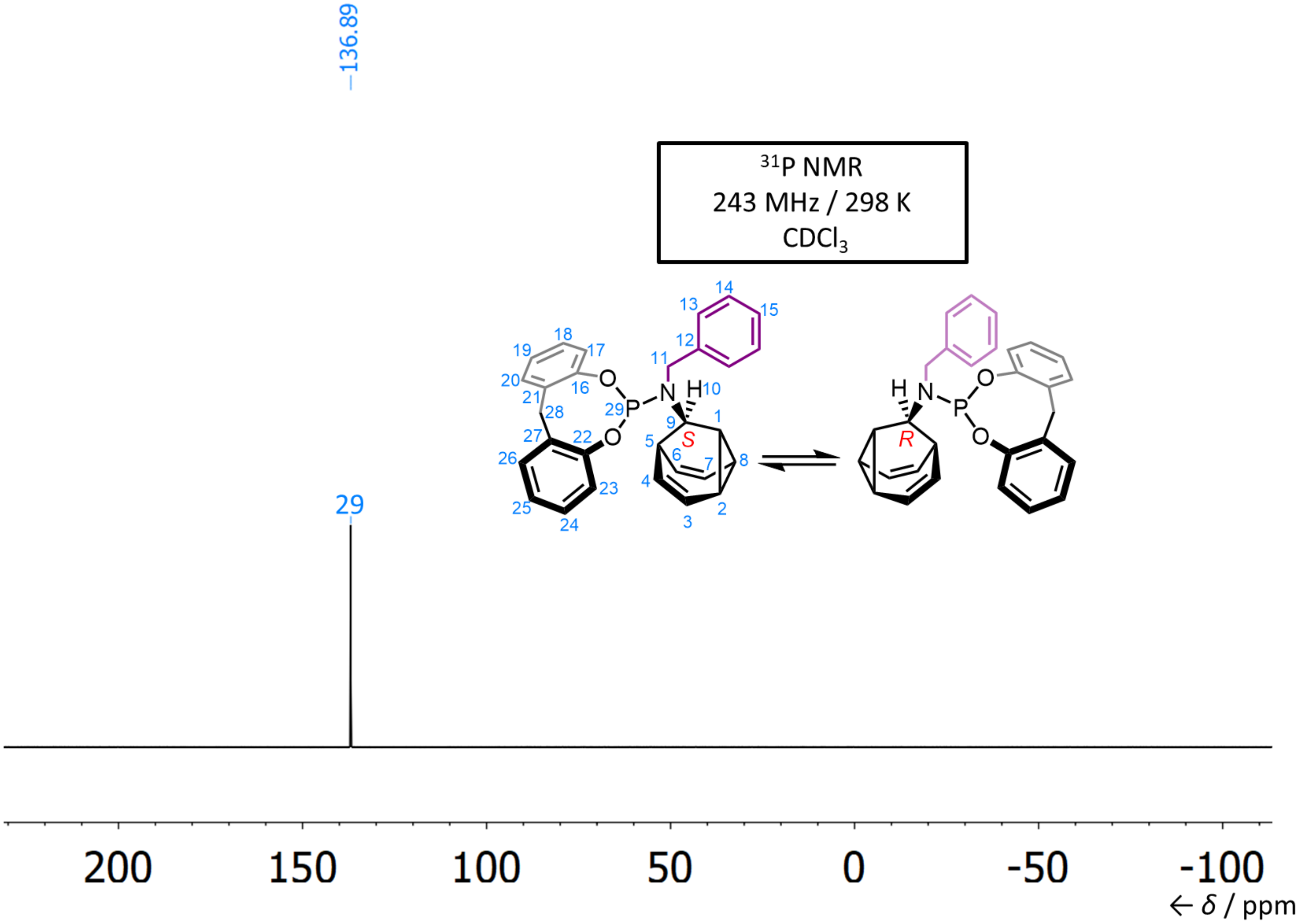

Figure S45. ^{31}P NMR spectrum of (*S*)/(*R*)-$\mathbf{L_{BB2}}$.

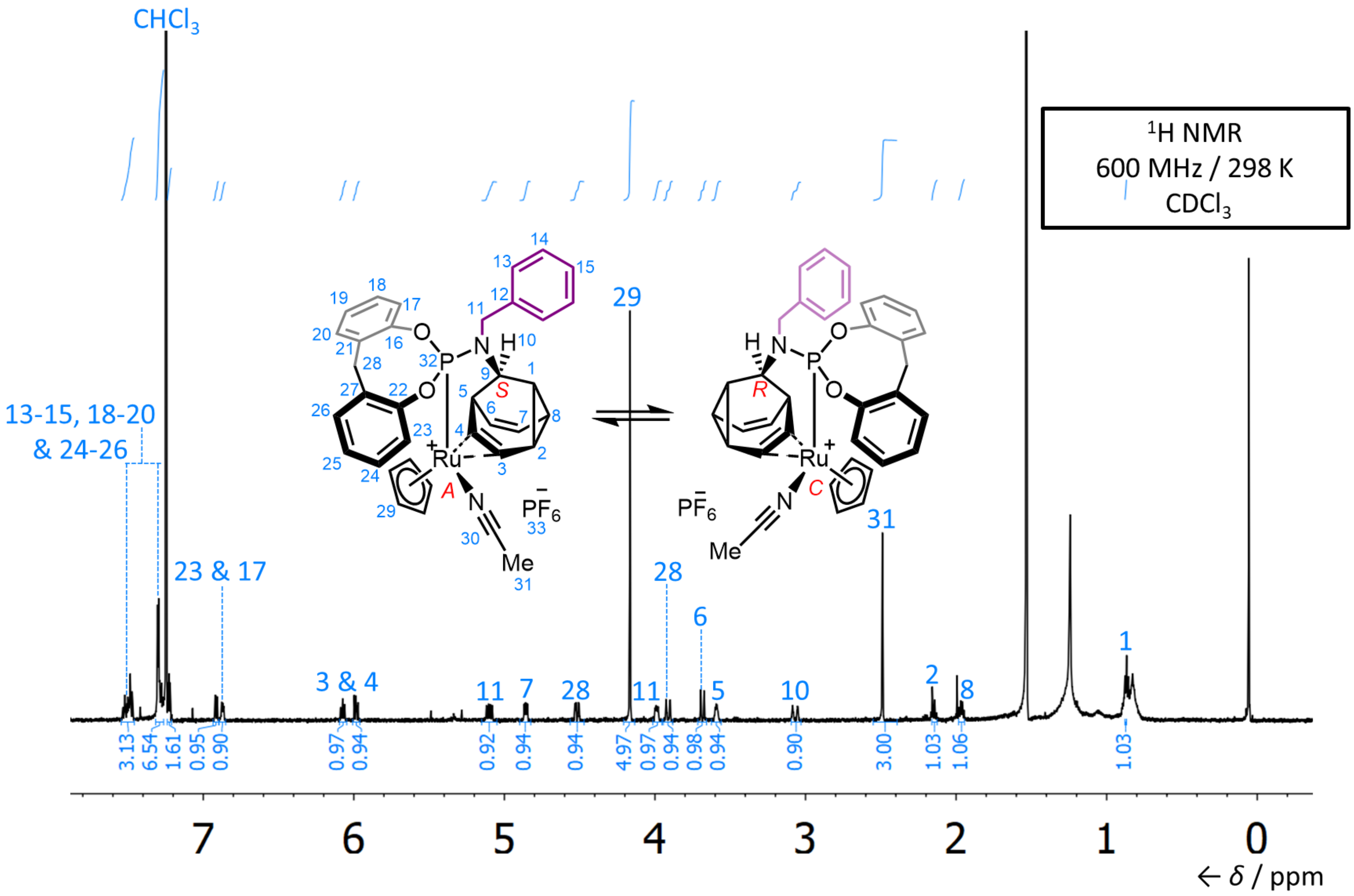

Figure S46. ^{1}H NMR spectrum of (*A*,*S*)/(*C*,*R*)-$\mathbf{L_{BB2}}$RuCp(NCMe)·PF_6.

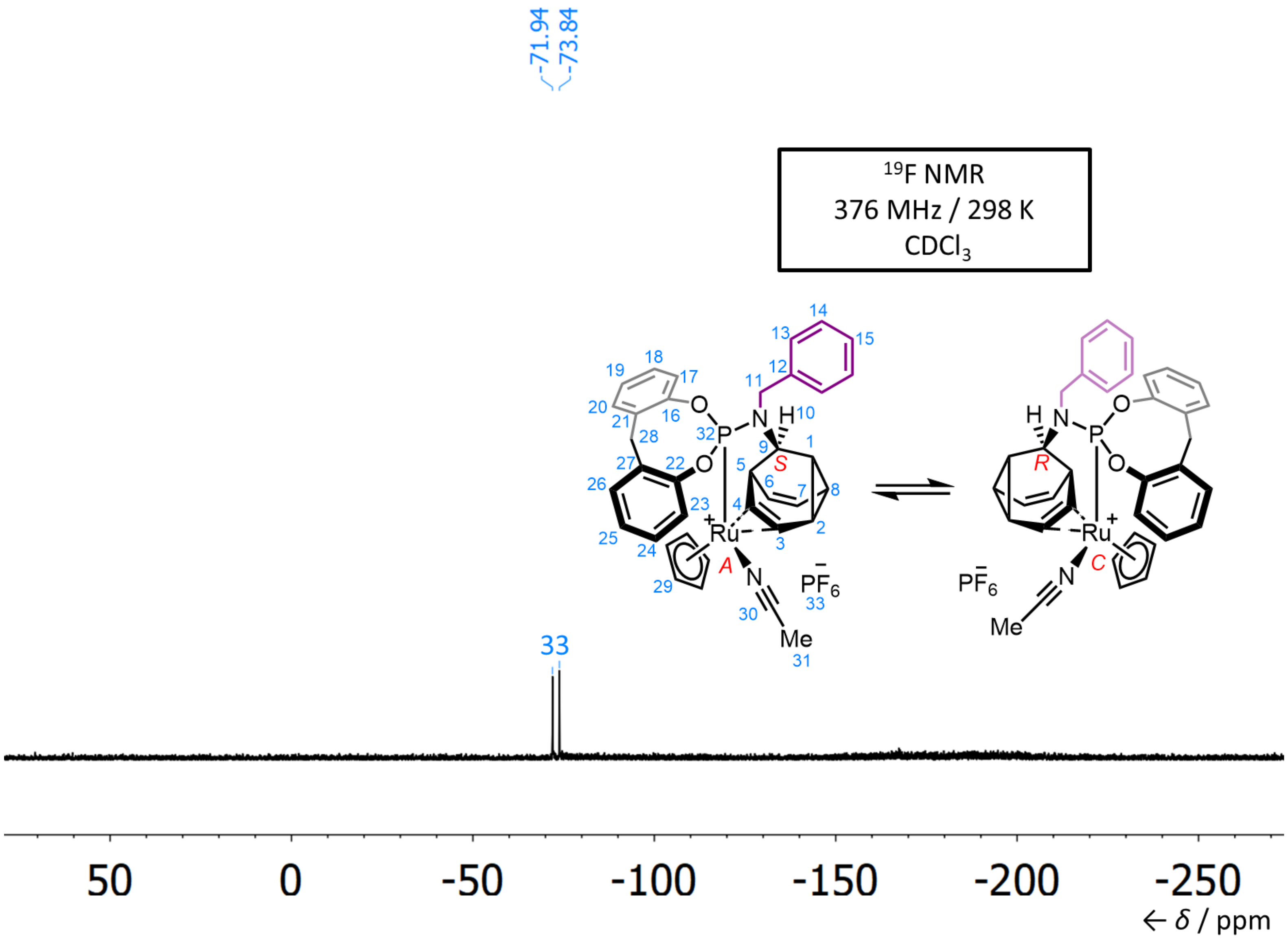

Figure S48. ^{19}F NMR spectrum of (*A*,*S*)/(*C*,*R*)-$\mathbf{L_{BB2}}$RuCp(NCMe)·PF_6.

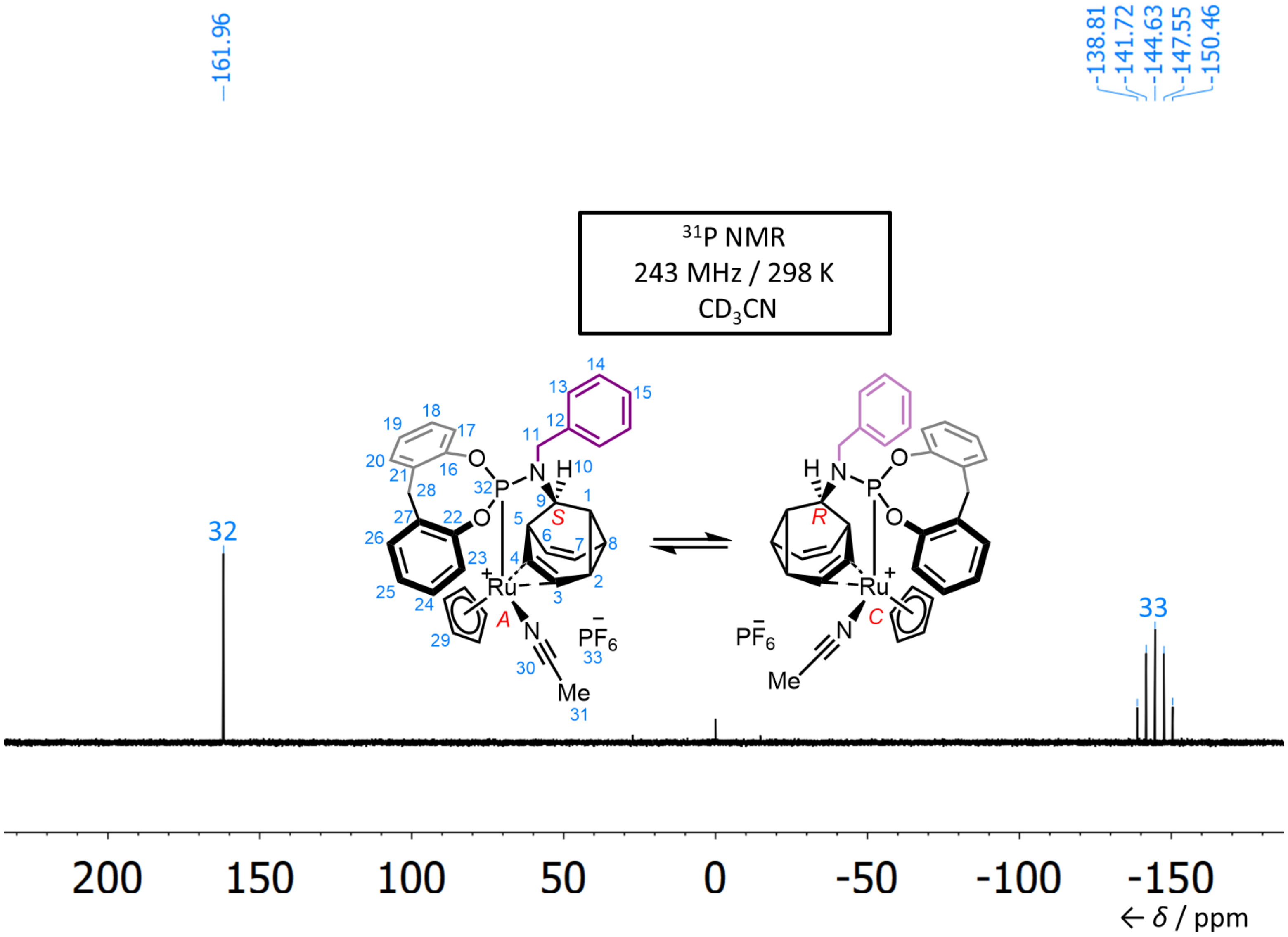

Figure S49. ^{31}P NMR spectrum of (*A*,*S*)/(*C*,*R*)-$\mathbf{L_{BB2}}$RuCp(NCMe)·PF_6.

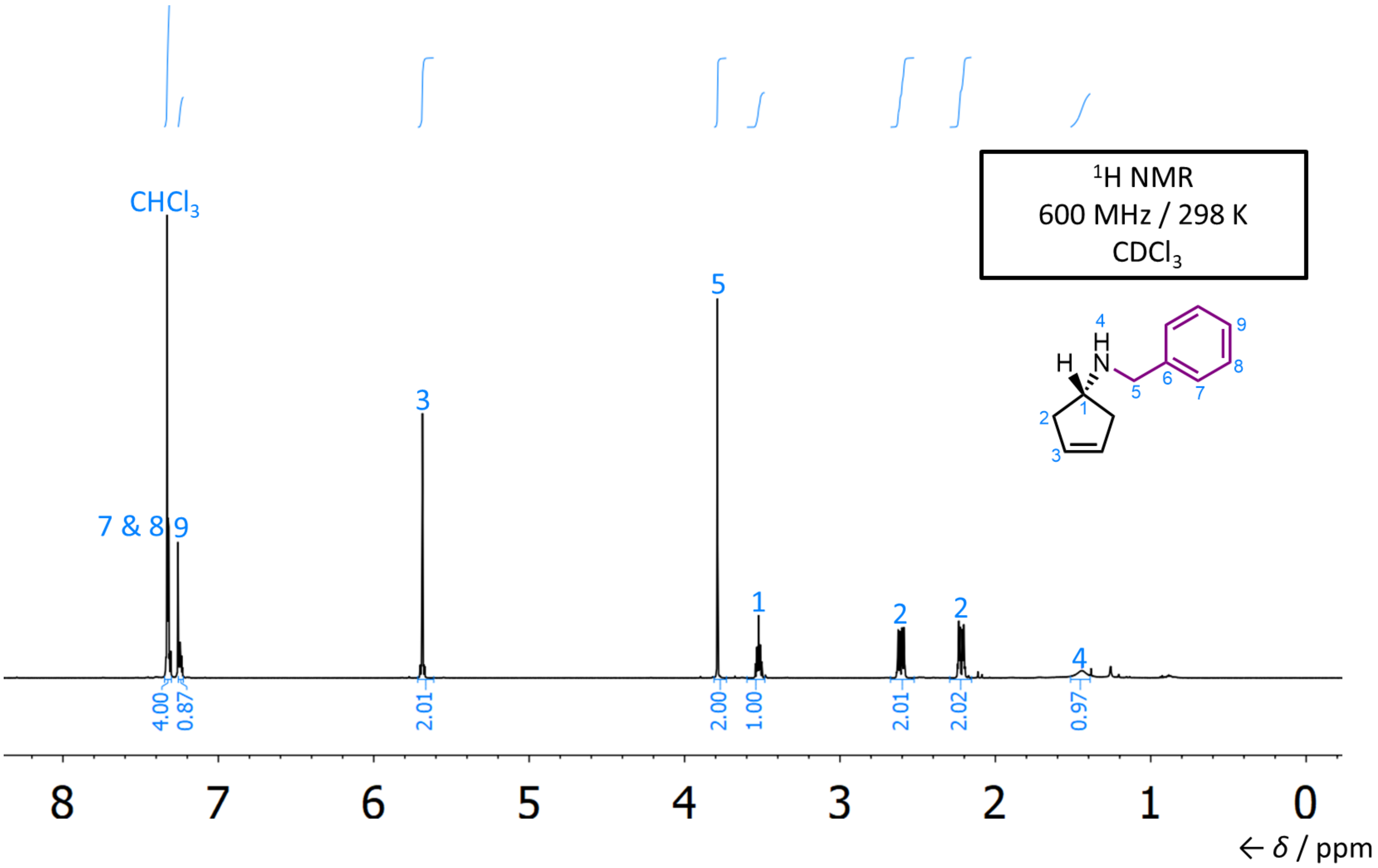

Figure S50. ^{1}H NMR spectrum of **S5**.

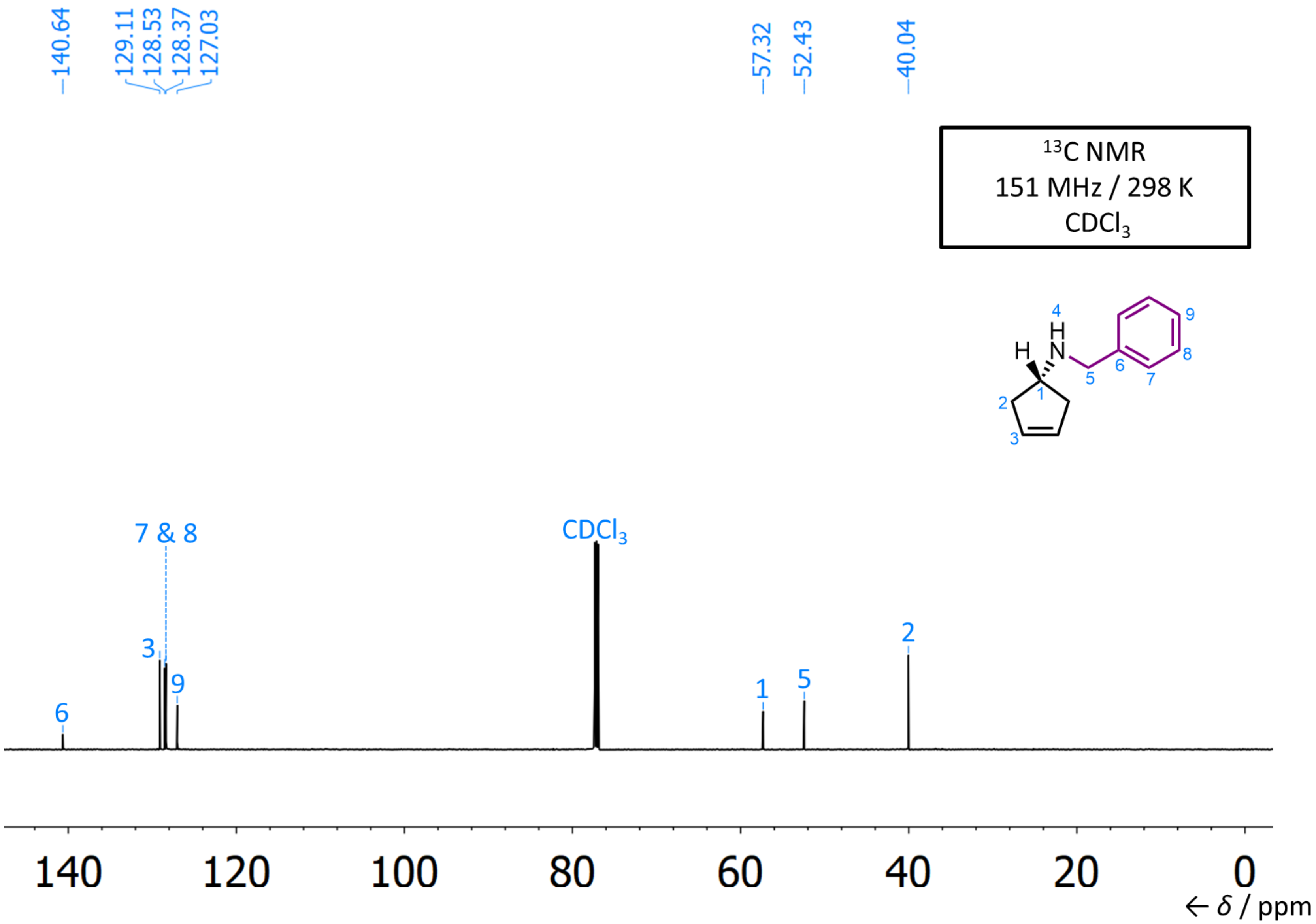

Figure S51. ^{13}C NMR spectrum of **S5**.

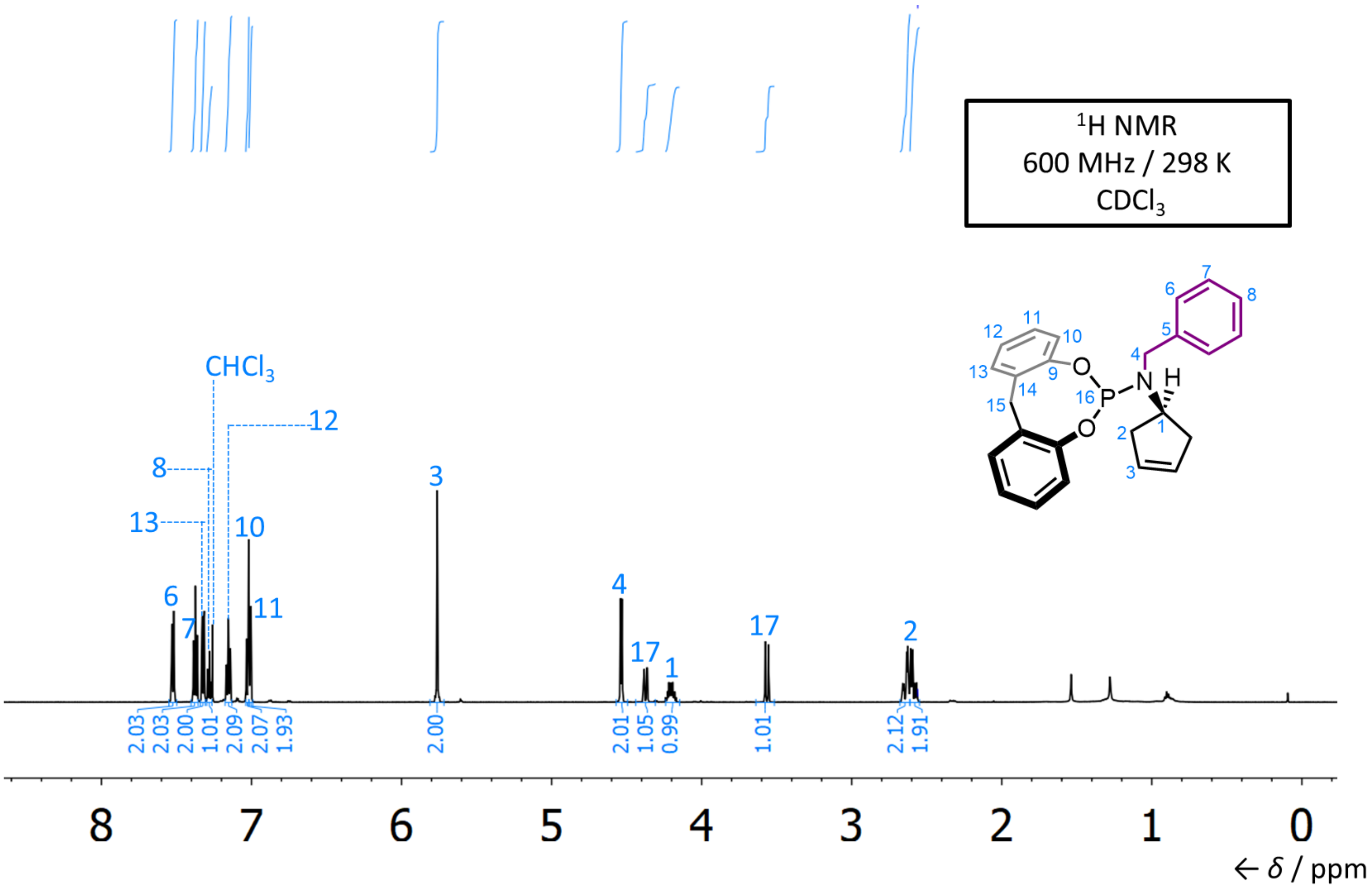

Figure S52. ^{1}H NMR spectrum of $\mathbf{L_{CP}}$.

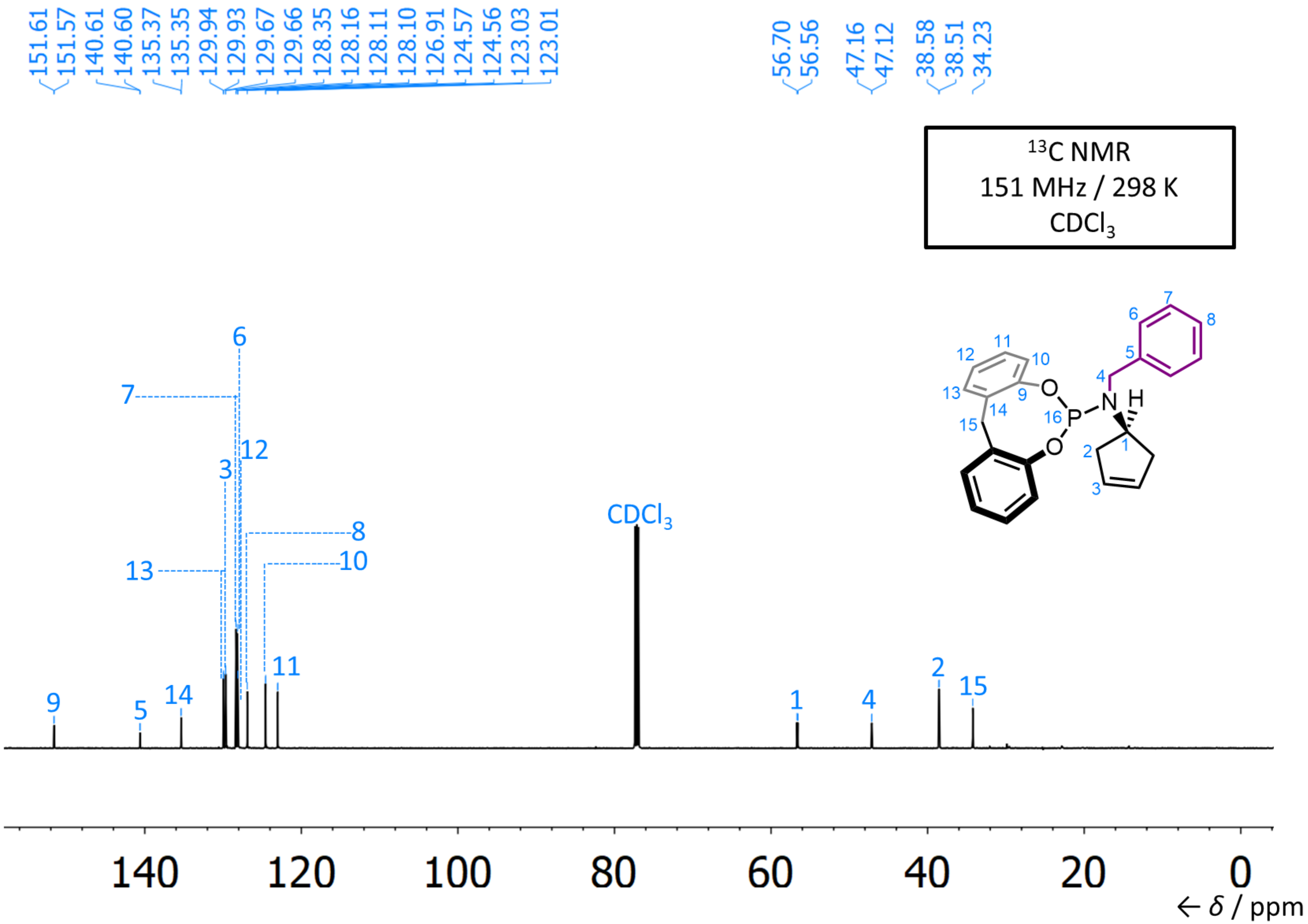

Figure S53. ^{13}C NMR spectrum of $\mathbf{L_{CP}}$.

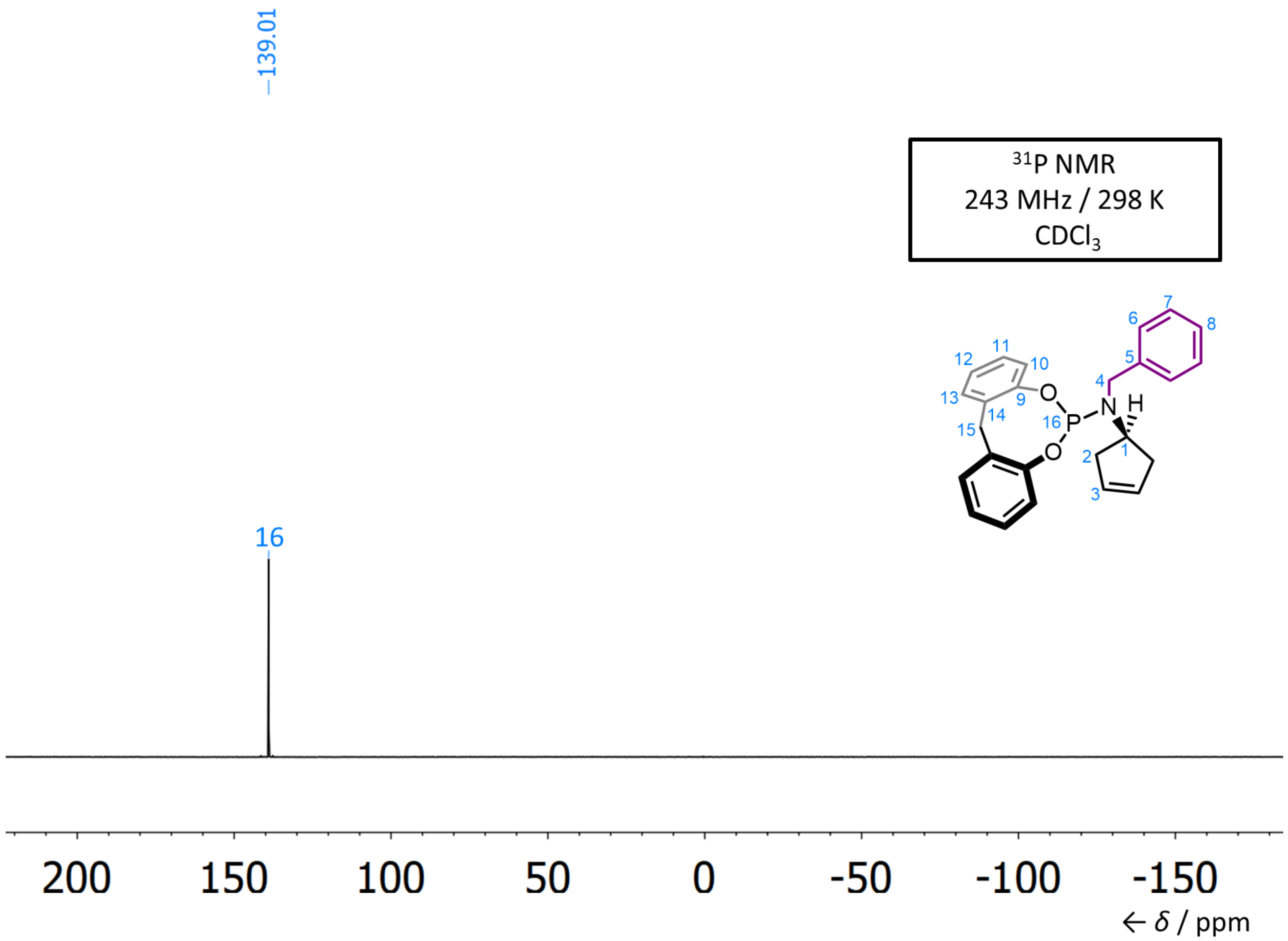

Figure S54. ^{31}P NMR spectrum of $\mathbf{L_{CP}}$.

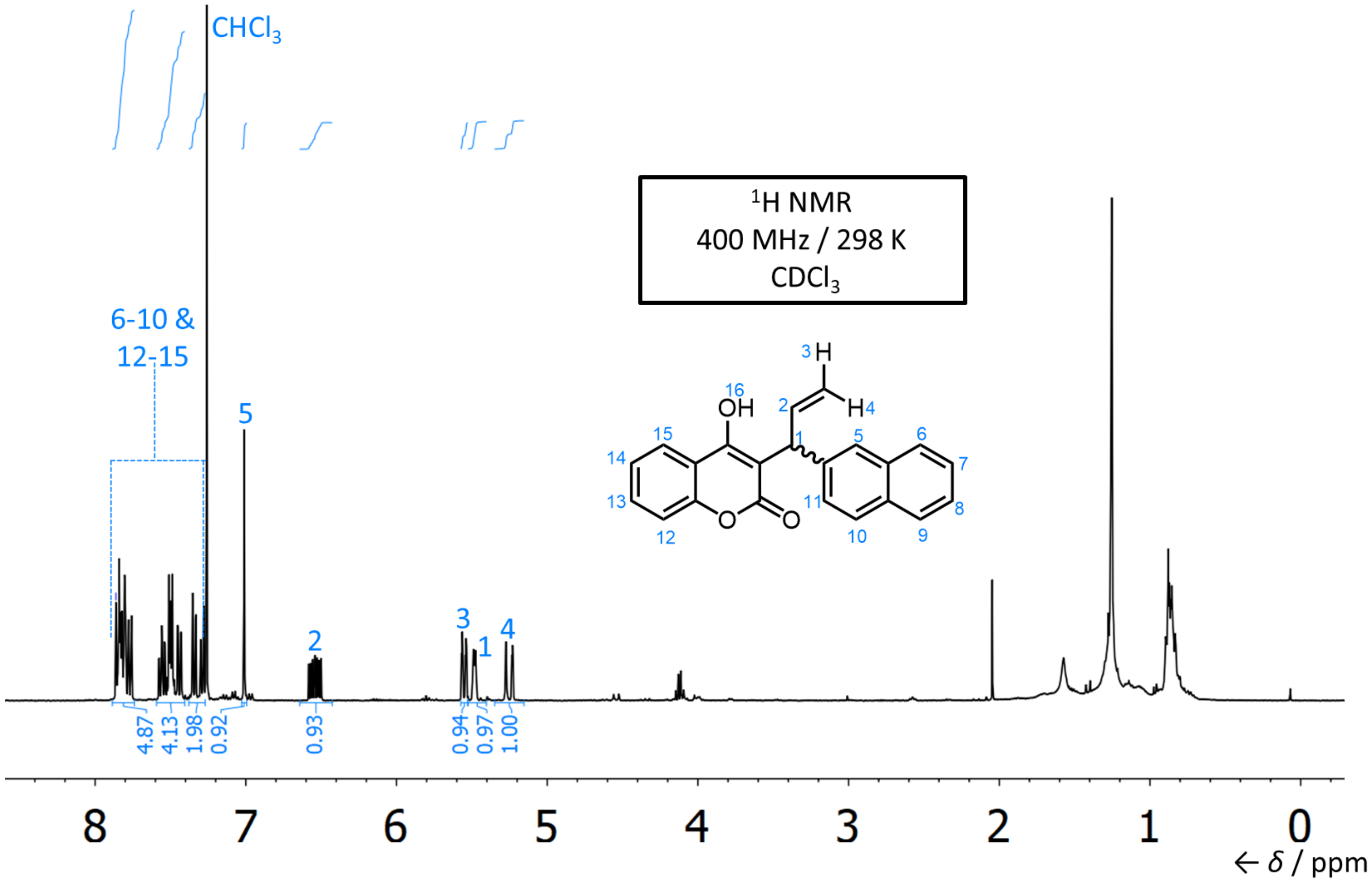

Figure S55. ^{1}H NMR spectrum of **9**.

3.1 Structural Assignment by Two-Dimensional (2D) NMR

In order to distinguish the 'front' of the molecule from the 'back' of the molecule, we used 2D NMR spectroscopy. Briefly, we are able to differentiate which peaks are on the same face of the molecule through analysis of COSY correlations (Figure S56). For example, the olefin which is either 7 or 3 only correlates to one pair of the olefin and cyclopropane positions (2 and 4 or 6 and 8). Therefore, we can confirm that these positions are on the same face.

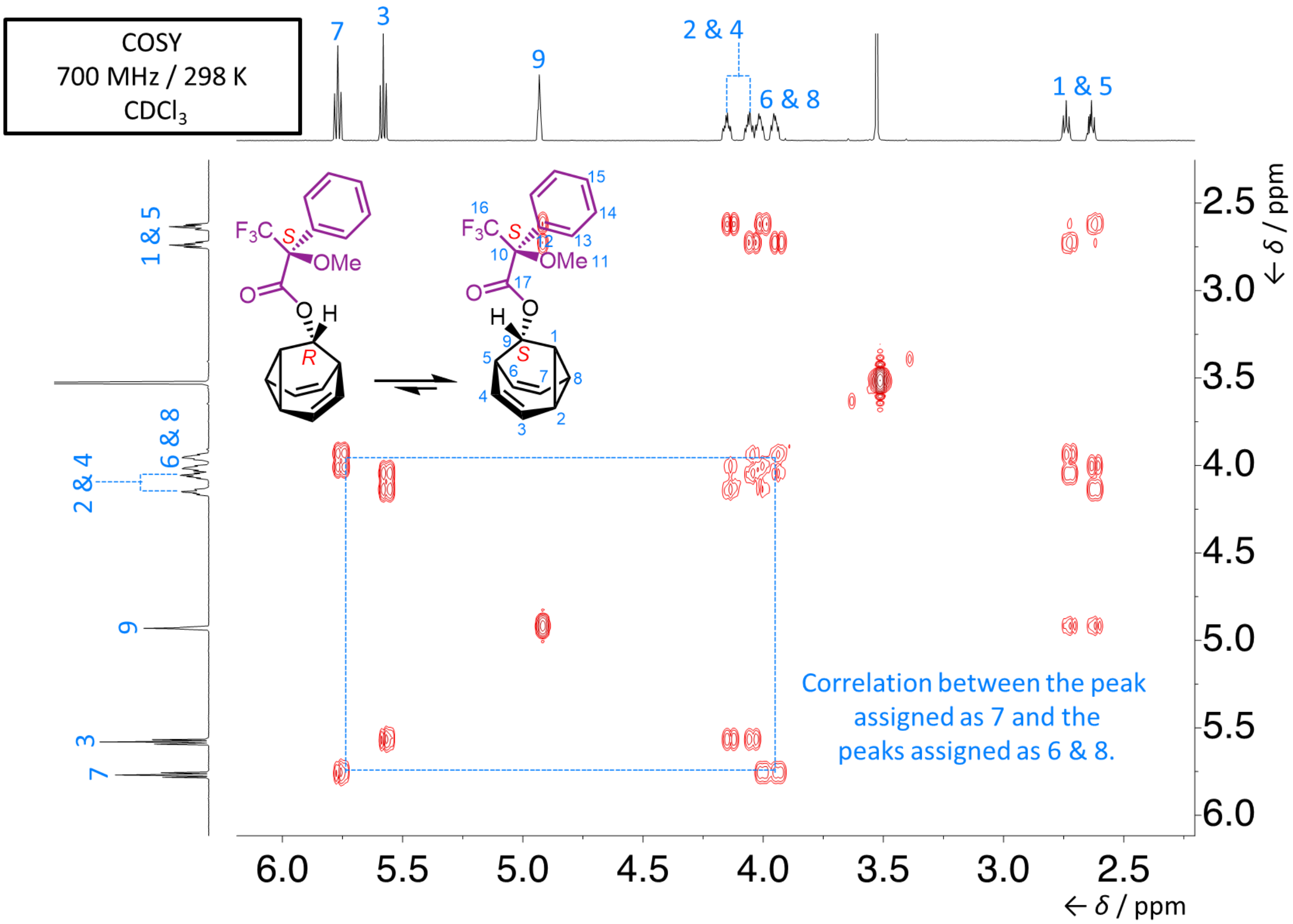

Figure S56. Partial COSY NMR spectrum of (*R*,*S*)/(*S*,*S*)-**2**.

A NOESY correlation between the hydrogen at position 9 to a proton environment on one of the faces of the barbaralyl core allows for determination of the relative configuration of this stereogenic centre. A critical NOESY correlation between the hydrogen at position 9 and a pair of olefin and

cyclopropane positions (2 and 4) confirms that these environments must be on the same face, therefore the front and back of the barbaralyl core can be distinguished (Figure S57). This assignment can be applied to barbaralane (*R*,*R*)/(*S*,*R*)-**2** and (*R*)/(*S*)-**6**. Barbaralanes (*S*,*S*)/(*R*,*S*)-**5** and (*R*,*S*)/(*R*,*R*)-**5** do not exhibit a NOESY correlation. However, we observe the same chemical shift peak pattern as barbaralane (*R*,*S*)/(*S*,*S*)-**2** and, therefore, assign the front and back signals by analogy. 2D NMR spectroscopy does not allow us to differentiate between some of the protons on the barbaralyl core. For example, we cannot differentiate between the positions 2 and 4 of compound (*R*,*S*)/(*S*,*S*)-**2**.

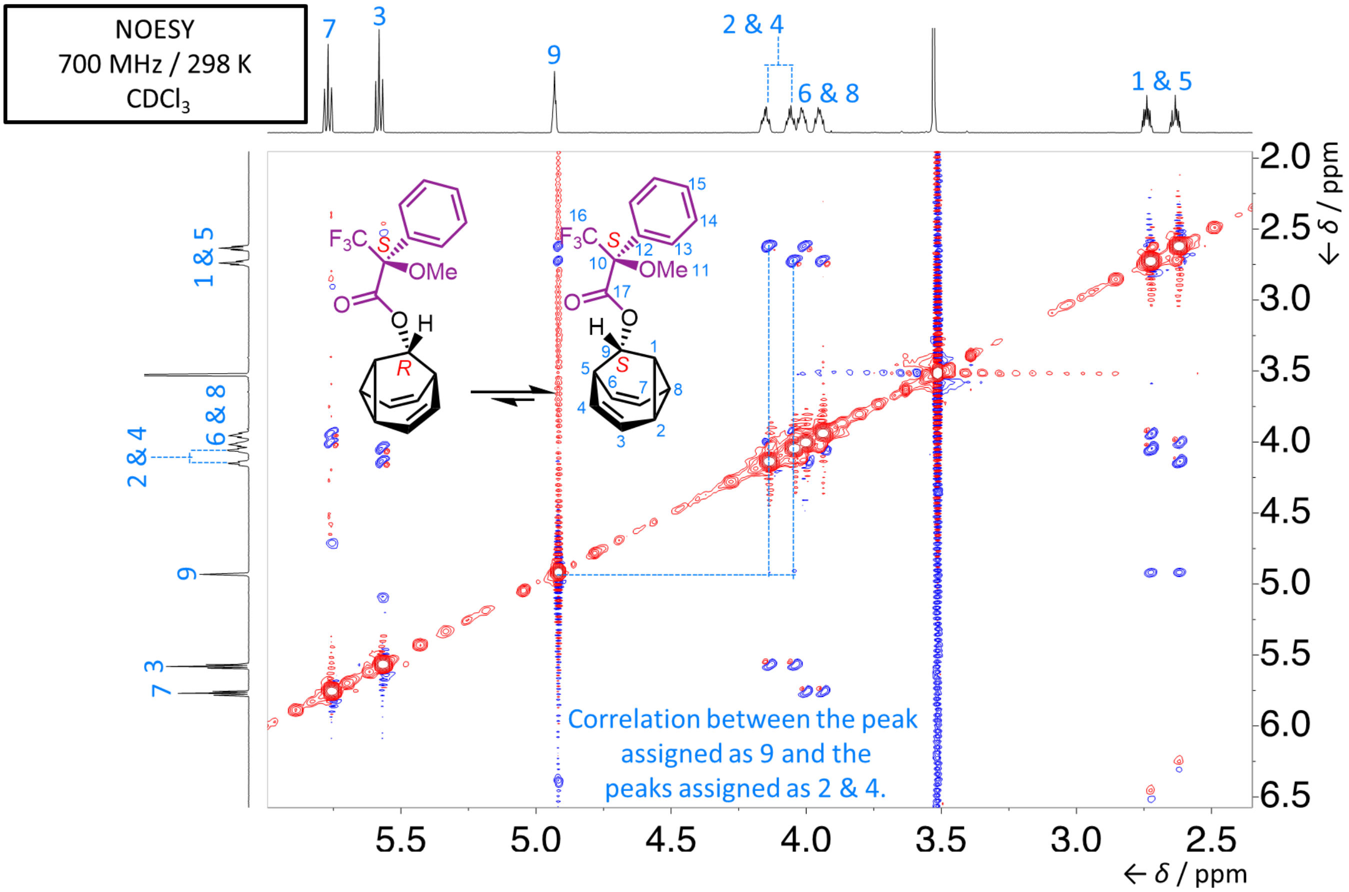

Figure S57. Partial NOESY NMR spectrum of (*R*,*S*)/(*S*,*S*)-**2**.

4. Enantioselective Ion-Pair Catalysis

The iridium-catalysed enantioselective catalysis mechanism proposed in Scheme S5 has been invoked previously[13-15] for allylic substitution reactions. It can be used to understand how a fluxionally chiral ligand (e.g., $\mathbf{L_{BB2}}$) can lead to enhanced enantiomeric excess. After, species **II** is formed and (*R*)-(−)BDHP is transformed into its conjugate base (phosphate anion), an ion pair is generated. The stereochemical equilibrium position of $\mathbf{L_{BB2}}$ ligands present in this ion pair is biased by diastereoselective ion paring ($K \neq 1$) such that **II** becomes enriched in one stereoisomer. This stereochemical information is passed on to the product when nucleophilic attack occurs to give **III**.

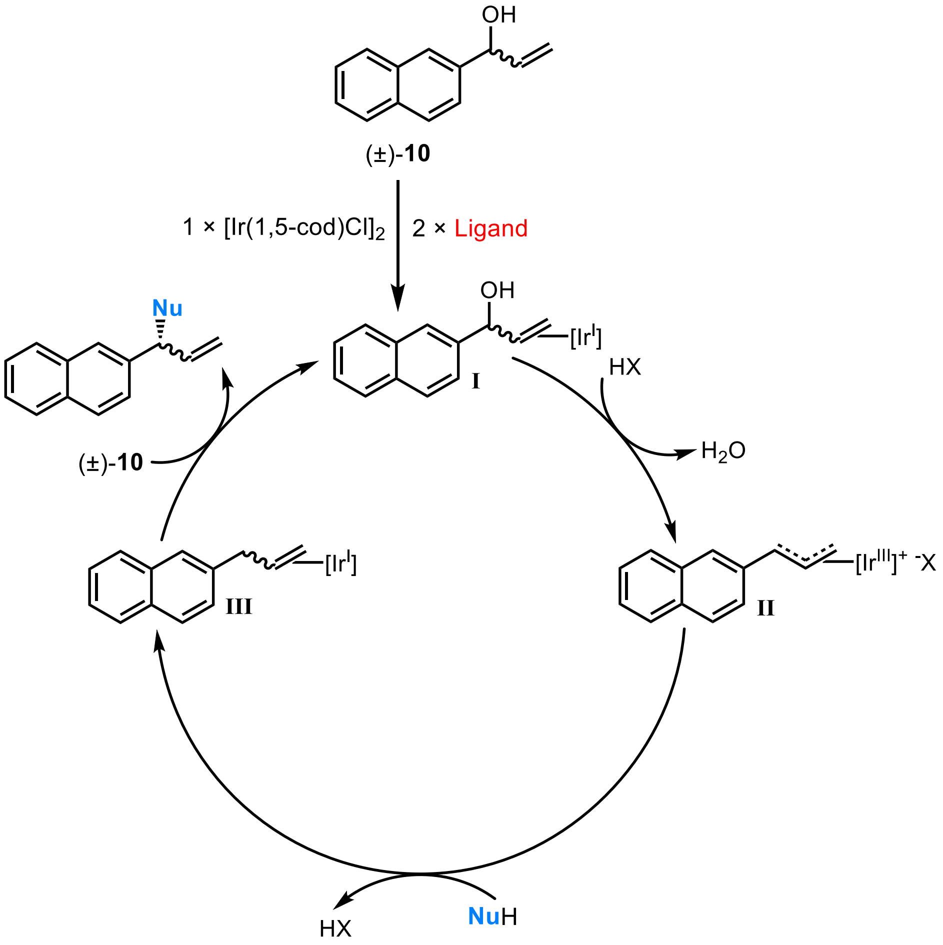

Scheme S5. Proposed mechanism for the iridium-catalysed asymmetric allylic substitution of **9**.

5. High-Performance Liquid Chromatography

Analytical chiral HPLC (Figures S58-S61 and Tables S2-S5) (Diacel ChiralPak AD column, isocratic elution with hexanes:^{i}PrOH (9:1) including 0.1% TFA in the mobile phase, 254 nm detection) was performed to separate the scalemic mixtures of compound **9**.

Entry 1 from Table S1: Ligand = (±)-Carreira Ligand and Acid = (±)-BDHP

The mixture was determined to have an enantiomeric ratio of 50:50 and an enantiomeric excess of 0.

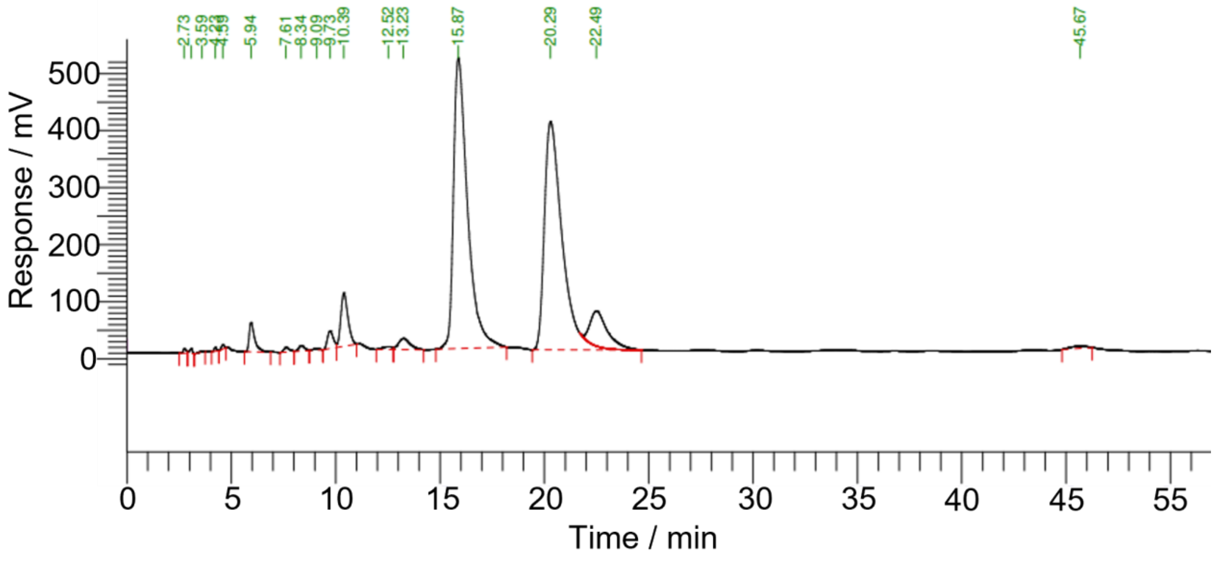

Figure S58. Chromatogram obtained for chiral HPLC of **9** using the (±)-Carreira Ligand and (±)-BDHP.

Table S2. Chiral HPLC report for entry 1 from Table S1.

Time / min	Area / uV*sec	Height / uV	Area / %	Adjusted Amount
15.87	23228622.23	509140.05	41.83	23.8266
20.29	23810883.05	400332.78	41.81	23.8109
**Total:**	47039505.28	909472.83	83.64	47.6375

Entry 2 from Table S1: Ligand = (±)-Carreira Ligand and Acid = (*R*)-(−)BDHP

The mixture was determined to have an enantiomeric ratio of 51:49 and an enantiomeric excess of 2.

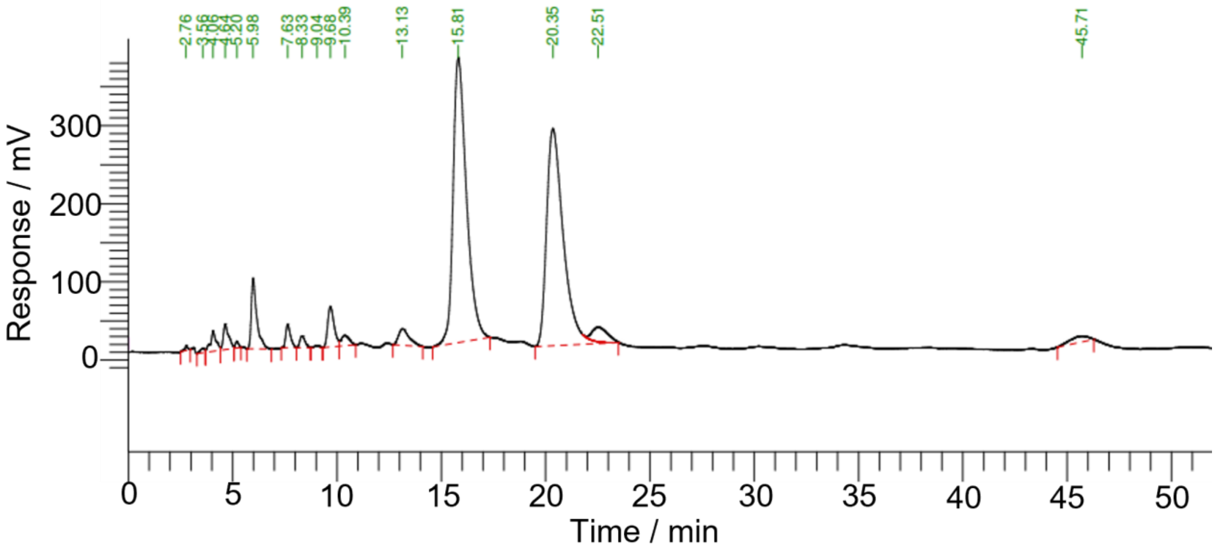

Figure S59. Chromatogram obtained for chiral HPLC of **9** using the (±)-Carreira Ligand and (*R*)-(−)BDHP.

Table S3. Chiral HPLC report for entry 2 from Table S1.

Time / min	Area / uV*sec	Height / uV	Area / %	Adjusted Amount
15.81	16248760.28	364866.91	41.40	16.2488
20.35	15549252.24	278125.67	39.61	15.5493
**Total:**	31798012.52	642992.58	81.01	47.6375

Entry 3 from Table S1: Ligand = $\mathbf{L_{BB2}}$ and Acid = (*R*)-(−)BDHP

The mixture was determined to have an enantiomeric ratio of 65:35 and an enantiomeric excess of 30.

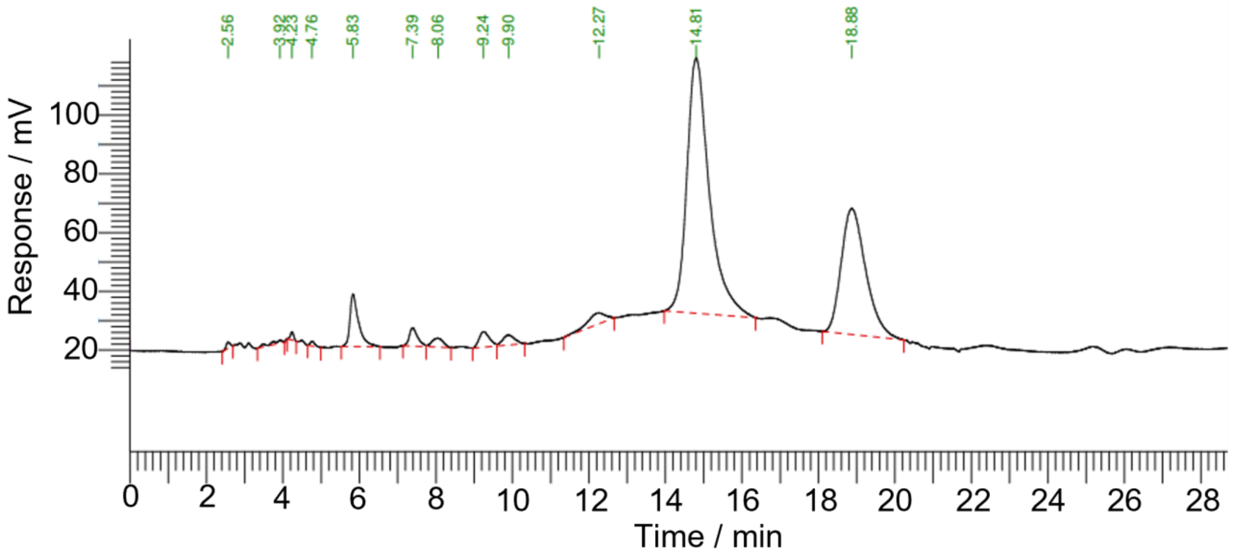

Figure S60. Chromatogram obtained for chiral HPLC of **9** using the $\mathbf{L_{BB2}}$ and (*R*)-(−)BDHP.

Table S4. Chiral HPLC report for entry 3 from Table S1.

Time / min	Area / uV*sec	Height / uV	Area / %	Adjusted Amount
14.81	3559897.60	86960.05	56.56	3.5599
18.88	1917505.45	42925.87	30.47	1.9175
**Total:**	5477403.05	129885.92	87.03	5.4774

Entry 4 from Table S1: Ligand = $\mathbf{L_{CP}}$ and Acid = (*R*)-(−)BDHP

The mixture was determined to have an enantiomeric ratio of 51:49 and an enantiomeric excess of 2.

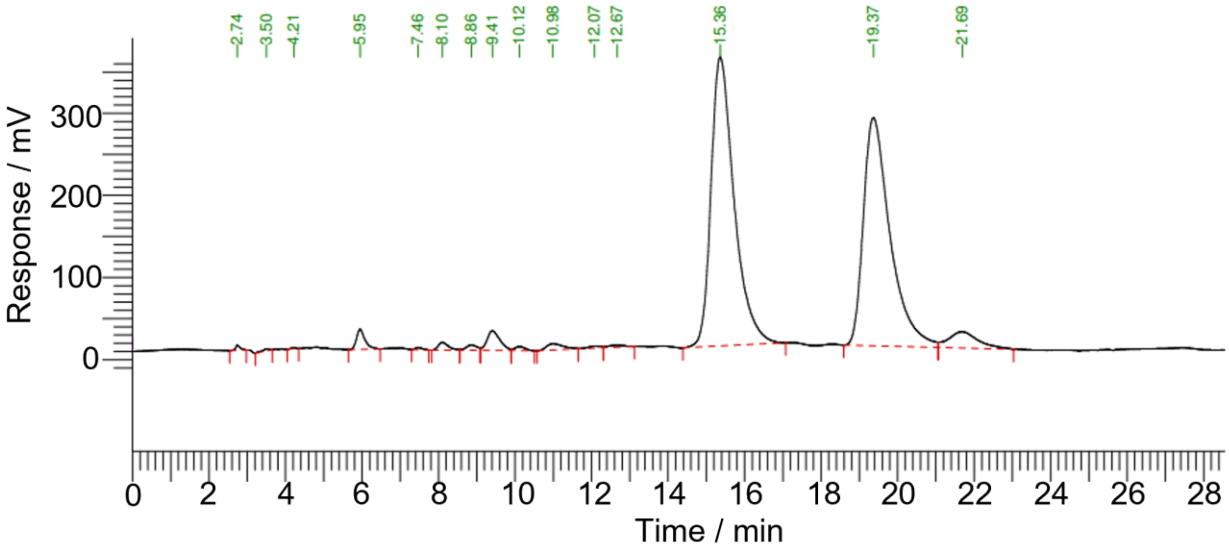

Figure S61. Chromatogram obtained for chiral HPLC of **9** using the $\mathbf{L_{CP}}$ and (*R*)-(−)BDHP.

Table S5. Chiral HPLC report for entry 4 from Table S1.

Time / min	Area / uV*sec	Height / uV	Area / %	Adjusted Amount
15.36	14774670.30	351671.64	47.39	14.7747
19.37	13481959.73	277923.45	43.24	13.4820
**Total:**	28256630.03	629595.09	90.63	28.2567

6. Additional NMR Spectroscopic Measurements

6.1 *In Situ* Formation of Diazinane 8

Upon subjecting *(R)*/(*S*)-**6** to cycloaddition reaction conditions, compound **7** is obtained. The ^{1}H NMR spectra shows (Figure S62) that this reaction halts the Cope rearrangement observed in *(R)*/(*S*)-**6** whilst also symmetrising the structure overall by forming a second cyclopropyl ring. The fluxional cage is easily regenerated in a two-step transformation through compound **8** (evidence in the crude NMR), which undergoes cycloreversion upon oxidation with $CuCl_2$. A ^{1}H NMR spectrum of the crude mixture of **8** displays characteristic proton resonances of the diazinane C-H (labelled as positions 1 and 2) and N-H environments (labelled as positions 3 and 4).

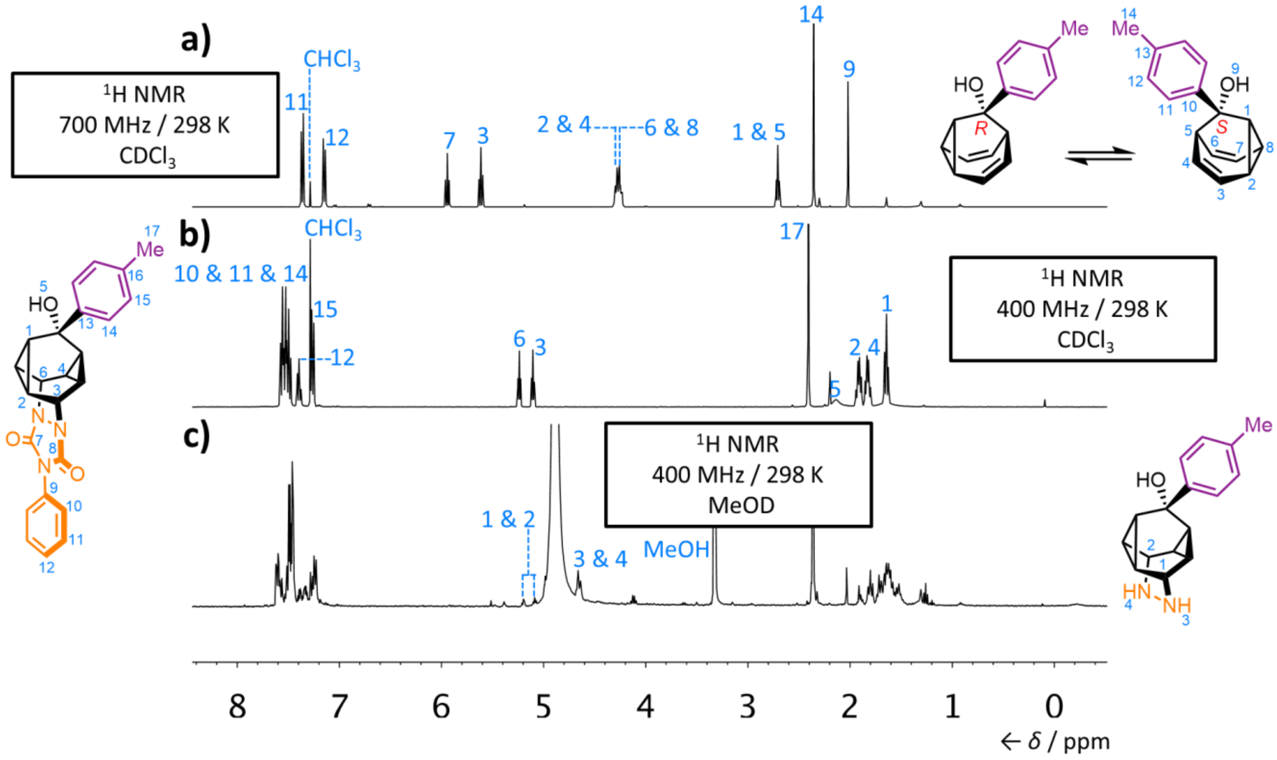

Figure S62. Partial ^{1}H NMR spectra of (a) *(R)*/(*S*)-**6**, (b) **7** and (c) **8**. The spectra show the stopping and re-starting of the Cope rearrangement observed in *(R)*/(*S*)-**6**.

6.2 Desymmetrisation of the Barbaralyl Core

Compound **3** is an achiral molecule as a result of an internal mirror plane (σ_v'') (in the plane of the carbonyl bridge), however another orthogonal mirror plane (σ_v') is effectively present on the timescale of NMR measurements as a result of the rapid dynamics of the barbaralane core. Due to this apparent mirror plane (σ_v'), the exchanging cyclopropane and olefinic environments appear as a broad singlet. In contrast, a 9-BB-type compound (e.g., *(R)*/(*S*)-**1**) lacks the internal mirror plane (σ_v'') – however, the orthogonal mirror plane (σ_v') arising from rapid Cope rearrangement is retained. The removal of the σ_v'' mirror plane results in the desymmetrisation of the molecule and subsequently the cyclopropane and olefinic environments split into two sets of two inequivalent peaks remaining in the fast-exchange regime (see Figure 3b in the manuscript and Figure S63).

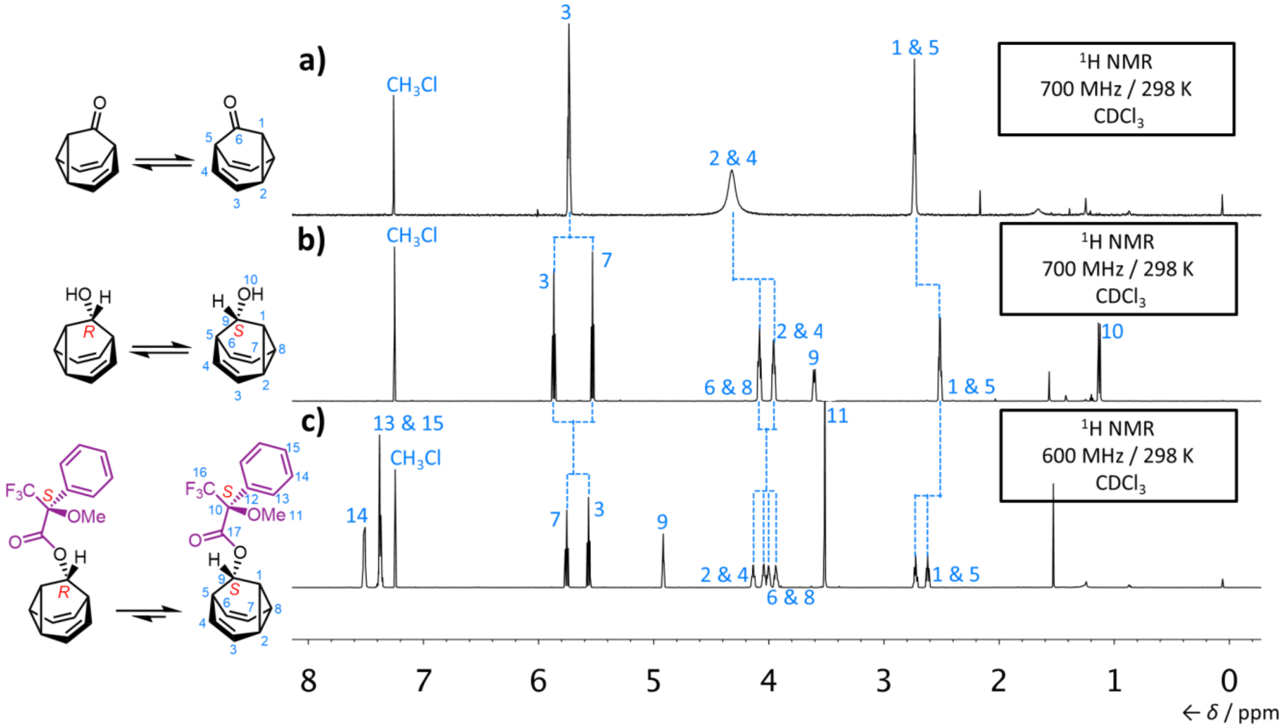

Figure S63. ^{1}H NMR spectra of (a) **3**, (b) *(R)*/(*S*)-**1** and (c) (*R*,*S*)/(*S*,*S*)-**2**. The spectra show the sequential desymmetrisation of the barbaralyl core with environments splitting as they become magnetically equivalent.

Covalently linking a chiral tether to the barbaralane core (as observed with (*R*,*S*)/(*S*,*S*)-**2**) removes the remaining mirror plane (σ'_v) and entirely desymmetrises the core so that every proton on the barbaralane core has its own unique chemical environment. For example, the broad singlet present in Figure S63a labelled as position 2 changes into four distinctly split peaks in Figure S63b. Although, desymmetrisation takes place, the chiral tether molecules still exist in solution as a mixture of interconverting nondegenerate diastereoisomers. Since *(R)*/(*S*)-**1** in the solution state interconverts between two degenerate isomers, the equilibrium constant, *K*, is 1.0 and therefore the averaged peaks are exactly halfway between the two individual structures in exchange.[8,16] For example, the proton signal corresponding to position 2 and 4 is exactly halfway between the signals for 2 and 4 if two structures were fixed in either state. Similarly, the positions of the four peaks (2, 4, 6, and 8) of (*R*,*S*)/(*S*,*S*)-**2** (Figure S63c) represent an average of the interconverting pair of stereoisomers, but weighted by the equilibrium distribution of isomers (where $K \neq 1$). The same type of symmetry breaking is observed for (*R*,*R*)/(*S*,*R*)-**2**, (*S*,*S*)/(*R*,*S*)-**5**, (*R*,*S*)/(*R*,*R*)-**5**, $\mathbf{L_{BB1}}$, and complexes (*R*,*S*)/(*S*,*S*)-$\mathbf{L_{BB1}}$AuCl, (*A*,*S*,*S*)/(*C*,*R*,*S*)-$\mathbf{L_{BB1}}$$PdCl_2$ and (*C*,*R*,*S*)-$\mathbf{L_{BB1}}$RuCp(NCMe)·PF_6.

6.3 Variable-Temperature (VT) NMR Spectroscopy of (*R*,*S*)/(*S*,*S*)-2

In order to probe the equilibrium further, we recorded solution-phase ^{1}H and ^{13}C NMR spectra of covalently modified barbaralane (*R*,*S*)/(*S*,*S*)-**2** at low temperatures ranging from 296 K to 149 K (Figures S64 and S65). At room temperature, the diastereomers appear to exist in a ratio which is almost 1:1 of interconverting configurations, owing to the close proximity of the olefinic and cyclopropane chemical shifts (the proton environments labelled as 2, 4, 6 and 8). Cooling below the coalescence temperature separates these environments into the slow-exchange regime, so that chemical shifts are closer to those expected for a divinyl cyclopropane and for a *cis*-dialkylolefin. Analysis of the chemical shift variation as a function of temperature could have, in principle, allowed quantitative evaluation of the kinetic and thermodynamic parameters determining the dynamic chirality of the carbon stereogenic centre of (*R*,*S*)/(*S*,*S*)-**2** if both isomers could be observed in slow exchange. But in the slow-exchange regime the equilibrium was deemed to shift almost entirely in preference of the thermodynamically favoured diastereoisomer (*S*,*S*)-**2** (Figure S64) on account of the shift in the Boltzmann distribution at low temperature. This result was confirmed by DFT calculations (see *In Silico* Modelling, Section 9). Diastereoisomers (*R*,*S*)/(*S*,*S*)-**2** are computed to have an energy difference of 6.8 $kJ \cdot mol^{-1}$ between their minimum energy geometries, with the lower energy isomer (*S*,*S*)-**2** being thermodynamically favoured. Overall, introduction of a covalently bound chiral tether of fixed absolute configuration allows the solution-state equilibrium to shift slightly in favour of the thermodynamically favoured configuration at room temperature. Upon cooling the solution-state equilibrium is shown to be dominated by the thermodynamically preferred diastereoisomer ((*S*,*S*)-**2**).

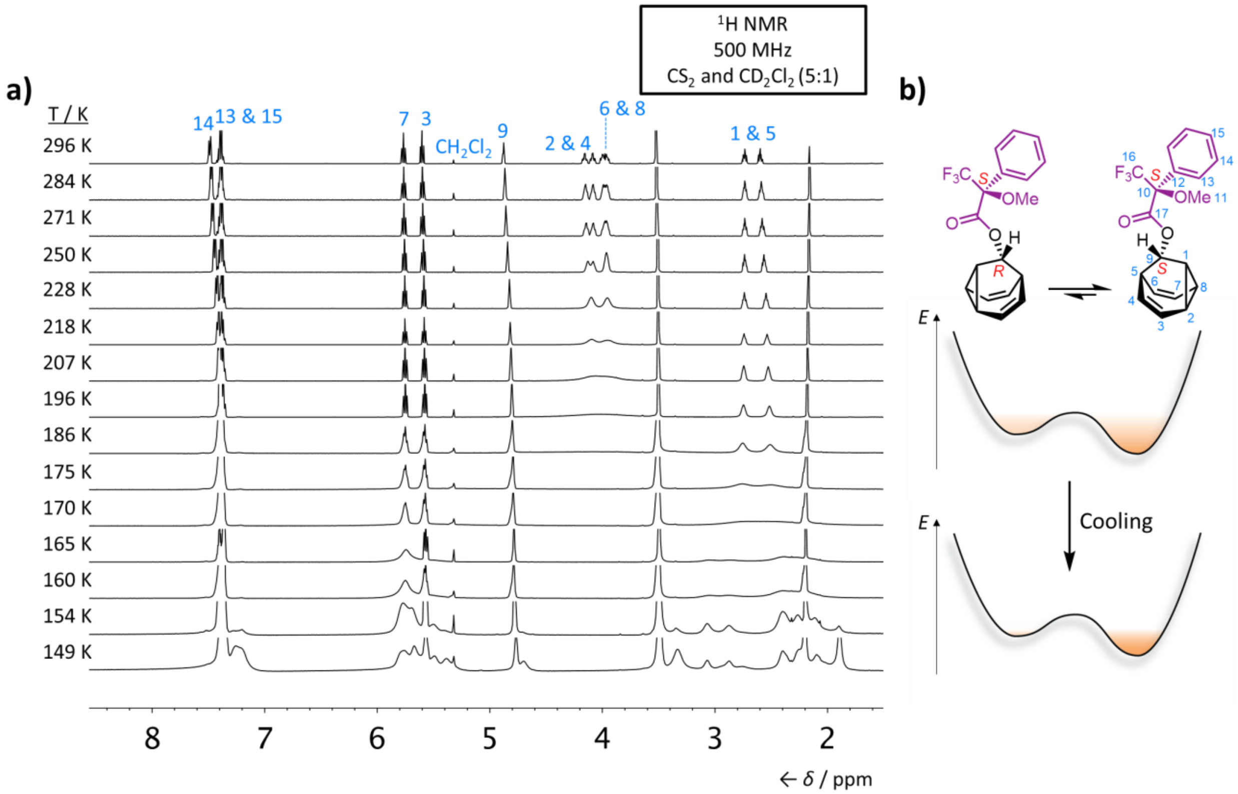

Figure S64. Partial ^{1}H VT NMR spectra of (a) (*R*,*S*)/(*S*,*S*)-**2** (296 K to 149 K) and (b) a schematic illustration of the change in equilibrium population on a simplified potential energy surface as the temperature decreases.

The barbaralanes undergo a rapid and reversible Cope rearrangement in solution. For example, diastereoisomer (*R*,*S*)-**2** is in fast exchange with isomer (*S*,*S*)-**2**, giving rise to a single set of resonances in the ^{13}C NMR spectrum recorded at ambient temperature. The chemical shift of each nucleus is indicative of its time-averaged chemical environment. Upon reducing the temperature to 159 K (Figure S65), the equilibrium shifts in favour of diastereoisomer (*S*,*S*)-**2**. This is evidenced as the peaks corresponding to the olefinic and cyclopropane environments broaden into the base and re-emerge. For example, two of the olefinic and cyclopropane environments are ~70 ppm at room temperature but at colder temperatures they have shifted to ~25 ppm. Environments labelled as 1, 5, 3 and 7 also broaden into the baseline at higher temperatures and re-emerge at colder temperatures.

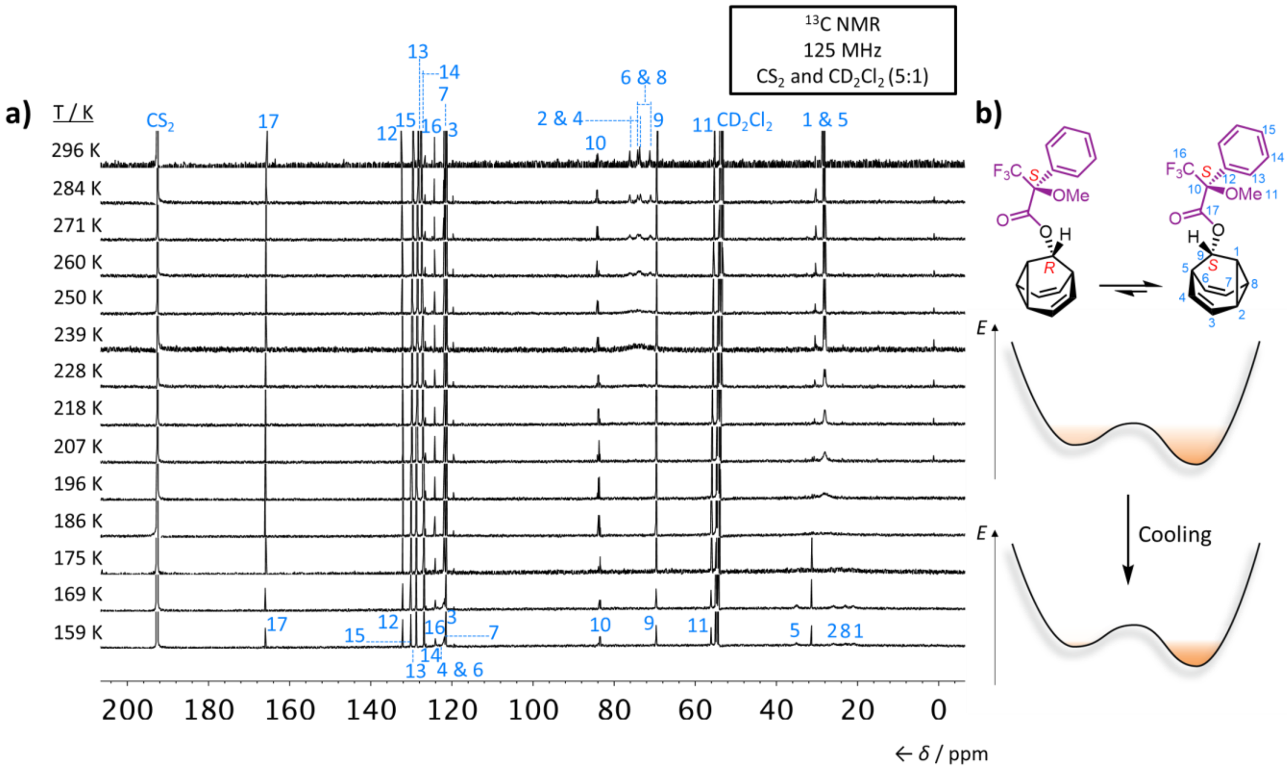

Figure S65. Partial ^{13}C VT NMR spectra of (a) (*R*,*S*)/(*S*,*S*)-**2** (296 K to 159 K) and (b) a schematic illustration of the change in equilibrium population on a simplified potential energy surface as the temperature decreases.

6.4 ^{13}C NMR Spectroscopic Comparison of (*R,S*)/(*S,S*)-2

The solid-state structures of (*R*,*R*)-**2** and (*S*,*S*)-**2** were probed further through ^{13}C solid-state NMR (ssNMR) spectroscopy (Figure S66 and Table S6). ssNMR experiments were performed for (*R*,*R*)-**2** (Figure S66). Comparison to the low-temperature solution-phase ^{13}C NMR spectrum of (*S*,*S*)-**2** (Figure S66c) supports the assignment that the diastereoisomer with the same configuration at position 9 of the barbaralane and at chiral tether (*i.e.*, *R* and *R*) is the thermodynamically favoured diastereoisomer and that the equilibrium is slightly shifted in solution at room temperate but is dominant at low temperatures. Comparison of the simulated ^{13}C NMR spectrum of (*R*,*R*)-**2** against the low-temperature solution-phase ^{13}C NMR spectrum of (*S*,*S*)-**2** and the ssNMR of (*R*,*R*)-**2** also supports our conclusion. The two derivatives studied, (*S*,*S*)-**2** and (*R*,*R*)-**2**, are enantiomers and thus their NMR spectroscopic signals can be directly compared. The upfield (<100 ppm) resonances of the ^{13}C ssNMR spectrum are well resolved. They match well with the experimental solution and simulated solid-state spectra (Figure S66), allowing for the reliable assignment of the upfield region of the spectra.

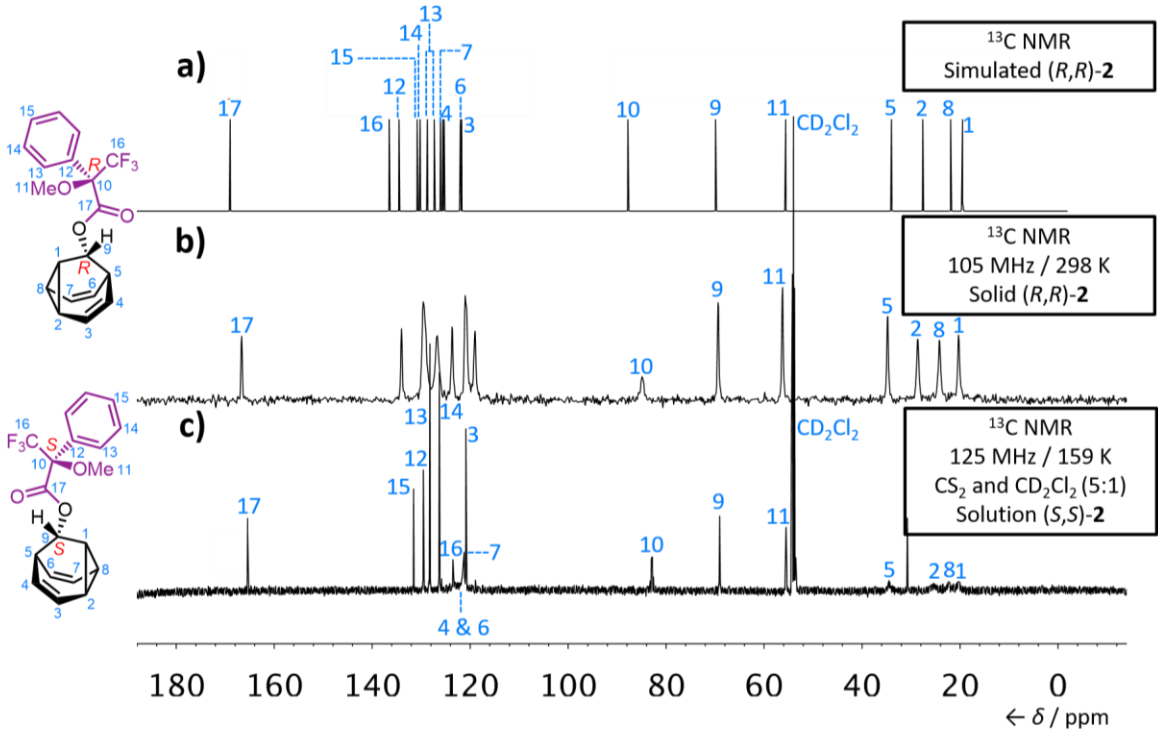

Figure S66. Comparison of ^{13}C NMR spectra of (*R*,*R*)-**2** and (*S*,*S*)-**2** under different conditions: (a) the simulated solid-state spectrum of (*R*,*S*)-**2**, (b) (*R*,*S*)-**2** in the solid state (as a powder) and (c) (*S*,*S*)-**2** in a CS_2–CD_2Cl_2 (5:1) solution at 159 K.

Table S6. Comparison of solid-state calculated ^{13}C NMR chemical shifts of (*R*,*R*)-**2** against solid-state experimental ^{13}C NMR chemical shifts of (*R*,*R*)-**2** and solution-state experimental ^{13}C NMR chemical shifts of (*S*,*S*)-**2**.

Atom	Solid-State Calculated ^{13}C NMR Chemical Shift (δ) (*R*,*R*)-2	Solid-State Experimental ^{13}C NMR Chemical Shift (δ) (*R*,*R*)-2	Solution-State Experimental ^{13}C NMR Chemical Shift (δ) (*S*,*S*)-2
1 or 5′	20.52	20.30	20.52
2 or 4′	22.35	24.21	22.35
3 or 3′	[a]	[a]	[a]
4 or 2′	[a]	[a]	[a]
5 or 1′	34.50	34.79	34.50
6 or 8′	[a]	[a]	[a]
7 or 7′	[a]	[a]	[a]
8 or 6′	25.20	28.63	25.20
9 or 9′	69.07	69.37	69.07
10 or 10′	165.43	166.66	165.43
11 or 11′	83.02	84.90	83.02
12 or 12′	[a]	[a]	[a]
13 or 13′	[a]	[a]	[a]
14 or 14′	[a]	[a]	[a]
15 or 15′	[a]	[a]	[a]
16 or 16′	[a]	[a]	[a]
17 or 17′	55.52	56.25	55.52

[a]Unable to assign umambigiously.

6.5 Dynamic NMR Spectroscopy of (*A*,*S*,*S*)/(*C*,*R*,*S*)-$\mathbf{L_{BB1}}$PdCl$_2$

At room temperature, several of the proton resonances of $\mathbf{L_{BB1}}$PdCl$_2$ are broadened due to exchange. The signals were resolved by recording a series of low-temperature ^{1}H NMR spectra (Figure S67). Signals corresponding to the two stereoisomers are present in an equilibrium ratio of 3:4 ($K = 1.33$) at 240 K. These signals were assigned to (*A*,*S*,*S*)- $\mathbf{L_{BB1}}$PdCl$_2$ and (*C*,*R*,*S*)-$\mathbf{L_{BB1}}$PdCl$_2$, respectively, on the basis of the energetic preference predicted by DFT (see *In Silico* Modelling, Section 9). The Gibbs energy difference at 240 K was calculated using the equation $\Delta G = -\mathrm{R}T\ln K$. A 2D exchange spectroscopy (EXSY) NMR spectrum acquired at 240 K (Figure S68) also allowed the rearrangement rate to be measured. The ratio of the on-diagonal signals to off-diagonal signals $[(I_{AA}+I_{BB})/(I_{AB}+I_{BA})]$ is 3.19 when using a mixing time τ_m = 200 ms, corresponding to a rate of 6.5 s^{-1}. The free energy of activation was then calculated by applying the Eyring equation.

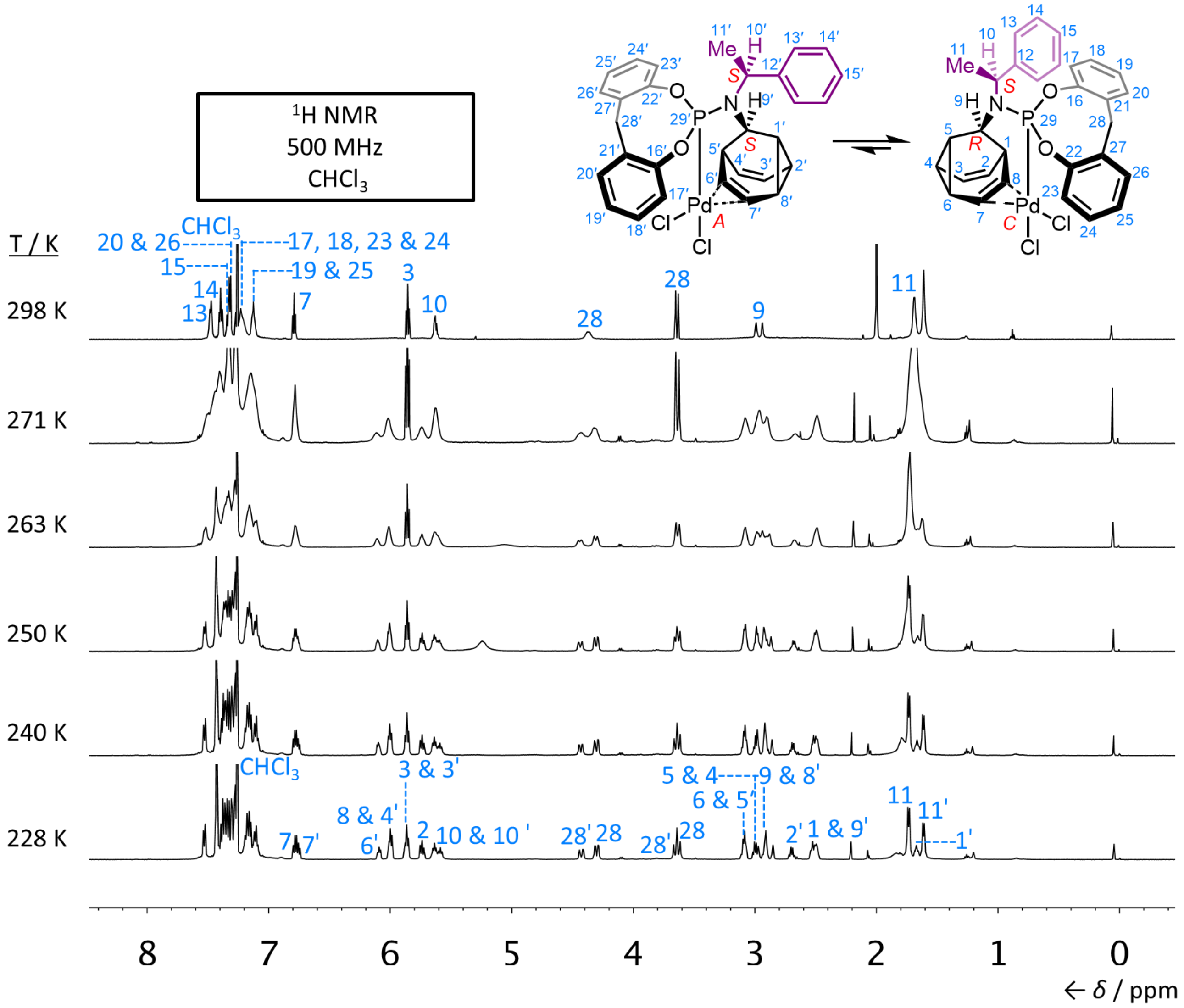

Figure S67. Partial ^{1}H VT NMR spectra of (*A*,*S*,*S*)/(*C*,*R*,*S*)-$\mathbf{L_{BB1}}PdCl_2$ (298 K to 228 K). The spectra at 298 K show that the diastereoisomers are in fast exchange, whereas at 228 K the diastereoisomers enter the slow-exchange regime and are in a ratio of 1.3:1 of (*C*,*R*,*S*)-$\mathbf{L_{BB1}}PdCl_2$ to (*A*,*S*,*S*)-$\mathbf{L_{BB}}PdCl_2$.

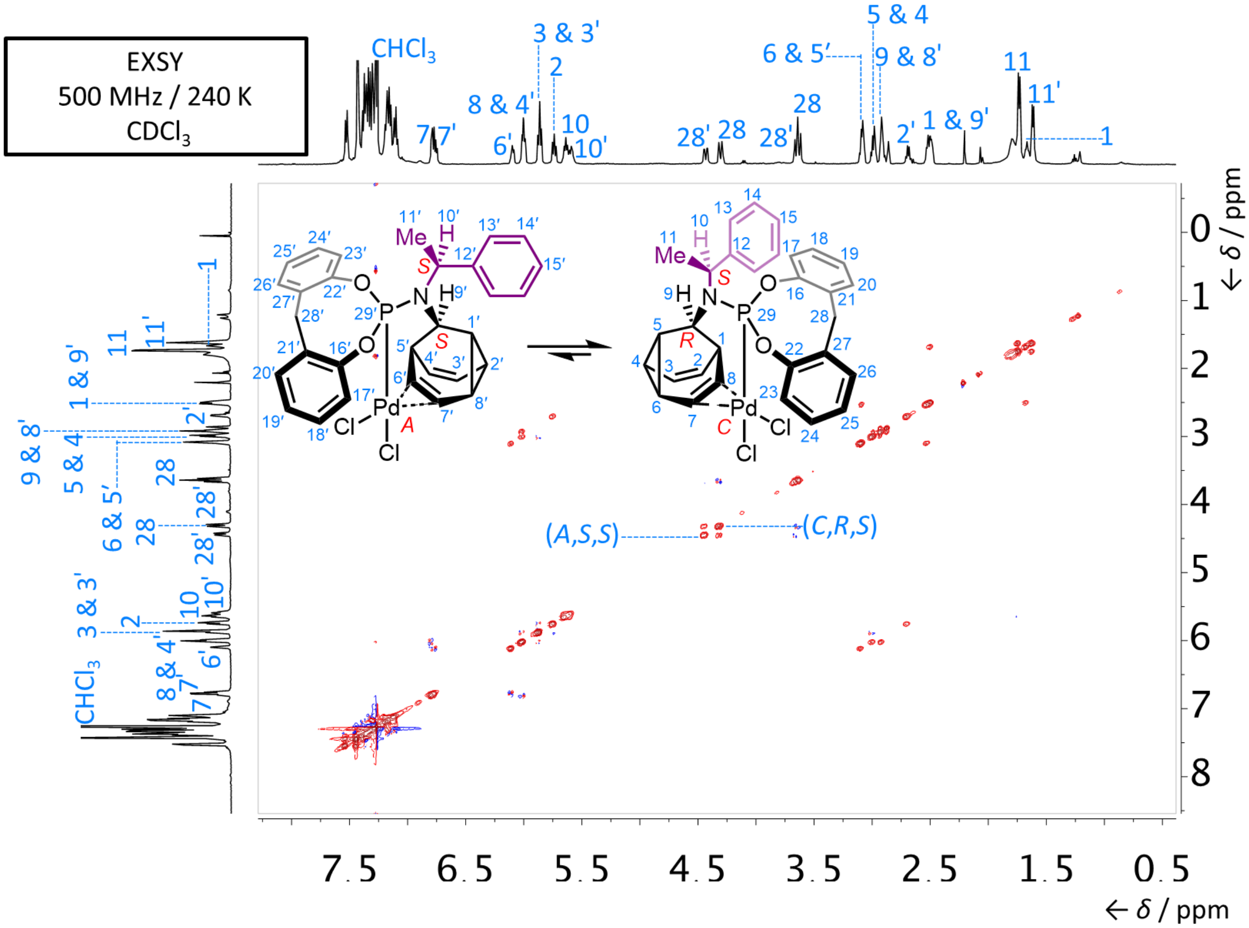

Figure S68. Partial EXSY NMR spectrum of (*A*,*S*,*S*)/(*C*,*R*,*S*)-**L$_{BB1}$**PdCl$_2$ with a mixing time τ_m = 200 ms at 240 K. The spectrum shows the resonances between the (*A*,*S*,*S*)-**L$_{BB1}$**PdCl$_2$ (minor) and (*C*,*R*,*S*)-**L$_{BB1}$**PdCl$_2$ (major) diastereoisomers.

6.6 Dynamic NMR Spectroscopy of (*C*,*R*,*S*)-$\mathbf{L_{BB1}}$RuCp(NCMe)·PF_6

In order to determine the dynamic stereoisomerisation rate of $\mathbf{L_{BB1}}$RuCp(NCMe)·PF_6 (Figure S69) we dissolved a crystalline sample of (*C*,*R*,*S*)-$\mathbf{L_{BB1}}$RuCp(NCMe)·PF_6 in $CDCl_3$ at 298 K where the equilibrium was established over a period of several minutes. A 4:1 equilibrium mixture of (*C*,*R*,*S*)-$\mathbf{L_{BB1}}$RuCp(NCMe)·PF_6 and (*A*,*S*,*S*)-$\mathbf{L_{BB1}}$RuCp(NCMe)·PF_6 is obtained. The rate of exchange was measured by recording sequential ^{1}H NMR spectra for a freshly dissolved sample of (*C*,*R*,*S*)-$\mathbf{L_{BB1}}$RuCp(NCMe)·PF_6 in $CDCl_3$ at 298 K. Assuming a first-order process with rate-limiting dissociation of MeCN, the rate was estimated (see Figure 5d in the manuscript) as the gradient of a straight line fitted to a plot of natural logarithm of the peak intensity against time, giving an observed rate constant k_{obs} of 2.56×10^{-3} s^{-1} ± 10%. Using the Eyring equation, this rate corresponds to a free energy of activation $\Delta G^{\ddagger}$ of 87.8 kJ·mol^{-1}.

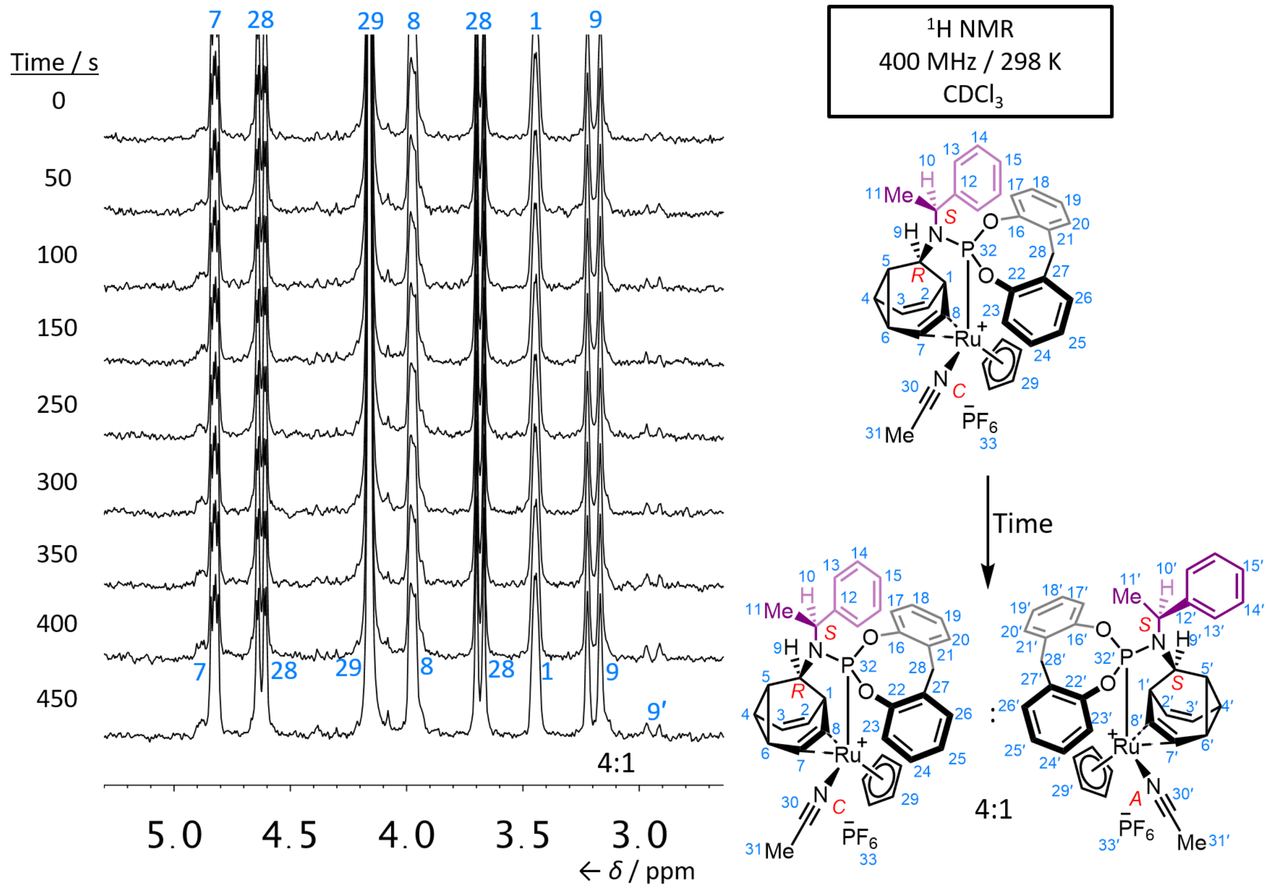

Figure S69. Partial ^{1}H NMR spectra (400 MHz, $CDCl_3$, 298 K) of $\mathbf{L_{BB1}}$RuCp(NCMe)·PF_6 obtained by acquiring spectra sequentially immediately after dissolving a crystalline sample of (*C*,*R*,*S*)-$\mathbf{L_{BB1}}$RuCp(NCMe)·PF_6. The growth of the signal at 2.9 ppm shows the equilibration to form (*A*,*S*,*S*)-$\mathbf{L_{BB1}}$RuCp(NCMe)·PF_6.

In order to determine the rate of MeCN exchange, the MeCN ligand of $\mathbf{L_{BB1}}$RuCp(NCMe)·PF_6 was exchanged for CD_3CN by dissolving 4.6 mg of the complex in $CDCl_3$ (0.6 mL) containing a drop of CD_3CN and standing overnight before evaporating solvent under reduced pressure. The resulting $\mathbf{L_{BB1}}$RuCp($NCCD_3$)·PF_6 complex was then dissolved in $CDCl_3$ (0.6 mL) at 298 K. A solution of MeCN (3.5 mg, ~15 equiv.) in $CDCl_3$ (0.1 mL) was added and rapidly mixed before a series of sequential ^{1}H NMR spectra were recorded (Figure S70).

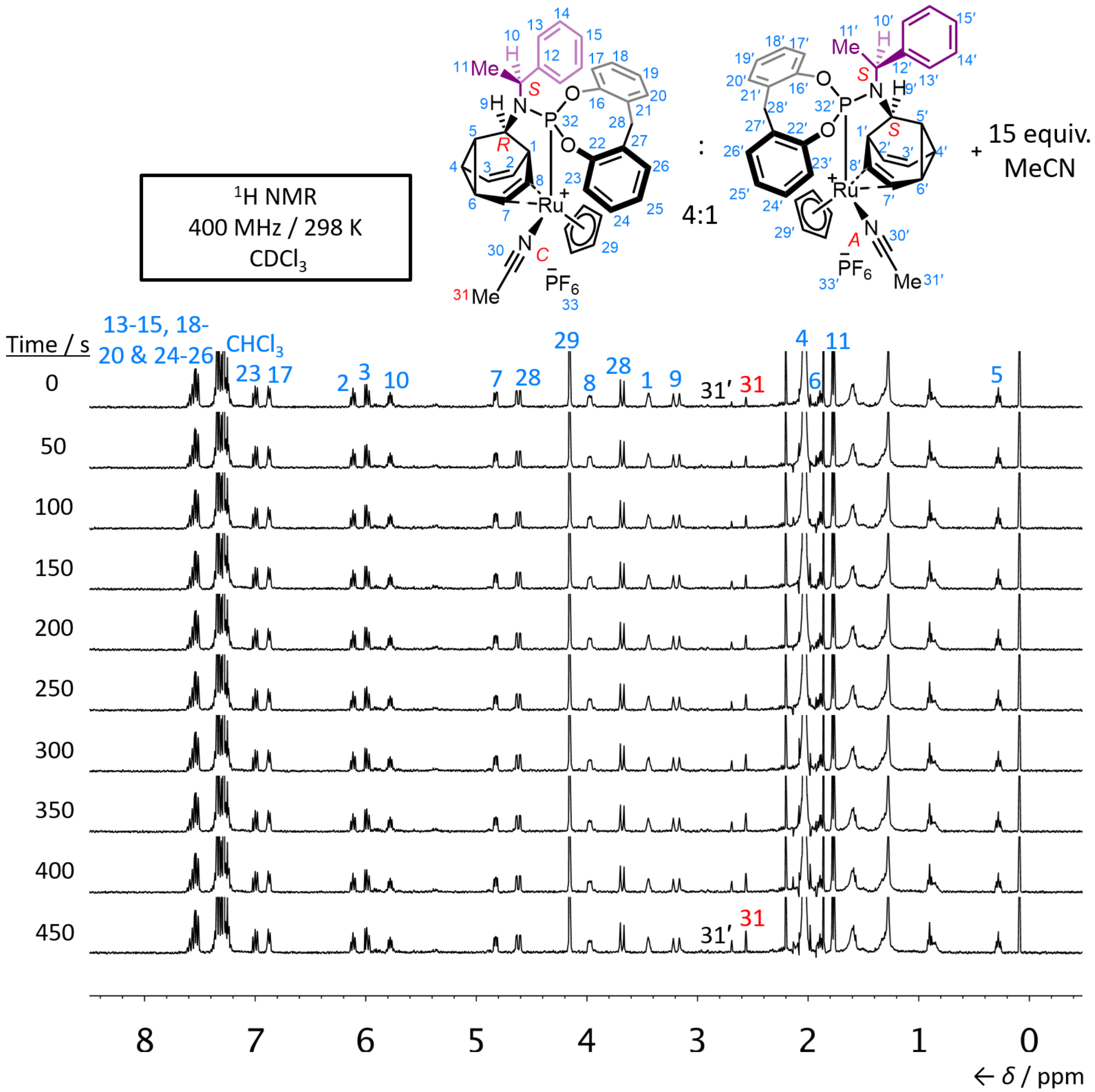

Figure S70. Partial ^{1}H NMR spectra of a 4:1 equilibrium mixture of (*C*,*R*,*S*)-**L$_{BB1}$**RuCp(NCCD$_3$)·PF$_6$ and (*A*,*S*,*S*)-**L$_{BB1}$**RuCp(NCCD$_3$)·PF$_6$ immediately after the addition of 15 equiv. MeCN showing the increase in the intensity of signals (31 and 31′) corresponding to coordinated MeCN. Note: Although the spectra show a 4:1 equilibrium mixture of (*C*,*R*,*S*)-**L$_{BB1}$**RuCp(NCCD$_3$)·PF$_6$ and (*A*,*S*,*S*)-**L$_{BB1}$**RuCp(NCCD$_3$)·PF$_6$, only the assignment of the major diastereoisomer ((*C*,*R*,*S*)-**L$_{BB1}$**RuCp(NCCD$_3$)·PF$_6$) is shown (blue and red labels), with the exception bar the signal corresponding to 31′ (black label).

The growth in the signals corresponding to non-deuterated MeCN proton environments of (*C*,*R*,*S*)-**L$_{BB1}$**RuCp(NCMe)·PF$_6$ (H$_{31}$) and (*A*,*S*,*S*)-**L$_{BB1}$**RuCp(NCMe)·PF$_6$ (H$_{31'}$) was monitored over several minutes. Assuming first-order processes with rate-limiting dissociation of CD$_3$CN, rates were

estimated (Figure S71) as the gradients of straight lines fitted to plots of natural logarithm of the peak intensity against time, giving observed rate constants, k_{obs}, of 1.27 × 10^{-3} s^{-1} ± 10.7% and 1.45 × 10^{-3} s^{-1} ± 13.5% for (*C*,*R*,*S*)-$\mathbf{L_{BB1}}$RuCp(NCMe)·PF_6 and (*A*,*S*,*S*)-$\mathbf{L_{BB1}}$RuCp(NCMe)·PF_6, respectively. Note that these two figures are within experimental error of one another and very close to the observed rate of dynamic stereoisomerisation. Using the Eyring equation, these rates corresponds to free energies of activation $\Delta G^{\ddagger}$ of 89.5 and 89.2 kJ·mol^{-1} for (*C*,*R*,*S*)-$\mathbf{L_{BB1}}$RuCp(NCMe)·PF_6 and (*A*,*S*,*S*)-$\mathbf{L_{BB1}}$RuCp(NCMe)·PF_6, respectively.

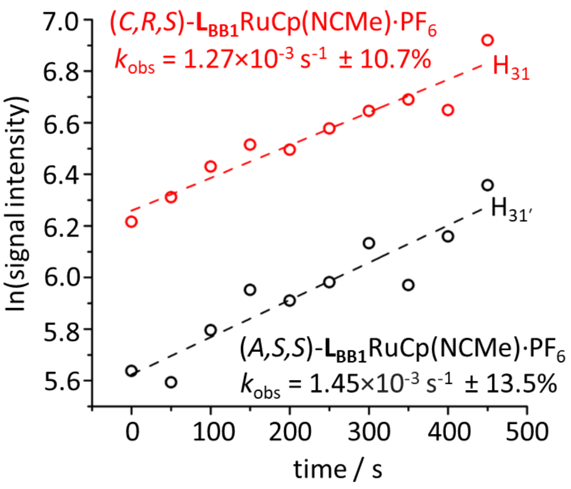

Figure S71. Plot of the natural logarithm of signal intensity for the two MeCN signals against time, showing the linear trend consistent with a first-order process with rate-limiting ligand dissociation.

6.7 NMR Measurements of (*A,S*)/(*C,R*)-L_{BB2}RuCp(NCMe)·PF_6 with Chiral Counterions

In order to determine the equilibrium constant of (*A,S*)/(*C,R*)-**L_{BB2}**RuCp(NCMe)·PF_6 upon the addition of two different chiral counterions (Bu_4N·*Δ*-TRISPHAT or Na·(*S*)-BORBIN), we dissolved (*A,S*)/(*C,R*)-**L_{BB2}**RuCp(NCMe)·PF_6 (1 equiv.) in $CDCl_3$ (0.45 mL) and added either Bu_4N·*Δ*-TRISPHAT or Na·(*S*)-BORBIN (1 equiv.) in $CDCl_3$ (0.45 mL). The resulting solution was rapidly mixed before a series of sequential ^{1}H NMR spectra were recorded (see Figure 6c in the manuscript and Figure S72).

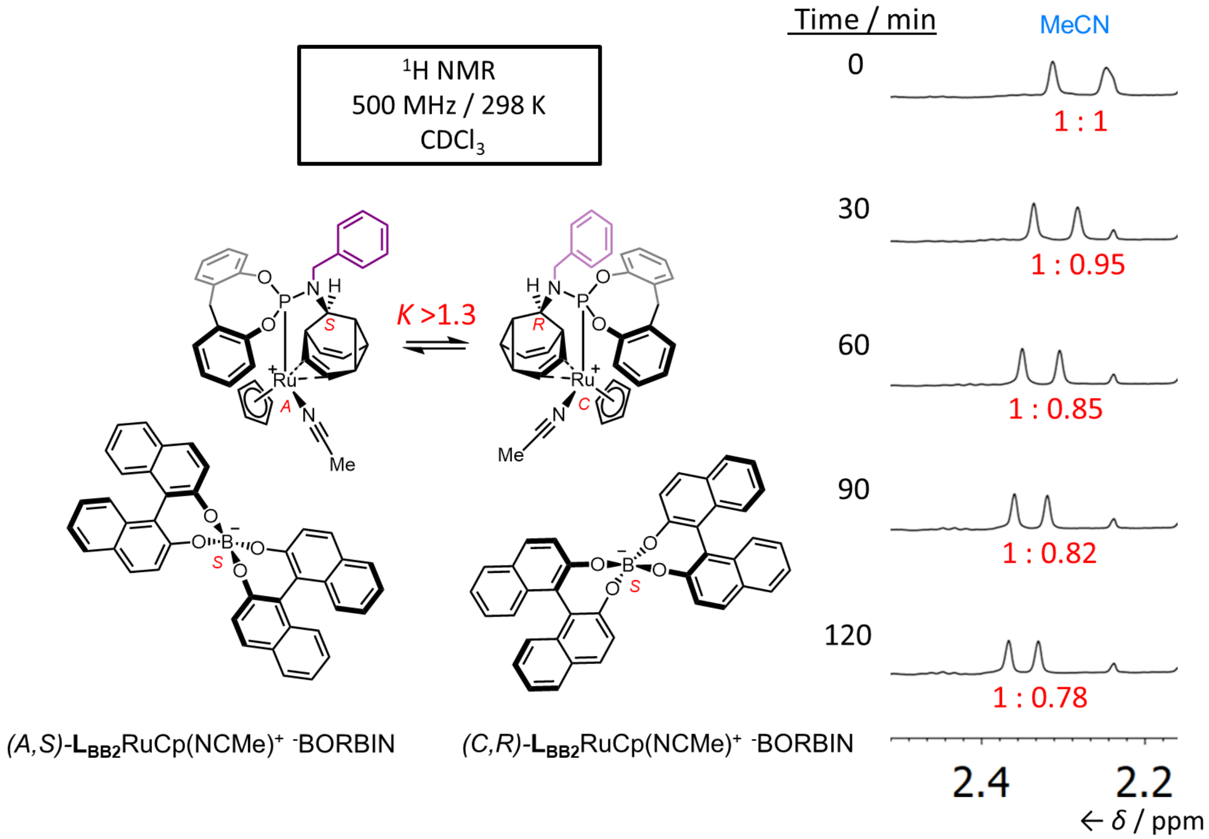

Figure S72. Evolution of partial ^{1}H NMR spectra of an equimolar mixture of (*A,S*)/(*C,R*)-**L_{BB2}**RuCp(NCMe)·PF_6 and Na·(*S*)-BORBIN.

In a solution of (*A,S*)/(*C,R*)-**L_{BB2}**RuCp(NCMe)·PF_6 in $CDCl_3$ we see a single ^{1}H NMR signal for the MeCN group (Figure S46), indicating the presence of the two enantiomeric complexes (which

appear identical by NMR). Upon the addition of chiral counterion, the MeCN signal is split in two. Over time, and by monitoring the integration of the MeCN resonances (initially 1:1 at 0 minutes but 1:0.78 at 120 minutes) we see that the initially racemic (*A,S*)/(*C,R*)**-L_{BB2}**RuCp(NCMe)·PF_6 becomes enriched in one stereoisomer with an equilibrium constant of K >1.3. Monitoring of (*A,S*)/(*C,R*)**-L_{BB2}**RuCp(NCMe)·PF_6 with the addition of either Bu_4N·*Δ*-TRISPHAT or Na·(*S*)-BORBIN was only possible for a limited time period due to degradation of the equilibrium mixture which is most likely caused by nucleophilic attack of adventitious water on the barbaralane core. We have observed that transition metal coordination increases reactivity of the barbaralane towards nucleophilic attack.

7. Photoluminescence Quantum Yield (PLQY) Measurements of (*C*,*R*,*S*)-L_{BB1}RuCp(NCMe)·PF_6

We tested the photoluminescence properties of (*C*,*R*,*S*)-$\mathbf{L_{BB1}}$RuCp(NCMe)·PF_6. The PLQY of (*C*,*R*,*S*)-$\mathbf{L_{BB1}}$RuCp(NCMe)·PF_6 in chloroform solution was determined by comparison against a quinine sulphate in an 0.1 M aqueous H_2SO_4 standard (Figure S73).[17,18] using the method recommended by Horiba Scientific.[19] A cuvette of path length 10 mm were used throughout the experiment. (*C*,*R*,*S*)-$\mathbf{L_{BB1}}$RuCp(NCMe)·PF_6 (Figure S74) is weakly luminescent upon excitation at 365 nm, emitting at 450 nm. However, a very low quantum yield of <0.01 was determined (calculated value 0.002).

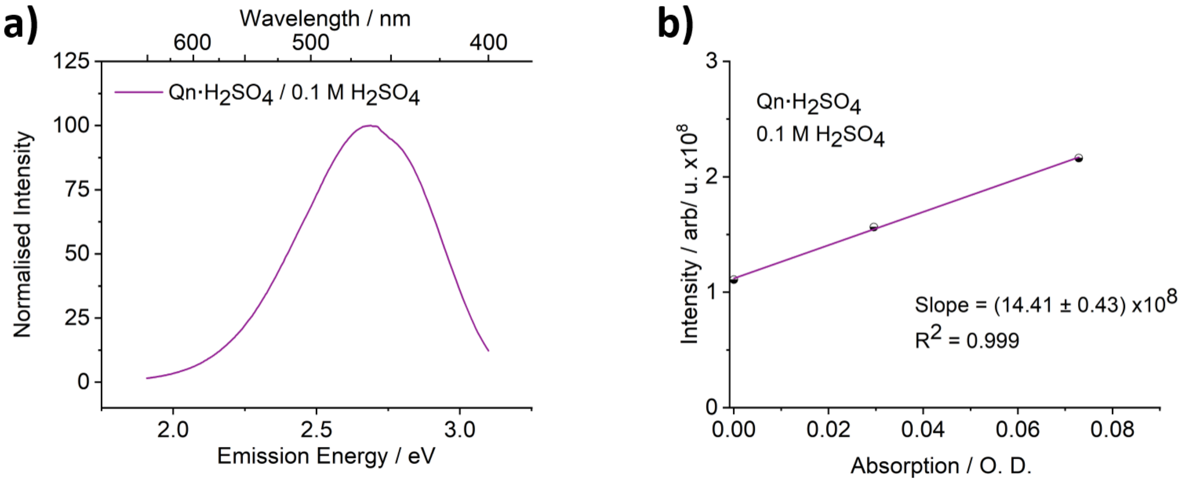

Figure S73. The (a) emission spectra and (b) emission *vs*. absorption slope of quinine sulphate in an 0.1 M aqueous H_2SO_4 solution.

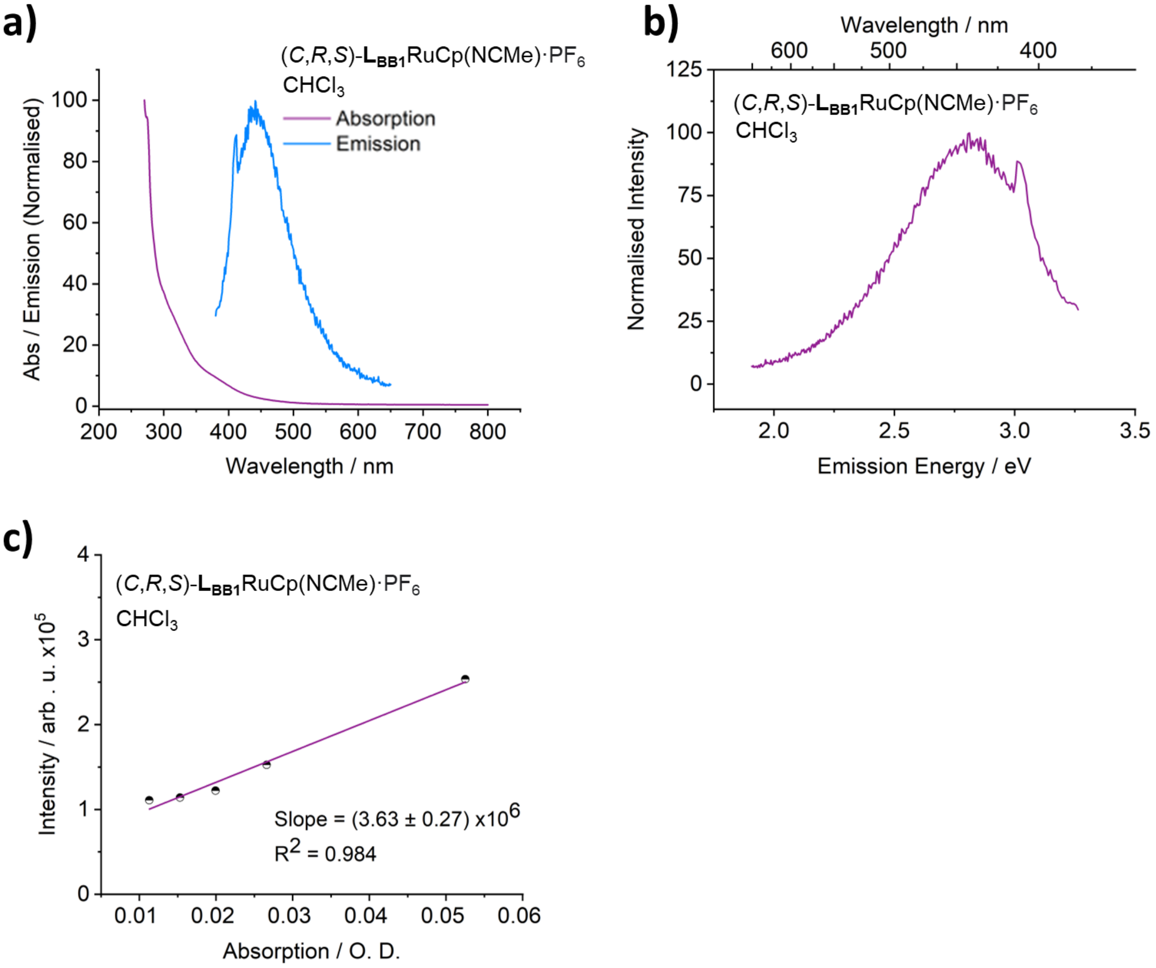

Figure S74. The (a) combined absorption and emission spectra (380 μM, λ_{ex} = 365 nm), (b) emission spectra and (c) emission *vs*. absorption slope of (*C*,*R*,*S*)-$\mathbf{L_{BB1}}$RuCp(NCMe)·PF_6 in $CHCl_3$.

8. X-Ray Crystallographic Analysis

8.1 (*R*)/(*S*)-1

Crystals of (*R*)/(*S*)-**1** suitable for X-ray diffraction were grown by slow evaporation of a saturated MeCN solution. Both enantiomers are present in the unit cell.

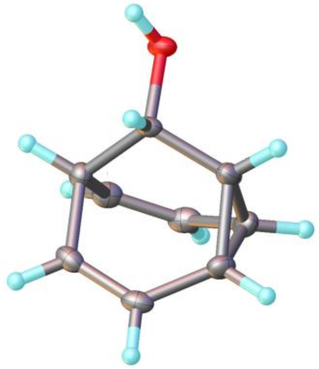

Figure S75. Solid-state structure of (*R*)/(*S*)-**1** including probability ellipsoids at 50%.

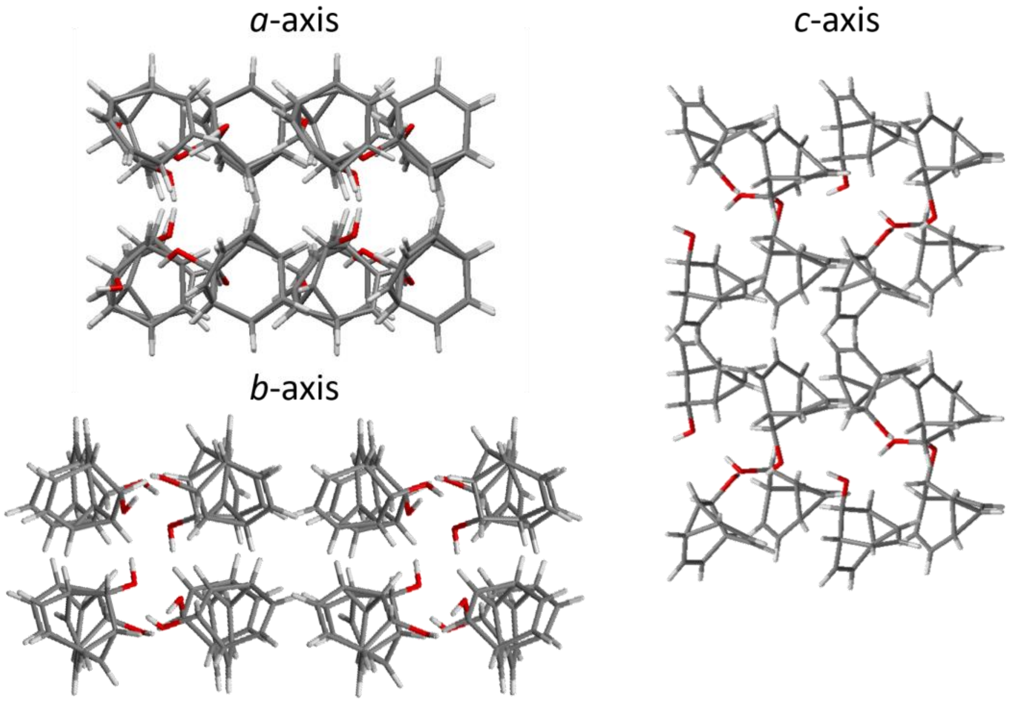

Figure S76. Solid-state superstructure of (*R*)/(*S*)-**1** viewed along the three unit cell axes.

Crystal data for (*R*)/(*S*)-1: $C_9H_{10}O$, M = 134.17, crystal system = monoclinic, space group = C2/c, a = 21.0930(5), b = 12.8259(3), c = 20.9409(5) Å, , α = 90°, β = 104.257(1)°, γ = 90°, U = 5490.8(2) $Å^3$, F(000) = 2304.0, Z = 32, D_c = 1.298 mg m^{-3}, μ = 0.653 mm^{-1} (Mo-Kα, λ = 0.71073 Å), T = 120(1) K. 31201 reflections were collected yielding 5092 unique data (R_{merg} = 0.0345). Final $wR_2(F^2)$ = 0.0945 for all data (377 refined parameters), conventional $R_1(F)$ = 0.0360 for 4356 reflections with I ≥ 2σ, GOF = 1.037. Crystallographic data for this structure has been deposited within the Cambridge Crystallographic Data Centre as supplementary publication CCDC-2068012.

8.2 (*S*,*S*)-2

Crystals of (*S*,*S*)-**2** suitable for X-ray diffraction were grown by slow evaporation of a saturated Et_2O solution.

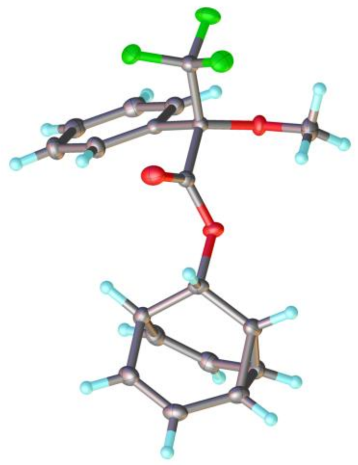

Figure S77. Solid-state structure of (*S*,*S*)-**2** including probability ellipsoids at 50%.

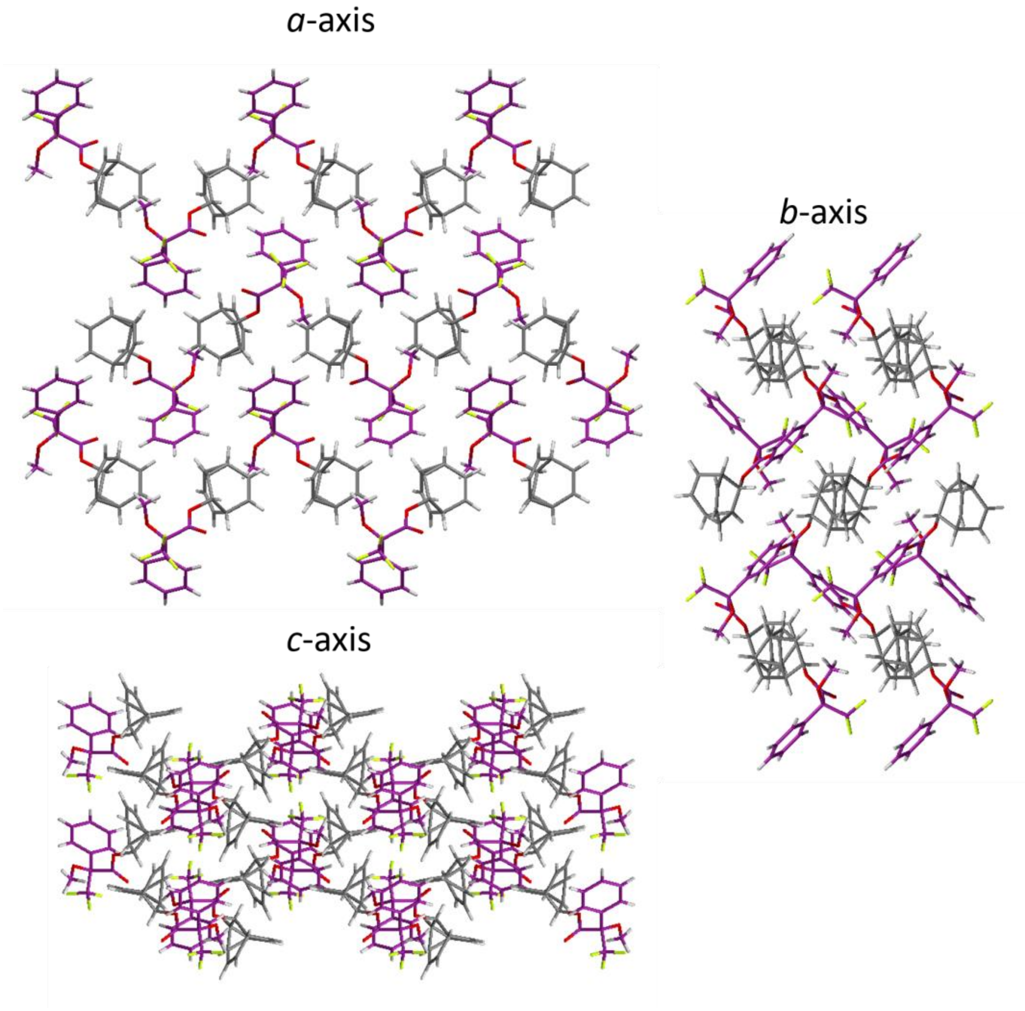

Figure S78. Solid-state superstructure of (*S*,*S*)-**2** viewed along the three unit cell axes.

Crystal data for (*S*,*S*)-2: $C_{19}H_{17}F_3O_3$, M = 350.32, crystal system = orthorhombic, space group = $P2_12_12_1$, a = 7.36678(15), b = 12.5886(2), c = 17.2671(3) Å, α = 90°, β = 90°, γ = 90°, U = 1601.30(5) Å^3, F(000) = 728.0, Z = 4, D_c = 1.453 mg m^{-3}, μ = 0.120 mm^{-1} (Mo-Kα, λ = 0.71073 Å), T = 120(1) K. 26817 reflections were collected yielding 4452 unique data (R_{merg} = 0.0387). Final $wR_2(F^2)$ = 0.0744 for all data (294 refined parameters), conventional $R_1(F)$ = 0.0311 for 4133 reflections with I ≥ 2σ, GOF = 1.014. Flack parameter 0.0(2), Hooft parameter -0.01(19). Crystallographic data for the structure has been deposited within the Cambridge Crystallographic Data Centre as supplementary publication CCDC-2068015.

8.3 (*R*,*R*)-2

Crystals of (*R*,*R*)-**2** suitable for X-ray diffraction were grown by slow evaporation of a saturated Et_2O solution.

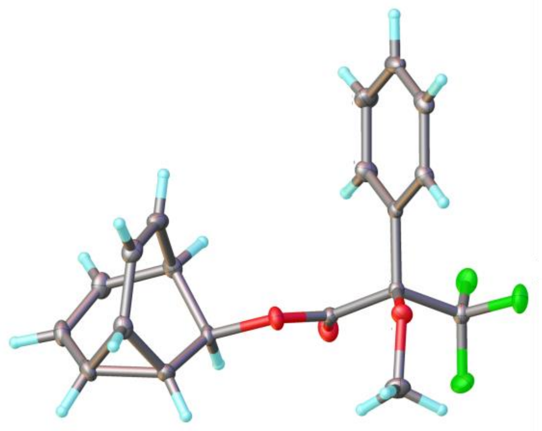

Figure S79. Solid-state structure of (*R*,*R*)-**2** including probability ellipsoids at 50%.

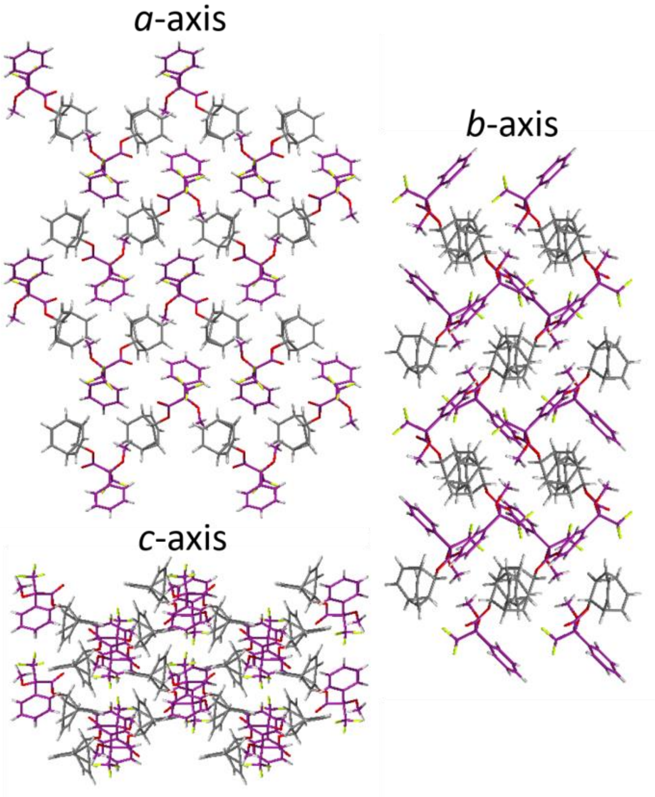

Figure S80. Solid-state superstructure of (*R*,*R*)-**2** viewed along the three unit cell axes.

Crystal data for (*R*,*R*)-2: $C_{19}H_{17}F_3O_3$, M = 350.32, crystal system = orthorhombic, space group = $P2_12_12_1$, a = 7.3559(3), b = 12.5594(5), c = 17.2298(7) Å, α = 90°, β = 90°, γ = 90°, U = 1591.79(11) $Å^3$, F(000) = 728.0, Z = 4, D_c = 1.462 mg m^{-3}, μ = 0.121 mm^{-1} (Mo-Kα, λ = 0.71073 Å), T = 120(1) K. 34205 reflections were collected yielding 4434 unique data (R_{merg} = 0.0342). Final $wR_2(F^2)$ = 0.0759 for all data (294 refined parameters), conventional $R_1(F)$ = 0.0309 for 4069 reflections with I ≥ 2σ, GOF = 1.051. Flack parameter -0.1(1), Hooft parameter -0.1(1). Crystallographic data for the structure has been deposited within the Cambridge Crystallographic Data Centre as supplementary publication CCDC-2068016.

8.4 (***R,R***)/(***S,S***)-4

Crystals of (*R,R*)/(*S,S*)-**4** suitable for X-ray diffraction were grown by slow cooling of a hot and saturated MeCN solution.

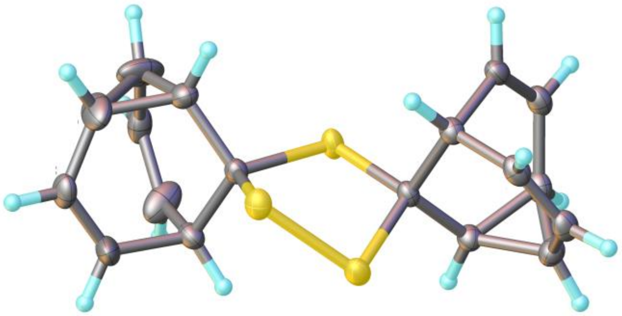

Figure S81. Solid-state structure of (*R,R*)/(*S,S*)-**4** including probability ellipsoids at 50%.

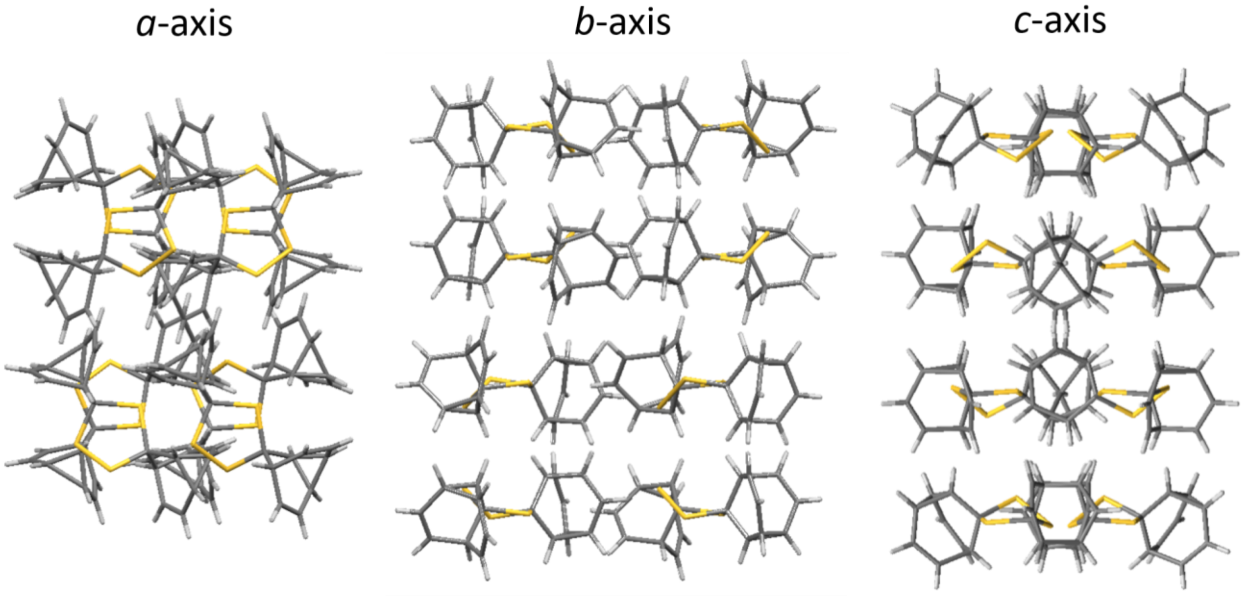

Figure S82. Solid-state superstructure of (*R,R*)/(*S,S*)-**4** viewed along the three unit cell axes.

Crystal data for (*R,R*)/(*S,S*)-4: $C_{18}H_{16}S_3$, M = 328.49, crystal system = orthorhombic, space group = Pbcn, a = 20.4767(4), b = 9.3487(2), c = 15.5799(3) Å, α = 90°, β = 90°, γ = 90°, U = 2982.5(1) Å^3, F(000) = 1376.0, Z = 8, D_c = 1.463 mg m^{-3}, μ = 0.486 mm^{-1} (Mo-Kα, λ = 0.71073 Å), T =

120(1) K. 27741 reflections were collected yielding 3964 unique data (R_{merg} = 0.0572). Final $wR_2(F^2)$ = 0.1187 for all data (190 refined parameters), conventional $R_1(F)$ = 0.0432 for 3198 reflections with $I \geq 2\sigma$, GOF = 1.038. Crystallographic data for this structure has been deposited within the Cambridge Crystallographic Data Centre as supplementary publication CCDC-2068013.

8.5 7

Crystals of **7** suitable for X-ray diffraction were grown by slow cooling of a hot and saturated MeCN solution.

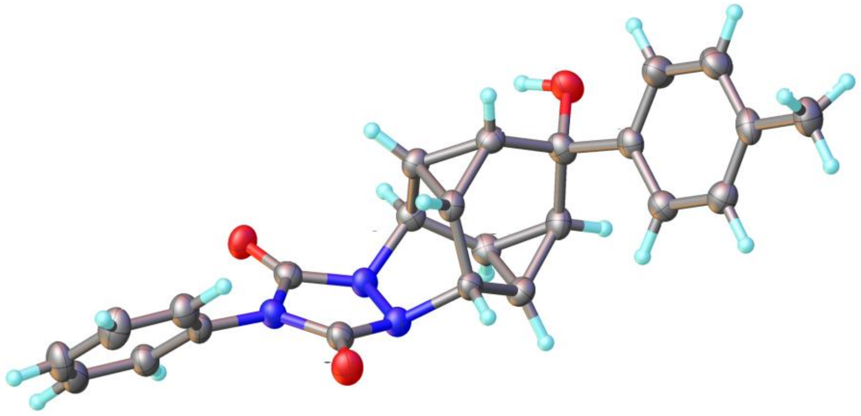

Figure S83. Solid-state structure of **7** including probability ellipsoids at 50%.

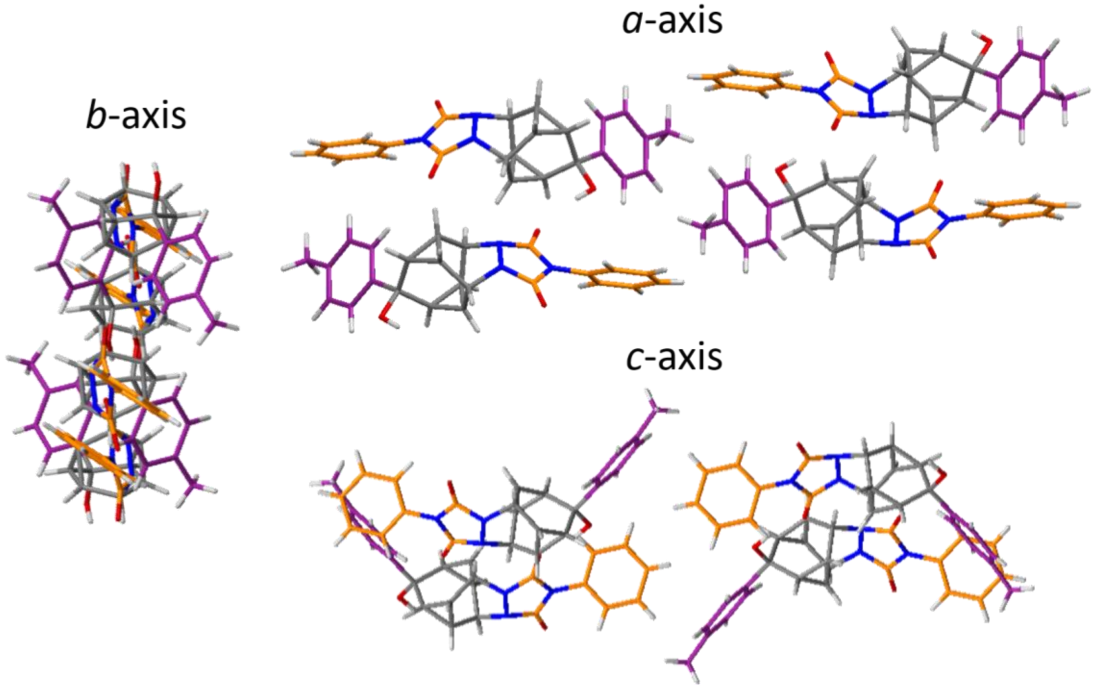

Figure S84. Solid-state superstructure of **7** viewed along the three unit cell axes.

Crystal data for 7: $C_{24}H_{21}N_3O_3$, M = 399.44, crystal system = monoclinic, space group = $P2_1/n$, a = 6.2961(4), b = 30.3288(13), c = 9.9483(7) Å, β = 98.960(6)°, α = 90°, β = 98.960(6)°, γ = 90°, U = 1876.48(19) Å^3, F(000) = 840.0, Z = 4, D_c = 1.414 mg m^{-3}, μ = 0.095 mm^{-1} (Mo-Kα, λ = 0.71073 Å), T = 120(1) K. 14914 reflections were collected yielding 3695 unique data (R_{merg} = 0.1009). Final $wR_2(F^2)$ = 0.1484 for all data (347 refined parameters), conventional $R_1(F)$ = 0.0586 for 2113 reflections with I ≥ 2σ, GOF = 1.039. Crystallographic data for the structure has been deposited within the Cambridge Crystallographic Data Centre as supplementary publication CCDC-2068017.

8.6 S1

Crystals of **S1** suitable for X-ray diffraction were grown by slow evaporation of a saturated CH_2Cl_2 solution.

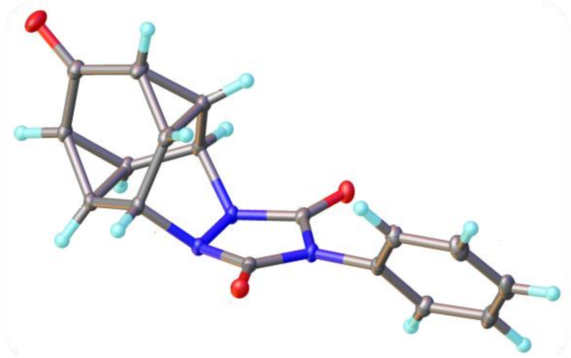

Figure S85. Solid-state structure of **S1** including probability ellipsoids at 50%.

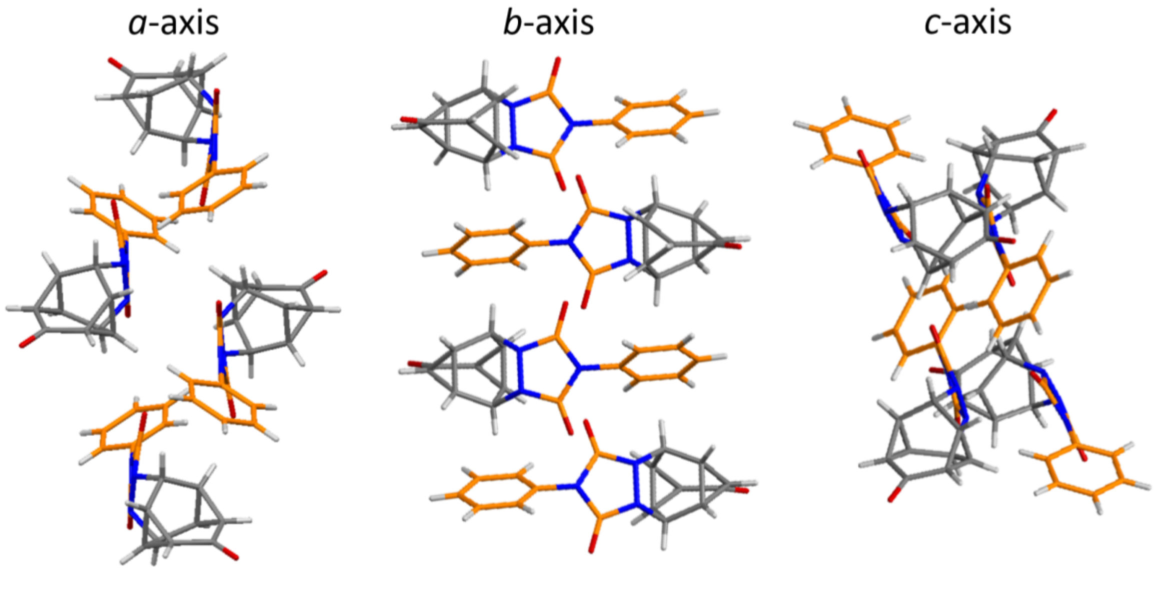

Figure S86. Solid-state superstructure of **S1** viewed along the three unit cell axes.

Crystal data for S1: $C_{17}H_{13}N_3O_3$, M = 307.30, crystal system = monoclinic, space group = $P2_1/n$, a = 14.3555(4), b = 6.2807(2), c = 16.0289(4) Å, β = 110.898(1)°, α = 90°, β = 110.8978(10)°, γ = 90°, U = 1350.14(7) Å^3, F(000) = 640.0, Z = 4, D_c = 1.512 mg m^{-3}, μ = 0.107 mm^{-1} (Mo-Kα, λ =

0.71073 Å), T = 120(1) K. 23181 reflections were collected yielding 3883 unique data (R_{merg} = 0.0441). Final $wR_2(F^2)$ = 0.0993 for all data (260 refined parameters), conventional $R_1(F)$ = 0.0381 for 3549 reflections with I ≥ 2σ, GOF = 1.061. Crystallographic data for the structure has been deposited within the Cambridge Crystallographic Data Centre as supplementary publication CCDC-2068019.

8.7 (*R,S*)-5

Crystals of (*R,S*)-**5** suitable for X-ray diffraction were grown by slow cooling of the sample at a temperature of 5 °C from room temperature and were transferred to the diffractometer by a 'dry ice' technique.

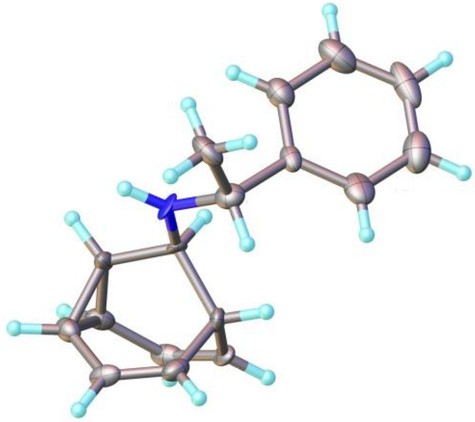

Figure S87. Solid-state structure of (*R,S*)-**5** including probability ellipsoids at 50%.

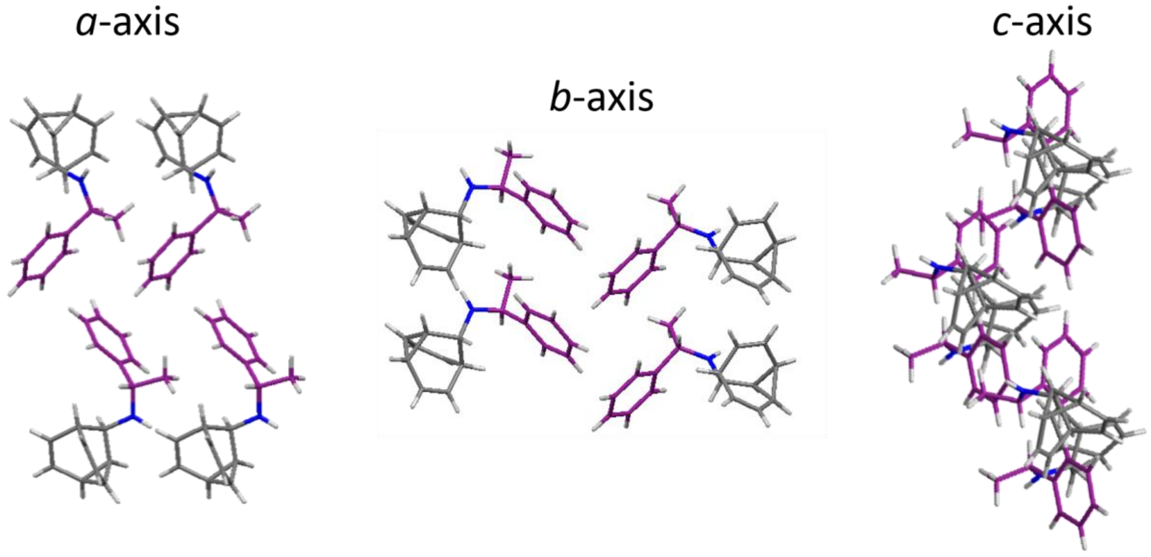

Figure S88. Solid-state superstructure of (*R,S*)-**5** viewed along the three unit cell axes.

Crystal data for (*S*,*R*)-5: $C_{17}H_{19}N$, M = 237.33, crystal system = triclinic, space group = P1, a = 6.1684(9), b = 6.3485(9), c = 28.326(4) Å, α = 84.356(4), β = 104.257(1), γ = 61.967(4)°, U = 974.4(2) Å^3, F(000) = 384.0, Z = 3, D_c = 1.213 mg m^{-3}, μ = 0.070 mm^{-1} (Mo-Kα, λ = 0.71073 Å), T = 120(1) K. 16714 reflections were collected yielding 7522 unique data (R_{merg} = 0.1671). Final $wR_2(F^2)$ = 0.1735 for all data (490 refined parameters), conventional R_1(F) = 0.0797 for 2845 reflections with I ≥ 2σ, GOF = 0.912. Flack parameter 5.6(10). Crystallographic data for the structure has been deposited within the Cambridge Crystallographic Data Centre as supplementary publication CCDC-2068015.

CheckCIF Alert Comments:

PLAT026_ALERT_3_B Ratio Observed / Unique Reflections (too) Low, 38% Check

Comment – Unstable at room temperature poor quality crystals of the compound didn't diffract well and very few reflections were observed even at maximum exposure. Nevertheless, the chemical identity of the compound has been confirmed.

PLAT340_ALERT_3_B Low Bond Precision on C-C Bonds, 0.01504 Ang.

Comment – Unstable at room temperature poor quality crystals of the compound didn't diffract well and very few reflections were observed even at maximum exposure. That inevitably affected the precision of determination of geometrical parameters.

8.8 (*A*,*S*,*S*)/(*C*,*R*,*S*)-$\mathbf{L_{BB1}}$PdCl$_2$

Crystals of (*A*,*S*,*S*)/(*C*,*R*,*S*)-$\mathbf{L_{BB1}}$PdCl$_2$ suitable for X-ray diffraction were grown by slow evapouration of a saturated EtOAc solution. The unit cell contains four independent molecules – two near-identical conformers of (*A*,*S*,*S*)-$\mathbf{L_{BB1}}$PdCl$_2$ and two near-identical conformers of (*C*,*R*,*S*)-$\mathbf{L_{BB1}}$PdCl$_2$.

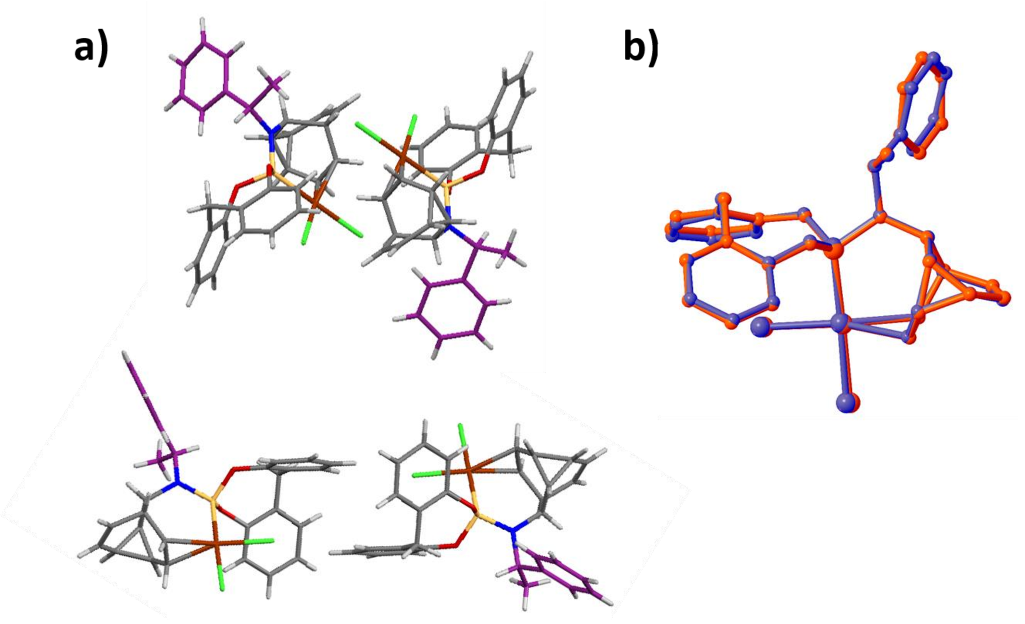

Figure S89. Solid-state structure of (a) the two near-identical conformers of (*A*,*S*,*S*)-$\mathbf{L_{BB1}}$PdCl$_2$ as well as the two near-identical conformers of (*C*,*R*,*S*)-$\mathbf{L_{BB1}}$PdCl$_2$ and (b) an overlay of two of the near-identical conformers of (*C*,*R*,*S*)-$\mathbf{L_{BB1}}$PdCl$_2$ showing the similarity of their geometries.

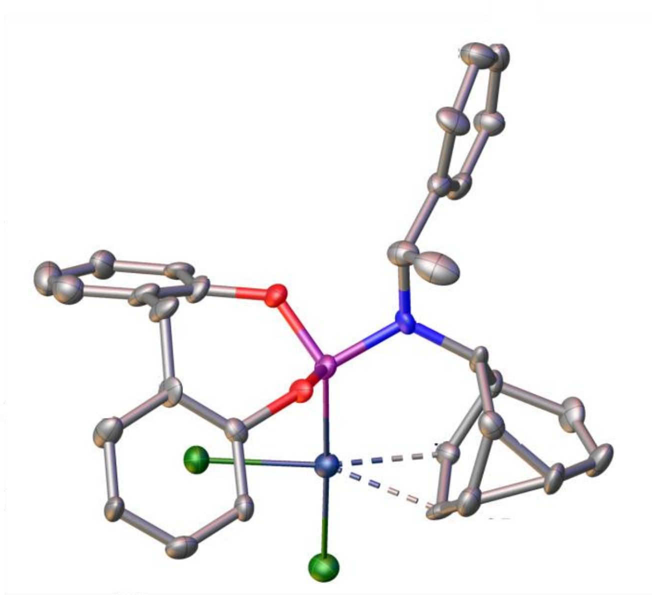

Figure S90. Solid-state structure of (*A*,*S*,*S*)-$\mathbf{L_{BB1}}$PdCl$_2$ including probability ellipsoids at 50%.

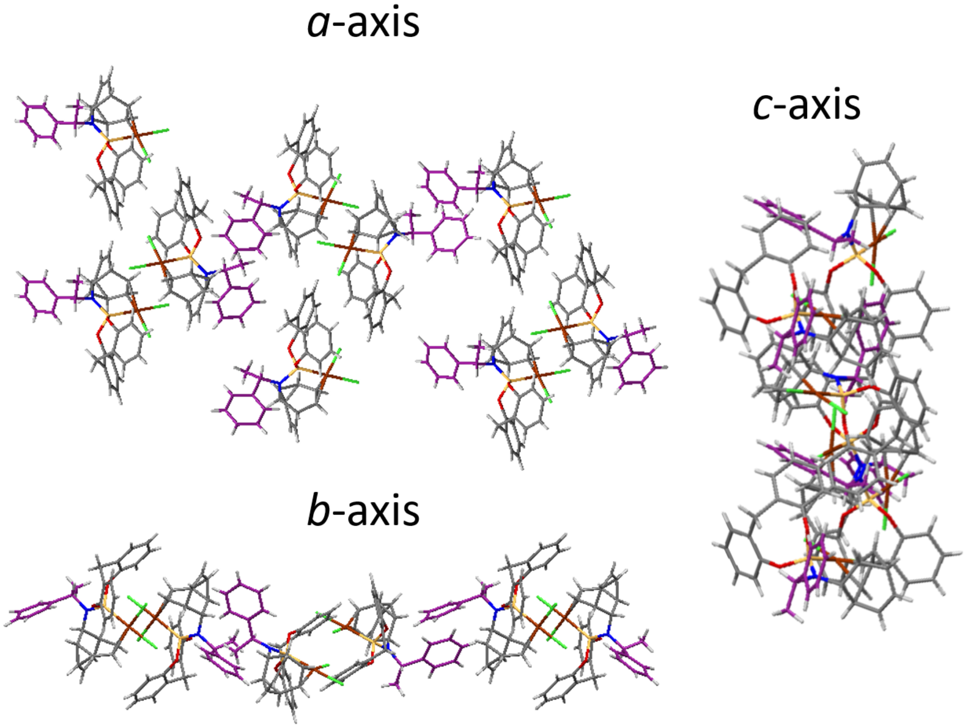

Figure S91. Solid-state superstructure of (*A*,*S*,*S*)/(*C*,*R*,*S*)-$\mathbf{L_{BB1}}$PdCl$_2$ viewed along the three unit cell axes.

Crystal data for (*A*,*S*,*S*)/(*C*,*R*,*S*)-L_{BB1}PdCl$_2$: $C_{30}H_{27}Cl_2NO_2PPd \times 0.625\ C_4H_8O_2$, M = 697.74, crystal system = triclinic, space group = P1, a = 9.2399(6), b = 12.7855(9), c = 28.2827(19) Å, α = 95.365(2), β = 98.805(2), γ = 110.791(2)°, U = 3047.1(4) Å^3, F(000) = 1424.0, Z = 4, D_c = 1.521 mg m^{-3}, μ = 0.872 mm^{-1} (Mo-Kα, λ = 0.71073 Å), T = 120(1) K. 48567 reflections were collected yielding 27453 unique data (R_{merg} = 0.0748). Final $wR_2(F^2)$ = 0.1787 for all data (1500 refined parameters), conventional $R_1(F)$ = 0.0711 for 17333 reflections with I ≥ 2σ, GOF = 1.029. Flack parameter -0.01(2), Hooft parameter -0.03(2). Crystallographic data for the structure has been deposited within the Cambridge Crystallographic Data Centre as supplementary publication CCDC-2068019.

CheckCIF Alert Comments:

PLAT250_ALERT_2_B Large U3/U1 Ratio for Average U(i,j) Tensor, 4.4 Note

Comment – The elongated thermal ellipsoids in the structure mainly belong to not fully occupied atoms of disordered solvent molecules. Furthermore elaborate treatment of this disorder was deemed to be outside of the scope of this structural investigation, focused on the geometry of metal complex.

PLAT331_ALERT_2_B Small Aver Phenyl C-C Dist C16C --C21C, 1.36 Ang.

Comment – This particular phenyl ring is slightly disordered, however the attempts to model this minor disorder did not result in any improvement of formal criteria of the refinement and thus were abandoned.

PLAT342_ALERT_3_B Low Bond Precision on C-C Bonds, 0.02287 Ang.

Comment – Disorder of solvent molecules and minor disorder of some peripheral Ph-rings are probably the cause of this alert. The elaborate treatment of these disordered fragments was deemed to be outside of the aim of this structural investigation, focused on the geometry of metal complex.

8.9 (*C*,*R*,*S*)-$\mathbf{L_{BB1}}$RuCp(NCMe)·PF_6

Crystals of (*C*,*R*,*S*)-$\mathbf{L_{BB1}}$RuCp(NCMe)·PF_6 suitable for X-ray diffraction were grown by slow evapouration of a saturated $CDCl_3$ solution. A single configurational and conformational isomer, (*C*,*R*,*S*)-$\mathbf{L_{BB1}}$RuCp(NCMe)·PF_6 is present in the unit cell, as a solvate with $CDCl_3$.

Distorted square pyramidal geometry: Taking the centroid of the μ^5-Cp ligand as a coordinated pseudo-atom, the complex can be treated as a distorted square pyramid. The bond angles between the apical Cp and each of the four ligands at the base of the square pyramid are all >115° (Cp-Ru-P 131.0°, Cp-Ru-N 121.0°, Cp-Ru-C7 119.8°, and Cp-Ru-C8 120.6°), whereas the bond angles between adjacent coordination sites at the base of the square pyramid are all <85° (P-Ru-N 84.4°, N-Ru-C7 83.7°, C7-Ru-C8 36.2°, and C8-Ru-P 80.2°).

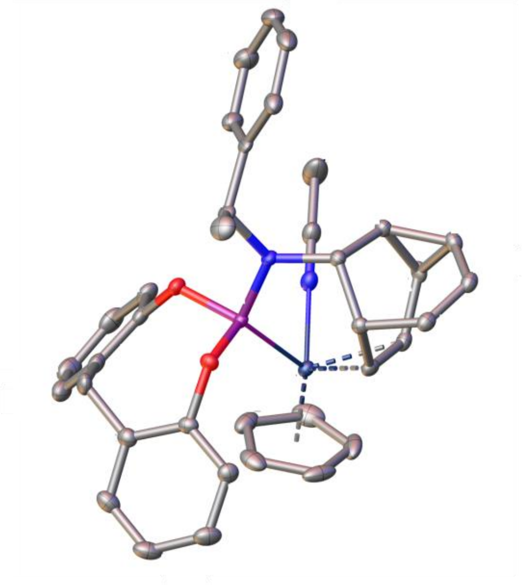

Figure S92. Solid-state structure of (*C*,*R*,*S*)-$\mathbf{L_{BB1}}$RuCp(NCMe)·PF_6 including probability ellipsoids at 50%. The PF_6^- counterion is omitted for clarity.

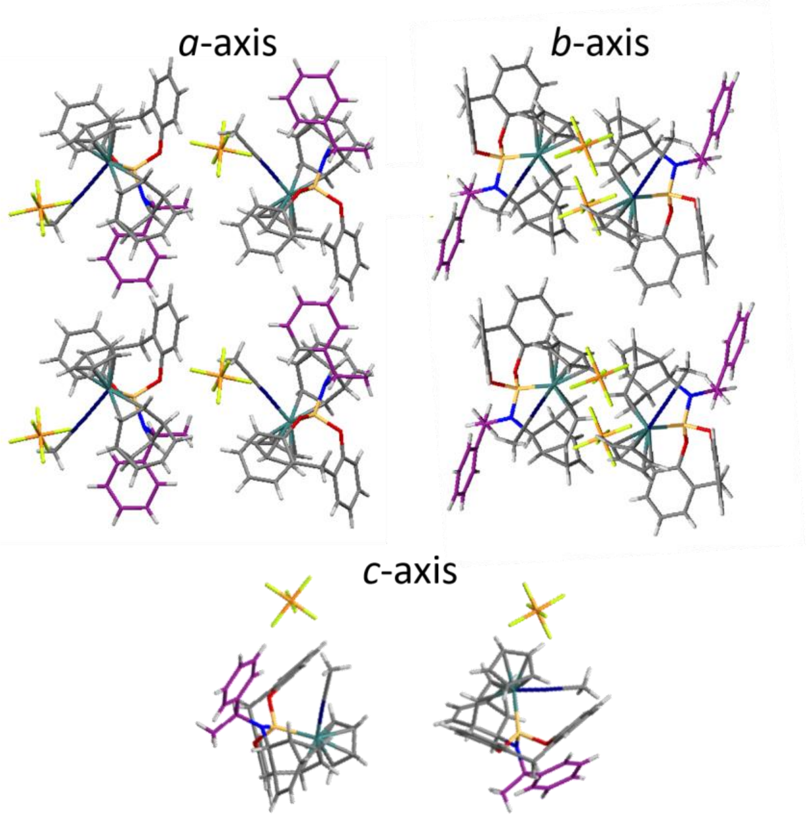

Figure S93. Solid-state superstructure of (*C*,*R*,*S*)-$\mathbf{L_{BB1}}$RuCp(NCMe)·PF_6 viewed along the three unit cell axes.

Crystal data for (*C*,*R*,*S*)-$\mathbf{L_{BB1}}$RuCp(NCMe)·$\mathbf{PF_6}$: $C_{38}H_{37}Cl_3F_6N_2O_2P_2Ru$, M = 937.05, crystal system = monoclinic, space group = $P2_1$, a = 8.7715(3), b = 18.7732(6), c = 11.9803(4) Å, α = 90°, β = 100.055(1)°, γ = 90°, U = 1942.48(11) $Å^3$, F(000) = 948.0, Z = 2, D_c = 1.602 mg m^{-3}, μ = 0.759 mm^{-1} (Mo-Kα, λ = 0.71073 Å), T = 120(1) K. 47392 reflections were collected yielding 11303 unique data (R_{merg} = 0.0405). Final $wR_2(F^2)$ = 0.0613 for all data (489 refined parameters), conventional $R_1(F)$ = 0.0300 for 10892 reflections with I ≥ 2σ, GOF = 1.039. Flack parameter -0.019(8), Hooft parameter -0.032(8). Crystallographic data for the structure has been deposited within the Cambridge Crystallographic Data Centre as supplementary publication CCDC-2068020.

8.10 (*A,S*)/(*C,R*)-$\mathbf{L_{BB2}}$RuCp(NCMe)·PF_6

Crystals of (*A,S*)/(*C,R*)-$\mathbf{L_{BB2}}$RuCp(NCMe)·PF_6 suitable for X-ray diffraction were grown by slow evapouration of a saturated $CDCl_3$ solution.

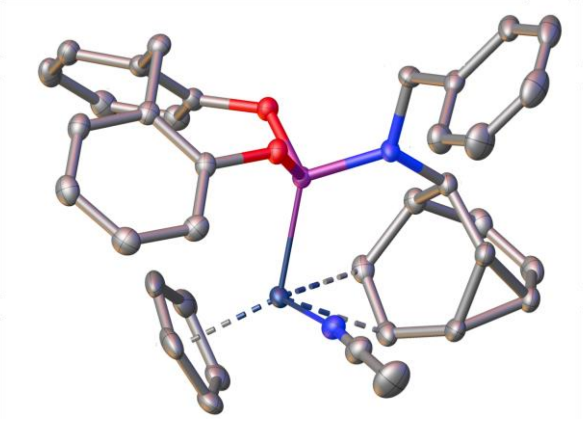

Figure S94. Solid-state structure of (*A,S*)/(*C,R*)-$\mathbf{L_{BB2}}$RuCp(NCMe) including probability ellipsoids at 50%. The PF_6^- counterion is omitted for clarity.

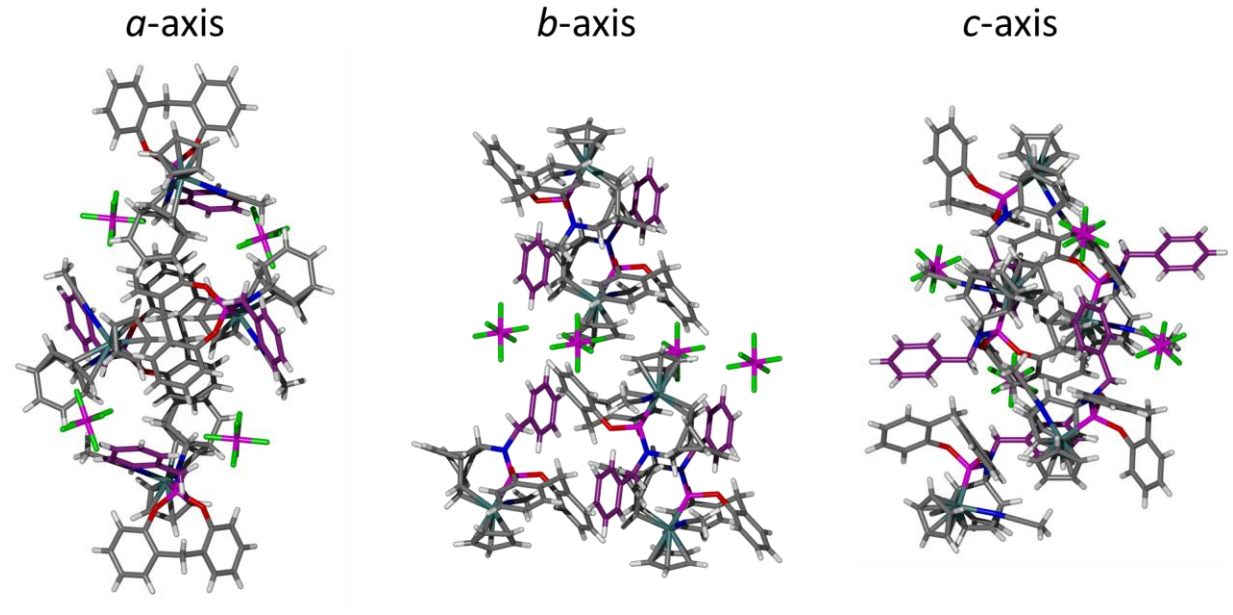

Figure S95. Solid-state superstructure of (*A,S*)/(*C,R*)-$\mathbf{L_{BB2}}$RuCp(NCMe)·PF_6 viewed along the three unit cell axes.

Crystal data for (*A,S*)/(*C,R*)-$\mathbf{L_{BB1}}$RuCp(NCMe)·PF_6: $C_{36}H_{34}F_6N_2O_2P_2Ru$, M = 803.66, crystal system = monoclinic, space group = P2_1/n, a = 12.8456(5), b = 13.6849(6), c = 19.4533(8) Å, α =

90°, β = 108.231(2)°, γ = 90°, U = 3248.0(2) Å^3, F(000) = 1632.0, Z = 4, D_c = 1.643 mg m^{-3}, μ = 0 0.654 mm^{-1}, (Mo-Kα, λ = 0.71073 Å), T = 120(1) K. 77345 reflections were collected yielding 9443 unique data (R_{merg} = 0.0414). Final $wR_2(F^2)$ = 0.0730 for all data (443 refined parameters), conventional $R_1(F)$ = 0.0296 for 8513 reflections with I ≥ 2σ, GOF = 1.047. Crystallographic data for the structure has been deposited within the Cambridge Crystallographic Data Centre as supplementary publication CCDC-2173984.

9. *In Silico* Modelling

9.1 General Methods

DFT calculations were carried out in Gaussian 16.[20] The minimum energy (ME) geometries and transition state (TS) geometries of the barbaralane isomers were optimised using the range-separated hybrid (RSH) general gradient approximation GGA functional ωB97XD.[21] We selected this functional as it includes dispersion correction and we have previously found that it performs well in estimating the equilibrium distribution of barbaralanes.[22] Calculations were performed using the 6-311++G(d,p) basis set for H, C, N, O, P, Cl atoms and SDD basis set for Ru, Au, and Pd atoms, with a polarizable continuum model (PCM) using the integral equation formalism variant (IEFPCM) to approximate CS_2 as the solvent. CS_2 was chosen as the solvent model to allow for comparison of the calculated data for **2** with the experimental results in Figure 3c, then subsequently to allow a consistent comparison of the calculated data of other compounds with **2**. Frequency calculations were carried out to confirm the lack of any negative vibrational frequencies for ME structures and to confirm their presence for TS structures (saddle points). Intrinsic rection coordinate (IRC) calculations[23,24] were performed to confirm that TS geometries identified lie on a potential surface linking the two barbaralane ME structures. For compounds with rotatable single bonds that lead to

several accessible conformers, (**2**, **8**, and **L_{BB1}**) a molecular mechanics conformational search was performed to identify conformers that were then taken on to DFT optimisation. Conformational searches were performed using confab[25] implemented in Open Babel,[26,27] identifying all conformers predicted to be within 15 kcal·mol^{-1} of the lowest energy structure and with a root mean square deviation of 1.0 Å or more. The conformers of **L_{BB1}**AuCl screened by DFT were prepared manually based on the conformers of **L_{BB1}** identified by confab. The free energy gaps (ΔG_{calc}) reported in Table S7 correspond to the difference in energy between the lowest energy conformers of the relevant configurational isomers. The relative energies given for **L_{BB1}**$RuCp^+$ and **L_{BB1}**$RuCp(NCMe)^+$ take into account the calculated energy of a 'free' MeCN molecule at infinite separation in the ligand coordination reaction of **L_{BB1}**$RuCp^+$ and MeCN giving **L_{BB1}**$RuCp(NCMe)^+$.

Table S7. Energetics of the dynamic sp^3-carbon equilibria.^{a}

Compounds	Major Isomer^b	ΔG_{calc} (ΔG_{exp}) kJ·mol^{-1}	$\Delta G^{\ddagger}_{calc}$ ($\Delta G^{\ddagger}_{exp}$) kJ·mol^{-1}
**BB**	–^{c}	0.0 (0.0)	38.5 (32.3^d)
**1**	–^{e}	0.0	38.0
(*R*,*S*)/(*S*,*S*)-**2**	*S*,*S*	4.5	35.5
**3**	-^{c}	0.0 (0.0^f)	47.8 (42.8^f)
(*R*,*R*)/*meso*/(*S*,*S*)-**4**	*S*,*S*/*R*,*R*^{g}	0.7	37.6
(*R*,*S*)/(*S*,*S*)-**8**	*R*,*S*	3.8	39.5
**L_{BB1}**	*S*,*S*	20.2	37.7
**L_{BB1}**AuCl	*R*,*S*	1.1	46.7
**L_{BB1}**$PdCl_2$	*C*,*R*,*S*	1.8 (0.5^h)	60.6 (54.6^h)
**L_{BB1}**$RuCp(NCMe)^+$	*C*,*R*,*S*	2.8 (4.0^i)	85.4^j (87.8^i)
**L_{BB1}**$RuCp^+$	*S*,*S*,*S*	25.7	31.8

^{a}The DFT calculated free energies ΔG_{calc} and $\Delta G^{\ddagger}_{calc}$ were obtained using the ωB97X-D functional, 6-311++G(d,p) basis set for H, C, N, O, P, Cl atoms and SDD basis set for Au, Pd, and Ru atoms, with a polarizable continuum model (PCM) using the integral equation formalism variant (IEFPCM) to approximate CS_2 as the solvent. ΔG_{exp} and $\Delta G^{\ddagger}_{exp}$ values were measured from NMR spectra in the slow exchange regime at suitably low temperatures. ^{b}Structure predicted to be lower in energy by DFT. ^{c}Achiral. ^{d}Reported in ref 28. ^{e}The two enantiomers are degenerate. ^{f}Reported in ref 29. ^{g}The enantiomeric pair of chiral stereoisomers is predicted to be lower in energy than the *meso* form. ^{h}Measured at 240 K in $CDCl_3$. ^{i}Measured at 298 K in $CDCl_3$. ^{j}Barrier predicted for coordination-coupled Cope (cc-Cope) rearrangement of **L_{BB1}**$RuCp(NCMe)^+$.

First principles calculation for the NMR shifts the crystalline form of (*R*,*R*)-**2** was carried out using the GIPAW method implemented in CASTEP v17.2.[30] The calculation was performed using the PBE functional[31] and on-the-fly generated ultrasoft pseudopotentials with a cut-off energy of 600 eV. Geometry optimisation of all atomic positions was carried out with the centre of mass and unit cell parameters fixed at the values determined by single-crystal X-ray diffraction. Integrals were taken over the Brillouin zone using a Monkhorst-Pack grid with a maximum *k*-point sample spacing of 0.1 $Å^{-1}$. ^{13}C isotropic shifts were obtained from calculations of NMR parameters from the optimised structures using tools relying on the magres file format and MagresPython library.[32]

9.2 Optimised Structures

Cartesian coordinates are given in Å, self-consistent field (SCF) Gibbs free energies (*G*) are given in Hartrees, and imaginary vibrational frequencies (*v*) are given in cm^{-1}.

BB

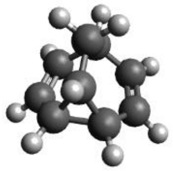

Table S8. Coordinates and energy for the optimised geometry of **BB**.

G = -348.756265			
		**Coordinates / Å**	
**Atom**	**x**	**y**	**z**
C	0.26283	0.00002	1.56714
C	1.33011	-0.00001	0.45155
C	-1.11592	0.00002	0.95427
C	-1.27200	-0.78622	-0.31916
C	-0.10814	-1.53378	-0.81916
C	1.13281	-1.20089	-0.44632
H	-0.28197	-2.34535	-1.51830
H	1.99888	-1.72691	-0.83420
C	1.13283	1.20086	-0.44636
H	2.32946	-0.00000	0.88919
C	-0.10811	1.53376	-0.81919
H	1.99892	1.72683	-0.83425
C	-1.27199	0.78624	-0.31917

H	-0.28192	2.34536	-1.51829
H	-1.95646	-0.00003	1.63873
H	-2.24529	1.21815	-0.51838
H	-2.24529	1.21813	-0.51842
H	0.39453	-0.88568	2.19615
H	0.39456	0.88576	2.19612

TS-**BB**

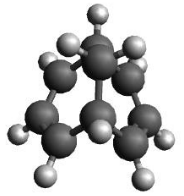

Table S9. Coordinates, energy, and imaginary vibrational frequency for the optimised geometry of TS-**BB**.

G = -348.741470 v = -406.10			
	Coordinates / Å		
**Atom**	**x**	**y**	**z**
C	-0.00003	-0.00083	1.60724
C	-1.24234	-0.00025	0.72750
C	1.24234	-0.00011	0.72751
C	1.20725	1.00944	-0.36990
C	-0.00009	1.50618	-0.84041
C	-1.20739	1.00927	-0.36992
H	-0.00014	2.19195	-1.68070
H	-2.14652	1.35996	-0.78047
C	-1.20729	-1.00905	-0.37064
H	-2.16971	-0.00047	1.29712
C	0.00011	-1.50548	-0.84143
H	-2.14636	-1.35963	-0.78141
C	1.20745	-1.00895	-0.37060
H	0.00015	-2.19081	-1.68208
H	2.16973	-0.00007	1.29709
H	2.14656	-1.35945	-0.78135
H	2.14634	1.36028	-0.78042
H	0.00000	-0.88772	2.24713
H	-0.00006	0.88466	2.24901

1

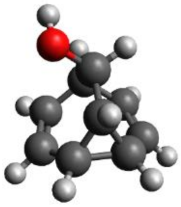

Table S10. Coordinates and energy for the optimised geometry of **1**.

	G = -423.973863		
		**Coordinates / Å**	
**Atom**	**x**	**y**	**z**
C	1.00325	0.05344	-0.84588
C	0.42154	1.22110	-0.01720
C	0.14821	-1.16971	-0.66435
C	-1.32790	-0.92275	-0.55854
C	-1.81422	0.46115	-0.68543
C	-0.99705	1.49639	-0.46676
H	-2.86128	0.61702	-0.92281
H	-1.35165	2.52006	-0.51594
C	0.30519	0.83398	1.43916
H	1.04972	2.10609	-0.14410
C	-0.15684	-0.38126	1.74744
H	0.52885	1.56726	2.20606
C	-0.47681	-1.35325	0.69231
H	-0.32125	-0.67256	2.77933
H	0.47745	-2.05698	-1.19152
H	-0.66039	-2.37185	1.01086
H	-1.98193	-1.70178	-0.93005
O	2.32661	-0.28444	-0.45679
H	0.99003	0.34834	-1.90384
H	2.88533	0.48547	-0.57809

TS-**1**

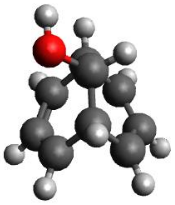

Table S11. Coordinates, energy, and imaginary vibrational frequency for the optimised geometry of TS-**1**.

	G = -423.959254		
	v = -399.12		
		**Coordinates / Å**	
**Atom**	**x**	**y**	**z**
C	1.05389	-0.01369	-0.80919
C	0.33281	1.23574	-0.32169

C	0.28923	-1.24304	-0.35628
C	-1.17093	-1.17847	-0.64890
C	-1.81036	0.04151	-0.80736
C	-1.12854	1.23473	-0.61380
H	-2.88507	0.06296	-0.94955
H	-1.63956	2.18633	-0.68981
C	-0.07403	1.19006	1.11154
H	0.84405	2.15236	-0.61535
C	-0.24193	-0.02203	1.76397
H	-0.21724	2.12691	1.63561
C	-0.11152	-1.21934	1.08019
H	-0.59543	-0.02956	2.78888
H	0.77377	-2.16435	-0.67208
H	-0.28435	-2.16475	1.57916
H	-1.71607	-2.10866	-0.74820
O	2.36914	-0.11194	-0.28650
H	1.08277	-0.00092	-1.90661
H	2.85226	0.68232	-0.52097

(*S*,*S*)-**2**

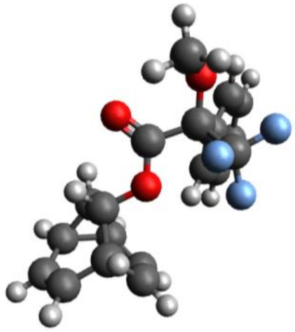

Table S12. Coordinates and energy for the optimised geometry of (*S*,*S*)-**2**.

G = -1259.080918			
	**Coordinates / Å**		
**Atom**	**x**	**y**	**z**
C	2.03331	-0.81625	-0.56427
C	2.91285	-1.08586	0.66236
C	2.35907	0.52104	-1.16938
C	3.81288	0.89616	-1.13390
C	4.77246	-0.07391	-0.58073
C	4.36678	-1.04344	0.24431
H	5.82032	0.04120	-0.83535
H	5.06440	-1.74229	0.69110
C	2.76169	0.03127	1.67190
H	2.64729	-2.05167	1.09232
C	2.72945	1.29489	1.23925
H	2.75121	-0.21018	2.72828
C	2.76602	1.59894	-0.19969

H	2.69414	2.12419	1.93740
H	1.78658	0.80703	-2.04197
H	2.53780	2.61771	-0.48708
H	4.16830	1.52197	-1.94262
O	0.66534	-0.83255	-0.08264
H	2.14453	-1.61560	-1.29888
C	-0.31152	-0.84937	-0.97317
O	-0.17734	-0.96466	-2.16021
C	-1.69428	-0.62442	-0.30512
O	-2.71069	-1.09315	-1.12761
C	-2.75736	-2.47728	-1.45758
H	-3.43569	-2.55043	-2.30577
H	-3.15214	-3.06776	-0.62715
H	-1.77748	-2.85799	-1.75339
C	-1.78212	-1.31334	1.09317
F	-1.34904	-2.58895	1.04535
F	-3.05781	-1.34619	1.49901
F	-1.07953	-0.69960	2.04638
H	-3.83344	0.85694	-1.05227
C	-3.07202	1.47592	-0.59898
C	-3.26496	2.84667	-0.47182
H	-4.18848	3.29018	-0.82634
C	-2.28293	3.64423	0.10208
H	-2.43501	4.71346	0.19772
C	-1.10416	3.06192	0.55291
H	-0.33063	3.67291	1.00462
C	-0.90659	1.69291	0.43160
H	0.01489	1.25681	0.79498
C	-1.89154	0.89140	-0.14773

(*S*,*R*)-**2**

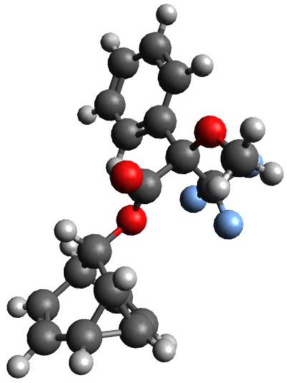

Table S13. Coordinates and energy for the optimised geometry of (*S*,*R*)-**2**.

G = -1259.079182			
		**Coordinates / Å**	

Atom	x	y	z
C	1.94555	0.63727	-0.71704
C	2.84147	-0.54078	-0.97532
C	2.55646	1.56338	0.34159
C	4.31265	-0.24217	-0.92985
9	3.60765	-1.05221	0.21526
H	2.49486	-1.25472	-1.71129
O	0.67532	0.19103	-0.17773
H	1.74699	1.18731	-1.63836
C	-0.21220	-0.31982	-1.01340
O	-0.07154	-0.43975	-2.19931
C	-1.52109	-0.72414	-0.28248
O	-2.19387	-1.69781	-1.01159
C	-1.56486	-2.95332	-1.24350
H	-2.14076	-3.42346	-2.03860
H	-1.59858	-3.58344	-0.35142
H	-0.53149	-2.83527	-1.57715
C	-1.23192	-1.24538	1.16179
F	-0.23467	-2.14969	1.17620
F	-2.32271	-1.85559	1.64494
F	-0.90278	-0.28335	2.02509
H	-0.91647	1.85982	0.48992
C	-1.95553	1.74447	0.20935
C	-2.80613	2.84018	0.27377
H	-2.41810	3.79960	0.59624
C	-4.14598	2.70802	-0.07142
H	-4.80873	3.56454	-0.02155
C	-4.62959	1.47226	-0.48205
H	-5.67288	1.35873	-0.75378
C	-3.78164	0.37303	-0.55017
H	-4.15621	-0.58708	-0.87630
C	-2.43904	0.50349	-0.20561
C	4.73701	1.14995	-0.70742
H	4.94617	-0.84553	-1.56760
C	3.91168	2.03436	-0.14004
H	5.74800	1.42730	-0.98506
H	4.22039	3.05246	0.06711
C	3.36093	-0.43048	1.52562
H	3.85267	-2.10685	0.21741
C	2.83428	0.79342	1.61397
H	3.63417	-0.98748	2.41492
H	2.66071	1.27240	2.57041
H	1.88321	2.40252	0.51994

TS-(*S*)-**2**

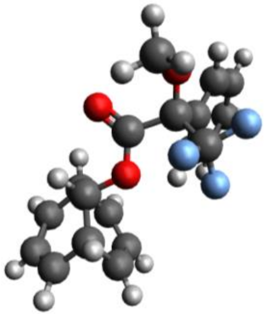

Table S14. Coordinates, energy, and imaginary vibrational frequency for the optimised geometry of TS-(*S*)-**2**.

	G = -1259.067269		
	v = -400.16		
		**Coordinates / Å**	
**Atom**	**x**	**y**	**z**
C	2.04834	-0.75570	-0.58289
C	2.92136	-1.08974	0.60647
C	2.40609	0.61309	-1.13257
C	3.87155	0.78851	-1.33993
C	4.78808	0.02817	-0.63043
C	4.37078	-0.85826	0.35115
H	5.84710	0.22405	-0.75257
H	5.08631	-1.41897	0.93847
C	3.04142	0.01664	1.59909
H	2.68339	-2.06032	1.03517
C	2.81319	1.33369	1.23110
H	3.28958	-0.24115	2.62059
C	2.54511	1.66750	-0.08705
H	2.96415	2.12213	1.95945
H	1.79402	0.89342	-1.98565
H	2.40414	2.69900	-0.38400
H	4.19666	1.51308	-2.07529
O	0.68831	-0.75872	-0.09047
H	2.13672	-1.51456	-1.36156
C	-0.29557	-0.80623	-0.97508
O	-0.16309	-0.91187	-2.16284
C	-1.68407	-0.64916	-0.29907
O	-2.67725	-1.17620	-1.11486
C	-2.65271	-2.56224	-1.43873
H	-3.33406	-2.67509	-2.28008
H	-3.00750	-3.16958	-0.60251
H	-1.65682	-2.89161	-1.74279
C	-1.72681	-1.33608	1.10247
F	-1.22818	-2.58768	1.05765
F	-2.99639	-1.43351	1.51663

F	-1.05108	-0.68128	2.04743
H	-3.91380	0.70189	-1.02523
C	-3.18513	1.36573	-0.58157
C	-3.45700	2.72276	-0.45243
H	-4.40919	3.11081	-0.79596
C	-2.51680	3.57729	0.10959
H	-2.73048	4.63579	0.20671
C	-1.30035	3.06613	0.54621
H	-0.55829	3.72180	0.98774
C	-1.02427	1.71081	0.42333
H	-0.07342	1.33207	0.77505
C	-1.96677	0.85272	-0.14427

3

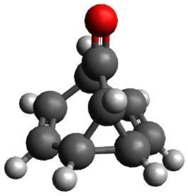

Table S15. Coordinates and energy for the optimised geometry of **3**.

G = -422.787511			
		**Coordinates / Å**	
**Atom**	**x**	**y**	**z**
C	1.26106	-0.31720	-0.00067
C	0.70430	1.10021	-0.00000
C	0.21085	-1.35697	-0.00081
C	-1.04473	-0.98472	-0.77166
C	-1.04563	0.28425	-1.52534
C	-0.21036	1.27317	-1.20246
H	-1.76948	0.40063	-2.32400
H	-0.22286	2.22853	-1.71407
C	-0.20915	1.27215	1.20360
H	1.52999	1.80889	-0.00008
C	-1.04411	0.28295	1.52644
H	-0.22114	2.22708	1.71602
C	-1.04396	-0.98537	0.77169
H	-1.76721	0.39866	2.32587
H	0.54190	-2.38635	-0.00137
H	-1.57546	-1.81638	1.21752
H	-1.57668	-1.81534	-1.21767
O	2.44890	-0.55707	-0.00086

TS-**3**

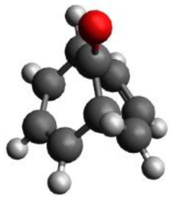

Table S16. Coordinates, energy, and imaginary vibrational frequency for the optimised geometry of TS-**3**.

	G = -422.769140		
	v = -439.89		
		Coordinates / Å	
**Atom**	**x**	**y**	**z**
C	-1.32015	-0.00005	-0.00025
C	-0.49249	1.24931	-0.00015
C	-0.49252	-1.24940	-0.00029
C	0.62858	-1.20593	0.99571
C	1.09692	-0.00006	1.49299
C	0.62840	1.20582	0.99607
H	1.94461	-0.00008	2.16789
H	1.02862	2.15166	1.33661
C	0.62882	1.20597	-0.99576
H	-1.09468	2.15214	-0.00014
C	1.09763	0.00016	-1.49251
H	1.02928	2.15182	-1.33599
C	0.62898	-1.20578	-0.99571
H	1.94572	0.00021	-2.16691
H	-1.09460	-2.15230	-0.00044
H	1.02954	-2.15153	-1.33610
H	1.02877	-2.15177	1.33628
O	-2.53028	-0.00005	-0.00022

(*R*,*R*)-**4**

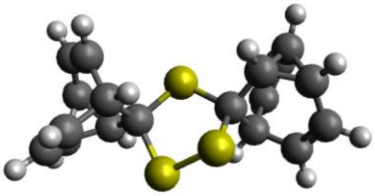

Table S17. Coordinates and energy for the optimised geometry of (*R*,*R*)-**4**.

	G = -1889.718361		
		Coordinates / Å	
**Atom**	**x**	**y**	**z**
S	-0.79874	-1.74869	-0.65801
S	0.79860	-1.74855	0.65764
C	-1.44024	-0.10308	-0.15050
C	1.44023	-0.10305	0.15000
S	0.00005	1.05855	-0.00101

C	-2.35128	0.38949	-1.24932
C	-2.22936	-0.16252	1.18104
C	2.35057	0.38978	1.24927
C	2.23004	-0.16282	-1.18105
H	-1.90367	0.47231	-2.23228
C	-3.35363	1.44057	-0.85075
C	-3.79578	-0.03220	-1.16192
C	-2.77467	1.21114	1.50762
H	-1.57174	-0.52619	1.96980
C	-3.45604	-1.03520	1.03242
H	1.90223	0.47270	2.23189
C	3.35291	1.44091	0.85108
C	3.79506	-0.03208	1.16284
C	2.77556	1.21078	-1.50766
H	1.57286	-0.52669	-1.97005
C	3.45672	-1.03538	-1.03158
H	-3.62943	2.15291	-1.61803
C	-3.33423	1.93502	0.53371
C	-4.20073	-0.93058	-0.07136
H	-4.31885	-0.14235	-2.10360
H	-2.76574	1.55038	2.53690
H	-3.74548	-1.67659	1.85690
H	3.62851	2.15336	1.61835
C	3.33433	1.93499	-0.53353
C	4.20069	-0.93060	0.07267
H	4.31776	-0.14176	2.10480
H	2.76755	1.54963	-2.53708
H	3.74683	-1.67667	-1.85591
H	-3.80090	2.89119	0.74259
H	-5.12446	-1.48759	-0.18140
H	3.80096	2.89118	-0.74238
H	5.12420	-1.48778	0.18349

(*R*,*S*)-**4**

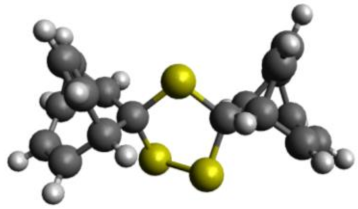

Table S18. Coordinates and energy for the optimised geometry of (*R*,*S*)-**4**.

G = -1889.718076			
	Coordinates / Å		
**Atom**	**x**	**y**	**z**
S	-0.80489	-1.74870	-0.69909

S	0.80397	-1.76173	0.60384
C	-1.44206	-0.10571	-0.17833
C	1.43703	-0.10729	0.10257
S	-0.00095	1.05562	-0.04155
C	-2.36619	0.39096	-1.26417
C	-2.21557	-0.17084	1.16238
C	2.37304	0.40056	1.22783
C	2.19855	-0.17084	-1.19591
H	-1.93047	0.47794	-2.25213
C	-3.36455	1.43987	-0.84961
C	-3.80933	-0.03178	-1.16144
C	-2.75762	1.20121	1.50078
H	-1.54858	-0.53723	1.94200
C	-3.44329	-1.04379	1.02457
H	1.81807	0.44473	2.16444
C	2.92894	1.75845	0.85390
C	3.58961	-0.49434	1.33876
C	3.21199	0.91674	-1.41901
H	1.63497	-0.51792	-2.05265
C	3.64685	-0.56316	-1.09906
H	-3.64965	2.15518	-1.61068
C	-3.32905	1.92873	0.53651
C	-4.20099	-0.93492	-0.06990
H	-4.34315	-0.13860	-2.09746
H	-2.73657	1.53636	2.53121
H	-3.72267	-1.68869	1.84976
H	3.04519	2.50679	1.62897
C	3.35040	1.96430	-0.39749
C	4.19352	-0.90728	0.22014
H	3.99165	-0.71249	2.32140
H	3.38906	1.21228	-2.44544
H	4.06215	-1.08964	-1.94922
H	-3.79343	2.88394	0.75469
H	-5.12565	-1.49198	-0.17149
H	3.82248	2.89843	-0.68099
H	5.10879	-1.48735	0.26123

TS-**4**

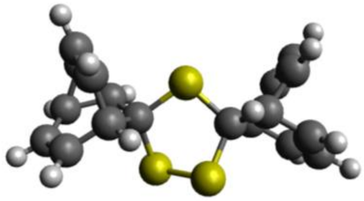

Table S19. Coordinates, energy, and imaginary vibrational frequency for the optimised geometry of TS-**4**.

G = -1889.703900
v = -395.21

	Coordinates / Å		
Atom	x	y	z
S	-0.80980	-1.75668	-0.66516
S	0.79492	-1.75371	0.64286
C	-1.44360	-0.10534	-0.16731
C	1.42728	-0.11059	0.12565
S	0.00082	1.05437	-0.04623
C	-2.36455	0.37879	-1.26144
C	-2.21877	-0.14960	1.17313
C	2.34728	0.40215	1.23146
C	2.21210	-0.18580	-1.17977
H	-1.92711	0.45097	-2.24983
C	-3.36039	1.43622	-0.86267
C	-3.80906	-0.03814	-1.15476
C	-2.75738	1.22847	1.49171
H	-1.55406	-0.50713	1.95877
C	-3.44872	-1.02098	1.04543
H	1.83681	0.47420	2.18887
C	3.11622	1.62207	0.85132
C	3.67829	-0.27188	1.28060
C	2.97769	1.05597	-1.48783
H	1.60114	-0.55171	-2.00103
C	3.54476	-0.84249	-1.05610
H	-3.64200	2.14182	-1.63401
C	-3.32529	1.94415	0.51662
C	-4.20463	-0.92514	-0.05146
H	-4.34178	-0.15643	-2.09001
H	-2.73680	1.57766	2.51746
H	-3.73088	-1.65366	1.87906
H	3.44788	2.27693	1.64652
C	3.36475	1.92346	-0.47881
C	4.20505	-0.89623	0.16051
H	4.20503	-0.27899	2.22622
H	3.20208	1.27170	-2.52464
H	3.96841	-1.30009	-1.94108
H	-3.78716	2.90363	0.72084
H	-5.13061	-1.48107	-0.14665
H	3.97100	2.78804	-0.72283
H	5.19685	-1.33021	0.21095

(*R*,*S*)-**8**

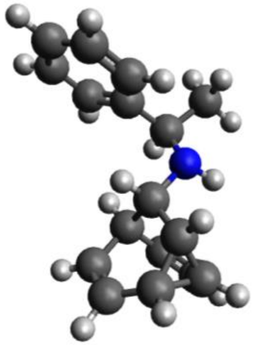

Table S20. Coordinates and energy for the optimised geometry of (*R*,*S*)-**8**.

	G = -713.616216		
		Coordinates / Å	
**Atom**	**x**	**y**	**z**
C	-0.82095	-0.08555	-0.41522
C	-1.99893	-0.10699	-1.35844
C	-1.32491	-0.37756	1.02774
C	-3.09584	-1.07922	-1.05362
C	-3.28733	0.46388	-0.81589
H	-1.76885	0.08473	-2.39975
N	-0.12653	1.18648	-0.58654
H	-0.12900	-0.88982	-0.68676
C	-2.91713	-1.99391	0.08479
H	-3.66105	-1.45889	-1.89591
C	-2.05692	-1.69955	1.06431
H	-3.52186	-2.89396	0.12158
H	-1.93818	-2.34876	1.92534
C	-3.28530	1.01588	0.54780
H	-3.95745	0.93232	-1.52622
C	-2.34812	0.65706	1.43359
H	-4.07900	1.70324	0.82199
H	-2.35348	1.04320	2.44750
H	-0.48144	-0.39466	1.71887
C	0.99762	1.44477	0.30800
H	-0.79709	1.94300	-0.50200
C	1.53214	2.85057	0.03302
H	1.87197	2.93234	-1.00253
H	2.37081	3.07698	0.69520
H	0.75080	3.59818	0.20216
H	4.93531	-2.19353	-0.34940
C	4.14133	-1.46694	-0.21709
C	3.53122	-0.88620	-1.32606
H	3.85021	-1.16109	-2.32568
C	2.51267	0.04230	-1.15720
H	2.02504	0.48596	-2.01867
C	2.08668	0.40482	0.12119

C	2.69822	-0.18505	1.22371
H	2.36889	0.08248	2.22371
C	3.72216	-1.11386	1.05943
H	4.18706	-1.56478	1.92936
H	0.69335	1.41107	1.36665

(*S*,*S*)-**8**

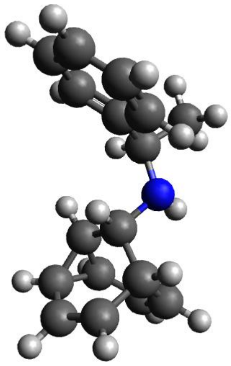

Table S21. Coordinates and energy for the optimised geometry of (*S*,*S*)-**8**.

	G = -713.614751		
		**Coordinates / Å**	
**Atom**	**x**	**y**	**z**
C	-0.81203	-0.11443	-0.21670
C	-1.89192	-0.36524	-1.29171
C	-1.46587	-0.12909	1.15306
C	-2.60975	-1.08059	1.34093
C	-2.96805	-1.96929	0.22487
C	-2.60929	-1.66887	-1.02740
H	-3.55460	-2.85587	0.44202
H	-2.89609	-2.29610	-1.86464
C	-2.97219	0.69198	-1.19983
H	-1.41734	-0.36102	-2.27306
C	-3.42416	1.04956	0.00797
H	-3.41241	1.08822	-2.10837
C	-2.84406	0.47348	1.23098
H	-4.24799	1.74821	0.11171
H	-0.81376	0.01696	2.00608
H	-3.13256	0.93510	2.16749
H	-2.76619	-1.47082	2.33934
N	-0.10163	1.12227	-0.53191
H	-0.09056	-0.93561	-0.27345
C	1.04186	1.43628	0.31881
H	-0.75754	1.89609	-0.51264
C	1.55525	2.83118	-0.03977
H	1.85937	2.86498	-1.08908

H	2.41475	3.09360	0.58095
H	0.77505	3.58194	0.12027
H	4.33393	-1.43209	1.99255
C	3.83101	-1.03874	1.11581
C	2.79927	-0.11588	1.26398
H	2.50184	0.20448	2.25843
C	2.13876	0.39991	0.15253
C	2.52325	-0.03132	-1.11755
H	1.99651	0.35426	-1.98383
C	3.54976	-0.95403	-1.27006
H	3.83647	-1.28269	-2.26324
C	4.20900	-1.46042	-0.15282
H	5.00886	-2.18280	-0.27222
H	0.76873	1.45031	1.38647

TS-(*S*)-**8**

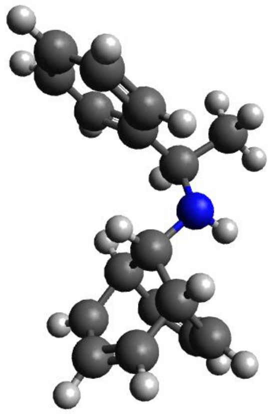

Table S22. Coordinates, energy, and imaginary vibrational frequency for the optimised geometry of TS-(*S*-)**8**.

	G = -713.601014		
	v = -393.68		
		**Coordinates / Å**	
**Atom**	**x**	**y**	**z**
C	-0.80297	-0.11371	-0.33887
C	-1.95085	-0.20365	-1.33559
C	-1.36519	-0.30156	1.07318
C	-2.31029	-1.44224	1.21761
C	-2.99411	-1.94781	0.12137
C	-2.87728	-1.34812	-1.12222
H	-3.72604	-2.73447	0.26721
H	-3.45064	-1.70803	-1.96773
C	-3.11879	0.66072	-0.98131
H	-1.60621	-0.08733	-2.36071
C	-3.34787	1.03764	0.33393
H	-3.77480	0.99531	-1.77544
C	-2.54881	0.55885	1.36482
H	-4.23722	1.60976	0.57466

H	-0.59647	-0.27940	1.84275
H	-2.76451	0.81677	2.39459
H	-2.43928	-1.88162	2.19942
N	-0.11002	1.15179	-0.55242
H	-0.10008	-0.92772	-0.54135
C	1.02265	1.42983	0.32555
H	-0.77906	1.91176	-0.48421
C	1.54479	2.83501	0.02464
H	1.87077	2.90415	-1.01627
H	2.39085	3.07506	0.67237
H	0.76151	3.58053	0.19373
H	4.26632	-1.52203	1.94591
C	3.78220	-1.09150	1.07613
C	2.75301	-0.16865	1.24124
H	2.43913	0.11480	2.24184
C	2.11694	0.39496	0.13883
C	2.52346	0.01198	-1.13990
H	2.01661	0.43527	-2.00050
C	3.54713	-0.91079	-1.30944
H	3.85087	-1.20180	-2.30924
C	4.18192	-1.46510	-0.20081
H	4.97981	-2.18730	-0.33357
H	0.73299	1.40855	1.38844

(*R*,*S*)-$\mathbf{L_{BB1}}$

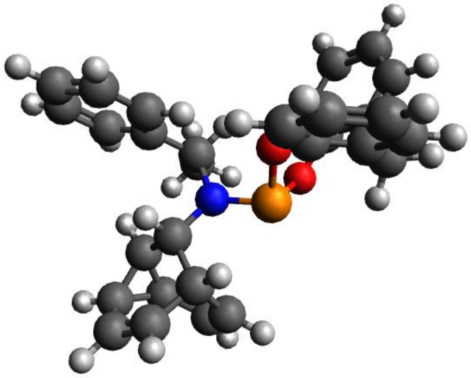

Table S23. Coordinates and energy for the optimised geometry of (*R*,*S*)-$\mathbf{L_{BB1}}$.

	G = -1706.031741		
	**Coordinates / Å**		
**Atom**	**x**	**y**	**z**
H	-0.07207	1.73092	0.30123
C	0.98434	1.47049	0.40508
C	1.64127	2.00491	-0.86063
C	2.74874	2.84670	-0.84411
C	3.31083	3.30642	-2.03332
C	2.76866	2.92962	-3.25399
C	1.65421	2.09428	-3.28077

C	1.09704	1.64000	-2.09490
C	1.45139	2.11091	1.71275
N	1.02683	-0.01209	0.46343
C	2.24782	-0.70946	0.00668
C	2.14049	-2.25194	0.13141
H	1.27324	-2.61649	-0.41984
C	2.07742	-2.66824	1.58376
C	2.89496	-2.07503	2.45931
C	3.79819	-0.99560	2.03416
H	4.29630	-0.44149	2.82033
C	3.47569	-0.25596	0.76769
C	4.66743	-1.16537	0.72969
C	4.58656	-2.38903	-0.07642
C	3.39693	-2.89900	-0.40511
H	2.40175	-0.48514	-1.05494
P	-0.39769	-0.86144	0.66647
O	-1.11138	0.08633	1.85565
C	-2.25447	0.83815	1.73212
C	-3.46135	0.27219	1.31441
C	-4.58219	1.09815	1.24430
C	-4.52074	2.44016	1.59383
C	-3.31450	2.98011	2.02559
C	-2.18243	2.18051	2.09135
C	-3.56709	-1.18633	0.93355
C	-3.40593	-1.43628	-0.55258
C	-2.28408	-0.98355	-1.25536
C	-2.15715	-1.20480	-2.62223
C	-3.15193	-1.88224	-3.31371
C	-4.27305	-2.34388	-2.63470
C	-4.38939	-2.11727	-1.26866
O	-1.29380	-0.26075	-0.64118
H	3.18421	3.15838	0.09859
H	4.17428	3.96180	-2.00070
H	3.20644	3.28625	-4.17963
H	1.21800	1.80108	-4.22950
H	0.22851	0.98927	-2.10705
H	1.26192	3.18824	1.69272
H	2.50997	1.95336	1.91833
H	0.88770	1.67031	2.53529
H	1.42263	-3.48154	1.87490
H	2.92962	-2.39371	3.49554
H	3.70079	0.79965	0.73025
H	5.63811	-0.68926	0.79334
H	5.50886	-2.87650	-0.37367
H	3.30779	-3.81896	-0.97208
H	-5.52343	0.67019	0.91276

H	-5.40913	3.05816	1.53577
H	-3.25141	4.02454	2.30923
H	-1.23362	2.58285	2.42374
H	-4.54746	-1.55832	1.23819
H	-2.83523	-1.77229	1.49736
H	-1.27272	-0.83536	-3.12824
H	-3.04853	-2.04891	-4.37987
H	-5.05534	-2.87481	-3.16450
H	-5.26822	-2.47241	-0.73960

(*S*,*S*)-**L**$_{\mathbf{BB1}}$

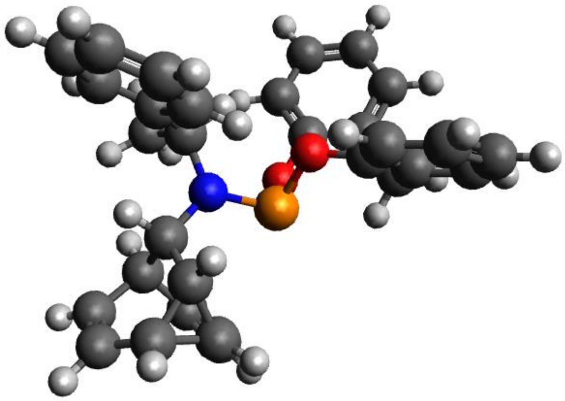

Table S24. Coordinates and energy for the optimised geometry of (*S*,*S*)-**L**$_{\mathbf{BB1}}$.

G = -1706.039523			
		**Coordinates / Å**	
**Atom**	**x**	**y**	**z**
H	-0.09359	1.67859	0.80472
C	0.97468	1.44594	0.81387
C	1.60879	2.30696	-0.27652
C	2.54955	3.29712	-0.00646
C	3.09738	4.06013	-1.03621
C	2.71092	3.84221	-2.35066
C	1.76756	2.85645	-2.63115
C	1.22471	2.09981	-1.60391
C	1.49053	1.73344	2.22235
N	1.08004	0.01546	0.46744
C	2.43157	-0.52540	0.27505
C	2.52362	-1.46364	-0.90567
H	2.11651	-1.08736	-1.83729
C	2.39063	-2.94033	-0.62449
C	2.09376	-3.36077	0.75133
C	2.34665	-2.55041	1.78427
C	3.02821	-1.22759	1.52595
C	4.47478	-1.57498	1.24771
C	4.79711	-2.10220	0.06258
C	3.77401	-2.29123	-0.97927

H	3.07003	0.33651	0.05647
P	-0.27184	-0.95291	0.33045
O	-1.05583	-0.57123	1.78026
C	-2.20863	0.15603	1.92480
C	-3.39871	-0.24361	1.31192
C	-4.53915	0.52846	1.52548
C	-4.51178	1.65558	2.33599
C	-3.32178	2.02789	2.95159
C	-2.17098	1.28076	2.74329
C	-3.45511	-1.46812	0.42916
C	-3.27101	-1.16123	-1.04340
C	-2.16404	-0.44714	-1.51513
C	-2.02319	-0.15771	-2.86796
C	-2.98525	-0.57783	-3.77592
C	-4.08963	-1.29313	-3.32954
C	-4.22191	-1.57489	-1.97553
O	-1.20719	0.04479	-0.66331
H	2.87032	3.48633	1.01092
H	3.83080	4.82464	-0.80424
H	3.13855	4.43423	-3.15210
H	1.45382	2.68058	-3.65455
H	0.48980	1.33252	-1.81558
H	1.23413	2.75168	2.52607
H	2.57577	1.62368	2.29235
H	1.02930	1.03455	2.92161
H	2.94290	-0.57218	2.39065
H	-5.46723	0.23085	1.04681
H	-5.41404	2.23581	2.49058
H	-3.28656	2.90178	3.59234
H	-1.23327	1.55535	3.21087
H	-4.42559	-1.95189	0.55788
H	-2.70765	-2.19580	0.75924
H	-1.15593	0.40456	-3.19323
H	-2.86984	-0.34557	-4.82848
H	-4.84700	-1.62678	-4.02930
H	-5.08854	-2.12698	-1.62549
H	4.13193	-2.54334	-1.96978
H	1.97674	-3.55081	-1.41732
H	1.66026	-4.34312	0.90392
H	2.13276	-2.85034	2.80395
H	5.20866	-1.46958	2.03944
H	5.80860	-2.43444	-0.14580

TS-(*S*)-**L**$_{\mathbf{BB1}}$

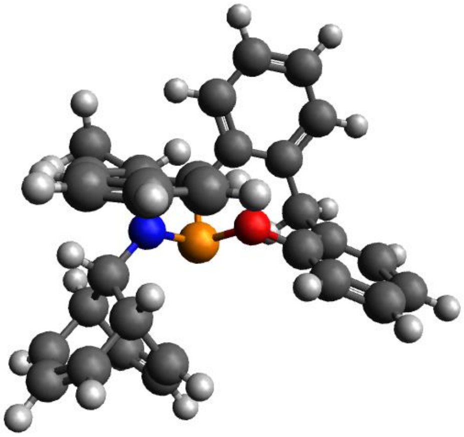

Table S25. Coordinates, energy, and imaginary vibrational frequency for the optimised geometry of TS-(*S*)**L**$_{\mathbf{BB1}}$.

	G = -1706.025012		
	v = -396.75		
		**Coordinates / Å**	
**Atom**	**x**	**y**	**z**
H	0.23050	1.99713	-0.25239
C	-0.83502	1.85523	-0.43533
C	-1.52249	2.09539	0.90150
C	-2.79174	2.65906	1.00316
C	-3.41217	2.79485	2.24216
C	-2.76348	2.37815	3.39736
C	-1.48604	1.83101	3.30801
C	-0.87378	1.69141	2.07032
C	-1.23849	2.82194	-1.54592
N	-0.99201	0.45232	-0.88720
C	-2.36295	-0.06994	-0.94425
C	-2.78342	-0.84434	0.30435
H	-2.56741	-0.30750	1.22318
C	-2.38733	-2.28190	0.32352
C	-2.10648	-2.97181	-0.85109
C	-2.25438	-2.35940	-2.07953
C	-2.65829	-0.92339	-2.17506
C	-4.06466	-1.42616	-2.19308
C	-4.77182	-1.59921	-1.01621
C	-4.16633	-1.38746	0.21892
H	-3.01624	0.80319	-1.01396
P	0.33469	-0.53705	-1.10137
O	1.34633	0.54188	-1.91714
C	2.49655	1.11536	-1.43813
C	3.56084	0.33948	-0.97060
C	4.70451	0.99935	-0.52364
C	4.80542	2.38387	-0.55439

C	3.74430	3.13732	-1.04404
C	2.59037	2.50323	-1.48222
C	3.49046	-1.17032	-0.94110
C	3.04042	-1.73702	0.39038
C	1.84230	-1.33249	0.98686
C	1.43525	-1.85072	2.21070
C	2.22545	-2.78564	2.86531
C	3.41982	-3.20394	2.29049
C	3.81472	-2.67923	1.06548
O	1.05322	-0.36735	0.41872
H	-3.31435	2.99331	0.11373
H	-4.40421	3.22880	2.30162
H	-3.24583	2.48480	4.36264
H	-0.96698	1.51246	4.20553
H	0.11055	1.24197	1.99341
H	-1.11299	3.85738	-1.21877
H	-2.27812	2.68993	-1.85650
H	-0.60665	2.64403	-2.41781
H	-2.34757	-0.43150	-3.09488
H	5.53450	0.40698	-0.15044
H	5.70905	2.87059	-0.20628
H	3.81156	4.21865	-1.08330
H	1.75056	3.07037	-1.86638
H	4.48001	-1.57289	-1.16744
H	2.83037	-1.52302	-1.73952
H	0.49765	-1.50583	2.63142
H	1.90678	-3.18659	3.82082
H	4.04361	-3.93406	2.79301
H	4.74962	-3.00379	0.61928
H	-4.70575	-1.56904	1.14000
H	-2.29341	-2.77157	1.28478
H	-1.86869	-4.02816	-0.80348
H	-2.07015	-2.89999	-2.99937
H	-4.51495	-1.65157	-3.15194
H	-5.76323	-2.03695	-1.05233

(*R*,*S*)-**L$_{BB1}$**AuCl

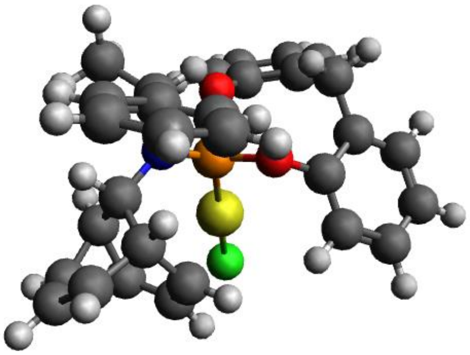

Table S26. Coordinates and energy for the optimised geometry of (*R*,*S*)-**L$_{BB1}$**AuCl.

	G = -2302.159281		
		**Coordinates / Å**	
**Atom**	**x**	**y**	**z**
H	1.44192	-2.30265	-1.38639
C	2.17504	-1.50332	-1.49013
C	3.39547	-1.93622	-0.69376
C	4.69596	-1.65657	-1.10293
C	5.78162	-2.01748	-0.30964
C	5.57713	-2.67201	0.89794
C	4.28023	-2.97084	1.30750
C	3.20087	-2.60494	0.51624
C	2.40903	-1.30379	-2.98414
N	1.54791	-0.27892	-0.90367
C	2.45357	0.86576	-0.68592
C	3.07515	0.94011	0.73440
H	3.44772	-0.03917	1.02392
C	2.08767	1.46823	1.74580
C	1.32433	2.51767	1.42306
C	1.37096	3.08961	0.06883
C	1.87849	2.21391	-1.05132
C	2.73356	3.38148	-0.63614
C	4.00369	3.11111	0.05943
C	4.19783	1.95441	0.69961
H	3.28660	0.68692	-1.36978
P	-0.00649	-0.37460	-0.38745
O	-0.61027	-1.39721	-1.51684
C	-1.97974	-1.55128	-1.69460
C	-2.69152	-2.43117	-0.88243
C	-4.05643	-2.56941	-1.13160
C	-4.68409	-1.85588	-2.14400
C	-3.94791	-0.98361	-2.93737
C	-2.58688	-0.83266	-2.71386
C	-2.02628	-3.17904	0.25298
C	-2.00512	-2.37044	1.53252
C	-0.97844	-1.46999	1.80830

C	-0.97281	-0.68614	2.95277
C	-2.01711	-0.79635	3.85965
C	-3.05174	-1.69199	3.61448
C	-3.03925	-2.46753	2.46263
O	0.09249	-1.35103	0.93097
H	4.87607	-1.14667	-2.04267
H	6.78819	-1.78514	-0.63896
H	6.42254	-2.95280	1.51593
H	4.11122	-3.48579	2.24664
H	2.18881	-2.80646	0.85148
H	2.87516	-2.19245	-3.41615
H	3.05261	-0.44657	-3.19632
H	1.44945	-1.13636	-3.47519
H	1.37355	2.27605	-2.00826
H	-4.63494	-3.24927	-0.51443
H	-5.74746	-1.97942	-2.31206
H	-4.42999	-0.41878	-3.72628
H	-1.98625	-0.15873	-3.31286
H	-2.57515	-4.10569	0.43155
H	-1.00766	-3.45071	-0.03137
H	-0.15566	0.00787	3.10731
H	-2.02359	-0.18109	4.75151
H	-3.87095	-1.78389	4.31792
H	-3.85087	-3.16254	2.27312
H	5.10624	1.75613	1.25749
H	2.07185	1.03956	2.74166
H	0.65304	2.97094	2.14418
H	0.58907	3.79535	-0.18196
H	2.70955	4.25043	-1.28161
H	4.75677	3.89178	0.07112
Au	-1.38158	1.40579	-0.06947
Cl	-2.82286	3.21523	0.21195

(*S*,*S*)-**L**$_{\mathbf{BB1}}$AuCl

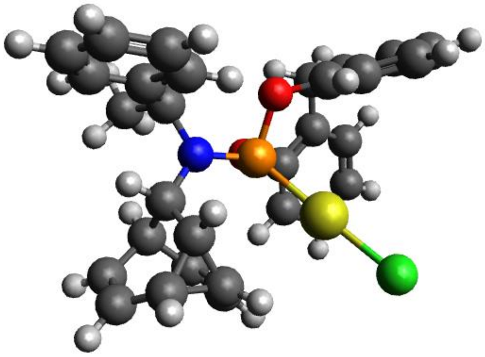

Table S27. Coordinates and energy for the optimised geometry of (*S*,*S*)-**L**$_{\mathbf{BB1}}$AuCl.

G = -2302.158857			
	**Coordinates / Å**		
**Atom**	**x**	**y**	**z**
H	1.50247	2.23239	1.49088
C	2.26193	1.45396	1.42564
C	3.36849	2.00535	0.53253
C	4.67323	2.19625	0.97728
C	5.64783	2.70714	0.12207
C	5.32742	3.03420	-1.18745
C	4.02298	2.85092	-1.64048
C	3.05609	2.34172	-0.78734
C	2.68620	1.12068	2.85264
N	1.61769	0.30052	0.73941
C	2.49821	-0.86548	0.52993
C	2.42908	-1.47900	-0.84810
H	2.46008	-0.78405	-1.67889
C	1.63334	-2.74956	-1.01740
C	0.85236	-3.23460	0.12957
C	1.15570	-2.84714	1.37295
H	0.60183	-3.20893	2.23186
C	2.37325	-1.98379	1.59756
C	3.55256	-2.91865	1.42853
C	3.90831	-3.28793	0.19416
C	3.19687	-2.76607	-0.98491
H	3.50595	-0.45294	0.63031
P	-0.00305	0.31455	0.47500
O	-0.64059	0.08351	1.97083
C	-1.99151	-0.20716	2.12898
C	-2.92502	0.82651	2.16619
C	-4.26128	0.47778	2.35842
C	-4.65079	-0.84766	2.49812
C	-3.69861	-1.85916	2.44751
C	-2.36146	-1.53724	2.26426
C	-2.51918	2.26911	1.95465

C	-2.51437	2.64187	0.48796
C	-1.40024	2.41807	-0.31694
C	-1.39704	2.71021	-1.67254
C	-2.53800	3.24080	-2.25733
C	-3.66439	3.47939	-1.47854
C	-3.64548	3.18232	-0.12216
O	-0.23172	1.91931	0.24565
H	4.94844	1.94599	1.99488
H	6.66015	2.84482	0.48545
H	6.08609	3.42966	-1.85334
H	3.76101	3.10692	-2.66114
H	2.04188	2.19702	-1.14284
H	3.02801	2.02586	3.35975
H	3.49586	0.38781	2.88955
H	1.83088	0.72363	3.40175
H	1.21380	-2.93439	-1.99826
H	0.03019	-3.91469	-0.06408
H	2.35870	-1.54379	2.59270
H	4.03165	-3.33464	2.30795
H	4.70064	-4.01124	0.03406
H	3.64904	-2.95948	-1.94933
H	-5.00822	1.26442	2.39250
H	-5.69713	-1.09060	2.64125
H	-3.99426	-2.89693	2.54561
H	-1.59621	-2.30131	2.21061
H	-3.22541	2.91487	2.47975
H	-1.53120	2.44358	2.38516
H	-0.50494	2.51051	-2.25394
H	-2.54575	3.46356	-3.31755
H	-4.55896	3.89448	-1.92764
H	-4.52752	3.36858	0.48207
Au	-1.03521	-0.89706	-1.14434
Cl	-2.09963	-2.09030	-2.83962

TS-$\mathbf{L_{BB1}}$AuCl

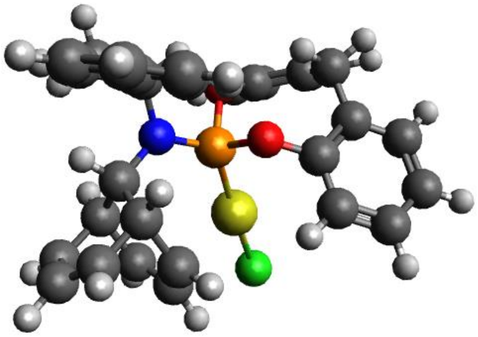

Table S28. Coordinates, energy, and imaginary vibrational frequency for the optimised geometry of TS-$\mathbf{L_{BB1}}$AuCl.

	G = -2302.141509 *v* = -399.18		
		**Coordinates / Å**	
**Atom**	**x**	**y**	**z**
H	1.44621	-2.18158	-1.65270
C	2.19130	-1.38937	-1.63294
C	3.35417	-1.91040	-0.80194
C	4.68123	-1.60509	-1.09127
C	5.70899	-2.05361	-0.26648
C	5.42027	-2.82189	0.85374
C	4.09756	-3.14705	1.14226
C	3.07631	-2.69505	0.31889
C	2.52417	-1.05663	-3.08355
N	1.53707	-0.21138	-0.98209
C	2.45752	0.88678	-0.62349
C	2.94766	0.85941	0.81989
H	3.24352	-0.13095	1.15100
C	2.13779	1.62953	1.80448
C	1.25642	2.63253	1.40093
C	1.17662	3.00208	0.07545
C	1.98587	2.29868	-0.96667
C	3.05915	3.30332	-0.68618
C	4.00359	3.07159	0.29192
C	3.94111	1.93192	1.09833
H	3.33547	0.71647	-1.24848
P	0.00078	-0.34756	-0.40820
O	-0.57612	-1.47277	-1.44328
C	-1.94140	-1.70572	-1.57034
C	-2.58589	-2.55435	-0.67345
C	-3.94729	-2.77951	-0.87442
C	-4.63464	-2.17865	-1.92056
C	-3.96404	-1.33399	-2.79747
C	-2.60783	-1.09844	-2.62371
C	-1.85804	-3.17735	0.49870

C	-1.85482	-2.27297	1.71260
C	-0.87129	-1.30298	1.89329
C	-0.88413	-0.43171	2.97231
C	-1.90295	-0.52378	3.90986
C	-2.89243	-1.48889	3.76120
C	-2.86262	-2.35093	2.67277
O	0.17433	-1.20412	0.98347
H	4.92784	-1.00824	-1.96228
H	6.73641	-1.80032	-0.50269
H	6.22050	-3.17096	1.49659
H	3.86269	-3.75189	2.01101
H	2.04436	-2.92663	0.56141
H	3.00495	-1.91106	-3.56556
H	3.19195	-0.19668	-3.17710
H	1.60156	-0.82514	-3.61857
H	1.56850	2.35802	-1.96975
H	-4.47517	-3.43756	-0.19179
H	-5.69381	-2.36790	-2.04945
H	-4.49345	-0.85595	-3.61292
H	-2.05738	-0.44279	-3.28751
H	-2.35253	-4.11499	0.75943
H	-0.83169	-3.41640	0.21242
H	-0.10150	0.31306	3.05211
H	-1.92451	0.15904	4.75089
H	-3.69055	-1.56753	4.49002
H	-3.63960	-3.09986	2.55804
H	4.66015	1.77235	1.89131
H	2.21400	1.34987	2.84791
H	0.71220	3.19960	2.14659
H	0.54331	3.82177	-0.23882
H	3.05799	4.22416	-1.25625
H	4.71919	3.84939	0.53385
Au	-1.42265	1.40963	-0.20211
Cl	-2.95710	3.16248	-0.06867

(*C*,*R*,*S*)-**L**$_{\textbf{BB1}}$PdCl$_2$

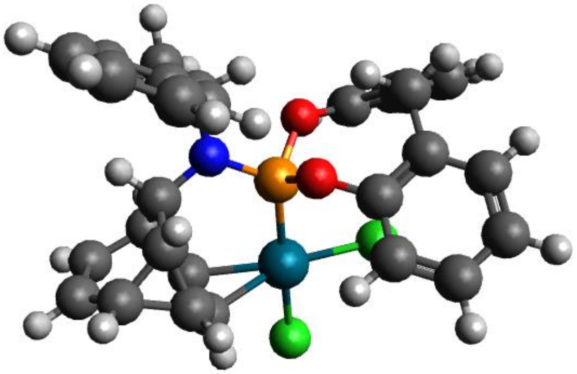

Table S29. Coordinates and energy for the optimised geometry of (C,*R*,*S*)-**L**$_{\textbf{BB1}}$PdCl$_2$.

G = -2754.516228			
	Coordinates / Å		
**Atom**	**x**	**y**	**z**
Pd	-0.68592	1.99138	0.03022
Cl	-1.43280	4.23049	0.25762
Cl	-2.88791	1.24386	0.21694
P	-0.09020	-0.15859	-0.34309
C	1.29166	2.78469	0.76768
C	2.16157	1.88451	1.53052
O	-0.87147	-0.96270	-1.50902
O	-0.19476	-1.13134	0.96103
N	1.44276	-0.42614	-0.86211
C	1.30482	2.77028	-0.61660
H	0.87868	3.64099	1.28680
H	1.95926	1.79640	2.58977
C	2.82498	0.72752	0.84698
C	-2.21816	-1.21633	-1.73572
C	-1.30014	-1.28100	1.79062
C	1.83903	-1.75129	-1.41344
C	2.52598	0.53123	-0.62272
C	2.32314	1.89257	-1.31238
C	3.59387	2.70137	-1.14921
H	0.93468	3.63245	-1.16078
H	2.96884	-0.18015	1.41986
C	3.69510	1.90447	1.16805
C	-2.82083	-0.52071	-2.77093
C	-2.88770	-2.19461	-1.00687
C	-2.29264	-2.18825	1.43743
C	-1.34058	-0.55224	2.96717
H	0.96229	-2.39307	-1.32560
C	2.15934	-1.61973	-2.89804
C	2.93285	-2.38255	-0.56208
H	3.41185	0.08771	-1.08065
H	2.06863	1.73825	-2.36064

H	3.94137	3.31669	-1.97075
C	4.19674	2.71609	0.04443
H	4.34014	1.82282	2.03319
H	-2.24956	0.23045	-3.30222
C	-4.14684	-0.77790	-3.08258
C	-4.22006	-2.43345	-1.34083
C	-2.23330	-2.94752	0.13060
C	-3.36460	-2.33375	2.31508
C	-2.41952	-0.71336	3.82411
H	-0.54006	0.14430	3.18603
H	2.97107	-0.91076	-3.08324
H	1.27167	-1.26398	-3.42268
H	2.45057	-2.58626	-3.31557
C	4.25187	-2.48987	-0.99382
C	2.60414	-2.85399	0.71125
H	5.06998	3.33524	0.21679
H	-4.62909	-0.22605	-3.88060
C	-4.85096	-1.73535	-2.36157
H	-4.76978	-3.18598	-0.78459
H	-1.19358	-3.17228	-0.11919
H	-2.74741	-3.90245	0.25580
H	-4.15961	-3.02792	2.06228
C	-3.43467	-1.60470	3.49530
H	-2.47073	-0.13772	4.74060
H	4.53454	-2.14193	-1.98090
C	5.22642	-3.04681	-0.16911
C	3.57166	-3.41159	1.53443
H	1.58116	-2.76297	1.06070
H	-5.88900	-1.94082	-2.59529
H	-4.28266	-1.73162	4.15803
H	6.24916	-3.12089	-0.52144
C	4.89014	-3.50721	1.09643
H	3.29768	-3.77413	2.51897
H	5.64794	-3.94284	1.73783

(*A*,*S*,*S*)-$\mathbf{L_{BB1}}$PdCl$_2$

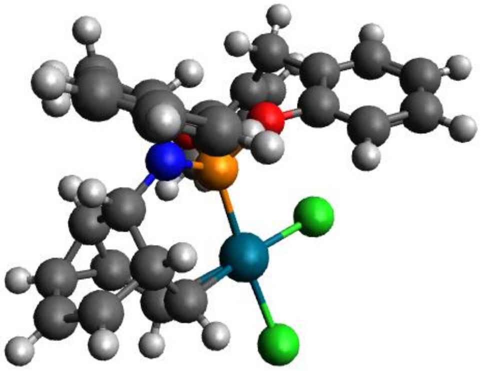

Table S30. Coordinates and energy for the optimised geometry of (A,*S*,*S*)-$\mathbf{L_{BB1}}$PdCl$_2$.

	G = -2754.516897		
		**Coordinates / Å**	
**Atom**	**x**	**y**	**z**
Pd	-0.49329	-1.67555	-0.93729
Cl	-0.95500	-3.48065	-2.40496
Cl	-2.67206	-0.88721	-1.21222
P	-0.09705	0.17823	0.29598
C	1.12366	-3.01184	-0.12823
C	1.68387	-2.17596	-1.07888
O	-0.83649	0.23091	1.74679
O	-0.44581	1.59996	-0.39293
N	1.46566	0.48483	0.68952
C	1.48019	-2.87512	1.28727
H	1.68033	-2.47835	-2.12050
C	2.76334	-1.21189	-0.63527
C	-2.19308	0.03597	1.98297
C	-1.60734	2.12515	-0.94357
C	1.86104	1.78911	1.27897
C	2.47981	-0.57145	0.73544
C	2.18640	-1.64176	1.76344
C	3.01402	-2.88629	1.63910
H	0.85846	-3.40519	1.99670
H	2.93397	-0.42765	-1.37205
C	3.96152	-2.12770	-0.49262
C	-2.61367	-1.22023	2.38584
C	-3.06476	1.11195	1.85659
C	-2.62722	2.59860	-0.12400
C	-1.65132	2.23691	-2.32335
H	1.01265	2.45754	1.12922
C	2.10040	1.67779	2.78154
C	3.01732	2.36318	0.47058
H	3.40523	-0.06750	1.02285
H	1.94964	-1.28939	2.75983
H	3.24088	-3.42540	2.54969

C	4.03315	-2.93092	0.57395
H	4.69268	-2.17342	-1.29099
H	-1.88902	-2.02243	2.46005
C	-3.95795	-1.42706	2.65851
C	-4.40881	0.87996	2.13997
C	-2.58120	2.46431	1.38217
C	-3.72953	3.17503	-0.75338
C	-2.75921	2.81457	-2.92372
H	-0.82786	1.84770	-2.90940
H	2.94109	1.02172	3.02261
H	1.20414	1.27979	3.25970
H	2.31027	2.66265	3.20473
C	4.25157	2.67580	1.03122
C	2.82698	2.57605	-0.89762
H	4.84269	-3.64469	0.67763
H	-4.30210	-2.40934	2.95959
C	-4.85731	-0.37420	2.53361
H	-5.11461	1.69867	2.04324
H	-1.56454	2.63955	1.74182
H	-3.21563	3.23774	1.81947
H	-4.54416	3.54750	-0.14063
C	-3.80450	3.28215	-2.13566
H	-2.80772	2.89158	-4.00336
H	4.42719	2.52499	2.08997
C	5.28014	3.18649	0.24128
C	3.84757	3.08597	-1.68526
H	1.86567	2.33475	-1.33928
H	-5.90938	-0.53000	2.74163
H	-4.67612	3.73243	-2.59597
H	6.23696	3.42227	0.69361
C	5.08244	3.39114	-1.11656
H	3.68184	3.24881	-2.74443
H	5.88249	3.78839	-1.73095
H	0.66591	-3.94239	-0.44044

TS-$\mathbf{L_{BB1}}$PdCl$_2$ (TS-Pd)

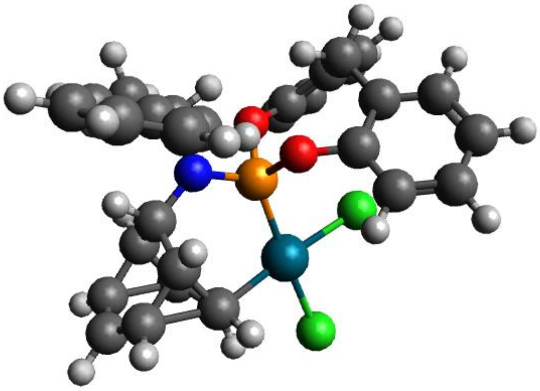

Table S31. Coordinates, energy, and imaginary vibrational frequency for the optimised geometry of TS-$\mathbf{L_{BB1}}$PdCl$_2$ (TS-Pd).

	G = -2754.493808		
	v = -424.14		
	Coordinates / Å		
**Atom**	**x**	**y**	**z**
Pd	-0.63804	1.92827	0.51987
Cl	-1.37160	3.99315	1.42007
Cl	-2.81392	1.09781	0.72279
P	-0.07655	-0.11705	-0.28165
C	1.32512	2.91916	0.36770
C	2.07516	2.09176	1.24351
O	-0.82171	-0.58566	-1.65257
O	-0.32080	-1.36381	0.73830
N	1.48086	-0.44921	-0.71156
C	1.52416	2.80133	-1.03189
H	1.01934	3.88700	0.74325
H	1.99213	2.23540	2.31290
C	2.90202	0.98166	0.72781
C	-2.17444	-0.63835	-1.95787
C	-1.45936	-1.75245	1.43012
C	1.81545	-1.78945	-1.26119
C	2.57556	0.51821	-0.68281
C	2.33987	1.70800	-1.60024
C	3.36897	2.78728	-1.47283
H	1.02322	3.48788	-1.70182
H	3.07981	0.15743	1.41212
C	3.91138	2.08455	0.78806
C	-2.69811	0.38195	-2.73452
C	-2.93492	-1.73103	-1.55330
C	-2.43827	-2.50497	0.78891
C	-1.53097	-1.43539	2.77643
H	0.93455	-2.41177	-1.09832
C	2.05470	-1.71636	-2.76555

C	2.94470	-2.40876	-0.44712
H	3.45535	-0.01236	-1.05159
H	2.10017	1.42055	-2.61994
H	3.55227	3.43375	-2.32032
C	4.14703	2.88424	-0.32494
H	4.51496	2.18801	1.67948
H	-2.05978	1.21054	-3.01633
C	-4.03280	0.33508	-3.10805
C	-4.27261	-1.75551	-1.94295
C	-2.35700	-2.82773	-0.68660
C	-3.52788	-2.91593	1.55403
C	-2.62604	-1.85596	3.51606
H	-0.74115	-0.84209	3.22097
H	2.90544	-1.07761	-3.01951
H	1.16378	-1.31225	-3.24722
H	2.24890	-2.71205	-3.17069
C	4.18339	-2.72595	-0.99659
C	2.72967	-2.65577	0.91178
H	4.88938	3.67143	-0.25876
H	-4.45451	1.13719	-3.70207
C	-4.82352	-0.73620	-2.70854
H	-4.89194	-2.59183	-1.63494
H	-1.31845	-3.01478	-0.96880
H	-2.91136	-3.74958	-0.87362
H	-4.31133	-3.49765	1.07885
C	-3.62959	-2.59612	2.90171
H	-2.69824	-1.59806	4.56595
H	4.37727	-2.55170	-2.04860
C	5.19157	-3.27384	-0.20570
C	3.73064	-3.20296	1.70045
H	1.76594	-2.40932	1.34458
H	-5.86840	-0.77866	-2.99293
H	-4.49087	-2.92521	3.47124
H	6.15104	-3.51385	-0.65012
C	4.96946	-3.51195	1.14306
H	3.54506	-3.39286	2.75183
H	5.75316	-3.93958	1.75817

(C,S,S)-**L**$_{\mathbf{BB1}}$RuCp(NCMe)$^{+}$

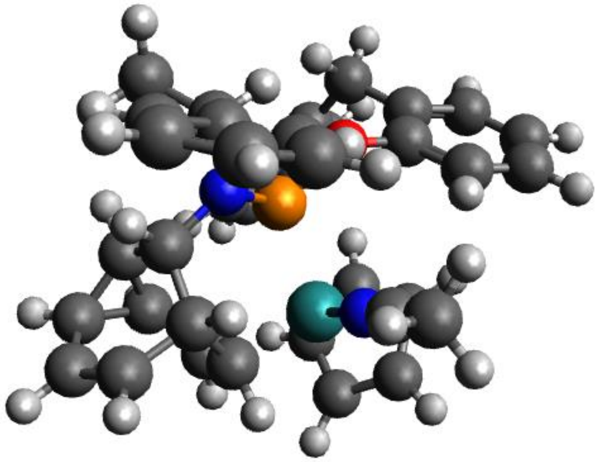

Table S32. Coordinates and energy for the optimised geometry of (C,S,S)-**L**$_{\mathbf{BB1}}$RuCp(NCMe)$^{+}$.

	G = -2127.043877		
		**Coordinates / Å**	
**Atom**	**x**	**y**	**z**
Ru	0.65890	-0.78705	1.44405
P	0.11287	0.09303	-0.57315
N	-0.72071	0.55293	2.19845
C	-0.30834	-2.81436	0.91510
C	-0.50887	-3.25104	-0.48142
C	2.88083	-0.61000	1.24520
C	2.43256	0.21075	2.31155
C	1.87675	-0.65178	3.32116
C	1.94358	-1.97277	2.85573
C	2.53767	-1.95512	1.54203
O	0.91061	-0.39752	-1.93406
O	0.24873	1.72857	-0.68755
N	-1.46620	-0.04321	-1.02293
C	-1.50523	1.26721	2.63190
C	-1.24381	-1.99351	1.53273
H	0.26988	-3.49567	1.52644
H	0.32623	-3.75571	-0.94927
C	-1.44770	-2.50362	-1.37342
H	3.38759	-0.26705	0.35708
H	2.55388	1.28054	2.37662
H	1.45625	-0.33380	4.26344
H	1.59301	-2.84640	3.38476
H	2.76747	-2.81378	0.93165
C	2.27027	-0.62905	-2.00075
C	1.32820	2.45958	-0.22476
C	-2.09367	0.84808	-2.02849
C	-2.16093	-1.31480	-0.76360
C	-2.50847	2.16575	3.16754

C	-2.46563	-1.58524	0.72455
C	-3.34777	-2.81374	0.80734
H	-1.40102	-2.08060	2.60239
H	-1.18351	-2.40946	-2.41936
C	-1.89840	-3.84959	-0.87695
C	2.72612	-1.93582	-1.90285
C	3.14488	0.43768	-2.20154
C	2.49527	2.54904	-0.98230
C	1.17873	3.16019	0.96438
H	-1.42309	1.70060	-2.13054
C	-2.20954	0.19165	-3.40164
C	-3.39341	1.38449	-1.44731
H	-3.13524	-1.21610	-1.24571
H	-3.49156	1.85434	2.81082
H	-2.31063	3.18392	2.82839
H	-2.48771	2.13645	4.25770
H	-2.95634	-0.70805	1.14735
H	-4.16890	-2.83505	1.51471
C	-3.02904	-3.88230	0.07168
H	-1.83261	-4.67551	-1.57379
H	2.00587	-2.73179	-1.76855
C	4.08785	-2.19518	-1.96419
C	4.50897	0.15404	-2.26180
C	2.63133	1.85375	-2.31896
C	3.53757	3.32358	-0.47174
C	2.22474	3.93329	1.44684
H	0.24105	3.08900	1.49930
H	-2.82713	-0.70994	-3.39125
H	-1.21475	-0.08395	-3.75577
H	-2.65222	0.89143	-4.11402
C	-3.31485	2.31353	-0.40636
C	-4.64900	0.97733	-1.88608
H	-3.59903	-4.80159	0.14951
H	4.44771	-3.21409	-1.88172
C	4.98370	-1.14531	-2.13490
H	5.20920	0.96853	-2.41616
H	1.66427	1.84906	-2.82585
H	3.32170	2.42811	-2.93912
H	4.45542	3.40672	-1.04466
C	3.41584	4.00341	0.73257
H	2.10897	4.48127	2.37466
H	-2.33444	2.63894	-0.06950
C	-4.46418	2.82564	0.17713
C	-5.80589	1.47949	-1.29315
H	-4.74022	0.26800	-2.70082
H	6.04861	-1.33889	-2.18401

H	4.23952	4.60311	1.10107
H	-4.39157	3.56640	0.96630
C	-5.71744	2.40380	-0.26223
H	-6.77647	1.15088	-1.64712
H	-6.61739	2.80411	0.19050

(*C*,*R*,*S*)-$\mathbf{L_{BB1}}$RuCp(NCMe)

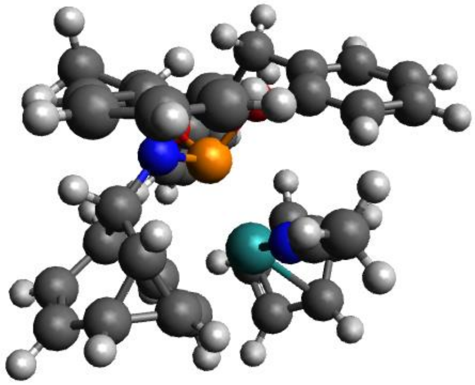

Table S33. Coordinates and energy for the optimised geometry of (C,*R*,*S*)-$\mathbf{L_{BB1}}$RuCp(NCMe).

G = -2127.049351			
	Coordinates / Å		
**Atom**	**x**	**y**	**z**
Ru	0.66029	-1.05844	1.24624
P	0.17318	0.18320	-0.58738
N	-0.73651	0.13616	2.16508
C	-0.99824	-2.56178	1.26239
C	-0.38847	-2.62847	0.00334
C	2.88358	-0.75761	1.18088
C	2.37524	-0.28058	2.41510
C	1.81269	-1.40040	3.12034
C	1.95909	-2.54149	2.30435
C	2.59572	-2.14771	1.08577
O	0.99687	-0.10249	-1.98973
O	0.35338	1.80934	-0.43048
N	-1.38841	0.15036	-1.10085
C	-1.52318	0.80404	2.66266
C	-2.38700	-2.05549	1.36591
H	-0.70234	-3.26586	2.03139
H	0.38924	-3.36582	-0.16071
C	-1.25154	-2.35038	-1.22214
H	3.39748	-0.16676	0.43836
H	2.44192	0.73467	2.77345
H	1.35926	-1.37240	4.09944
H	1.62746	-3.54067	2.54461
H	2.87695	-2.79744	0.27309
C	2.34872	-0.36522	-2.07961

C	1.44254	2.40466	0.18230
C	-1.95280	1.20853	-1.97514
C	-2.14443	-1.11194	-1.02544
C	-2.54068	1.63305	3.27691
C	-2.97097	-1.27017	0.23495
C	-3.49492	-2.66916	0.47019
H	-2.72570	-1.80779	2.36431
H	-0.63834	-2.21274	-2.11332
C	-2.13245	-3.57234	-1.35352
C	2.75457	-1.68411	-2.22134
C	3.26085	0.68877	-2.08219
C	2.63861	2.57246	-0.51428
C	1.27643	2.88954	1.47209
H	-1.27349	2.05572	-1.89070
C	-1.98537	0.80068	-3.44600
C	-3.27926	1.65710	-1.38422
H	-2.83948	-1.09602	-1.86623
H	-3.47939	1.50195	2.73574
H	-2.24485	2.68230	3.22592
H	-2.67352	1.34482	4.32045
H	-3.63421	-0.44468	0.46480
H	-4.46007	-2.75313	0.95379
C	-3.17237	-3.70738	-0.52782
H	-1.87145	-4.33937	-2.07357
H	2.00370	-2.46538	-2.23520
C	4.10691	-1.97607	-2.33350
C	4.61388	0.37177	-2.19366
C	2.79639	2.12091	-1.95019
C	3.69142	3.19468	0.15764
C	2.33388	3.51658	2.11484
H	0.31656	2.76647	1.95627
H	-2.60707	-0.07649	-3.63867
H	-0.97146	0.57353	-3.77893
H	-2.37697	1.62305	-4.04905
C	-3.25201	2.46479	-0.24494
C	-4.51025	1.25383	-1.89034
H	-3.80267	-4.58921	-0.56632
H	4.43023	-3.00477	-2.44183
C	5.04021	-0.94558	-2.30953
H	5.34304	1.17544	-2.19773
H	1.84638	2.24428	-2.47447
H	3.52549	2.77020	-2.43814
H	4.63304	3.33446	-0.36307
C	3.55248	3.65551	1.45982
H	2.20564	3.89584	3.12195
H	-2.29086	2.78463	0.14868

C	-4.42900	2.85582	0.37671
C	-5.69404	1.63466	-1.26233
H	-4.56003	0.63653	-2.78015
H	6.09748	-1.16541	-2.39731
H	4.38565	4.14108	1.95365
H	-4.39530	3.50154	1.24793
C	-5.65723	2.43388	-0.12794
H	-6.64564	1.30933	-1.66710
H	-6.57886	2.73878	0.35452

TS-(*C*,*S*,*S*)-to-(*C*,*R*,*S*)-$\mathbf{L_{BB1}}$RuCp(NCMe)$^+$ (TS1-Ru)

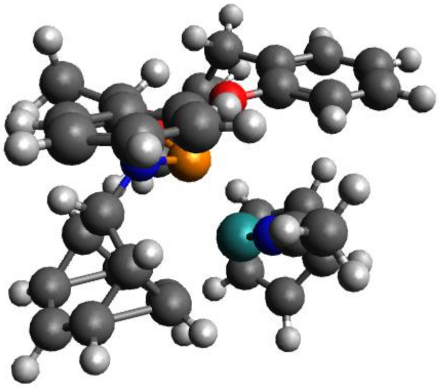

Table S34. Coordinates, energy, and imaginary vibrational frequency for the optimised geometry of TS-(*C*,*S*,*S*)-to-(*C*,*R*,*S*)-$\mathbf{L_{BB1}}$RuCp(NCMe)$^+$ (TS1-Ru).

G = -2127.016824

v = -488.30

	Coordinates / Å		
**Atom**	**x**	**y**	**z**
Ru	0.75414	-0.70878	1.48765
P	0.12193	0.07115	-0.60237
N	-0.68943	0.57296	2.18462
C	-0.72710	-2.56545	1.34942
C	-0.48898	-2.99106	0.01687
C	2.94654	-1.03365	1.18352
C	2.73408	0.18872	1.91789
C	2.10870	-0.16135	3.15702
C	1.88714	-1.56865	3.18019
C	2.41442	-2.10722	1.94219
O	0.93016	-0.46775	-2.07971
O	0.27101	1.79946	-0.83311
N	-1.49082	-0.08020	-1.10759
C	-1.47171	1.31896	2.59483
C	-1.93206	-1.88960	1.63965
H	-0.26815	-3.13650	2.14574
H	0.38775	-3.57714	-0.21120
C	-1.37279	-2.56867	-1.10991

H	3.45037	-1.11773	0.23959
H	3.07070	1.16778	1.62949
H	1.82106	0.53289	3.92765
H	1.46594	-2.13413	3.99213
H	2.43674	-3.14640	1.66463
C	2.29686	-0.74913	-2.12242
C	1.37551	2.49177	-0.31505
C	-2.09404	0.85940	-2.09105
C	-2.22370	-1.32994	-0.84302
C	-2.47614	2.23425	3.10183
C	-2.83169	-1.40897	0.55569
C	-3.48183	-2.73734	0.84801
H	-2.18022	-1.65191	2.66195
H	-0.90390	-2.54352	-2.08576
C	-2.03523	-3.86500	-0.74537
C	2.69447	-2.08235	-2.10786
C	3.22331	0.29516	-2.19657
C	2.60156	2.45967	-0.98502
C	1.19759	3.21886	0.85610
H	-1.41650	1.70887	-2.13762
C	-2.16594	0.24796	-3.49112
C	-3.40806	1.36968	-1.51717
H	-3.05706	-1.34644	-1.54457
H	-3.41887	2.06770	2.58172
H	-2.17028	3.26749	2.94054
H	-2.62811	2.07827	4.16895
H	-3.43673	-0.54048	0.78818
H	-4.29908	-2.76631	1.55223
C	-3.10021	-3.87907	0.15269
H	-1.74189	-4.75921	-1.27284
H	1.93628	-2.85143	-2.08200
C	4.04960	-2.39735	-2.12608
C	4.58050	-0.04321	-2.20724
C	2.76578	1.73614	-2.30677
C	3.66862	3.15778	-0.40840
C	2.27045	3.91031	1.40854
H	0.22093	3.23149	1.31544
H	-2.77186	-0.65878	-3.53296
H	-1.15738	-0.00453	-3.81584
H	-2.59307	0.96666	-4.19173
C	-3.35567	2.32219	-0.49100
C	-4.65381	0.90872	-1.94338
H	-3.57713	-4.82109	0.38689
H	4.36505	-3.43092	-2.11394
C	4.99601	-1.37253	-2.15982
H	5.31871	0.74609	-2.26668

H	1.81762	1.76251	-2.84381
H	3.49426	2.28259	-2.90666
H	4.62790	3.15336	-0.90940
C	3.51518	3.86770	0.77997
H	2.13761	4.47899	2.31791
H	-2.38698	2.68569	-0.16742
C	-4.52336	2.80554	0.09097
C	-5.82718	1.38089	-1.35081
H	-4.72243	0.18721	-2.74633
H	6.05071	-1.60752	-2.16862
H	4.35581	4.39931	1.20244
H	-4.47304	3.56440	0.86106
C	-5.76575	2.32866	-0.33393
H	-6.78517	1.01368	-1.69242
H	-6.67477	2.70429	0.11503

(*A*,*S*,*S*)-**L$_{BB1}$**RuCp(NCMe)$^+$

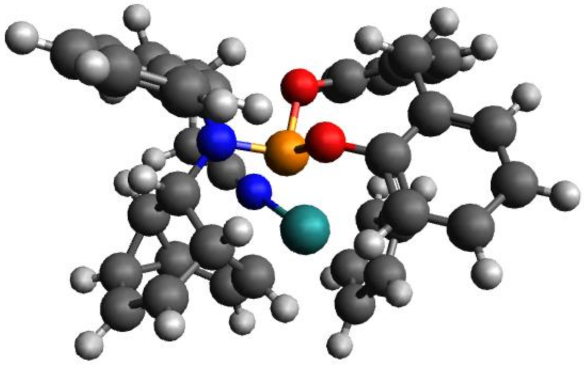

Table S35. Coordinates and energy for the optimised geometry of (*A*,*S*,*S*)-**L$_{BB1}$**RuCp(NCMe)$^+$.

G = -2127.048275			
	**Coordinates / Å**		
**Atom**	**x**	**y**	**z**
Ru	-0.99802	-1.52044	-0.63296
P	0.01242	0.27265	0.31598
N	-0.52282	-2.45894	1.13434
C	0.73790	-2.68551	-1.42889
C	1.74416	-3.16179	-0.45164
C	-3.18752	-1.58146	-0.29886
C	-2.91691	-0.48448	-1.15514
C	-2.31604	-0.98352	-2.34456
C	-2.27524	-2.40930	-2.24292
C	-2.79855	-2.78228	-0.98765
O	-0.58361	0.76313	1.76654
O	0.03651	1.69552	-0.52149
N	1.59422	0.10001	0.73630
C	-0.24615	-2.96880	2.12230
C	0.84869	-1.38743	-1.94210

H	0.27717	-3.45724	-2.03418
H	1.48918	-4.06815	0.08361
C	2.59929	-2.16500	0.26500
H	-3.64626	-1.52053	0.67534
H	-3.12664	0.55185	-0.93871
H	-2.01672	-0.39679	-3.19743
H	-1.88685	-3.08173	-2.99338
H	-2.89155	-3.78987	-0.61184
C	-1.93741	0.88315	2.03554
C	-1.00113	2.22709	-1.25892
C	2.24197	0.97406	1.74862
C	2.48239	-0.70376	-0.12184
C	0.13777	-3.61472	3.36189
C	2.10758	-0.59301	-1.61077
C	3.19538	-1.23812	-2.43873
H	0.43513	-1.17713	-2.92240
H	2.85047	-2.36182	1.30035
C	3.25367	-3.04018	-0.78186
C	-2.54292	-0.12125	2.77820
C	-2.64123	2.01725	1.63189
C	-1.99665	2.96188	-0.61778
C	-0.98263	2.06358	-2.63604
H	1.47722	1.68032	2.07378
C	2.64148	0.14785	2.96689
C	3.36951	1.79696	1.13285
H	3.47235	-0.25473	-0.01905
H	1.14350	-4.02554	3.25681
H	-0.55883	-4.42212	3.59061
H	0.12984	-2.88807	4.17555
H	1.99209	0.46010	-1.86901
H	3.50272	-0.77121	-3.36727
C	3.70542	-2.40303	-2.03463
H	3.85415	-3.86617	-0.42170
H	-1.94847	-0.96736	3.09619
C	-3.88944	-0.02866	3.09798
C	-3.99727	2.08207	1.95527
C	-1.97515	3.14978	0.88150
C	-3.01385	3.49655	-1.40684
C	-2.00520	2.60611	-3.40226
H	-0.16886	1.50896	-3.08825
H	3.40884	-0.59197	2.72372
H	1.76169	-0.37683	3.34348
H	3.02798	0.78977	3.76165
C	3.07349	2.67027	0.08246
C	4.68667	1.71317	1.57686
H	4.46124	-2.91400	-2.62097

H	-4.36183	-0.81262	3.67841
C	-4.62412	1.07225	2.67203
H	-4.56539	2.95465	1.64993
H	-0.94198	3.25335	1.21967
H	-2.49263	4.07896	1.12609
H	-3.80168	4.07181	-0.93145
C	-3.02939	3.31528	-2.78450
H	-1.99960	2.47830	-4.47844
H	2.05261	2.74046	-0.27564
C	4.06926	3.43229	-0.51083
C	5.68812	2.47874	0.98377
H	4.95079	1.04911	2.39109
H	-5.67690	1.15422	2.91439
H	-3.83021	3.74228	-3.37630
H	3.81977	4.10565	-1.32342
C	5.38408	3.33806	-0.06218
H	6.70768	2.39894	1.34390
H	6.16323	3.93348	-0.52432

(*A*,*R*,*S*)-$\mathbf{L_{BB1}}$RuCp(NCMe)$^+$

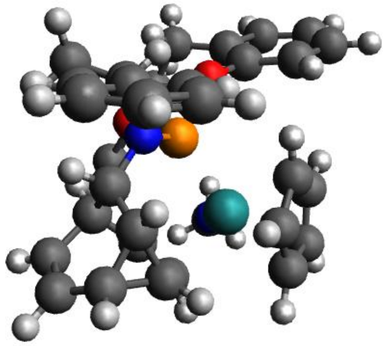

Table S36. Coordinates and energy for the optimised geometry of (*A*,*R*,*S*)-$\mathbf{L_{BB1}}$RuCp(NCMe)$^+$.

G = -2127.025013			
	Coordinates / Å		
**Atom**	**x**	**y**	**z**
Ru	0.36059	-1.00806	1.40241
P	0.08863	0.41387	-0.33689
N	2.18756	-1.79705	0.81871
C	-1.08038	-2.72742	0.84329
C	-0.44364	-2.41971	-0.34316
C	0.98679	-1.16982	3.55464
C	1.08438	0.18555	3.09612
C	-0.24107	0.60715	2.76848
C	-1.12497	-0.47631	2.95767
C	-0.35341	-1.58055	3.46786
O	0.82761	0.15768	-1.79905
O	0.38983	2.01522	-0.10789

N	-1.50441	0.50650	-0.78774
C	3.25758	-2.17359	0.65422
C	-2.49542	-2.33321	1.02149
H	-0.69940	-3.53497	1.45678
H	0.46191	-2.95464	-0.60273
C	-1.28449	-1.89766	-1.50141
H	1.81523	-1.78666	3.86947
H	1.97393	0.79448	3.07434
H	-0.53986	1.57825	2.41356
H	-2.19141	-0.43906	2.81344
H	-0.73679	-2.55173	3.74082
C	2.12484	-0.21266	-2.07252
C	1.57521	2.55032	0.38326
C	-2.08211	1.69675	-1.46420
C	-2.22072	-0.76991	-1.03170
C	4.61847	-2.62726	0.44511
C	-3.06914	-1.27841	0.12110
C	-3.55880	-2.69984	-0.04733
H	-2.87839	-2.38665	2.03234
H	-0.65806	-1.52930	-2.30921
C	-2.10165	-3.07896	-1.96716
C	2.39529	-1.52768	-2.42784
C	3.11016	0.76926	-2.11546
C	2.72517	2.61746	-0.40581
C	1.54808	3.12711	1.64781
H	-1.51471	2.54980	-1.09381
C	-1.91235	1.65125	-2.98121
C	-3.51082	1.87412	-0.97242
H	-2.90396	-0.57189	-1.85912
H	4.62540	-3.44245	-0.27902
H	5.03899	-2.97566	1.38940
H	5.21700	-1.80208	0.05731
H	-3.76169	-0.56217	0.54642
H	-4.53157	-2.93108	0.36770
C	-3.16988	-3.44489	-1.25720
H	-1.77606	-3.63267	-2.84034
H	1.59345	-2.25486	-2.43669
C	3.68691	-1.88785	-2.78638
C	4.40415	0.38457	-2.46238
C	2.74539	2.20966	-1.86058
C	3.87333	3.16697	0.16805
C	2.69681	3.68252	2.19049
H	0.61875	3.16287	2.19842
H	-2.37376	0.76839	-3.43126
H	-0.84991	1.63132	-3.22782
H	-2.36162	2.53632	-3.43765

C	-3.71128	2.28323	0.34770
C	-4.62302	1.59989	-1.76138
H	-3.76347	-4.30718	-1.54032
H	3.89901	-2.91117	-3.07470
C	4.69864	-0.93307	-2.78938
H	5.18310	1.13892	-2.50638
H	1.76621	2.40950	-2.30209
H	3.46635	2.84607	-2.37663
H	4.77596	3.21998	-0.43244
C	3.87699	3.67724	1.45740
H	2.66262	4.12623	3.17847
H	-2.84924	2.49960	0.97092
C	-4.98967	2.40485	0.87122
C	-5.90969	1.71687	-1.23964
H	-4.49903	1.29078	-2.79303
H	5.70782	-1.20791	-3.07309
H	4.78288	4.10404	1.87090
H	-5.12702	2.72856	1.89701
C	-6.09670	2.11545	0.07651
H	-6.76549	1.49696	-1.86785
H	-7.09794	2.20845	0.48157

TS-(*A*,*S*,*S*)-to-(*A*,*R*,*S*)-$\mathbf{L}_{\mathbf{BB1}}$RuCp(NCMe)$^+$ (TS5-Ru)

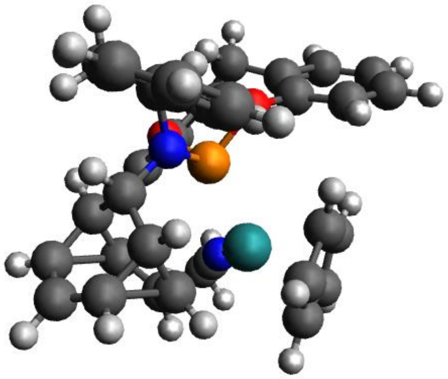

Table S37. Coordinates, energy, and imaginary vibrational frequency for the optimised geometry of TS-(*A*,*S*,*S*)-to-(*A*,*R*,*S*)-$\mathbf{L}_{\mathbf{BB1}}$RuCp(NCMe)$^+$ (TS5-Ru).

G = -2127.007073

v = -476.80

	Coordinates / Å		
**Atom**	**x**	**y**	**z**
Ru	-0.49485	1.08262	1.49954
P	0.00545	-0.33096	-0.26734
N	-2.09506	1.97516	0.58038
C	0.71836	2.86697	0.46463
C	0.56341	2.76221	-0.94426

C	-1.59599	1.18025	3.43555
C	-1.39090	-0.19020	3.06379
C	0.03559	-0.41043	3.04787
C	0.68497	0.80777	3.36927
C	-0.33364	1.81078	3.59847
O	-0.77295	-0.18316	-1.85268
O	-0.14548	-2.04789	-0.00619
N	1.61205	-0.36106	-0.82574
C	-3.06700	2.50315	0.24603
C	1.90862	2.36736	1.04359
H	0.21925	3.68915	0.96064
H	-0.30672	3.18732	-1.41975
C	1.50228	1.94765	-1.77615
H	-2.55398	1.66210	3.52847
H	-2.15220	-0.93387	2.91859
H	0.53797	-1.33810	2.85000
H	1.74555	0.93141	3.48655
H	-0.16589	2.83148	3.89352
C	-2.12299	-0.03635	-2.15855
C	-1.28294	-2.69966	0.49794
C	2.17759	-1.54093	-1.53415
C	2.32936	0.91253	-1.01562
C	-4.29759	3.15526	-0.15924
C	2.85852	1.52106	0.27944
C	3.48600	2.87224	0.09215
H	2.13403	2.58614	2.07352
H	1.08346	1.55021	-2.69141
C	2.14622	3.30095	-1.88583
C	-2.56589	1.19591	-2.63177
C	-2.97352	-1.14296	-2.10415
C	-2.41243	-2.88908	-0.30376
C	-1.20585	-3.24068	1.77761
H	1.54407	-2.38037	-1.25553
C	2.10247	-1.38003	-3.05323
C	3.56347	-1.80955	-0.96148
H	3.20312	0.67206	-1.62135
H	-4.99774	2.41629	-0.54573
H	-4.09889	3.88599	-0.94199
H	-4.75003	3.66622	0.68975
H	3.44029	0.81511	0.85932
H	4.25847	3.17672	0.78200
C	3.16243	3.66540	-0.99550
H	1.89539	3.92637	-2.72830
H	-1.85892	2.00885	-2.71451
C	-3.89534	1.35260	-3.00994
C	-4.31196	-0.95878	-2.46618

C	-2.42536	-2.51133	-1.76922
C	-3.50875	-3.54553	0.26767
C	-2.30127	-3.90337	2.31970
H	-0.28270	-3.15881	2.32946
H	2.68768	-0.53192	-3.41506
H	1.06314	-1.22321	-3.33738
H	2.47122	-2.27891	-3.54934
C	3.66607	-2.09319	0.40696
C	4.72727	-1.77354	-1.72893
H	3.62912	4.63551	-1.09878
H	-4.23748	2.30321	-3.39531
C	-4.77818	0.27710	-2.90624
H	-4.98475	-1.80616	-2.43526
H	-1.40748	-2.57941	-2.15623
H	-3.02039	-3.25573	-2.29966
H	-4.39320	-3.70128	-0.33650
C	-3.46956	-4.03297	1.57012
H	-2.23813	-4.32174	3.31413
H	2.76099	-2.11975	1.00168
C	4.90271	-2.33097	0.99469
C	5.97272	-2.00953	-1.14046
H	4.67657	-1.56547	-2.78874
H	-5.81250	0.39185	-3.19828
H	-4.32917	-4.54092	1.98354
H	4.96573	-2.55577	2.05102
C	6.06441	-2.28642	0.21938
H	6.86633	-1.97715	-1.74872
H	7.02831	-2.47076	0.67331

MeCN

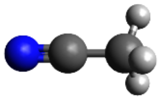

Table S38. Coordinates and energy for the optimised geometry of MeCN.

G = -132.725235			
	Coordinates / Å		
**Atom**	**x**	**y**	**z**
N	-1.42978	-0.00012	0.00030
C	-0.27973	0.00028	-0.00064
C	1.17529	-0.00007	0.00016
H	1.54537	0.24223	-0.99665
H	1.54469	-0.98470	0.28882
H	1.54502	0.74204	0.70862

(R,R,S)-**L**$_{\mathbf{BB1}}$RuCp$^+$

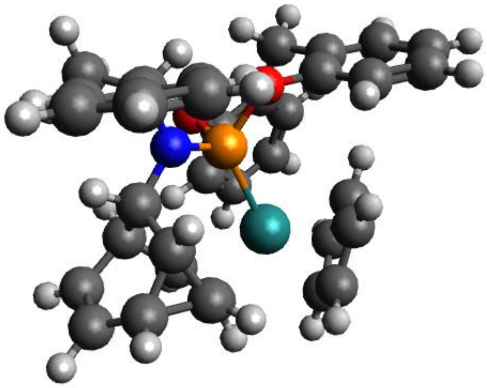

Table S39. Coordinates and energy for the optimised geometry of (R,R,S)-**L**$_{\mathbf{BB1}}$RuCp$^+$.

G = -1994.291459			
	Coordinates / Å		
**Atom**	**x**	**y**	**z**
Ru	-1.09099	-1.27956	-0.65929
P	0.02073	0.33223	0.55113
C	0.66463	-2.69866	-1.10065
C	0.48378	-2.69094	0.26968
C	-3.12027	-1.42683	-1.50157
C	-2.64373	-0.09820	-1.67917
C	-1.47310	-0.17614	-2.49390
C	-1.25808	-1.54711	-2.82012
C	-2.28755	-2.33125	-2.21078
O	-0.64123	0.21251	2.07814
O	-0.16982	1.93002	0.26841
N	1.63390	0.28532	0.80106
C	1.87548	-2.09715	-1.68580
H	0.16803	-3.46170	-1.68746
H	-0.16688	-3.44572	0.70214
C	1.64010	-2.22473	1.14623
H	-3.96369	-1.70174	-0.88536
H	-3.10394	0.79703	-1.29409
H	-0.87497	0.65191	-2.83799
H	-0.46598	-1.91409	-3.45421
H	-2.42385	-3.39790	-2.29376
C	-1.94843	-0.23216	1.99049
C	-1.28584	2.54425	-0.27612
C	2.36969	1.36770	1.50627
C	2.35732	-0.97795	0.58962
C	2.74551	-1.20618	-0.85394
C	3.26037	-2.58937	-1.15027
H	1.85926	-1.91665	-2.75328
H	1.31476	-2.03163	2.16937
C	2.61001	-3.38625	1.07850

C	-2.19275	-1.60426	1.97132
C	-2.97857	0.70626	1.90540
C	-2.47101	2.70008	0.44639
C	-1.14206	3.05216	-1.55980
H	1.70137	2.22866	1.50506
C	2.65057	1.00232	2.96090
C	3.58309	1.75308	0.67546
H	3.28306	-0.88288	1.15904
H	3.23482	-0.37092	-1.34030
H	4.01837	-2.68068	-1.91738
C	3.33887	-3.54759	-0.03009
H	2.64692	-4.09854	1.89446
H	-1.37419	-2.28770	2.15538
C	-3.51011	-2.05989	1.87955
C	-4.27904	0.22295	1.82025
C	-2.65367	2.18298	1.85950
C	-3.52529	3.35156	-0.19655
C	-2.20619	3.69370	-2.17564
H	-0.18827	2.93282	-2.06016
H	3.25990	0.10098	3.06273
H	1.70660	0.83121	3.48074
H	3.17945	1.81994	3.45560
C	3.37410	2.47277	-0.50308
C	4.88006	1.38055	1.01286
H	4.00737	-4.39481	-0.13510
H	-3.71191	-3.12433	1.88985
C	-4.54821	-1.14557	1.80485
H	-5.10122	0.92879	1.76303
H	-1.75012	2.37275	2.44155
H	-3.46720	2.74010	2.32692
H	-4.45783	3.48766	0.34097
C	-3.40811	3.83595	-1.49266
H	-2.09393	4.08388	-3.18007
H	2.36361	2.76860	-0.76825
C	4.43661	2.80768	-1.32905
C	5.94956	1.71045	0.18319
H	5.07233	0.83251	1.92843
H	-5.57359	-1.49056	1.74610
H	-4.24695	4.33732	-1.96005
H	4.26036	3.37501	-2.23613
C	5.73112	2.42170	-0.98849
H	6.95484	1.41254	0.45876
H	6.56419	2.68239	-1.63125

(*S*,*S*,*S*)-$\mathbf{L_{BB1}}$RuCp$^+$

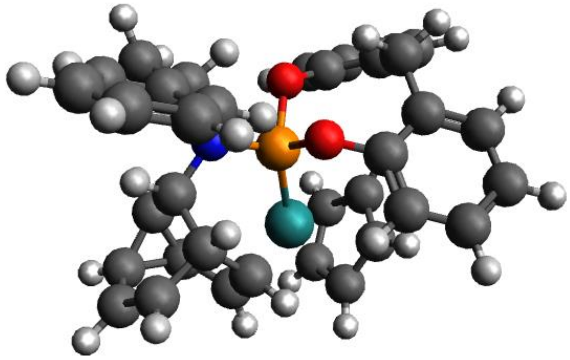

Table S40. Coordinates and energy for the optimised geometry of (*S*,*S*,*S*)-$\mathbf{L_{BB1}}$RuCp$^+$.

G = -1994.299357			
		**Coordinates / Å**	
**Atom**	**x**	**y**	**z**
Ru	-0.88143	1.73231	0.09350
P	0.00040	-0.32813	-0.36817
C	0.68396	3.03603	0.70169
C	1.27743	3.21630	-0.63222
C	-2.87057	2.18707	-0.79504
C	-3.01071	1.08361	0.06997
C	-2.59768	1.48344	1.38203
C	-2.28949	2.88002	1.32535
C	-2.42586	3.30917	-0.01468
O	-0.62599	-1.21118	-1.60192
O	0.01039	-1.44924	0.83771
N	1.56121	-0.28597	-0.86060
C	1.01476	1.87685	1.40867
H	0.27022	3.90776	1.19247
H	0.78520	3.91020	-1.30280
C	2.14562	2.11944	-1.25441
H	-3.07938	2.18958	-1.85367
H	-3.35574	0.09982	-0.20540
H	-2.61291	0.86621	2.26620
H	-1.96629	3.48305	2.16073
H	-2.23632	4.30417	-0.38883
C	-1.97701	-1.32884	-1.86901
C	-1.05432	-1.63973	1.70095
C	2.23062	-1.43481	-1.52391
C	2.39493	0.84753	-0.46807
C	2.31333	1.16092	1.03522
C	3.41383	2.14549	1.36343
H	0.72883	1.81557	2.45363
H	2.08004	2.01018	-2.33000
C	2.85753	3.30948	-0.71911

C	-2.50357	-0.54544	-2.88613
C	-2.75998	-2.23174	-1.15119
C	-2.14194	-2.41545	1.30328
C	-0.96809	-1.09014	2.97227
H	1.49724	-2.24274	-1.53744
C	2.55384	-1.08078	-2.97055
C	3.41272	-1.91593	-0.69015
H	3.42364	0.54210	-0.67116
H	2.42507	0.23398	1.59740
H	3.96544	2.04097	2.29036
C	3.64106	3.15990	0.52360
H	3.19223	4.05648	-1.42706
H	-1.84483	0.12604	-3.42428
C	-3.85604	-0.62940	-3.18447
C	-4.11628	-2.29924	-1.47185
C	-2.17574	-3.09054	-0.05234
C	-3.18334	-2.57498	2.21792
C	-2.01073	-1.27496	3.86937
H	-0.08444	-0.52370	3.23989
H	3.24557	-0.23606	-3.03819
H	1.63089	-0.81236	-3.48681
H	3.00464	-1.93136	-3.48591
C	3.17204	-2.40725	0.59569
C	4.72396	-1.87994	-1.15642
H	4.39179	3.91098	0.74010
H	-4.27400	-0.01775	-3.97534
C	-4.66691	-1.50337	-2.46827
H	-4.74926	-2.99492	-0.93120
H	-1.16284	-3.39268	-0.32721
H	-2.77428	-3.99933	0.03097
H	-4.04212	-3.17558	1.93688
C	-3.12935	-2.00662	3.48428
H	-1.94931	-0.84980	4.86434
H	2.15383	-2.43865	0.96855
C	4.21691	-2.84538	1.39593
C	5.77514	-2.31938	-0.35454
H	4.94309	-1.51114	-2.15152
H	-5.72375	-1.57667	-2.69511
H	-3.94947	-2.15401	4.17690
H	4.01087	-3.22827	2.38930
C	5.52590	-2.80081	0.92268
H	6.78986	-2.28477	-0.73475
H	6.34363	-3.14464	1.54572

TS-(R,R,S)-to-(S,S,S)-$\mathbf{L_{BB1}}$RuCp$^+$ (TS3-Ru)

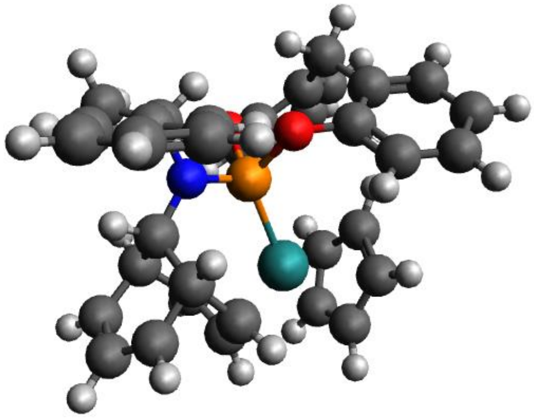

Table S41. Coordinates, energy, and imaginary vibrational frequency for the optimised geometry of TS-(R,R,S)-to-(S,S,S)-$\mathbf{L_{BB1}}$RuCp$^+$ (TS3-Ru).

G = -2127.012482

v = -177.23

Atom	Coordinates / Å x	y	z
Ru	-0.65053	-1.63773	-0.70371
P	-0.02082	0.25176	0.42831
C	0.92646	-3.03745	-0.71124
C	0.80502	-2.81329	0.68415
C	-2.69497	-0.89229	-1.21497
C	-2.06933	-1.35499	-2.40703
C	-1.78896	-2.75459	-2.23912
C	-2.24480	-3.14627	-0.94326
C	-2.79135	-1.99045	-0.29325
O	-0.79200	0.68740	1.93776
O	-0.16916	1.81220	-0.35515
N	1.58468	0.35915	0.88952
C	1.62084	-2.03340	-1.46025
H	0.61162	-3.95755	-1.17803
H	0.30444	-3.56334	1.28295
C	1.95603	-2.07525	1.40171
H	-3.06684	0.10246	-1.04546
H	-1.87168	-0.76511	-3.28298
H	-1.33580	-3.39716	-2.97341
H	-2.18282	-4.13375	-0.51986
H	-3.25213	-1.95820	0.67635
C	-2.17191	0.55729	2.14244
C	-1.27868	2.15593	-1.13585
C	2.17011	1.57949	1.50273
C	2.43215	-0.79620	0.68562
C	2.68198	-1.14157	-0.78551
C	3.60560	-2.27955	-0.91393

H	1.67368	-2.11368	-2.53671
H	1.69391	-1.85431	2.43264
C	3.03720	-3.10607	1.28837
C	-2.62486	-0.55448	2.84439
C	-3.04377	1.55033	1.68677
C	-2.43767	2.64068	-0.52394
C	-1.17698	2.01462	-2.51503
H	1.39638	2.34369	1.43211
C	2.46831	1.34212	2.98196
C	3.34697	2.04079	0.65060
H	3.40231	-0.53038	1.10802
H	2.93335	-0.26841	-1.37609
H	4.16583	-2.39665	-1.83148
C	3.78813	-3.19747	0.14635
H	3.15827	-3.83088	2.08278
H	-1.90305	-1.27963	3.19201
C	-3.98736	-0.71002	3.08064
C	-4.40981	1.36841	1.92814
C	-2.52712	2.78999	0.98090
C	-3.51575	2.96779	-1.35298
C	-2.26365	2.34224	-3.32016
H	-0.25118	1.64595	-2.93338
H	3.17684	0.52519	3.13569
H	1.53893	1.09151	3.49061
H	2.88634	2.24108	3.43639
C	3.08266	2.52082	-0.63946
C	4.67024	1.97281	1.08786
H	4.49777	-4.00166	0.02133
H	-4.34574	-1.56991	3.62877
C	-4.88329	0.24967	2.60957
H	-5.10757	2.12354	1.58984
H	-1.54209	3.03910	1.37508
H	-3.19315	3.62228	1.20960
H	-4.42308	3.35205	-0.90556
C	-3.43887	2.81407	-2.73555
H	-2.19265	2.23456	-4.39331
H	2.05403	2.57310	-0.97445
C	4.12176	2.91854	-1.47371
C	5.71516	2.36924	0.24945
H	4.89954	1.61924	2.08386
H	-5.94331	0.13624	2.78682
H	-4.28686	3.07214	-3.35371
H	3.90373	3.29629	-2.46353
C	5.44457	2.84016	-1.03143
H	6.73577	2.31350	0.60272
H	6.25321	3.15165	-1.67816

9.3 Conformational Search Results

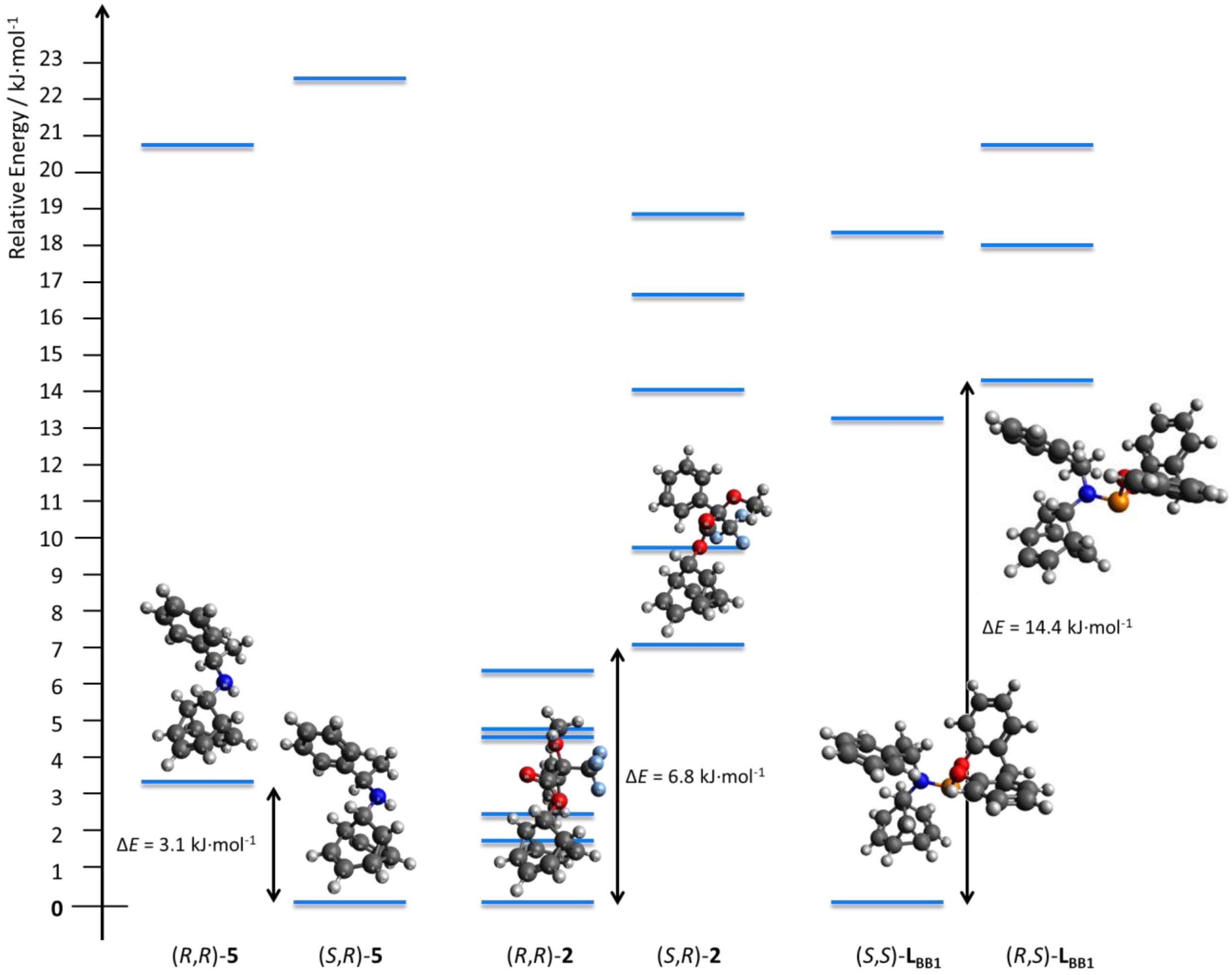

Figure S96. Relative energies of DFT optimised conformers of **2**, **5**, and $\mathbf{L_{BB1}}$. The geometries of the minimum energy structures are shown.

10. References

[1] Raskatov, J. A.; Brown, J. M.; Thompson, A. L. *CrystEngComm*. **2011**, *13*, 2923.
[2] Ruggen, M.; Carreria, E. M. *Angew. Chem. Int. Ed*. **2011**, *50*, 5568.
[3] Chen, C.; Xu, R.; Li, B. *Science China Chemistry*. **2012**, *55*, 1257.
[4] Einaru, S.; Shitamichi, K.; Nagano, T.; Matsumoto, A.; Asano, K.; Matsubara, S. *Angew. Chem. Int. Ed*. **2018**, *57*, 13863.
[5] Barrett, S.; O'Brien, P.; Steffens, H. C.; Towers, T. D.; Voith, M. *Tetrahedron.* **2000**, *56*, 96333.
[6] Dolomanov, O. V.; Bourhis, L. J.; Gildea, R. J.; Howard, J. A. K.; Puschmann, H. *J. Appl. Crystallogr.* **2009**, *42*, 339.
[7] Sheldrick, G. M. Acta Crystallogr. Sect. A Found. *Crystallogr.* **2008**, *64*, 112.
[8] Ferrer, S.; Echavarren, A. M. *Angew. Chem. Int. Ed*. **2016**, *55*, 11178.
[9] Dale, J. A.; Dull, D. A.; Mosher, H. S. *J. Org. Chem.* **1969**, *34*, 2543.
[10] Nomenclature of Inorganic Chemistry, IUPAC Recommendations 2005; Connelly, N. G.; Damhus. T.; Hartshorn, R. M.; Hutton, A. T., Eds.; RSC Publishing: Cambridge, 2005.
[11] Bauer, E. B. *Chem. Soc. Rev.* **2012**, *41*, 3153.
[12] Xu, R.; Li, K.; Wang, J.; Lu, J.; Pan, L.; Zeng, X.; Zhong, G. *Chem. Commun.* **2020**, *56*, 8404.
[13] Rössler, S. L.; Petrone, D. A.; Carreria. E. *Acc. Chem. Res*. **2019**, *52*, 2657.
[14] Cheng, Q.; Tu, H.-F.; Zheng, C.; Qu, J.-P.; Helmchen, G.; You, S.-L. *Chem. Rev.* **2019**, *119*, 1855.
[15] Rössler, S. L.; Krautwald, S.; Carreria. *J. Am. Chem. Soc*. **2017**, *139*, 3603.
[16] McGonigal, P. R.; de León, C.; Wang, Y.; Homs, A.; Solorio-Alvarado, C. R.; Echavarren, A. M. *Angew. Chem. Int. Ed*. **2012**, *51*, 13093.
[17] Williams, A. T. R.; Winfield, S. A.; Miller, J. N. *Analyst.* **1983**, *108*, 1067.
[18] Melhuish, W. H. *J. Phys. Chem.* **1961**, *65*, 229.
[19] https://www.horiba.com/en_en/applications/materials/material-research/quantum-dots/recording-fluorescence-quantum-yields/ (accessed February 2021)
[20] Guassian 16, Revision A.03, Frisch, M. J.; Trucks, G. W.; Schlegel, H. B.; Scuseria, G. E.; Robb, M. A.; Cheeseman, J. R.; Scalmani, G.; Barone, V.; Petersson, G. A.; Nakatsuji, H.; Li, X.; Caricato, M.; Marenich, A. V.; Bloino, J.; Janesko, B. G.; Gomperts, R.; Mennucci, B.; Hratchian, H. P.; Ortiz, J. V.; Izmaylov, A. F.; Sonnenberg, J. L.; Williams-Young, D.; Ding, F.; Lipparini, F.; Egidi, F.; Goings, J.; Peng, B.; Petrone, A.; Henderson, T.; Ranasinghe, D.; Zakrzewski, V. G.; Gao, J.; Rega, N.; Zheng, G.; Liang, W.; Hada, M.; Ehara, M.; Toyota, K.; Fukuda, R.; Hasegawa, J.; Ishida, M.; Nakajima, T.; Honda, Y.; Kitao, O.; Nakai, H.; Vreven, T.; Throssell, K.; Montgomery, J. A., Jr.; Peralta, J. E.; Ogliaro, F.; Bearpark, M. J.; Heyd, J. J.; Brothers, E. N.; Kudin, K. N.; Staroverov, V. N.; Keith, T. A.; Kobayashi, R.; Normand, J.; Raghavachari, K.; Rendell, A. P.; Burant, J. C.; Iyengar, S. S.; Tomasi, J.; Cossi, M.; Millam, J. M.; Klene, M.; Adamo, C.; Cammi, R.; Ochterski, J. W.; Martin, R. L.; Morokuma, K.; Farkas, O.; Foresman, J. B.; Fox, D. J. Gaussian, Inc., Wallingford CT, 2016.
[21] Chai, J.-D.; Head-Gordon, M. *Phys. Chem. Chem. Phys.* **2008**, *10*, 6615.
[22] Bismillah, A. N.; Sturala, J.; Chapin, B. M.; Yufit, D. S.; Hodgkinson, P.; McGonigal, P. R. *Chem. Sci.* **2018**, *9*, 8631.
[23] Fukui, K. *Acc. Chem. Res.* **1981**, *14*, 363.
[24] Hratchian, H.P.; Schlegel, H. B. in *Theory and Applications of Computational Chemistry: The First 40 Years*, Ed. C. E. Dykstra, G. Frenking, K. S. Kim, and G. Scuseria (Elsevier, Amsterdam, 2005) 195–249.
[25] O'Boyle, N. M; Vandermeersch, T.; Flynn, C. J.; Maguire, A. R.; Hutchinson, G. R. J. *Cheminf.* **2011**, *3*, 8.
[26] O'Boyle, N. M; Banck, M.; James, C. A.; Morley, C.; Vandermeersch, T.; Hutchinson, G. R. J. *Cheminf.* **2011**, *3*, 33.
[27] The Open Babel Package, version 3.0.0 http://openbabel.org (accessed October 2019).
[28] Günther, H,; Runsink, J.; Schmickler, H; Schmitt. P. *J. Org. Chem.* **1985**, *50*, 289.
[29] Engdahl, C.; Ahlberg, P. *J. Am. Chem. Soc.* **1979**, *101*, 3940.
[30] Clark, S. J.; Segall, M. D.; Pickard, C. J.; Hasnip, P. J.; Probert, M. J.; Refson, K.; Payne, M. C. Z. *Kristallogr.* **2005**, *220*, 567.
[31] Perdew, J. P.; Burke, K.; Ernzerhof, M. *Phys. Rev. Lett.* **1996**, *77*, 3865.
[32] Sturniolo, S.; Green, T. F. G.; Hanson, R. M.; Zilka, M.; Refson, K.; Hodgkinson, P.; Brown, S. P.; Yates, J. R. *Solid State Nucl. Magn. Reson.* **2016**, *78*, 64.
